# Porcine circovirus type 2 infection promotes the SUMOylation of nucleophosmin-1 to facilitate the viral circular single-stranded DNA replication

**DOI:** 10.1371/journal.ppat.1012014

**Published:** 2024-02-23

**Authors:** Qian Du, Lei Zhu, Jianhui Zhong, Xueqi Wei, Qi Zhang, Tengfei Shi, Cong Han, Xinhuan Yin, Xingqi Chen, Dewen Tong, Yong Huang

**Affiliations:** 1 College of Veterinary Medicine, Northwest A&F University, Yangling, China; 2 Engineering Research Center of Efficient New Vaccines for Animals, Ministry of Education, Yangling, China; 3 Key Laboratory of Ruminant Disease Prevention and Control (West), Ministry of Agriculture and Rural Affairs, Yangling, China; 4 Engineering Research Center of Efficient New Vaccines for Animals, Universities of Shaanxi Province, Yangling, China; 5 Department of Immunology, Genetics and Pathology, Uppsala University and Science for Life Laboratory, Uppsala, Sweden; Pirbright Institute, UNITED KINGDOM

## Abstract

The mechanism of genome DNA replication in circular single-stranded DNA viruses is currently a mystery, except for the fact that it undergoes rolling-circle replication. Herein, we identified SUMOylated porcine nucleophosmin-1 (pNPM1), which is previously reported to be an interacting protein of the viral capsid protein, as a key regulator that promotes the genome DNA replication of porcine single-stranded DNA circovirus. Upon porcine circovirus type 2 (PCV2) infection, SUMO2/3 were recruited and conjugated with the K263 site of pNPM1’s C-terminal domain to SUMOylate pNPM1, subsequently, the SUMOylated pNPM1 were translocated in nucleoli to promote the replication of PCV2 genome DNA. The mutation of the K263 site reduced the SUMOylation levels of pNPM1 and the nucleolar localization of pNPM1, resulting in a decrease in the level of PCV2 DNA replication. Meanwhile, the mutation of the K263 site prevented the interaction of pNPM1 with PCV2 DNA, but not the interaction of pNPM1 with PCV2 Cap. Mechanistically, PCV2 infection increased the expression levels of Ubc9, the only E2 enzyme involved in SUMOylation, through the Cap-mediated activation of ERK signaling. The upregulation of Ubc9 promoted the interaction between pNPM1 and TRIM24, a potential E3 ligase for SUMOylation, thereby facilitating the SUMOylation of pNPM1. The inhibition of ERK activation could significantly reduce the SUMOylation levels and the nucleolar localization of pNPM1, as well as the PCV2 DNA replication levels. These results provide new insights into the mechanism of circular single-stranded DNA virus replication and highlight NPM1 as a potential target for inhibiting PCV2 replication.

## Author summary

Different types of DNA viruses employ different mechanisms to replicate their genome DNA. Porcine circovirus type 2 (PCV2) is the most representative circular single-stranded DNA virus that harms the pig industry all over the world. In this study, we found that the PCV2 Cap interacting protein pNPM1 also interacts with PCV2 DNA in a SUMOylated form to promote PCV2 DNA replication. The SUMOylation of pNPM1 at the conserved K263 site is critical for the interaction of pNPM1 with PCV2 DNA and the replication of PCV2 DNA. Furthermore, we found that PCV2 infection promotes the SUMO2/3 mediated SUMOylation of pNPM1, while does not significantly alter the expression level of pNPM1. PCV2 Cap is the major component that promotes pNPM1 SUMOylation by activating ERK/Ubc9/TRIM24 signalings. These results contribute to a better understanding of the replication mechanism of circular single-stranded DNA viruses, particularly PCV2.

## Introduction

DNA viruses, especially small-size DNA viruses, lack the necessary enzyme system for replication, which usually employs cellular DNA replication machinery to replicate their genomes in the nucleus along with the cellular DNA replication or repair processes [1]. Cellular DNA replication and repair are multiple-step highly regulated events and involve several mechanisms including many proteins to ensure fidelity and coordination with surrounding chromatin [1,2]. Thus, different viruses employ different host proteins to replicate their DNA. For adeno-associated virus (AAV, a linear single-stranded DNA virus), the viral DNA replication needs replication factor C (RFC), replication protein A (RPA), proliferating cell nuclear antigen (PCNA), DNA polymerase δ, and the minichromosome maintenance (MCM) complex [3]. For adenovirus (AdV, a linear double-stranded DNA virus), the viral DNA replication needs the transcription activating factor (TAF)-Iβ (also known as SET), TAF-II (NAP-1), and TAF-III (B23/nucleophosmin) [4]. For simian virus 40 (SV40, a circular double-stranded DNA virus), the viral DNA replication needs RPA, Topoisomerase I, and DNA polymerase α-primase (Pol-prim) complex for initiation [5]. Yet, for circular single-stranded DNA viruses like porcine circovirus type 2 (PCV2, one of the smallest mammalian viruses), little is known about the mechanism of viral DNA replication except that it undergoing the replicase complex (Rep/Rep’) associated rolling-circle replication [6,7].

The post-translational modification (PTM) of proteins is a crucial regulatory mechanism in the control of cellular processes. The small ubiquitin-like modifier (SUMO) modification (SUMOylation) is a broadly described PTM of proteins that not only mediates protein degradation, but also regulates protein activity, subcellular localization, and protein-protein interactions for a variety of cellular activities [8]. During cellular DNA replication and repair, SUMOylation plays a prominent role in modifying host proteins, and is involved in every step from origin licensing to the eviction of the replisome upon DNA replication termination [9–11]. SUMOylation is also reported to participate in some DNA virus replication, for example, AdV E1A enhances cellular factor DREF (ZBED1) SUMOylation to regulate viral DNA replication [12], Kaposi’s sarcoma-associated herpesvirus (KSHV, same family as AdV) infection enhances the SUMOylation of the host 90-kDa ribosomal S6 kinases 1 (RSK1) to promote viral DNA replication [13], Human papillomaviruses (HPV, a circular double-stranded DNA virus) L2 interacts with SUMO and/or SUMOylated proteins to deliver viral DNA to promyelocytic leukemia nuclear bodies for transcription and replication [14]. Our previous studies have reported that PCV2 infection induces PTMs of proteins involved in replication or pathogenesis, that its infection can promote the phosphorylation and ubiquitination of cGAS for degradation resulting in inhibition of type I interferon induction [15], downregulate E3 ubiquitin ligase family member Makorin RING finger protein (MKRN1) in cells to avoid MKRN1-mediated Cap ubiquitination and degradation promoting viral replication and pathogenesis [16,17], employ host C1q receptor gC1qR to facilitate viral nuclear egress by activating and recruiting of PKC-δ to phosphorylate lamin A/C [18]. Therefore, it is reasonable considering that PCV2 would employ the host SUMOylation system to promote viral DNA replication.

In this present study, we initially used a biotin-labeled PCV2 DNA to capture the interacting host proteins in PK-15 cells at the early stage of infection, then used an anti-SUMO antibody to precipitate the SUMOylated proteins interacting with PCV2 DNA. The precipitated proteins were analyzed by LC-MS/MS, and the PCV2 Cap interacting protein porcine nucleophosmin-1 (pNPM1) was identified. Further results confirmed the interaction of PCV2 DNA with pNPM1, and the SUMOylation site of pNPM1 at K263 of the C-terminal domain, which was found critical for the interaction of PCV2 DNA with pNPM1, but not for the interaction of PCV2 Cap with pNPM1. We also found that the SUMOylation at the K263 site was important for the nucleolar localization of pNPM1, and the SUMOylated pNPM1 participated in the regulation of PCV2 DNA replication in nucleoli. Furthermore, we found Cap was the major component of PCV2 to promote the SUMOylation of pNPM1 through activating the ERK/Ubc9/TRIM24 signalings. These results provide new insights into the roles of SUMOylation of pNPM1 during PCV2 DNA replication and lay a foundation for further elucidation of the PCV2 replication mechanism.

## Results

### PCV2 DNA interacts with SUMOylated pNPM1 in PK-15 cells

**Fig 1:**
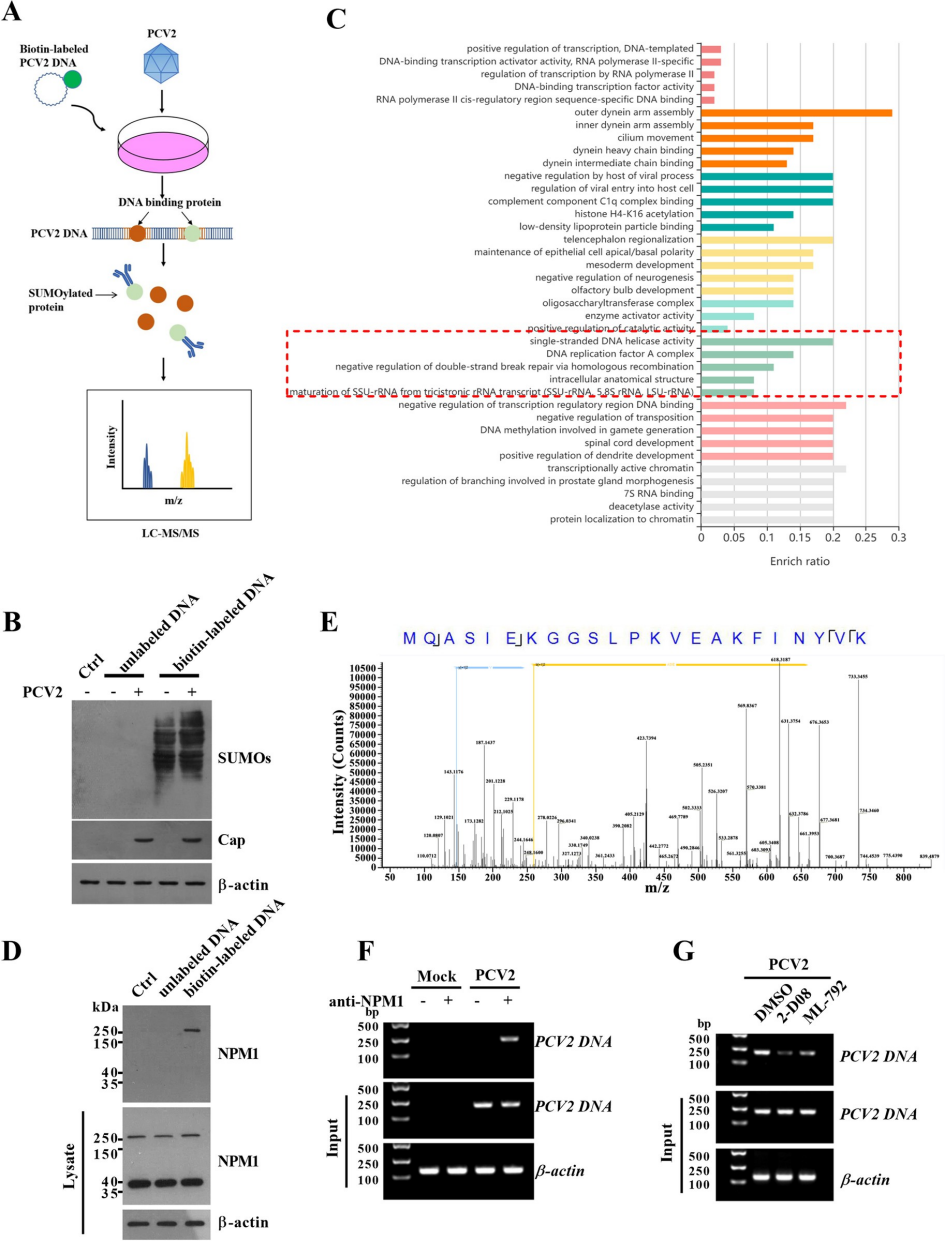
PCV2 DNA interacts with SUMOylated host protein NPM1. (**A**) Schematic diagram of the experiment design. The biotin-labeled PCV2 DNA was transfected into PK-15 cells for 24 h, and then the cells were infected with 1 MOI PCV2 or mock for 12 h. The PCV2 DNA binding host proteins were pulled down using streptavidin-conjugated beads. The PCV2 DNA pulled-down proteins were further immunoprecipitated with an anti-Pan SUMO antibody, and the precipitated proteins were analyzed by Liquid Chromatography-Mass Spectrometer/Mass Spectrometry (LC-MS/MS). (**B**) The PCV2 DNA pulled-down proteins were detected for the SUMOylation by western blot. (**C**) The SUMOylated proteins interacting with PCV2 DNA were analyzed using KEGG enrichment and GO enrichment analysis on KOBAS (http://kobas.cbi.pku.edu.cn/). Each row represents an enriched function, and the length of the bar represents the enrichment ratio. The color of the bar represents different clusters. For each cluster, if there are more than 5 terms, the top 5 with the highest enrich ratio are displayed. (**D**) The PCV2 DNA pulled-down proteins were detected using anti-NPM1 antibodies by western blot. (**E**) The anti-NPM1 antibody detected protein was analyzed by LC-MS/MS. One of the typical peptide fragments (251 aa-273 aa) was shown. (**F**) PK-15 cells were infected with 1 MOI PCV2 for 12 h, and then the NPM1 interacting DNA was analyzed by ChIP assays using PCV2 DNA-specific primers. (**G**) PK-15 cells were pre-treated with the SUMOylation inhibitors 2-D08 (200 μM) or ML-792 (10 μM) for 24 h, and then the cells were infected with 1 MOI PCV2 for 12 h with the presence of 2-D08 or ML-792. The NPM1 interacting DNA was analyzed by ChIP assays using PCV2 DNA-specific primers.

To identify which SUMOylated host proteins are involved in regulating PCV2 DNA early replication, we transfected biotin-labeled PCV2 DNA or unlabeled PCV2 DNA into PK-15 cells, then infected the cells with 1 MOI PCV2 for 12 h. The PCV2 DNA interacting host proteins were pulled down using beads conjugating with streptavidin (Fig 1A). To first confirm that there were indeed SUMOylated proteins interacting with PCV2 DNA, we detected the pull-down proteins with an anti-SUMO antibody, and the results showed that SUMOs could be detected in the biotin-labeled PCV2 DNA pull-down proteins, confirming that SUMOylated proteins indeed interacted with the PCV2 DNA (Fig 1B). Then we used the anti-SUMO antibody to precipitate the SUMOylated PCV2 DNA interacting proteins and identified the precipitated proteins by LC-MS/MS. The Kyoto Encyclopedia of Genes and Genomes (KEGG) enrichment analysis combined with the Gene Ontology (GO) enrichment analysis results showed that the identified proteins are highly associated with DNA replication, including single-stranded DNA helicase activity and DNA replication factor A complex, etc. (Fig 1C). Among these PCV2 DNA interacting SUMOylated proteins, we noticed the previously reported PCV2 Cap interacting protein porcine nucleophosmin-1 (pNPM1), which is also known to play important roles in host cellular DNA replication [19]. To confirm the interaction of PCV2 DNA and pNPM1, we detected the PCV2 DNA pulled-down SUMOylated proteins using an anti-NPM1 antibody, and the results showed that NPM1 could be detected in the PCV2 DNA pulled-down SUMOylated proteins with a molecular weight higher than 250 kDa, which were much higher than the regular molecular weight of pNPM1 (~38 kDa) (Fig 1D), and this anti-NPM1 antibody-detected higher molecular weight band was confirmed to be pNPM1 by LC-MS/MS (Fig 1E). Meanwhile, we also detected the interaction of pNPM1 and PCV2 DNA by ChIP assays, and the results showed that pNPM1 could interact with PCV2 DNA in PCV2-infected PK-15 cells (Fig 1F). Furthermore, to make clear if the SUMOylation of pNPM1 is critical for the interaction of pNPM1 and PCV2 DNA, we pretreated PK-15 cells with the inhibitors of SUMOylation (2-D08 or ML-792), then infected the cells with 1 MOI PCV2 for 12 h. The ChIP results showed that inhibition of the SUMOylation in cells significantly reduced the interaction of pNPM1 and PCV2 DNA (Fig 1G). These results indicate that PCV2 DNA interacts with multiple SUMOylated proteins in PCV2-infected PK-15 cells, including the previously reported PCV2 Cap interacting host protein pNPM1.

### The C-terminal K263 site is critical for the SUMOylation of pNPM1 and its interaction with PCV2 DNA but not for its interaction with PCV2 Cap

**Fig 2:**
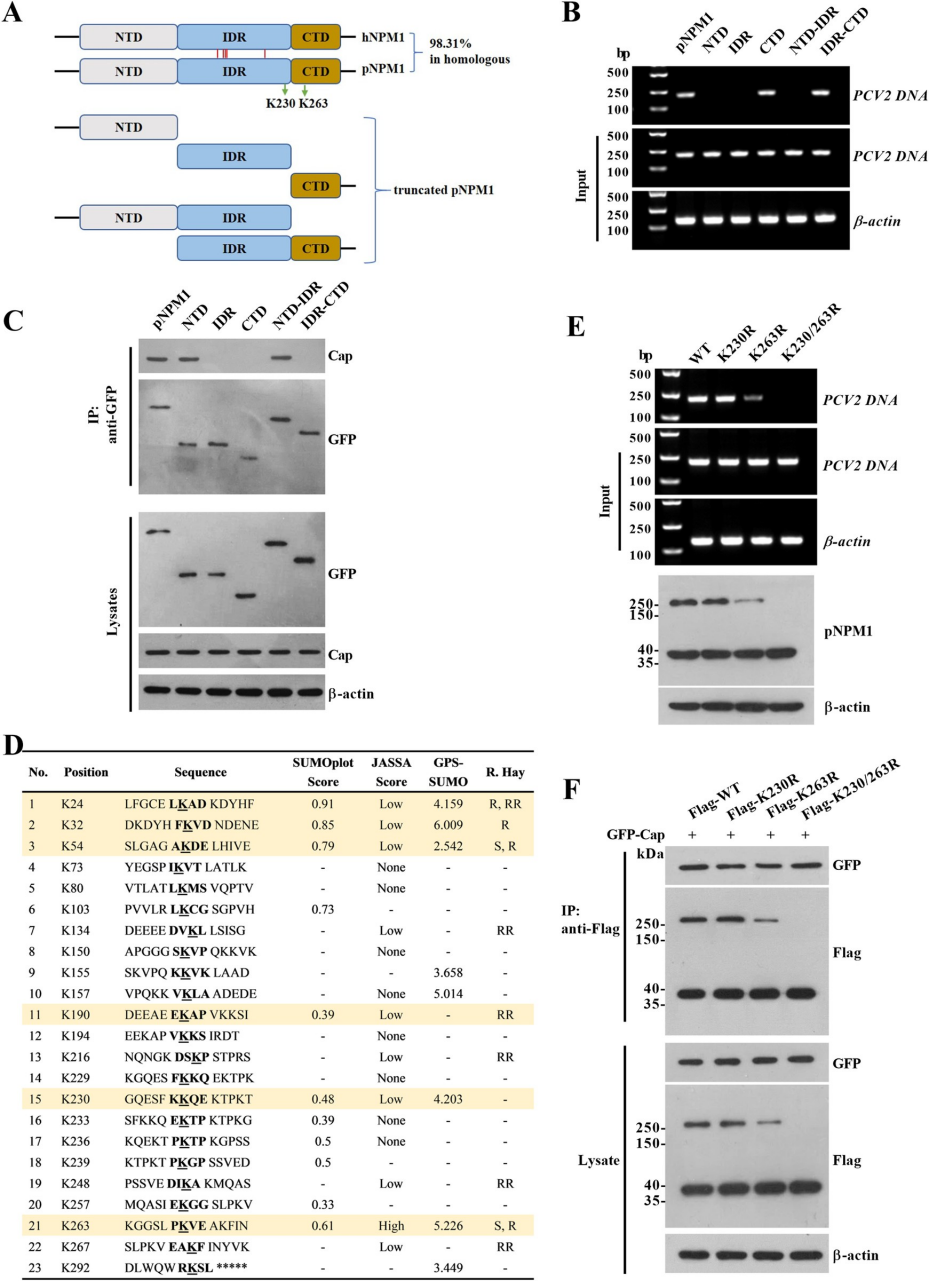
The K263 SUMOylation site on the C-terminal domain of porcine NPM1 is critical for the interaction of porcine NPM1 and PCV2 DNA. (**A**) Schematic diagram of the structure of human NPM1 (hNPM1) and porcine NPM1 (pNPM1), as well as the truncates of pNPM1. The red lines indicate the sites of the different amino acids between hNPM1 and pNPM1. The green arrows indicate the K230 and K263 SUMOylation sites on pNPM1. (**B, C**) Equal amounts of plasmids expressing the GFP-fused pNPM1 truncates were transfected into the pNPM1 knockout PK-15 (PK-15^*npm1-/-*^) cells for 24 h, then the cells were infected with 1 MOI PCV2 for 12 h. (**B**) The interaction of the pNPM1 truncates with PCV2 DNA was measured by ChIP assays. (**C**) The interaction of the pNPM1 truncates with PCV2 Cap was measured by co-IP assays. (**D**) The potential SUMOylation sites on pNPM1 were predicted by four online software, including SUMOplot (https://www.abcepta.com/sumoplot), JASSA (http://www.jassa.fr/), GPS-SUMO (http://sumosp.biocuckoo.org/), and Ron Hay (https://www.lifesci.dundee.ac.uk/groups/ron_hay/pages/SumomotifQuery.html). Details of the SUMO conjugation motifs and a summary of prediction software scores are depicted in the table. S = strict, R = relaxed, and RR = relaxed reverted in Ron Hay analysis. The short horizontal lines in the table represent have not been predicted by the analysis. (**E**) The same amount of the SUMOylation sites mutant pNPM1 expressing plasmids (K230R, K263R, and K230/263R) were transfected into PK-15^*npm1-/-*^ cells for 24 h, and then the cells were infected with 1 MOI PCV2 for 12 h. The interactions of the SUMOylation sites mutated pNPM1 with PCV2 DNA were measured by ChIP assays. (**F**) The plasmids expressing Flag-tag labeled SUMOylation sites mutated pNPM1 as well as the GFP-tag labeled PCV2 Cap were transfected into PK-15^*npm1-/-*^ cells for 24 h, and then the interaction of the SUMOylation sites mutated pNPM1 with PCV2 Cap were measured by co-IP assays.

NPM1 is a multifunctional protein participating in the different life processes of cells [20], and NPM1 structurally comprises distinct functional domains that mediate multifaceted roles in different cellular events [21]. To figure out which domain of pNPM1 is critical for the interaction of pNPM1 with PCV2 DNA, we first aligned the amino acid sequences of porcine NPM1 (GenBank no. XP_013846116) and human NPM1 (hNPM1, GenBank no. AAA58386 and AAW67758), and the results showed that the amino acid sequence of pNPM1 was 98.31% homologous to that of hNPM1 (Fig 2A). Then we constructed a serial of truncates of pNPM1 according to the structure of hNPM1, including the N-terminal domain (NTD) of pNPM1 (1–119 aa), central intrinsically disordered region (IDR) of pNPM1 (120–242 aa), the C-terminal domain (CTD) of pNPM1 (243–294 aa), NTD-IDR (1–242 aa), and IDR-CTD (120–294 aa), and all these truncates were fused with GFP (Fig 2A). Besides, we previously generated a pNPM1 knockout PK-15 cell line (PK-15^*npm1-/-*^) using the CRISPR/Cas9 system [22]. We transfected the PK-15^*npm1-/-*^ cells with GFP-fused pNPM1 truncates for 24 h and then infected the cells with 1 MOI PCV2 for 12 h, respectively. The ChIP results showed that the CTD and IDR-CTD truncates interacted with PCV2 DNA, but NTD, IDR, and NTD-IDR did not (Fig 2B). Meanwhile, we checked the interaction between pNPM1 truncates and PCV2 Cap through IP assays. The results showed that NTD and NTD-IDR truncates interacted with Cap, while IDR, CTD, and IDR-CTD did not (Figs 2C and S1). These results show that the C-terminal domain of pNPM1 is responsible for the interaction of pNPM1 with PCV2 DNA, while the N-terminal domain of pNPM1 is responsible for the interaction of pNPM1 with PCV2 Cap.

To further investigate the SUMOylation sites of pNPM1, we predicted the potential SUMO-conjugation consensus motifs on pNPM1 using four bioinformatics analysis (SUMOplot, JASSA, GPS-SUMO, and R. Hay), and the results showed that there are twenty-three potential SUMO-conjugation consensus motifs in pNPM1, while of which only six sites (K24, K32, K54, K190, K230, and K263) were predicted by three or more analysis (Fig 2D). Further, considering that the K190 site is predicted through the Relaxed Reverted Criteria of R. Hay analysis and its low scores in both SUMOplot and JASSA analysis, as well as there is a previous study has shown that only K230 and K263 sites, but not K24, K32, and K54 sites, can be effectively SUMOylated in hNPM1 [23], we considered the K230 and K263 to be more plausible SUMOylation sites of pNPM1 (Fig 2A). Thus, we constructed the K230 and K263 SUMOylation sites single and dual mutant pNPM1 expressing vectors (K230R, K263R, and K230/263R), and then transfected these expressing vectors into pNPM1 knockout PK-15 (PK-15^*npm1-/-*^) cells followed with PCV2 infection for 12 h. The ChIP results showed that the mutation of the K230 site could not visibly change the interaction between pNPM1 and PCV2 DNA, yet the mutation of the K263 site or both K230/K263 sites significantly reduced the interaction between pNPM1 and PCV2 DNA (Fig 2E). However, the co-IP results showed that the mutations of the K230 site, K263 site, or both K230/K263 sites all did not reduce the levels of PCV2 Cap protein interacted with pNPM1 when precipitated using pNPM1 antibodies, although the mutation of the K263 site, especially both K230/K263 sites, significantly reduced the levels of the pNPM1 with a molecular weight higher than 250 kDa in PK-15 cells (Fig 2F). These results further indicate that compared with the K230 site, the K263 site of pNPM1 plays a more important role in the SUMOylation of pNPM1 as well as the interaction of pNPM1 and PCV2 DNA, but not for the interaction of pNPM1 and PCV2 Cap.

### SUMOylation determines the nucleolar localization of pNPM1 which is critical for PCV2 DNA replication

**Fig 3:**
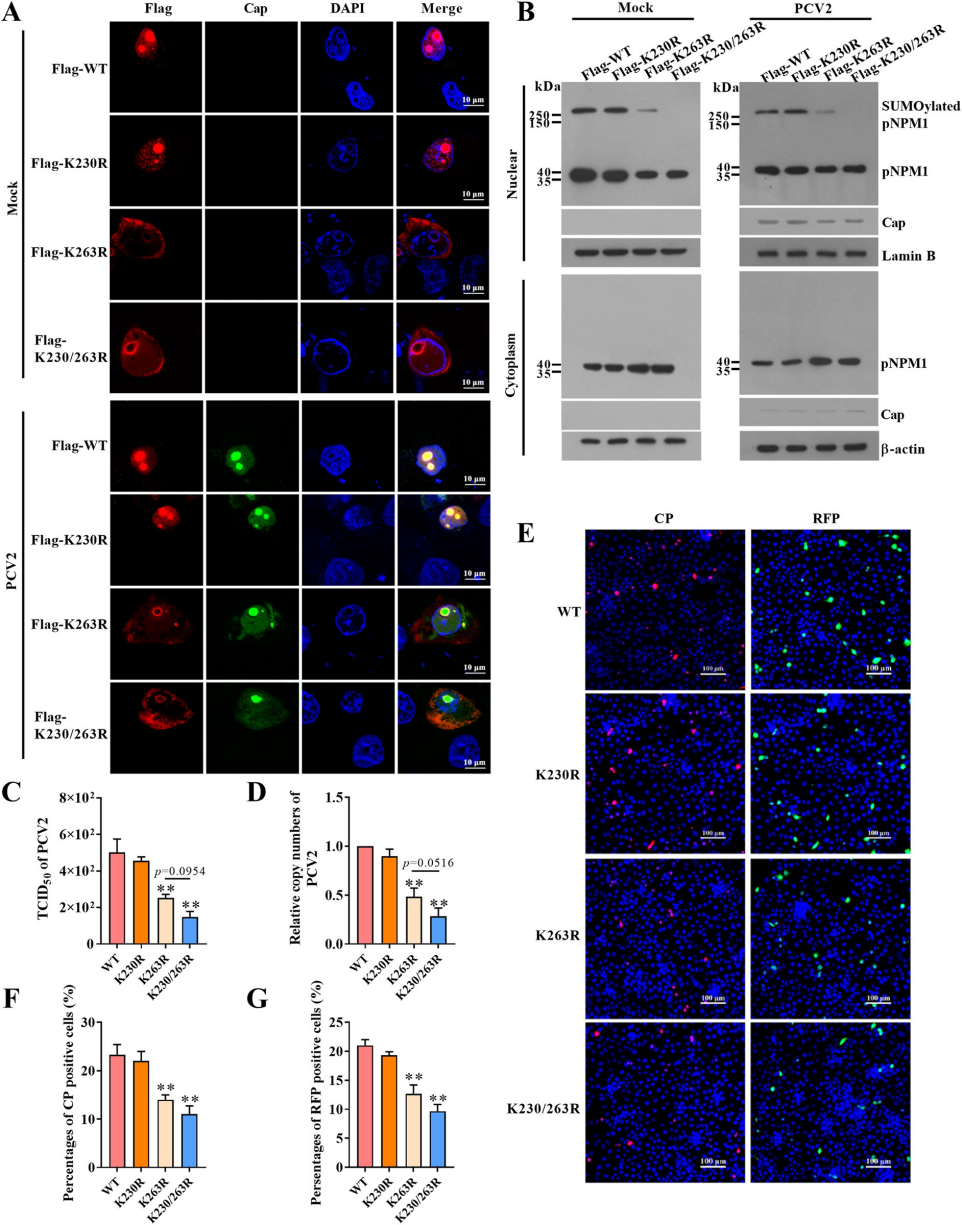
The SUMOylation facilitates the nucleoli localization of pNPM1 that promotes PCV2 DNA replication. PK-15^*npm1-/-*^ cells were transfected with equal amounts of the wild-type pNPM1, pNPM1 (K230R), pNPM1 (K263R), or pNPM1 (K230/263R) for 24 h, respectively. Then the cells were infected with 1 MOI PCV2 for 12 h. (**A**) The localizations of the wild-type pNPM1 (WT), pNPM1 (K230R), pNPM1 (K263R), or pNPM1 (K230/263R) were measured using confocal microscopy. The bars = 10 μm were indicated in each panel. (**B**) The cells were harvested and lysed with a nuclear and cytoplasmic protein extraction kit, then the expression of the pNPM1 and Cap were measured by western blot. (**C**) The PCV2 TCID_50_ of these cells were measured and calculated. (**D**) The copy numbers of PCV2 DNA were detected by qPCR and calculated. (**E, F, G**) The PCV2 genomes (CP) or replication form DNA (RFP) were detected using fluorescently-labeled specific DNA probes. (**E**) The positive cells were photographed by a fluorescence microscope. The bars = 100 μm were indicated in each panel. (**F**) The percentages of CP-positive cells were quantified per 500 cells. (**G**) The percentages of RFP-positive cells were quantified per 500 cells. **p < 0.01 versus the wild-type pNPM1 transfected cells in (C), (D), (F), and (G).

The PTMs of NPM1 are reported critical for its localization in cells [24], to investigate whether the SUMOylation of pNPM1 induced by PCV2 infection influences the localization of pNPM1 in PK-15 cells, we transfected PK-15^*npm1-/-*^ cells with wild-type pNPM1, pNPM1(K230R), pNPM1(K263R), and pNPM1(K230/263R), respectively, and then infected the cells with 1 MOI PCV2 or mock for 12 h. The results showed that whether PCV2 infected or not, in the wild-type pNPM1 transfected cells, pNPM1 was mainly localized in the nucleoli of cells in which Cap major localized (Fig 3A). In the pNPM1(K230R) transfected cells, the localization of pNPM1 was not significantly changed (Fig 3A). However, in the pNPM1(K263R) and pNPM1(K230/263R) transfected cells, pNPM1 were majorly distributed out of the nucleoli of cells, and even in the cytoplasm of the cells (Fig 3A). Further, we separated the cytoplasmic and nuclear fractions of the wild-type pNPM1, K230R, K263R, and K230/263R transfected PK-15^*npm-/-*^ cells, then analyzed the levels of pNPM1 by western blotting, they showed similar results of the IFA assays that the mutation of K263 led pNPM1 locating out of the nucleoli of cells, and even in the cytoplasm (Fig 3B). Furthermore, although the previous study has found that pNPM1 participating in the regulation of PCV2 replication, the mechanism is still not clear [25]. To clarify the role of nucleolar localization of SUMOylated pNPM1 in PCV2 replication, we transfected PK-15^*npm1-/-*^ cells with the same doses of wild-type pNPM1, pNPM1(K230R), pNPM1(K263R), and pNPM1(K230/263R), respectively, followed by PCV2 infection at 1 MOI for 12 h. TCID_50_ results showed the viral titers were similar level between in the PCV2-infected pNPM1(K230R) transfected cells and in the PCV2-infected wild-type pNPM1 transfected cells, while the viral titers were significantly lower in the PCV2-infected pNPM1(K263R) and pNPM1(K230/263R) transfected cells (Fig 3C). The detection of the PCV2 DNA copy numbers in these cells showed similar results (Fig 3D). Further, we used DNA probes for PCV2 genome (CP) or replication form (RFP) to analyze the replication of PCV2 in these cells with FISH assay. The results showed that the CP and RFP forms of PCV2 DNA could be all detected in these cells (Fig 3E). Similar to the above results, compared with that in the wild-type pNPM1 transfected cells, the numbers of CP and RFP forms in the pNPM1(K230R) transfected cells did not significantly change, whereas that decreased in the pNPM1(K263R) and pNPM1(K230/263R) transfected cells (Fig 3E, 3F and 3G). These results indicate that the SUMOylation of pNPM1 is critical for its nucleolar localization and mediating PCV2 DNA replication.

### PCV2 infection promotes conjugation of SUMO2/3 with pNPM1 and nucleolar localization of SUMO2/3-conjugated pNPM1

**Fig 4:**
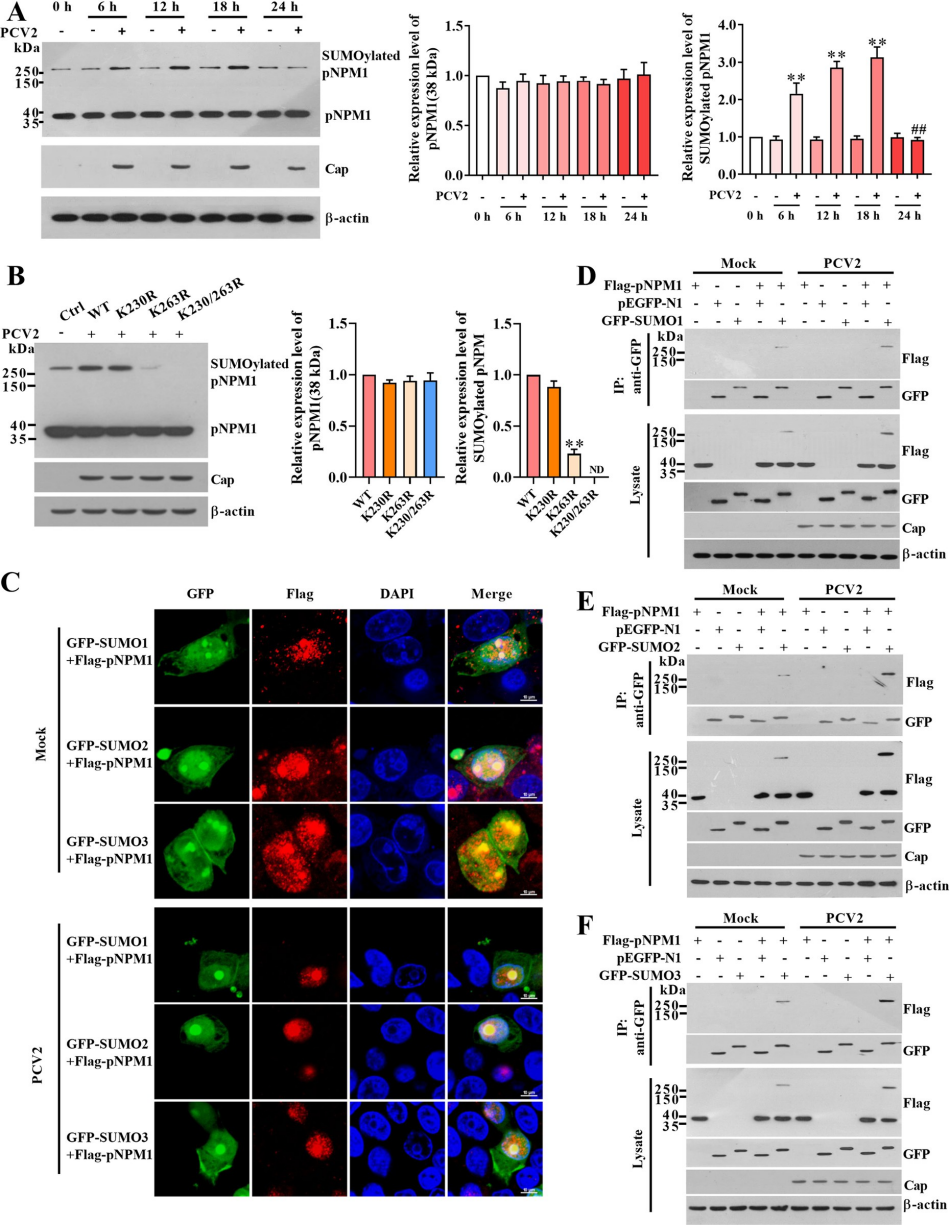
PCV2 infection promotes SUMO2/3 binding to pNPM1 within 24 h. (**A**) PK-15 cells were infected with 1 MOI PCV2 or mock (the same volume of medium) for 0 h, 6 h, 12 h, 18 h, and 24 h. The expression levels of pNPM1, SUMOylated pNPM1, and PCV2 Cap were detected by western blot. The expression levels of pNPM1 and SUMOylated pNPM1 relative to β-actin were calculated. (**B**) Equal amounts of plasmids expressing the pNPM1 SUMOylation site mutants were transfected into PK-15^*npm1-/-*^ cells for 24 h, and then the cells were infected with PCV2 for 12 h. The expression levels of pNPM1 and SUMOylated pNPM1 were detected by western blot and calculated. (**C, D, E, F**) The PK-15^*npm1-/-*^ cells were transfected with Flag-tagged pNPM1 and GFP-fused SUMO proteins, respectively. Then the cells were infected with mock or 1 MOI PCV2 for 12 h. (**C**) The localizations of pNPM1 and SUMO proteins in these cells were detected by confocal microscopy. The bars were indicated in each panel. The interactions of pNPM1 and SUMO1 (**D**), SUMO2 (**E**), or SUMO3 (**F**) were measured by IP assays, the pEGFP-N1 represents the vector control of SUMO proteins. **p < 0.01 versus the mock-infected cells in (A), versus the wild-type pNPM1 transfected cells in (B). ^##^ *p* < 0.01 versus the PCV2-infected cells at 18 h post-infection in (A). ND represents not detected.

To further refine the expression and change signature of the both pNPM1 and SUMOylated pNPM1 during PCV2 infection, we examined the expression levels of pNPM1 and found that within 24 h of PCV2 infection, the pNPM1 (~38 kDa) expression levels did not show visible changes in PCV2-infected cells compared with that in the mock-infected cells, but the SUMOylated pNPM1 were significantly upregulated in the PCV2-infected cells than that in the mock-infected cells at 6 h, 12 h, and 18 h post-infection, and then reduced to the same level of the mock-infected cells at 24 h post-infection (Fig 4A). To further identify the subsequent change pattern of the SUMOylated pNPM1 and Cap, we continually monitored the SUMOylated pNPM1 levels and Cap expression levels at 36 h and 48 h post-infection. The results showed that the levels of SUMOylated pNPM1 and Cap at 36 h and 48 h post-infection were significantly increased compared to them at 24 h post-infection (S2A Fig). Consistent with these changes, the PCV2 DNA copy numbers were dramatically increased at 36 h and 48 h post-infection (S2B Fig). These results suggest that the SUMOylation of pNPM1 is associated with PCV2 Cap expression changes and the PCV2 DNA replication.

Next, we monitored the effect of the K230, K263, or K230/K263 mutation on the SUMOylation of pNPM1. In the PK-15^*npm1-/-*^ cells expressed with the K263 SUMOylation sites-mutated pNPM1, SUMOylated pNPM1 were not upregulated followed by PCV2 infection, and exhibited a significantly reduced level compared with that in the PK-15^*npm1-/-*^ cells expressed with wild-type pNPM1 (Fig 4B). As there are three major SUMO proteins, including SUMO1, SUMO2, and SUMO3, which participate in the SUMOylation process in eukaryotes, we want to know which SUMO is conjugated to pNPM1 for modification during PCV2 infection. We co-expressed the GFP-fused porcine SUMO1, SUMO2, or SUMO3 proteins with Flag-tagged wild-type pNPM1 in PK-15^*npm1-/-*^ cells, respectively, and then the cells were infected with PCV2 for 12 h. The co-localization assay showed that whether the cells were infected with PCV2 or not, the three SUMO proteins were broadly distributed in the cells (Fig 4C). Nevertheless, although pNPM1 was also widely distributed in the mock-infected cells, they were predominantly located in the nucleolus of the PCV2-infected cells (Fig 4C). All three SUMO proteins were found co-localized with pNPM1 in both these mock- and PCV2-infected cells (Fig 4C). To further explore which SUMO proteins conjugated with pNPM1 were significantly changed in PCV2-infected cells, co-IP assays were done using the above cells. The results further confirmed that pNPM1 could interact with all three SUMO proteins, no matter whether the cells were infected with PCV2 or not (Figs 4D, 4E, 4F and S3). However, PCV2 infection significantly promoted the interaction of pNPM1 with SUMO2 and SUMO3, but not with SUMO1 in the cells compared with mock infection (Figs 4D, 4E, 4F and S3), suggesting that PCV2 infection promotes SUMO2/3 conjugation with pNPM1 in the nucleoli of infected cells, but has no obvious effect on SUMO1 and pNPM1 binding.

### PCV2 infection upregulates Ubc9 expression to promote the interaction of TRIM24 with pNPM1 to enhance pNPM1 SUMOylation

**Fig 5:**
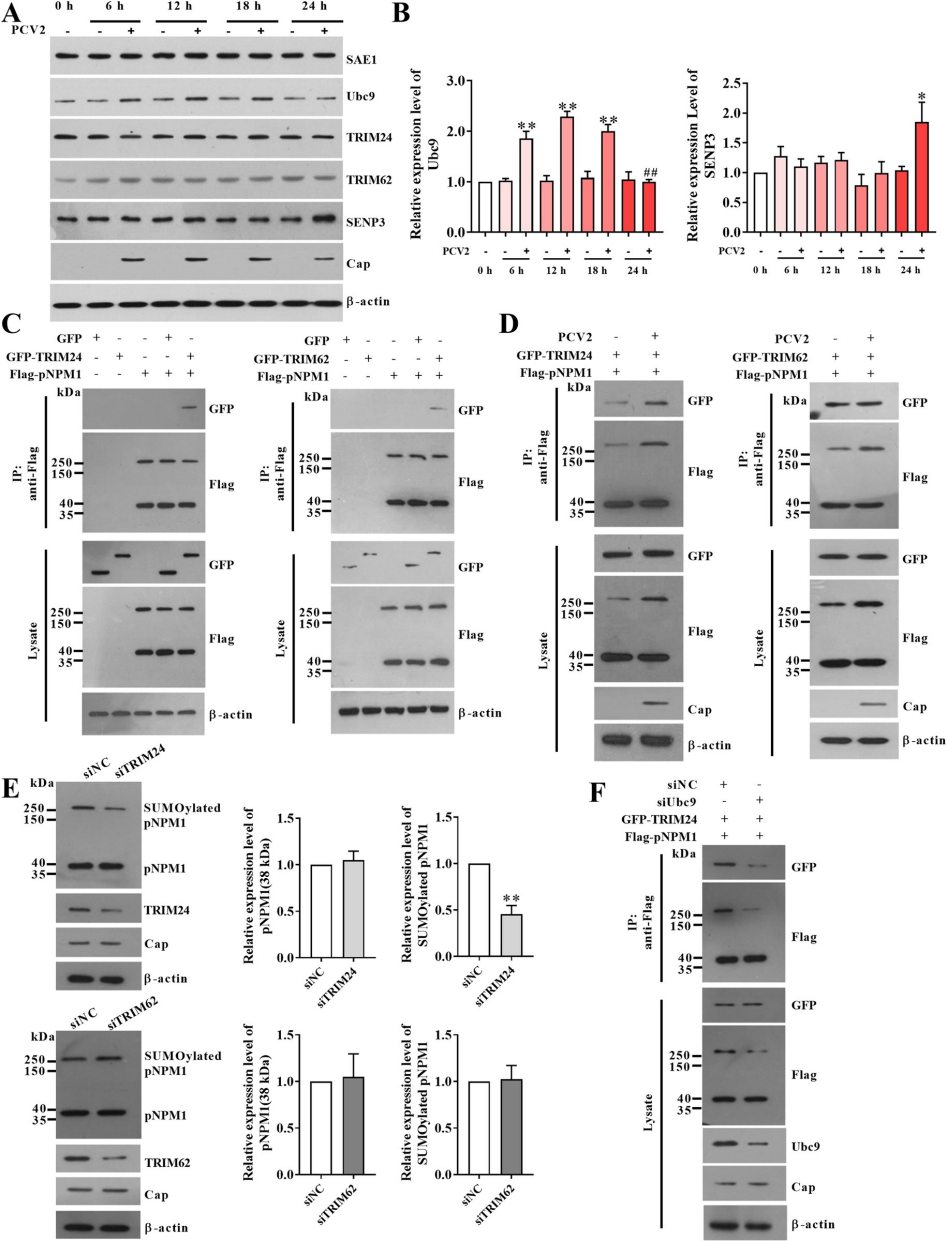
PCV2 infection promotes Ubc9/TRIM24 signalings to enhance the SUMOylation of pNPM1. (**A, B**) 1 MOI PCV2 or mock (the same volume of medium) infected PK-15 cells for 0 h, 6 h, 12 h, 18 h, and 24 h. (**A**) The expression levels of SAE1, Ubc9, TRIM24, TRIM62, and SENP3 were analyzed by western blot. (**B**) The expression levels of Ubc9 and SENP3 relative to β-actin were calculated. (**C**) The plasmids expressing TRIM24, TRIM62, and pNPM1 were transfected into 293T cells for 48 h, and the interactions of TRIM24 and TRIM62 with pNPM1 were detected by co-IP assays. (**D**) The plasmids expressing TRIM24, TRIM62, and pNPM1 were transfected into PK-15^*npm1-/-*^ cells for 24 h, and then the cells were infected with 1 MOI PCV2 or mock for 12 h. The interactions of TRIM24 and TRIM62 with pNPM1 were detected by co-IP assays. (**E**) The specific siRNAs targeting TRIM24 or TRIM62 were transfected into PK-15 cells for 24 h, then the cells were infected with 1 MOI PCV2 for 12 h. The expression levels of TRIM24, TRIM62, pNPM1, and SUMOylated pNPM1 were detected by western blot and calculated. (**F**) The specific siRNA targeting Ubc9 was transfected into PK-15 cells for 24 h, then the cells were infected with 1 MOI PCV2 for 12 h. The interactions of TRIM24 with pNPM1 were detected by co-IP assays. **p < 0.01, *p < 0.05 versus the mock-infected cells at the same time point in (B), **p < 0.01 versus the siNC-transfected cells in (E), ^##^*p* < 0.01 versus the PCV2-infected cells at 18 h in (B).

SUMOylation is a multistep process to activate and attach SUMO proteins to substrates [8]. To make clear how PCV2 infection promotes the conjugation of SUMO2/3 with pNPM1 in cells, we first detected the SAE1 (SUMO1 Activating Enzyme Subunit 1, E1 enzyme of SUMOylation) and Ubc9 (Ubiquitin-conjugating enzyme 9, E2 enzyme of SUMOylation) expression levels during PCV2 infection. The results showed that PCV2 infection did not change SAE1 expression levels, but significantly upregulated the expression levels of Ubc9 at 6 hpi, peaked at 12 hpi, and finally subsided to the same levels as mock-infected cells at 24 hpi (Fig 5A and 5B). On the other hand, the nucleolar SUMO-specific protease single nucleotide polymorphisms 3 (SENP3) is reported to catalyze the deSUMOylation of NPM1 [26]. Thus, we checked the SENP3 expression levels in the PK-15 cells during PCV2 infection. The results showed that the SENP3 expression levels were not visibly changed from 6 h to 18 h post-PCV2 infection, but significantly upregulated at 24 h post-PCV2 infection, compared with the mock infection (Fig 5A and 5B). It has been reported that the tripartite motif-containing protein 28 (TRIM28) acts as a E3 ligase for the alternative reading frame protein (ARF)-mediated SUMOylation of NPM1 [27,28]. However, our results showed that PCV2 infection did not significantly alter the expression levels of ARF and TRIM28 in PK-15 cells compared with the mock infection (S4A Fig). We further found that PCV2 infection did not influence the interaction of TRIM28 with pNPM1 compared with the mock infection (S4B Fig), and the inhibition of the TRIM28 expression did not alter the PCV2 infection-induced pNPM1 SUMOylation level (S4C Fig). Thus, we sought the TRIMs family members in the MS results, and TRIM24 and TRIM62 were found and the interaction of them with pNPM1 was confirmed (Figs 5C and S4D). Although PCV2 infection also did not affect the expression levels of TRIM24 and TRIM62 (Fig 5A), PCV2 infection promoted the interaction of TRIM24 with pNPM1, but not TRIM62 with pNPM1 (Figs 5D and S4E). Further, our results showed that down-regulation of the TRIM24 expression significantly reduced the SUMOylation level of pNPM1, while down-regulation of TRIM62 expression did not (Fig 5E), and down-regulation of the Ubc9 expression suppressed the PCV2 infection-induced the interaction of TRIM24 with pNPM1 (Figs 5F and S4F). These results show that PCV2 infection upregulates Ubc9 expression to promote the interaction of TRIM24 with pNPM1, leading to the enhancement of pNPM1 SUMOylation at the early stage of infection, while increasing the expression level of SENP3 at a later stage that may mediate the reduction of the pNPM1 SUMOylation.

### Cap is the major component of PCV2 that enhances pNPM1 SUMOylation during infection via activation of the ERK signaling pathway

**Fig 6:**
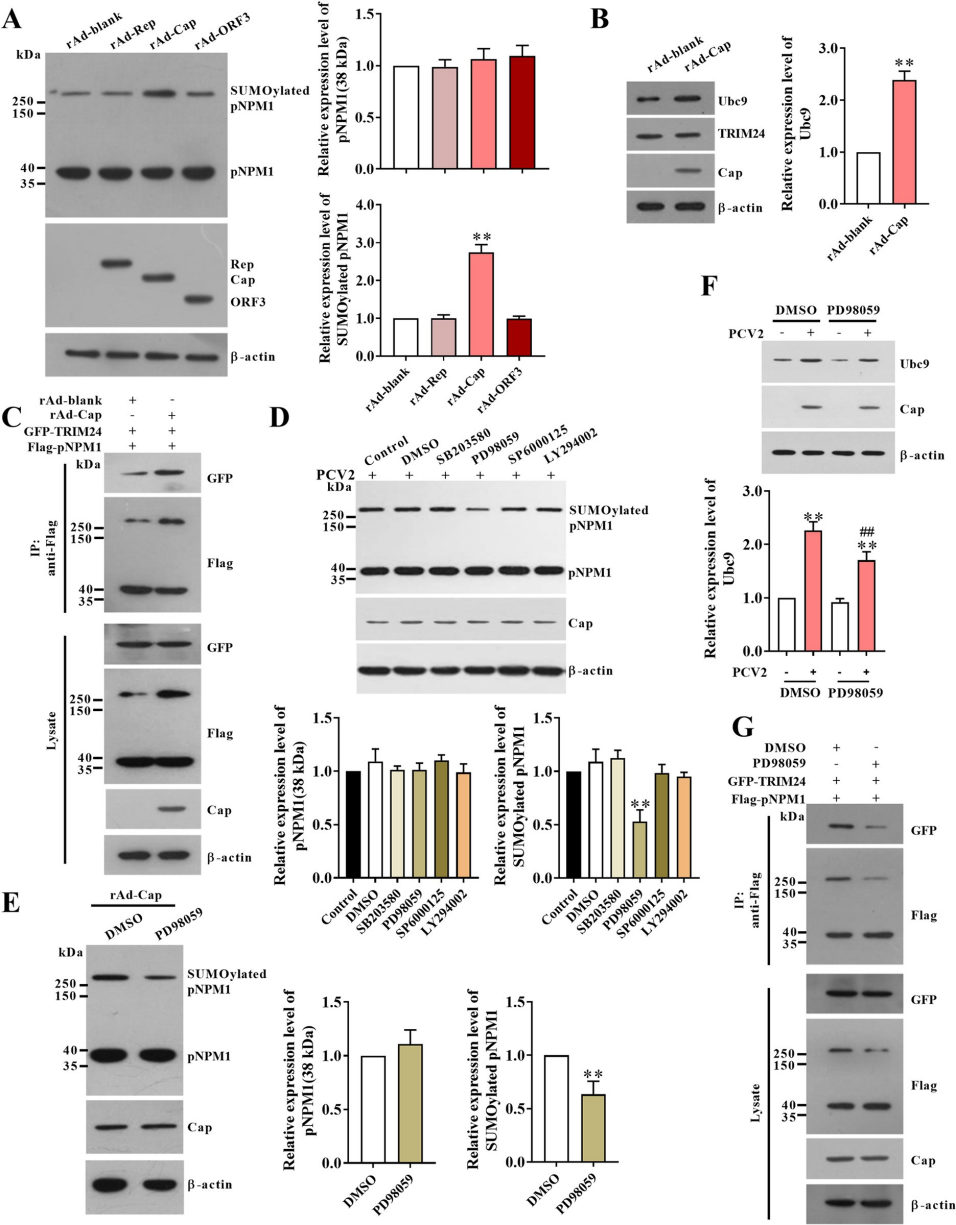
PCV2 Cap is the major component promoting pNPM1 SUMOylation with the activation of the ERK pathway. (**A**) PK-15 cells were infected with 100 MOI of the blank recombinant adenovirus (rAd-blank), the recombinant adenovirus expressing PCV2 Rep (rAd-Rep), the recombinant adenovirus expressing PCV2 Cap (rAd-Cap), or the recombinant adenovirus expressing PCV2 ORF3 (rAd-ORF3) for 24 h. The expressions of pNPM1, Rep, Cap, and ORF3 were analyzed by western blot. The expression levels of pNPM1 were calculated. (**B**) PK-15 cells were infected with 100 MOI rAd-blank or rAd-Cap for 24 h. The expression levels of Ubc9 and TRIM24 were analyzed by western blot and calculated. (**C**) PK-15 cells were infected with 100 MOI rAd-blank or rAd-Cap for 24 h. The interactions of TRIM24 with pNPM1 were detected by co-IP assays. (**D**) PK-15 cells were pre-treated with Control, DMSO, or specific inhibitors for PI3K/Akt (LY294002, 10 μM), ERK (PD98059, 20 μM), p38 (SB203580, 10 μM), and JNK (SP6000125, 10 μM) for 2 h, respectively. Then the cells were infected with 1 MOI PCV2 for 12 h with the presence of the inhibitors. The expressions of pNPM1 were analyzed by western blot, and the expression levels of pNPM1 and SUMOylated pNPM1 relative to β-actin were calculated. (**E**) PK-15 cells were pre-treated with DMSO or PD98059 for 2 h, and then infected with 100 MOI rAd-Cap for 24 h with the presence of PD98059. The expressions of pNPM1 were analyzed by western blot, and the expression levels of pNPM1 and SUMOylated pNPM1 relative to β-actin were calculated. (**F, G**) PK-15 cells were pretreated with DMSO or PD98059 for 2 h. The cells were infected with 1 MOI PCV2 for 12 h with the presence of PD98059. (**F**) The expression levels of Ubc9 were analyzed by western blot and calculated. (**G**) The interaction of TRIM24 with pNPM1 were detected by co-IP assays. **p < 0.01 versus the rAd-blank-infected cells in (A) and (B), **p < 0.01 versus the DMSO-treated cells in (D), (E), and (F).

PCV2 expresses three major proteins, the replicase Rep, the capsid protein Cap, and the non-structure protein ORF3 during infection, to figure out which component plays a predominant role in the enhancement of pNPM1 SUMOylation, PK-15 cells were infected with the same doses of recombinant adenovirus expressing PCV2 Rep (rAd-Rep), PCV2 Cap (rAd-Cap), and PCV2 ORF3 (rAd-ORF3), as well as blank recombinant adenovirus (rAd-blank), respectively. The results showed that compared with the rAd-blank infection, the rAd-Rep, rAd-Cap, or rAd-ORF3 infections all did not significantly affect the expression of 38 kDa pNPM1, while the rAd-Cap infection, but not the rAd-Rep or rAd-ORF3 infections, significantly upregulated the SUMOylated pNPM1 expression (the bands above 250 kDa) (Fig 6A). In the rAd-Cap-infected cells, the Ubc9 expression levels were also upregulated when compared with that in the rAd-blank-infected cells, while the TRIM24 expression levels were not changed (Fig 6B). Similarly, the rAd-Cap infection promoted the interaction of TRIM24 with pNPM1 compared with the rAd-blank infection (Figs 6C and S5A). The above results indicate that PCV2 Cap is the major component in enhancing pNPM1 SUMOylation in PK-15 cells.

PCV2 Cap activates PI3K/Akt, ERK, p38, and JNK signaling pathways during PCV2 infection, and these signaling pathways are known to participate in multiple cellular processes [18], we wondered whether these signaling pathways were involved in the regulation of pNPM1 SUMOylation in PCV2-infected cells. We used the specific inhibitors of PI3K/Akt (LY294002), ERK (PD98059), p38 (SB203580), and JNK (SP6000125) signaling pathways to pretreat PK-15 cells, and then infected the cells with PCV2 for 12 h with the present of the inhibitors. The results showed that inhibitions of PI3K/Akt, ERK, p38, or JNK signaling pathways did not affect the 38 kDa pNPM1 expression levels, yet inhibition of ERK signaling pathway significantly reduced the levels of SUMOylated pNPM1, whereas inhibitions of PI3K/Akt, p38, or JNK signaling pathways could not (Figs 6D and S5B). Inhibition of the ERK signaling pathway also reduced the rAd-Cap infection-induced SUMOylation of pNPM1 (Fig 6E). Furthermore, we found that the inhibition of the ERK signaling pathway reduced the PCV2 infection-induced upregulation of Ubc9 expression levels as well as the interaction of TRIM24 with pNPM1 (Figs 6F, 6G and S5C). These results demonstrate that PCV2 infection activates the ERK signaling pathway through Cap to enhance the SUMOylation of pNPM1 in PK-15 cells.

### Inhibition of PCV2 Cap-induced ERK signaling activation suppresses pNPM1 nucleolar localization and PCV2 DNA replication

**Fig 7:**
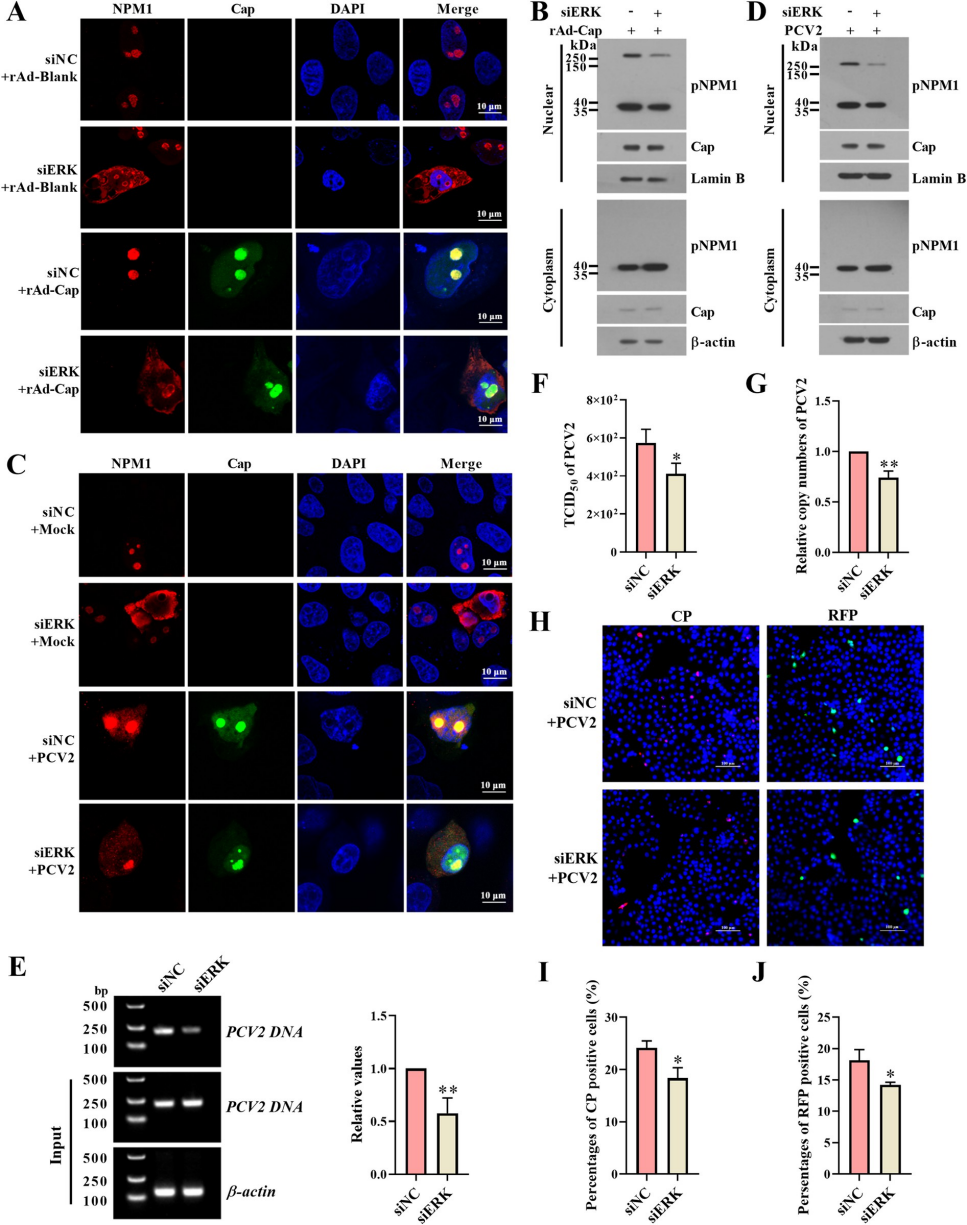
The ERK-specific siRNA treatment suppresses the nucleolar localization of pNPM1 and the replication of PCV2 DNA. (**A**) PK-15 cells were transfected with the siRNA specific for ERK (siERK) or negative control siRNA (siNC) for 24 h and then infected with 100 MOI rAd-Cap or rAd-blank for another 24 h. The localizations of pNPM1 in these cells were measured by confocal microscopy. The bars = 10 μm were indicated in each panel. (**B**) The cells were harvested and lysed with a nuclear and cytoplasmic protein extraction kit, then the expression of the pNPM1 and Cap were measured by western blot. (**C-J**) PK-15 cells were transfected with the specific siERK or siNC for 24 h and then infected with 1 MOI PCV2 for 12 h. (**C**) The localizations of pNPM1 in these cells were measured by confocal microscopy. The bars = 10 μm were indicated in each panel. (**D**) The cells were harvested and lysed with a nuclear and cytoplasmic protein extraction kit, then the expression of the pNPM1 and Cap were measured by western blot. (**E**) The interactions of the pNPM1 with PCV2 DNA were measured by ChIP assays. (**F**) The TCID_50_ of PCV2 in these cells were measured and calculated. (**G**) The copy numbers of PCV2 DNA were detected by qPCR and calculated. (**H, I, J**) The PCV2 genomes (CP) or replication form DNA (RFP) of the PCV2-infected siERK-transfected cells were detected using fluorescently-labeled specific DNA probes. (**H**) The positive cells were photographed by a fluorescence microscope. The bars = 100 μm were indicated in each panel. (**I**) The percentages of CP-positive cells were quantified per 500 cells. (**J**) The percentages of RFP-positive cells were quantified per 500 cells. *p < 0.05, **p < 0.01 versus the siNC transfected cells in (E), (F), (G), (I) and (J).

To further confirm the role of PCV2 Cap-induced ERK signaling activation in pNPM1 localization, we first transfected PK-15 cells with the specific siRNA of ERK (siERK) or the negative control siRNA (siNC) for 24 h and then infected the cells with 100 MOI rAd-Cap. The results showed that inhibition of ERK expression by specific siRNA reduced the Cap-induced nucleolus localization of pNPM1, while the nucleolus localization of Cap was not visibly changed (Figs 7A, 7B, S6A and S6B). We also infected the siERK-transfected PK-15 cells with 1 MOI PCV2 for 12 h, and the results confirmed that the nucleolar localization of pNPM1 induced by PCV2 infection was reduced in the siERK-transfected cells than that in the siNC-transfected cells (Figs 7C, 7D, S6B and S6C). To further figure out the role of the ERK signaling in PCV2 DNA replication, we found that the siERK transfection significantly inhibited the interaction of pNPM1 with PCV2 DNA by ChIP assays (Fig 7E), correspondingly, the TCID_50_ and viral copy numbers of PCV2 were significantly decreased in the siERK-transfected cells than that in the siNC-transfected cells (Fig 7F and 7G). Furthermore, the FISH results showed that both the PCV2 genome (CP) and the replication form (RFP) were less detected in the siERK-transfected cells than in the siNC-transfected cells (Fig 7H, 7I and 7J). These results further demonstrate that the PCV2 Cap-induced ERK signaling activation is important for the nucleolar localization of pNPM1, in which place the SUMOylated pNPM1 participates in the regulation of PCV2 DNA replication.

## Discussion

Viral DNA replication is a complicated process relying on the cellular DNA replication system. The circular single-stranded DNA virus PCV2 is one of the most spread swine pathogens that leads to a huge economic loss to the swine industry in the world. The PCV2 infection-induced clinical syndromes may relate to the viral loads in pigs, that PCV2 vaccination can prevent the occurrence of PCV2 infection-induced clinical syndromes, but PCV2 still exists in pigs at low levels [29,30], while clinically diseased pigs tend to infect with high levels of PCV2 [18]. SUMOylation is an important post-transcriptional modification that has been found to play critical roles during cellular DNA replication. In this study, we found that the previously reported PCV2 Cap interacting protein pNPM1 could also interact with PCV2 DNA in a SUMOylated form to regulate the PCV2 DNA replication. PCV2 infection did not change the expression level of pNPM1 within 24 h but promoted the SUMOylation of pNPM1. PCV2 Cap was the major component that activated ERK/Ubc9/TRIM24 signalings to promote SUMO2/3 conjugation to pNPM1. The SUMOylated pNPM1 localized in the nucleoli of the cells and interacted with PCV2 Cap to participate in the regulation of PCV2 DNA replication.

NPM1, or called B23, is a multifunctional protein participating in the different life cycles of cells, including DNA replication and repair, histone- and protein-chaperone activity, ribosome assembly and export, centrosome duplication, cell cycle control, response to stress stimuli, embryogenesis [20]. Several studies show that NPM1 is involved in the replicate regulation of some viruses through different mechanisms. For example, NPM1 interacts with the core dimers of the Hepatitis B virus to promote capsid assembly [31], and NPM1 is helpful for the formation of DNA replication compartments and viral DNA accumulation sites of adenovirus [32]. It has been previously found that pNPM1 is an interacting protein of PCV2 Cap [33], and pNPM1 binds with Cap in the nucleus of cells and participates in the PCV2 replication [25]. In this study, we further found that pNPM1 is also an interacting protein of PCV2 DNA. As it has been reported, NPM1 is a nucleocytoplasmic shuttling protein, that can interact with proteins and nucleic acids through different domains [20,34,35]. The previous study found that the N-terminal domain of pNPM1 is responsible for the interaction of pNPM1 and PCV2 Cap [25], while our present results showed that the C-terminal domain of pNPM1 is critical for the interaction of pNPM1 and PCV2 DNA, this is consistent with the structure of pNPM1 that the N-terminal interacts with proteins and the C-terminal interacts with DNA/RNA [20]. However, whether the interaction between pNPM1 and PCV2 DNA depends on the interaction between pNPM1 and Cap remains to be further investigated.

The subcellular localization and biological function of NPM1 are regulated by post-translational modifications, including phosphorylation, acetylation, ubiquitylation, glutathionylation, and SUMOylation [24,36]. It has also been reported that human immunodeficiency virus type 1 (HIV-1) infection induces acetylation of NPM1, which is critical for the nuclear localization of Tat as well as Tat-mediated viral gene transcription [37]. In this study, we identified pNPM1 within the SUMOylated proteins that interacted with PCV2 DNA and found that when the SUMOylation of pNPM1 was been suppressed, the interaction of pNPM1 and PCV2 DNA was reduced. Interestingly, our study also found that PCV2 infection did not alter the expression level of pNPM1 as the previous [25]. Notably, our study found that PCV2 infection promotes the SUMOylation of pNPM1 in PK-15 cells. However, a phenomenon that is well worthy of our attention during PCV2 infection, since the host protein MKRN1 (a Cap interacting protein) mediates the degradation of Cap that limits the Cap protein levels at the early phase of infection, our results also showed that the Cap expression levels were not increased during PCV2 infection within 24 h in this study, which is similar as the results presented in our previous study [16]. However, the Cap levels then dramatically increased to higher levels at 36 h and 48 h post-infection. Consistently, the pNPM1 SUMOylation levels were increased at 6 h to 18 h post-infection, slightly decreased at 24 h post-infection, and subsequently, markedly increased again at 36 h and 48 h post-infection. Meanwhile, the data present in this study demonstrate that Cap is a major viral component in promoting the SUMOylation of pNPM1. On the other hand, it should be noted that our previous study also indicates that PCV2 downregulates the expression of MKRN1 to avoid the MKRN1-mediated Cap ubiquitination and degradation at the late phase of infection, thereby promoting viral replication [16]. When we infected the cells with a higher titer of PCV2, the viral-induced MKRN1 reduction and the associated Cap ubiquitination and degradation would occur at a much earlier time point, but not the 24 hpi as in this manuscript [15]. Additionally, we found that MKRN1 mediates PCV2 Cap ubiquitination and degradation by targeting the lysine residues 164, 179, and 191, yet the lysine residues 164 and 179 are highly conserved, while the lysine residue 191 is quite diverse in different strains, which may lead to different degrees of Cap ubiquitination and degradation [16,38]. It can be thus expected that the pNPM1 SUMOylation induced by Cap would have different dynamics when cells were infected with different PCV2 strains or different titers of PCV2.

Numerous studies have investigated the PTMs of human NPM1, including 8 phosphorylation sites (S4, S10, S70, S125, T199, T219, T234, and S254), 5 acetylation sites (K211, K214, K229, K257, and K267), 2 SUMOylation sites (K230 and K263), and 1 glutathionylation site (C275) [28,39–41]. We found most of these PTM sites were conserved on pNPM1 by alignment of the amino acid sequences of human NPM1 and pNPM1. Despite that, we still wondered if there are other SUMOylation sites on pNPM1, so we analyzed the potential SUMO-conjugation consensus motifs on pNPM1 using four bioinformatics analysis (SUMOplot, JASSA, GPS-SUMO, and R. Hay). A total of 23 potential SUMOylation sites were found, but only K24, K32, K54, K190, K230, and K263 sites were predicted by more than three bioinformatics analyses. Among these potential SUMOylation sites, K24, K32, and K54 sites have been proven that could not be SUMOylated on human NPM1, while K230 and K263 sites can be SUMOylated, the K263 site is the major SUMOylation site of human NPM1, and the K230 site also contributes to SUMOylation when human NPM1 is multi-SUMOylated [23]. For the K190 site, which has the lowest scores of SUMOplot and JASSA, and can only be predicted by the Relaxed Reverted Criteria model of R. Hay analysis, we did not tend to consider it as the SUMOylation site of pNPM1. Therefore, we mutated the K230 site, the K263 site, or both sites of pNPM1, and the results found that the mutation of the K230 site only slightly, but not significantly, affected the SUMOylation level of porcine NPM1, the mutation of the K263 site dramatically reduced the porcine NPM1 SUMOylation, and the mutation of both K230 and K263 sites completely abolished the SUMOylation of porcine NPM1. We speculate that the K263 site is also the major SUMOylation site of porcine NPM1, and the K230 site of porcine NPM1 could be partially SUMOylated when the SUMOylation of the K263 site is blocked, while the porcine NPM1 SUMOylation would be completely blocked when both sites are been mutated at the same time. Of course, the SUMOylation of pNPM1 resulting from PCV2 infection is best determined by mass spectrometry, but we found that the SUMOylation of pNPM1 could also be detected in the mock-infected cells, it would be difficult to confirm that the SUMOylation of pNPM1 is indeed caused by PCV2 infection using mass spectrometry. The SUMOylation of NPM1 also affects its subcellular localization, and it has been found that mutation of the NPM1 K263 site, but not the K230 site, leads to the abolishing of its nucleolar localization [23]. In this study, we the same found that the K263R mutant pNPM1 did not correctly locate in the nucleoli of PK-15 cells, in which place PCV2 Cap is mainly located. Since the PCV2 DNA replicates in the nucleoli of cells, it is reasonable to consider that the SUMOylated pNPM1 participates in regulating PCV2 DNA replication, and our results confirmed that. However, the detailed mechanism of SUMOylated pNPM1 regulates PCV2 DNA replication still needs further exploration.

It has been reported that the hNPM1 contains two nuclear export signals (NES) at 42–49 aa and 94–102 aa on the N-terminal domain, a bipartite nuclear localization signal (NLS) at 152–157 aa and 191–197 aa on the IDR domain, and an atypical nucleolar localization signal (NoLS) at W288 and W290 on the C-terminal domain [20]. Although there is no such study on pNPM1, the amino acid sequence analysis result showed that all the NES, NLS, and NoLS sequences are conservative on pNPM1 with that on hNPM1. The previous studies found that the wild-type hNPM1 predominantly located in the nucleoli of cells, while the mutation and deletion of NES(94–102) made the hNPM1 largely distributed in the nucleoplasm, the mutation of NLS(152–157) abolished the localization of hNPM1 in the nucleoplasm but mainly found in the cytoplasm and nucleoli, the NoLS(W288 and W290) mutation also made hNPM1 mainly located in the nucleus of cells [42]. Recently, there is a study showed that the truncated pNPM1 that deficiency the NTD domain, the IDR domain, and the CTD domain all lost the ability to localize the nucleoli, the pNPM1-ΔNTD and the pNPM1-ΔCTD mainly localized in the nucleus but not aggregated in the nucleoli, while the pNPM1-ΔIDR was mainly distributed in the cytoplasm of ST cells [43]. It has also been reported that the K263 SUMOylation is essential for hNPM1 nucleolar residency, yet the K230 site mutation does not affect the hNPM1 nucleolar location [23]. However, there is no direct evidence to show that the SUMOylation of the K236 site will affect the NLS or the NoLS of NPM1. Thus, how the SUMOylation affects the subcellular location of pNPM1 needs further study.

Among the eukaryotic cells, there are at least three SUMO proteins (SUMO1, SUMO2, and SUMO3). SUMO2 and SUMO3 are highly homologous so often referred to as SUMO2/3, but they share only about 50% sequence identity with SUMO1 [8]. We found that PCV2 infection majorly promotes SUMO2/3, but not SUMO1, binding to pNPM1. Normally, the SUMO proteins conjugate with proteins in the form of chains similar to ubiquitin, so the target proteins would exhibit a ladder of different molecular weights. However, in this study, we found that the SUMOylated pNPM1 only presents a single band, which is consistent with the previously reported results [23]. In addition, SUMOylation is a multistep process that involves the E1 enzyme (SAE1/SAE2), E2 enzyme (Ubc9), and E3 ligases that promote the transfer of activated SUMO to substrates. We found that PCV2 infection did not alter the expression level of SAE1, but significantly upregulated the expression level of Ubc9 in PK-15 cells. Although there is a report showed that TRIM28 is an E3 ligase for ARF-mediated SUMOylation of NPM1, the authors also claimed that ARF/TRIM28 did not affect the SUMOylation of NPM1 on K230 and K263 sites [28]. Our results did find that PCV2 infection did not visibly influence the expression levels of ARF and TRIM28 as well as the interaction of TRIM28 and pNPM1 in PK-15 cells, thus the PCV2 infection-induced SUMOylation may not be through the ARF/TRIM28 signalings. Despite Ubc9 being able to conjugate SUMO to some proteins independent of E3 ligase, we still checked if the TRIMs family members, TRIM24 and TRIM62, found in the MS results would be the E3 ligases for pNPM1 SUMOylation. Our results showed that although both TRIM24 and TRIM62 interacted with pNPM1, only the interaction of TRIM24 with pNPM1 was enhanced during PCV2 infection to promote pNPM1 SUMOylation and the enhancement was Ubc9 dependent. Regarding how PCV2 infection regulates Ubc9 expression, we found that inhibition of the ERK signaling activated by PCV2 infection in PK-15 cells can significantly reduce the expression level of Ubc9 as well as the SUMOylated pNPM1. ERK plays multiple functions. It has been reported that ERK can dynamically regulate gene expression through its targeting transcriptional factors, including c-FOS, Elk1, Ets1, and SP-1 [44]. We predicted the transcriptional factors binding sites on the promoter of the porcine Ubc9 gene using JASPAR and found it contains the potential motifs that bind with c-FOS, Elk1, Ets1, and SP-1. Thus, we consider that PCV2 can activate ERK signaling to regulate Ubc9 expression. Yet, our results also showed that ERK signaling may not be the only signaling that regulates Ubc9 expression in PK-15 cells, since inhibition of ERK signaling could not prevent the PCV2 infection-induced Ubc9 expression. In addition, we confirmed that Cap is the major component of PCV2 that promotes pNPM1 SUMOylation in PK-15 cells, and Cap mediated the activation of ERK/Ubc9/TRIM24 signalings. That would be interesting to further make clear the relationship between the activation of signalings by Cap and the interaction of pNPM1 and Cap. Finally, it has been reported that the SUMOylation of NPM1 can be negatively regulated by SENP3 [26], we checked the expression levels of SENP3 in PCV2-infected PK-15 cells, the results showed that PCV2 infection upregulated the expression of SENP3 at 24 h post-infection, indicating that the decrease of the SUMOylation level of pNPM1 may be also relative to the deSUMOylation activity of SENP3. Nevertheless, the underlying mechanism and role of pNPM1 deSUMOylation at the later phase of PCV2 infection still needs further investigation.

In conclusion, we found that PCV2 infection promotes the SUMOylation of pNPM1 through the Cap-mediated activation of ERK/Ubc9/TRIM24 signalings. The SUMOylated pNPM1 is located in the nucleoli of cells to participate in the regulation of PCV2 DNA replication.

## Materials and methods

### Viruses, reagents, and antibodies

PCV2 (GenBank: MH492006) was stocked in our laboratory and propagated in PK-15 cells. The 50% tissue culture infective dose (TCID_50_) of PCV2 was measured as follows: the viral preparation was serially diluted and then incubated to PK-15 cells, post 48 h incubation, the PCV2-infected cells were detected by indirect immunofluorescence assay with a PCV2 Cap antibody, the TCID_50_ was calculated according to the Reed–Muench method [45]. The recombinant adenovirus expressing PCV2 Rep, Cap, ORF3, and blank were constructed and stored in our lab [46].

The PI3K/Akt inhibitor (LY294002), ERK1/2 MAPK inhibitor (PD98059), and p38 MAPK inhibitor (SB203580) were purchased from Merck, the JNK inhibitor (SP6000125) and the SUMOylation inhibitor 2-D08 (SML1052) were purchased from Sigma, and the SUMOylation inhibitor ML-792 (1644342-14-2) was purchased from MCE. The effects of 2-D08 and ML-792 on cell viability were measured as shown in S7 Fig, and the effects of other reagents on cell viability were measured in our previous study [18]. The mouse anti-NPM1 monoclonal antibody (sc-271737) was purchased from Santa Cruz. The rabbit anti-NPM1 Polyclonal antibody (10306-1-AP), the mouse anti-Flag-Tag monoclonal antibody (66008-4-Ig), and the rabbit anti-GFP-Tag Polyclonal antibody (50430-2-AP) were purchased from Proteintech, China. The rabbit anti-Flag-Tag monoclonal antibody (14793) was purchased from Cell Signaling Technology. The rabbit anti-Pan SUMO polyclonal antibody (abs101685) was purchased from absin, China. The rabbit anti-Ubc9 monoclonal antibody (CY5571), the rabbit anti-SAE1 monoclonal antibody (CY8264), the rabbit anti-ARF monoclonal antibody (CY8312), and the mouse anti-GFP-Tag monoclonal antibody (AB0005) were purchased from Abways, China. The rabbit anti-SENP3 polyclonal antibody (DF8198) was purchased from Affinity Bioscience, China. The rabbit sera anti-PCV2 Rep, anti-PCV2 Cap, and anti-PCV2 ORF3 were previously obtained in our lab [47]. The mouse anti-β-actin monoclonal antibody (A00702) was purchased from Genscript, China. The rabbit anti-Lamin B1 polyclonal antibody (R1508-1) was purchased from HUABIO China. The HRP-conjugated goat anti-rabbit IgG (H+L) (31460) and HRP-conjugated goat anti-mouse IgG (H+L) (31430) were purchased from Thermo. The Alexa Fluor 594 affiniPure goat anti-mouse IgG (H+L) (33212ES60) was purchased from Yesen Biotechnology, China.

### DNA pull-down and immunoprecipitation

The Biotin-labeled primers (Biotin-GAACCGCGGGCTGGCTGAACTCTTGAAAGT-Biotin and Biotin-GCACCGCGGAAATTTCTGACAAACGTTACA-Biotin) were used for PCR amplification of the full length of PCV2 DNA. The PCR products were cyclized and transfected into PK-15 cells. Then the cells were infected with 1 MOI PCV2 for 12 h. The infected cells were lysed on ice and the PCV2 DNA interacting proteins were pulled down using streptavidin-conjugated beads. The pulled proteins were then further incubated with the rabbit anti-Pan SUMO polyclonal antibodies overnight at 4°C, and the SUMOylated proteins were precipitated using protein A agarose beads and analyzed by Liquid Chromatography-Mass Spectrometer/Mass Spectrometry (LC-MS/MS).

### Chromatin immunoprecipitation (ChIP)

PK-15 cells were infected with 1 MOI PCV2 or mock for 12 h. The cells were cross-linked by formaldehyde and lysed by nuclei lysis buffer for the chromatin. The lytic chromatin was sonicated, then quantified and divided into 100 μg DNA portions. The DNA portions were precleared by protein A(G)-agarose/salmon sperm DNA beads and then incubated with the NPM1 monoclonal antibodies and irrelevant antibodies at 4°C overnight. Protein A(G)-agarose/salmon sperm DNA beads were added to the samples again to bind to the antibody-conjugated compounds. Then the compounds were digested by proteinase K, and extracted by Phenol: Chloroform to purify nucleic acids. The nucleic acids were resuspended in H_2_O and analyzed by PCR. The primers for the PCV2 sequence containing the replication origin region were 5’-CGGTGTCTTCTTCTCCGGTA-3’ and 5’-AGCGTGAACACCCACCTTTTA-3’. The primers for β-actin were 5’-GGACTTCGAGCAGGAGATGG-3’ and 5’-AGGAAGGAGGGCTGGAAGAG-3’.

### Western blot

Equal amounts of protein were electrophoresed by SDS-PAGE and transferred to polyvinyl difluoride (PVDF) membranes (Millipore). After blocking for 1 h with blocking buffer (5% nonfat milk and 0.1% Tween-20 in PBS), the membranes were incubated with the primary antibodies at 4 ^**o**^C overnight and then incubated with HRP-conjugated second antibodies. The conjugated PVDF membranes were incubated with ECL and exposed in dark.

### Generation of pNPM1 knockout cells

The pNPM1 knockout PK-15 cells were generated by CRISPR/Cas9 system. Two gRNAs targeting pNPM1 were designed as the sequences were 5’-AGGCCTCAGAACTATCTTTTCGG-3’ and 5’-AGTATATCTGGAAAGCGTTCTGC-3’. The gRNAs were cloned into the lentiCRISPRv2 plasmid (Addgene #52961). The recombinant plasmids were co-transfected with psPAX2 and pMD2G into 293T cells to obtain recombinant lentiviruses. PK-15 cells were infected with the recombinant lentiviruses and had been screened using puromycin at 24 h post-infection. The monoclonal pNPM1 knockout PK-15 (PK-15^*npm1*-/-^) cells were verified by western blot and sequencing.

### Confocal microscopy assay

The PK-15^*npm1*-/-^ cells were seeded on round coverslips in 24-well plates overnight. The plasmids expressing label fusion proteins were co-transfected to the cells for 24 h. After the optional further PCV2 infection, the cells were fixed with 4% paraformaldehyde for 30 min and permeabilized with 0.1% Triton X-100 for 20 min. Then the cells were incubated with primary antibodies after blocking with 2% BSA and were incubated with corresponding fluorescent secondary antibodies, followed by staining with 4,6-diamidino-2-phenylindole (DAPI) for 10 min and finally examined with confocal microscopes.

### Co-immunoprecipitation (co-IP) assay

The PK-15^*npm1*-/-^ cells were seeded in 10 cm cell culture dishes, and then co-transfected with plasmids for 24 h. After the optional further PCV2 infection, the cells were harvested and lysed in an ice bath for 30 min, then centrifugated at 12000 rpm for 10 min, and the supernatants were collected. The obtained supernatants were incubated with antibodies and Protein G-beads, then the beads were washed five times and the proteins were eluted for western blot.

### Quantitative PCR (qPCR)

The total DNA was extracted from PCV2-infected cells with phenol-chloroform and treated with RNase to eliminate potential viral RNA contamination. Quantification of copies of the PCV2 genome was performed as previously described [48].

### Fluorescence in situ hybridization (FISH)

Two probes targeted for PCV2 DNA genomes (CP) or DNA replication form (RFP) were designed and synthesized. The probe for CP was labeled by FAM and the sequence was 5’-FAM-CCTTCCTTCCTCTCCCTCGCCAATAAAATAATCAAA-3’. The probe for RFP was labeled by CY3 and the sequence was 5’-CY3-TTTGATTATTTTTGTGGCGAGGAGGTAGGTAGGAGGATAGAGGAGGAGGAG-3’.

The PK-15^*npm1*-/-^ cells were seeded on round coverslips and then transfected with wild-type pNPM1 or the SUMOylation sites mutant pNPM1 (K230R, K263R, and K230/263R) for 24 h. The cells were infected by 1 MOI PCV2 for 12 h and then washed twice. The cells on the coverslips were fixed with ethanol for 30 min and permeabilized with 0.1% Triton X-100 for 20 min. After three times washing, the cells were incubated with 2×SSC at 37°C for 30 min. The cells were then dehydrated with 75%, 90%, and 100% ethanol, respectively. The freshly prepared hybridization buffer containing probes was used for the incubation of the cells in dark at 37°C overnight. The hybridization buffer was replaced by 0.4×SSC containing 0.3% Tween20, which was preheated in advance at 65°C, and the cells were incubated at 37°C for 2 min. The cells were subsequently incubated with preheated 2×SSC containing 0.1% Tween20 at 37°C for 2 min. Finally, the cells were stained with DAPI at 37°C for 15 min. After three times of washing, the cells were prepared for photographs.

### Statistical analysis

The data are presented as the mean ± SD. Statistical analyses were performed with GraphPad Prism 8 software. Comparisons between two groups were performed by an unpaired Student’s *t*-test, whereas data from multiple groups were analyzed by ANOVA, followed by the Bonferroni post hoc test. Statistically significant and very significant results were defined as *p* < 0.05 and *p* < 0.01.

## Supporting information

S1 Fig The N terminal domain of pNPM1 interacts with PCV2 Cap. The truncates of pNPM1 including the N-terminal domain (NTD) of pNPM1, central intrinsically disordered region (IDR) of pNPM1, the C-terminal domain (CTD) of pNPM1, NTD-IDR, and IDR-CTD were cloned into the pBiFC VC155 vectors, and PCV2 Cap were cloned into the pBiFC VN173 vector. The pBiFC VC155 and pBiFC VN173 vectors were con-transfected to 293T cells for 24 h, and the interaction was detected using a fluorescence microscope. ×200 fold.
(TIF)

S2 Fig PCV2 replicates and promotes pNPM1 SUMOylation in high levels at the late phase of infection. PK-15 cells were infected with 1 MOI PCV2 or mock (the same volume of medium) for 0 h, 24 h, 36 h, and 48 h. (A) The expression levels of pNPM1, SUMOylated pNPM1, and PCV2 Cap were detected by western blot. (B) The PCV2 copy numbers were detected by qPCR. **p < 0.01 versus 24 h post-infection.
(TIF)

S3 Fig PCV2 infection promotes the interaction of SUMO2/3. The PK-15 cells were infected with 1MOI PCV2 or mock (the same volume of medium) for 12 h, then the interaction of NPM1 and SUMO1 or SUMO2/3 were measured by co-IP assays using specific antibodies of SUMO1 or SUMO2/3.
(TIF)

S4 Fig TRIM24, but not TRIM28 and TRIM62, is the E3 ligase of SUMOylation for pNPM1. (**A**) 1 MOI PCV2 or mock (the same volume of medium) infected PK-15 cells for 0 h, 6 h, 12 h, 18 h, and 24 h. The expression levels of ARF and TRIM28 were analyzed by western blot. (**B**) The plasmids expressing TRIM28 and pNPM1 were transfected into PK-15^*npm1-/-*^ cells for 24 h, and then the cells were infected with 1 MOI PCV2 or mock for 12 h. The interactions of TRIM28 with pNPM1 were detected by co-IP assays. (**C**) The specific siRNAs targeting TRIM28 were transfected into PK-15 cells for 24 h, then the cells were infected with 1 MOI PCV2 for 12 h. The expression levels of TRIM28, pNPM1, and SUMOylated pNPM1 were detected by western blot. (**D**) The interactions of pNPM1 and TRIM24 or TRIM62 in PK-15 cells were analyzed by co-IP assays using specific antibodies of pNPM1. (**E**) The PK-15 cells were infected with 1MOI PCV2 or mock for 12 h, then the interactions of NPM1 and TRIM24 or TRIM62 were measured by co-IP assays using specific antibodies of pNPM1. (**F**) The specific siRNA targeting Ubc9 was transfected into PK-15 cells for 24 h, then the cells were infected with 1 MOI PCV2 for 12 h. The interactions of NPM1 and TRIM24 were measured by co-IP assays using specific antibodies of pNPM1.
(TIF)

S5 Fig PCV2 Cap is the major component that promotes the interaction of pNPM1 and TRIM24. (**A**) The PK-15 cells were infected with 100 MOI of the blank recombinant adenovirus (rAd-blank) or the recombinant adenovirus expressing PCV2 Cap (rAd-Cap) for 24 h, the interaction of pNPM1 and TRIM24 was measured by co-IP assays using specific antibodies of pNPM1. (**B**) PK-15 cells were pre-treated with Control, DMSO, or specific inhibitors for p38 (SB203580, 10 μM), ERK (PD98059, 20 μM), JNK (SP6000125, 10 μM), and PI3K/Akt (LY294002, 10 μM) for 2 h, respectively. Then the cells were infected with 1 MOI PCV2 for 12 h with the presence of the inhibitors. The expression levels of p-Akt, Akt, p-ERK, ERK, p-p38, p38, p-JNK, and JNK were analyzed by western blot. (**C**) The PK-15 cells were pretreated with DMSO or PD98059 for 2 h, and then the cells were infected with 1 MOI PCV2 for 12 h with the presence of PD98059. The interaction of pNPM1 and TRIM24 was measured by co-IP assays using specific antibodies of pNPM1.
(TIF)

S6 Fig The siERK treatment suppresses the nucleolar localization of pNPM1. (**A**) PK-15 cells were transfected with the siRNA specific for ERK (siERK) or negative control siRNA (siNC) for 24 h and then infected with 100 MOI rAd-Cap or rAd-blank for another 24 h. The localizations of pNPM1 in these cells were measured by confocal microscopy. The bars = 10 μm were indicated in each panel. (**B**) The rAd-blank-infected cells were harvested and lysed with a nuclear and cytoplasmic protein extraction kit, then the expression of the pNPM1 and Cap were measured by western blot. (**C**) PK-15 cells were transfected with the specific siERK or siNC for 24 h and then infected with 1 MOI PCV2 for 12 h. The localizations of pNPM1 in these cells were measured by confocal microscopy. The bars = 10 μm were indicated in each panel. (**D**) The mock-infected cells were harvested and lysed with a nuclear and cytoplasmic protein extraction kit, then the expression of the pNPM1 and Cap were measured by western blot.
(TIF)

S7 Fig The effect of 2-D08 and ML-792 on cell viability. PK-15 cells were treated with various concentrates of 2-D08 (A) or ML-792 (B) for 24 h, the cell viabilities were measured by the MTT assay.
(TIF)

S1 Data Data for Figs 1B, 1D, 1F, 1G, 2B, 2C, 2E, 2F, 3A–3G, 4A–4F, 5A–5F, 6A–6G and 7E–7J. (ZIP)

S2 Data Data for Fig 7A–7D. (ZIP)

S3 Data Data for S1, S2, S3, S4A–S4F, S5A–S5C and S7 Figs. (ZIP)

S4 Data Data for S6A and S6B Fig. (ZIP)

S5 Data Data for S6C and S6D Fig. (ZIP)
