# Supplementary figures and images for: Porcine circovirus type 2 infection promotes the SUMOylation of nucleophosmin-1 to facilitate the viral circular single-stranded DNA replication (part 1 of 2)
Source: PLoS Pathog. 2024 Feb 23;20(2):e1012014. doi: 10.1371/journal.ppat.1012014 (PMC10917307; doi:10.1371/journal.ppat.1012014)

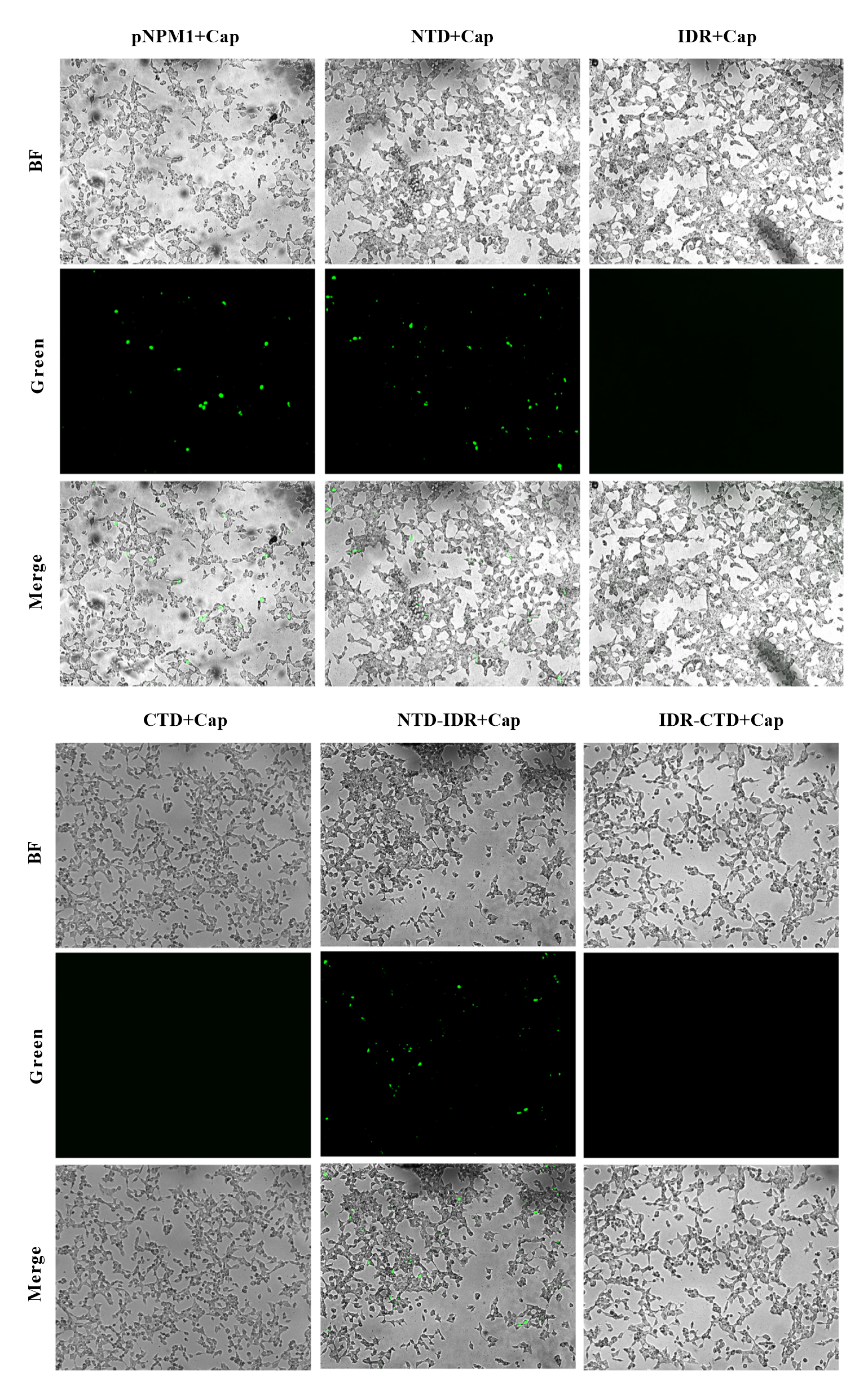

Supplement: S1 Fig — The truncates of pNPM1 including the N-terminal domain (NTD) of pNPM1, central intrinsically disordered region (IDR) of pNPM1, the C-terminal domain (CTD) of pNPM1, NTD-IDR, and IDR-CTD were cloned into the pBiFC VC155 vectors, and PCV2 Cap were cloned into the pBiFC VN173 vector. The pBiFC VC155 and pBiFC VN173 vectors were con-transfected to 293T cells for 24 h, and the interaction was detected using a fluorescence microscope. ×200 fold. (TIF) [file ppat.1012014.s001.tif]

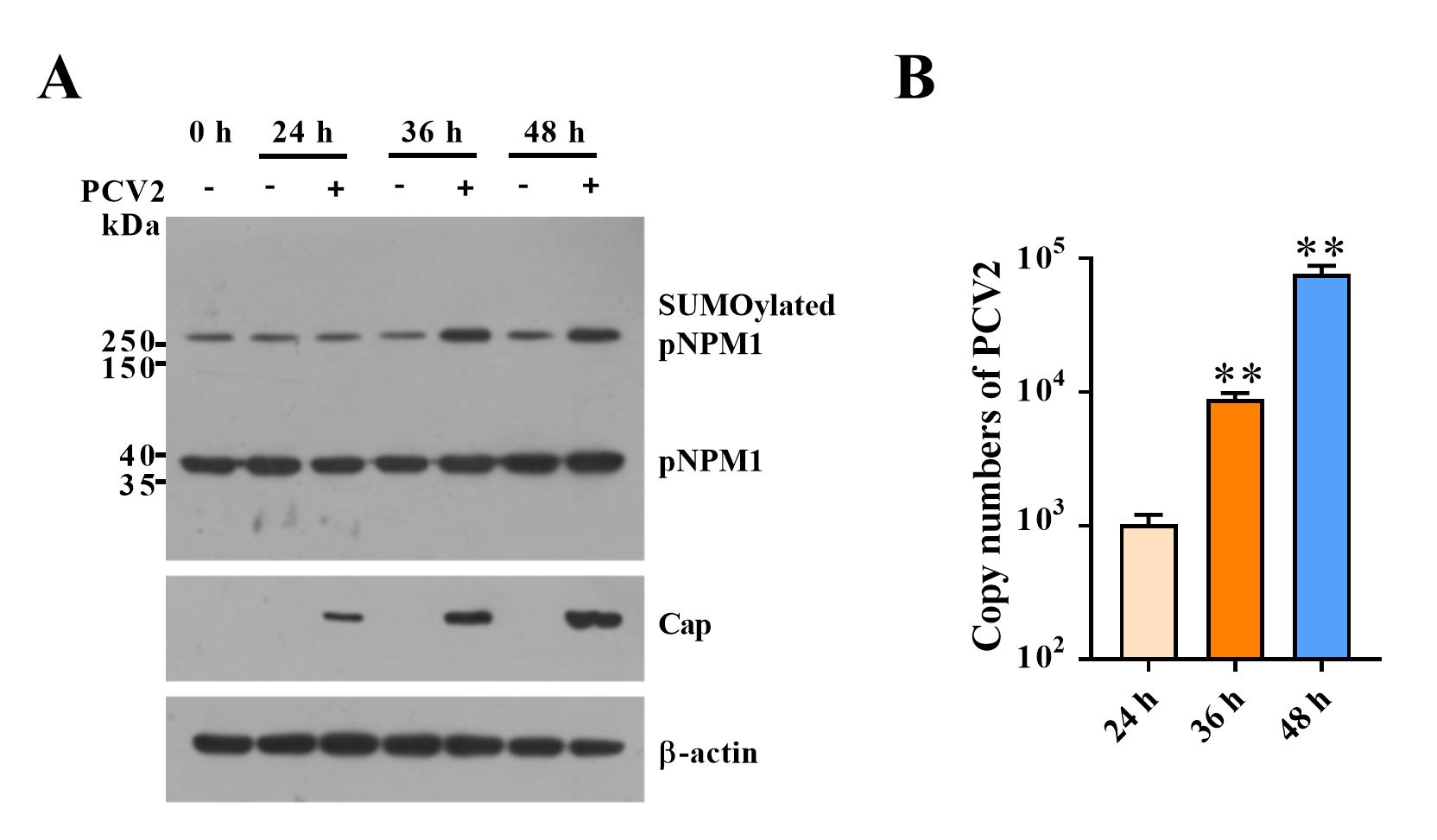

Supplement: S2 Fig — PK-15 cells were infected with 1 MOI PCV2 or mock (the same volume of medium) for 0 h, 24 h, 36 h, and 48 h. (A) The expression levels of pNPM1, SUMOylated pNPM1, and PCV2 Cap were detected by western blot. (B) The PCV2 copy numbers were detected by qPCR. **p < 0.01 versus 24 h post-infection. (TIF) [file ppat.1012014.s002.tif]

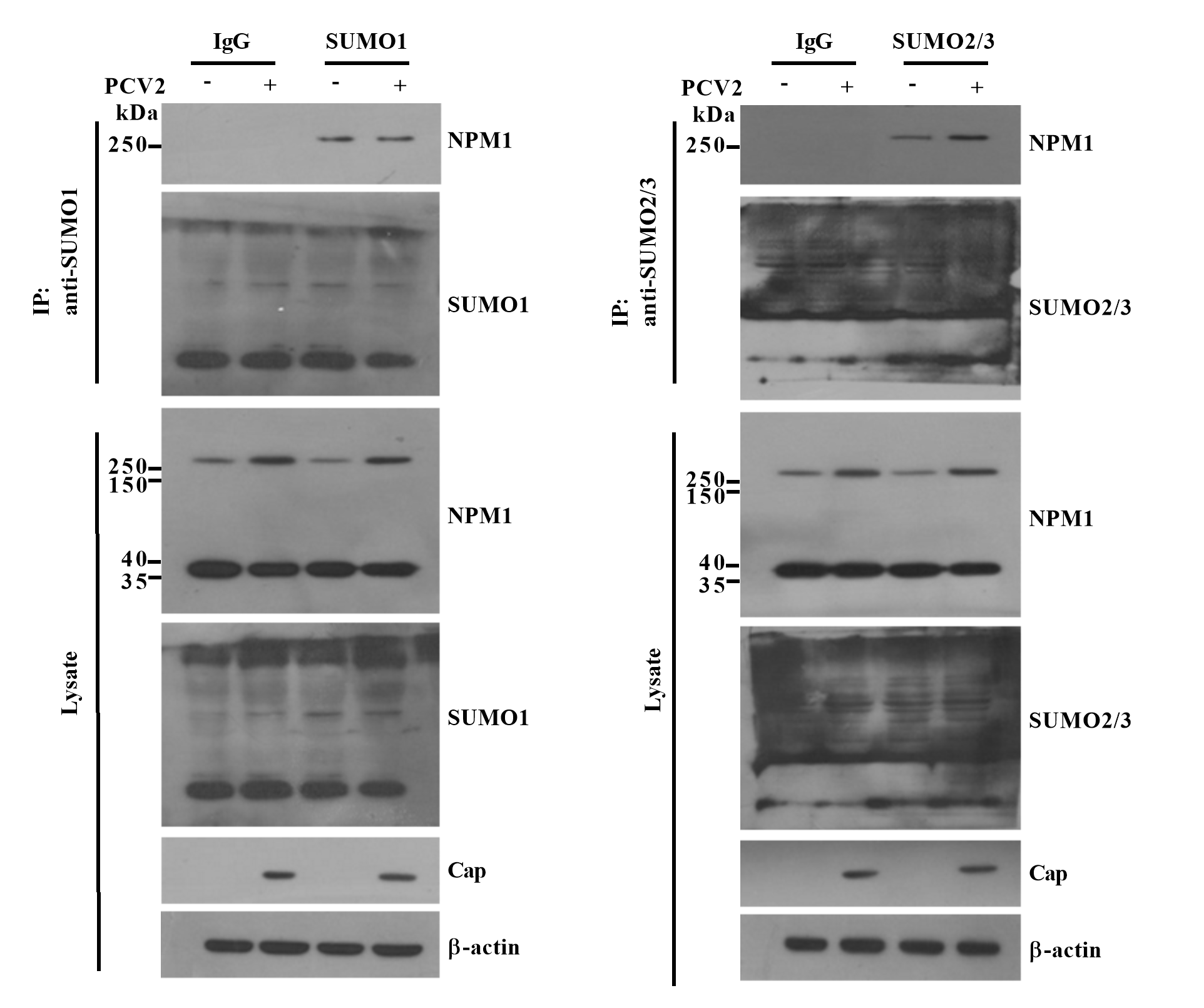

Supplement: S3 Fig — The PK-15 cells were infected with 1MOI PCV2 or mock (the same volume of medium) for 12 h, then the interaction of NPM1 and SUMO1 or SUMO2/3 were measured by co-IP assays using specific antibodies of SUMO1 or SUMO2/3. (TIF) [file ppat.1012014.s003.tif]

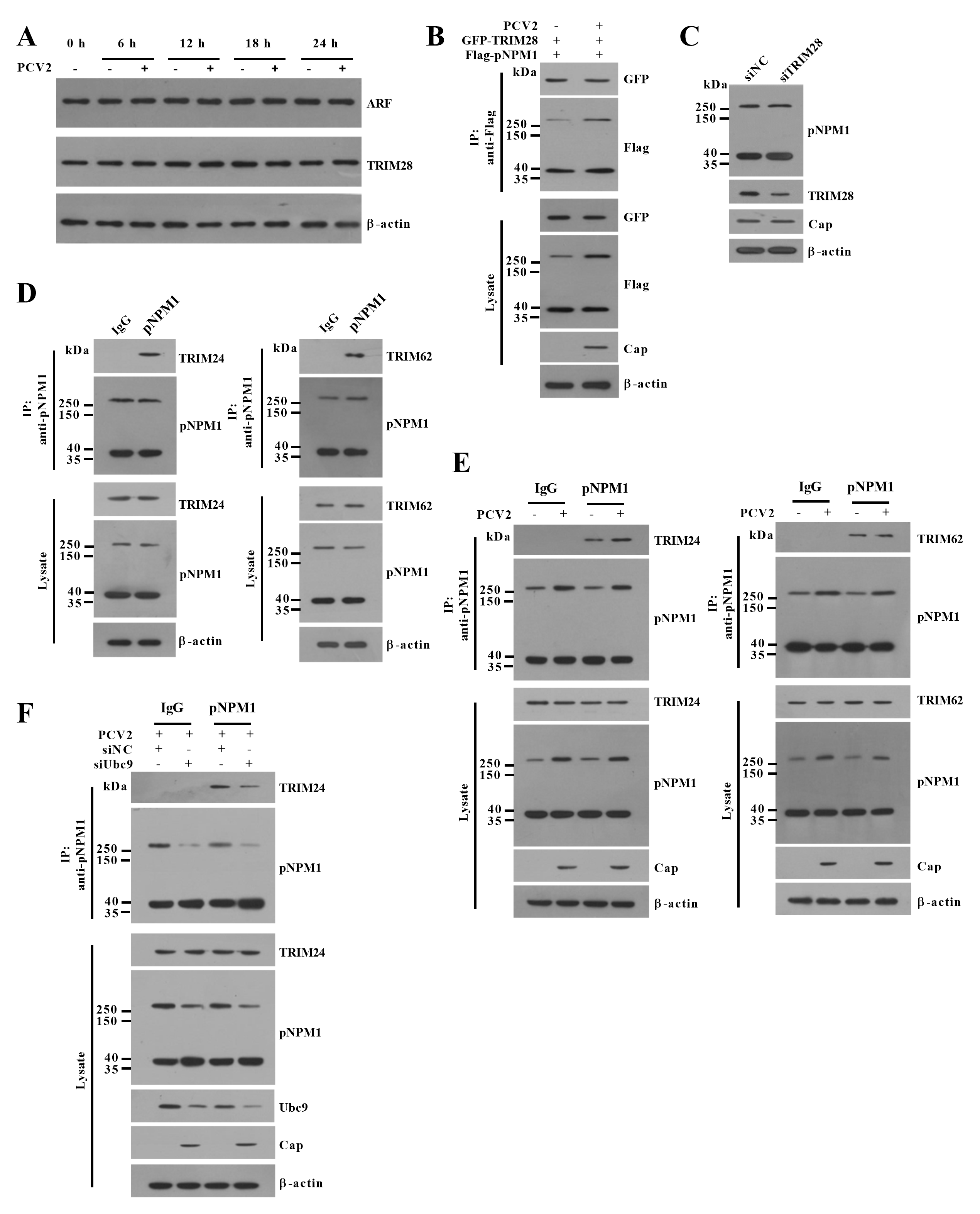

Supplement: S4 Fig — (A) 1 MOI PCV2 or mock (the same volume of medium) infected PK-15 cells for 0 h, 6 h, 12 h, 18 h, and 24 h. The expression levels of ARF and TRIM28 were analyzed by western blot. (B) The plasmids expressing TRIM28 and pNPM1 were transfected into PK-15npm1-/- cells for 24 h, and then the cells were infected with 1 MOI PCV2 or mock for 12 h. The interactions of TRIM28 with pNPM1 were detected by co-IP assays. (C) The specific siRNAs targeting TRIM28 were transfected into PK-15 cells for 24 h, then the cells were infected with 1 MOI PCV2 for 12 h. The expression levels of TRIM28, pNPM1, and SUMOylated pNPM1 were detected by western blot. (D) The interactions of pNPM1 and TRIM24 or TRIM62 in PK-15 cells were analyzed by co-IP assays using specific antibodies of pNPM1. (E) The PK-15 cells were infected with 1MOI PCV2 or mock for 12 h, then the interactions of NPM1 and TRIM24 or TRIM62 were measured by co-IP assays using specific antibodies of pNPM1. (F) The specific siRNA targeting Ubc9 was transfected into PK-15 cells for 24 h, then the cells were infected with 1 MOI PCV2 for 12 h. The interactions of NPM1 and TRIM24 were measured by co-IP assays using specific antibodies of pNPM1. (TIF) [file ppat.1012014.s004.tif]

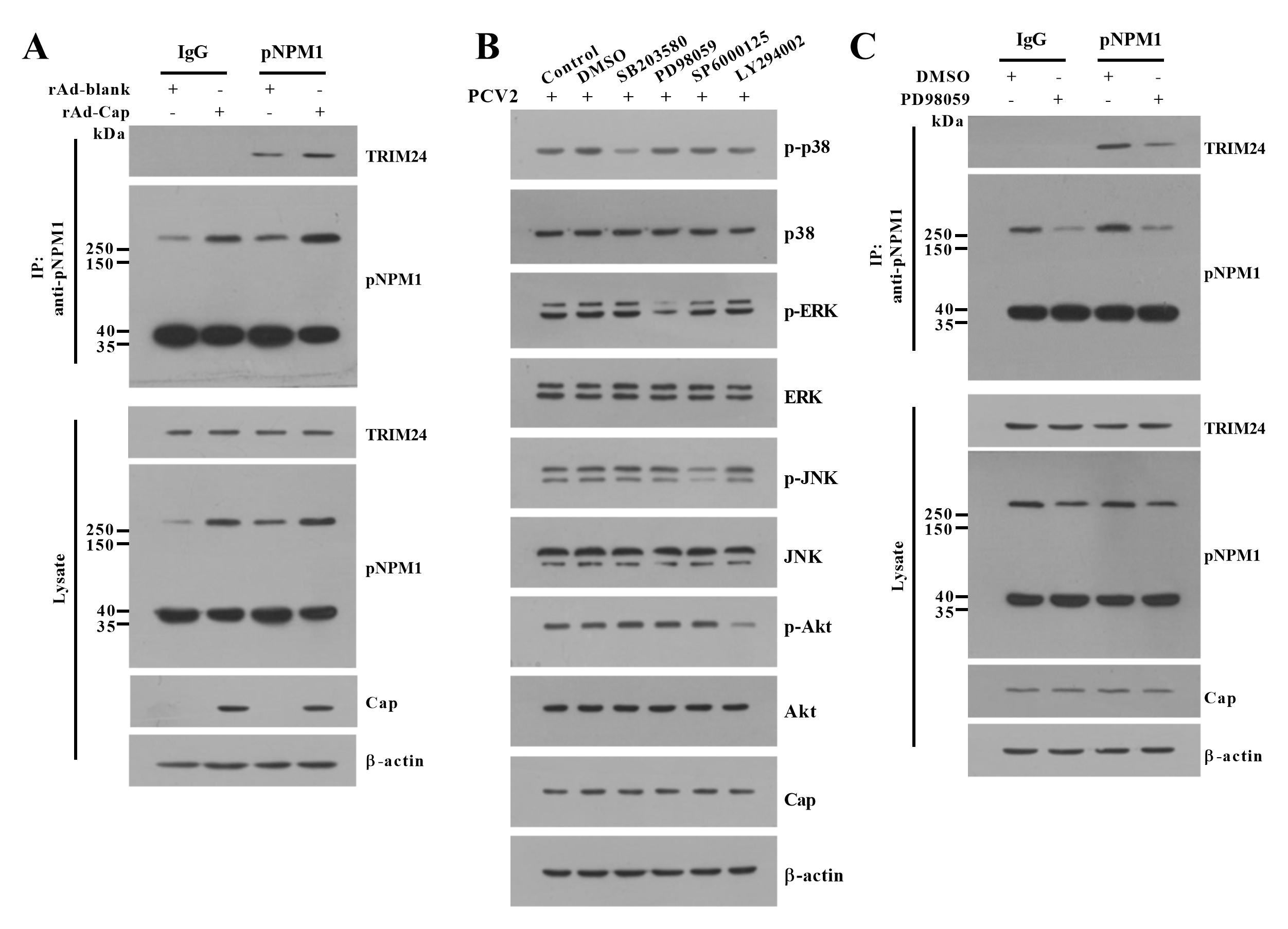

Supplement: S5 Fig — (A) The PK-15 cells were infected with 100 MOI of the blank recombinant adenovirus (rAd-blank) or the recombinant adenovirus expressing PCV2 Cap (rAd-Cap) for 24 h, the interaction of pNPM1 and TRIM24 was measured by co-IP assays using specific antibodies of pNPM1. (B) PK-15 cells were pre-treated with Control, DMSO, or specific inhibitors for p38 (SB203580, 10 μM), ERK (PD98059, 20 μM), JNK (SP6000125, 10 μM), and PI3K/Akt (LY294002, 10 μM) for 2 h, respectively. Then the cells were infected with 1 MOI PCV2 for 12 h with the presence of the inhibitors. The expression levels of p-Akt, Akt, p-ERK, ERK, p-p38, p38, p-JNK, and JNK were analyzed by western blot. (C) The PK-15 cells were pretreated with DMSO or PD98059 for 2 h, and then the cells were infected with 1 MOI PCV2 for 12 h with the presence of PD98059. The interaction of pNPM1 and TRIM24 was measured by co-IP assays using specific antibodies of pNPM1. (TIF) [file ppat.1012014.s005.tif]

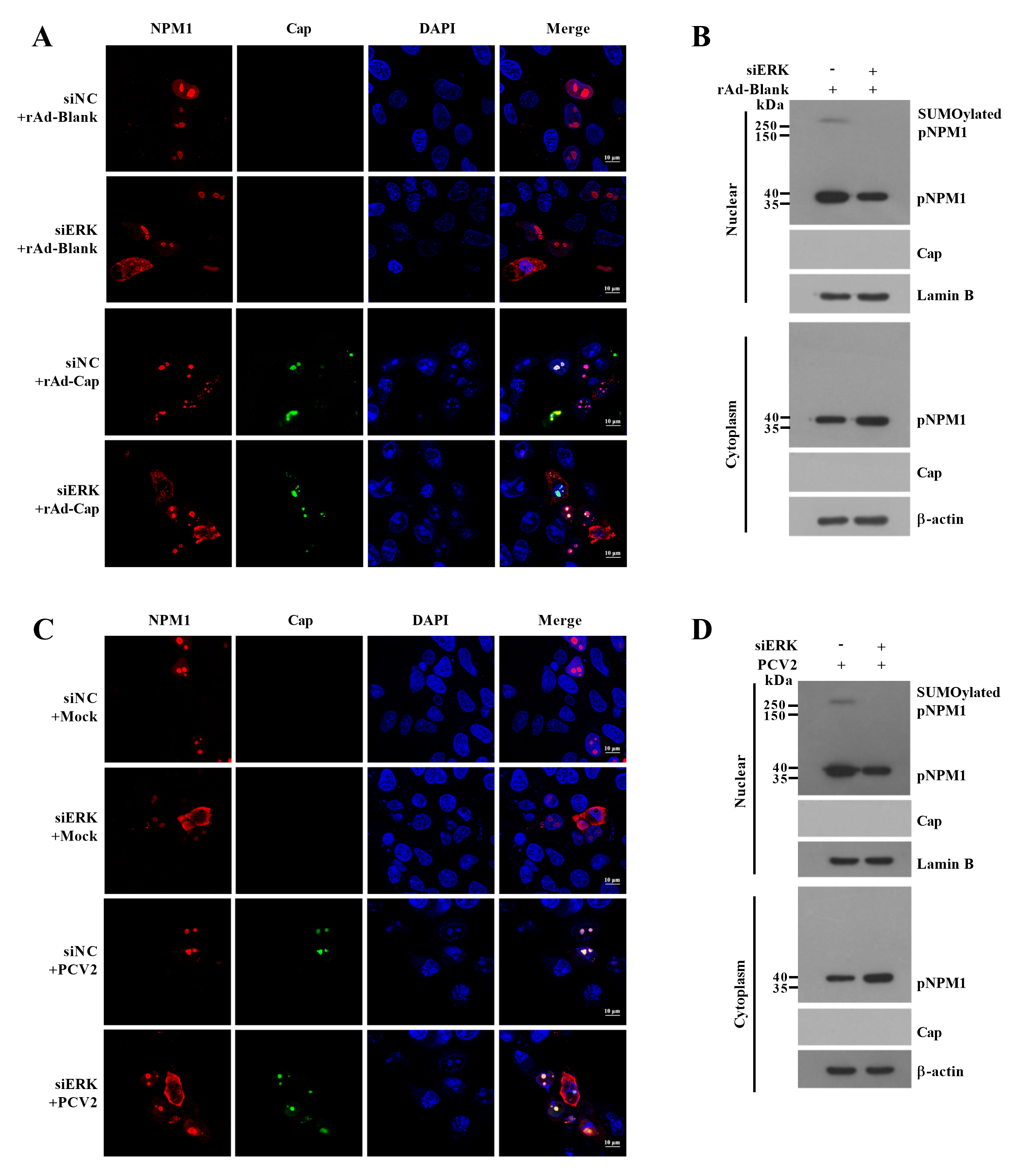

Supplement: S6 Fig — (A) PK-15 cells were transfected with the siRNA specific for ERK (siERK) or negative control siRNA (siNC) for 24 h and then infected with 100 MOI rAd-Cap or rAd-blank for another 24 h. The localizations of pNPM1 in these cells were measured by confocal microscopy. The bars = 10 μm were indicated in each panel. (B) The rAd-blank-infected cells were harvested and lysed with a nuclear and cytoplasmic protein extraction kit, then the expression of the pNPM1 and Cap were measured by western blot. (C) PK-15 cells were transfected with the specific siERK or siNC for 24 h and then infected with 1 MOI PCV2 for 12 h. The localizations of pNPM1 in these cells were measured by confocal microscopy. The bars = 10 μm were indicated in each panel. (D) The mock-infected cells were harvested and lysed with a nuclear and cytoplasmic protein extraction kit, then the expression of the pNPM1 and Cap were measured by western blot. (TIF) [file ppat.1012014.s006.tif]

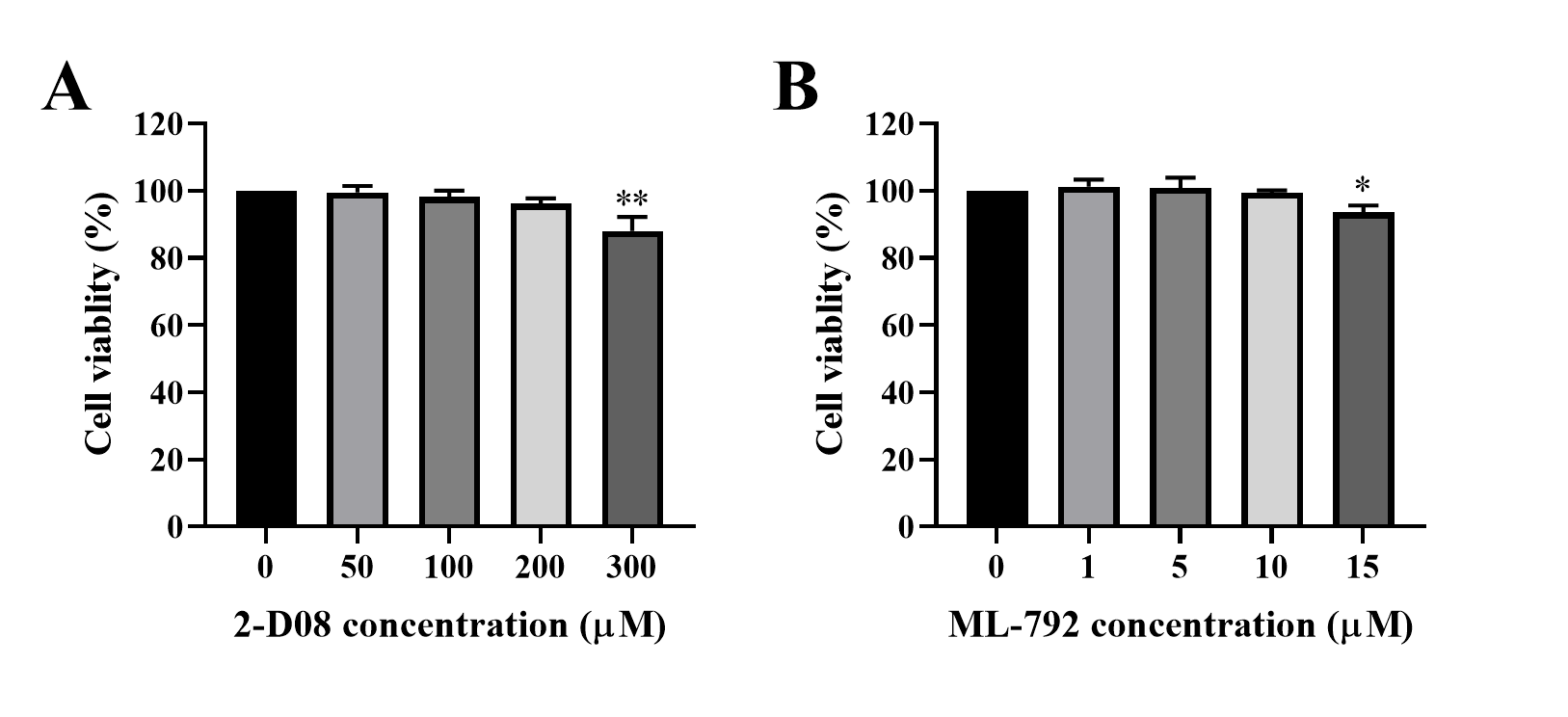

Supplement: S7 Fig — PK-15 cells were treated with various concentrates of 2-D08 (A) or ML-792 (B) for 24 h, the cell viabilities were measured by the MTT assay. (TIF) [file ppat.1012014.s007.tif]

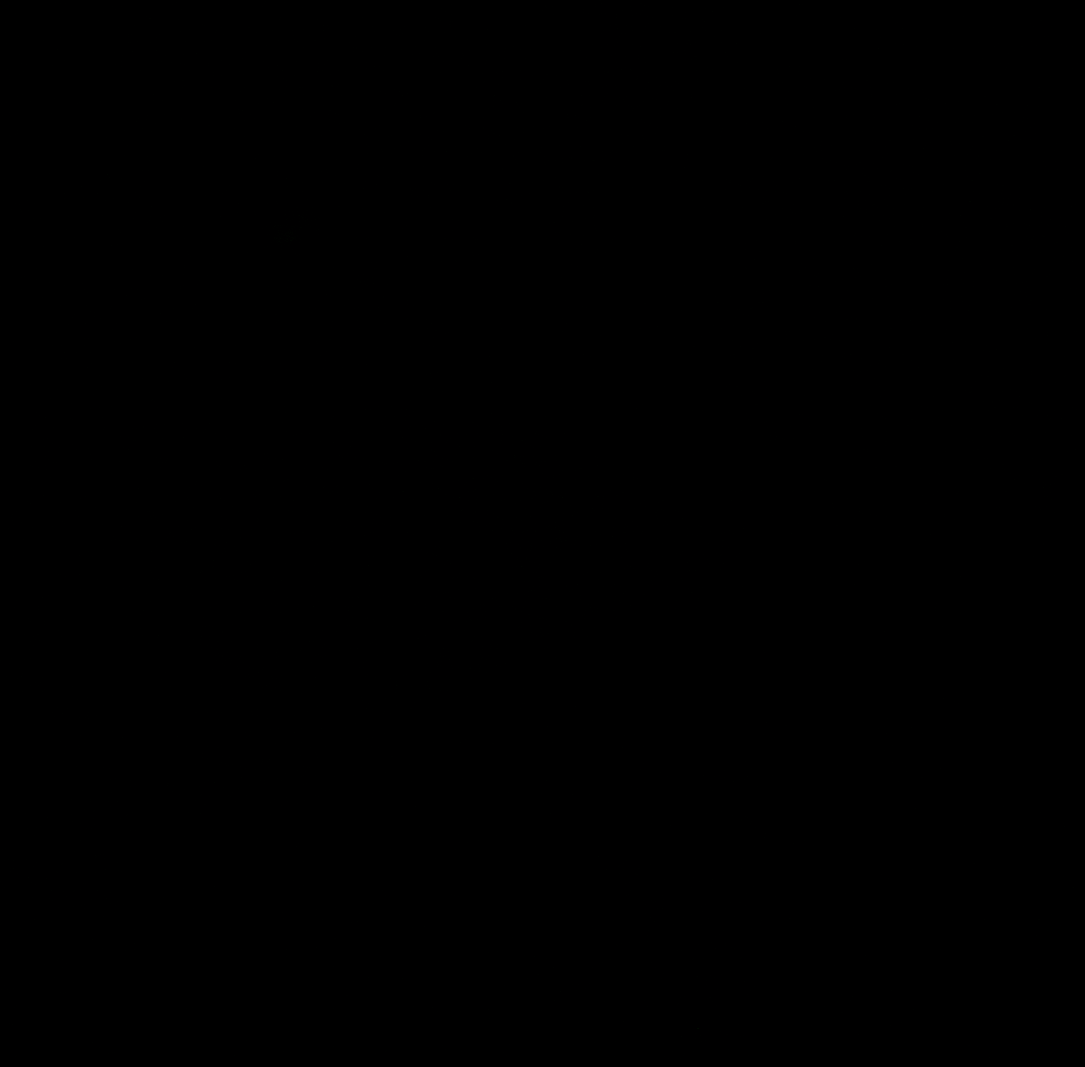

Supplement: S2 Data — (ZIP) [file ppat.1012014.s009.zip › A/A-1/siERK+rAd-Blank Cap.tif]

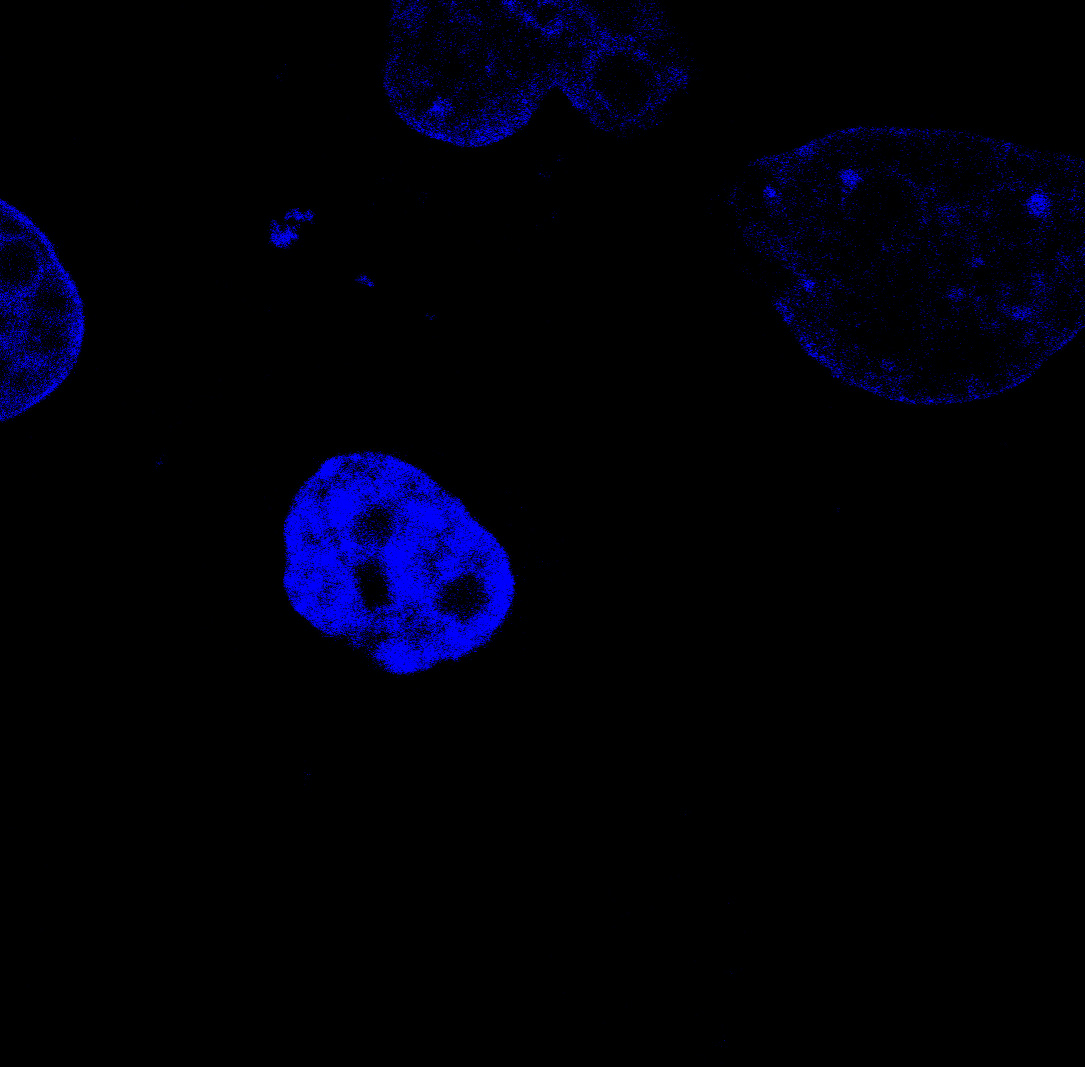

Supplement: S2 Data — (ZIP) [file ppat.1012014.s009.zip › A/A-1/siERK+rAd-Blank DAPI.tif]

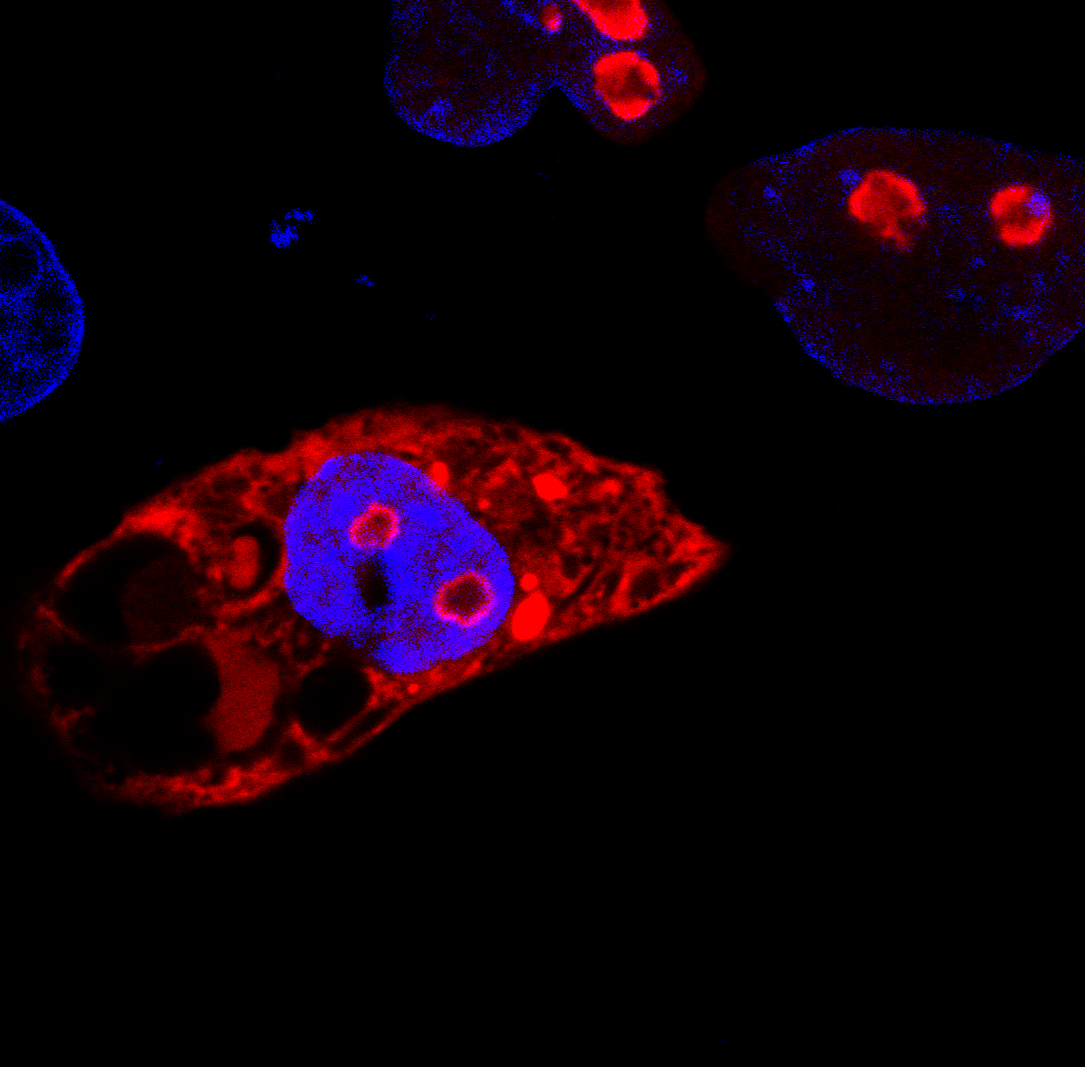

Supplement: S2 Data — (ZIP) [file ppat.1012014.s009.zip › A/A-1/siERK+rAd-Blank Merge.tif]

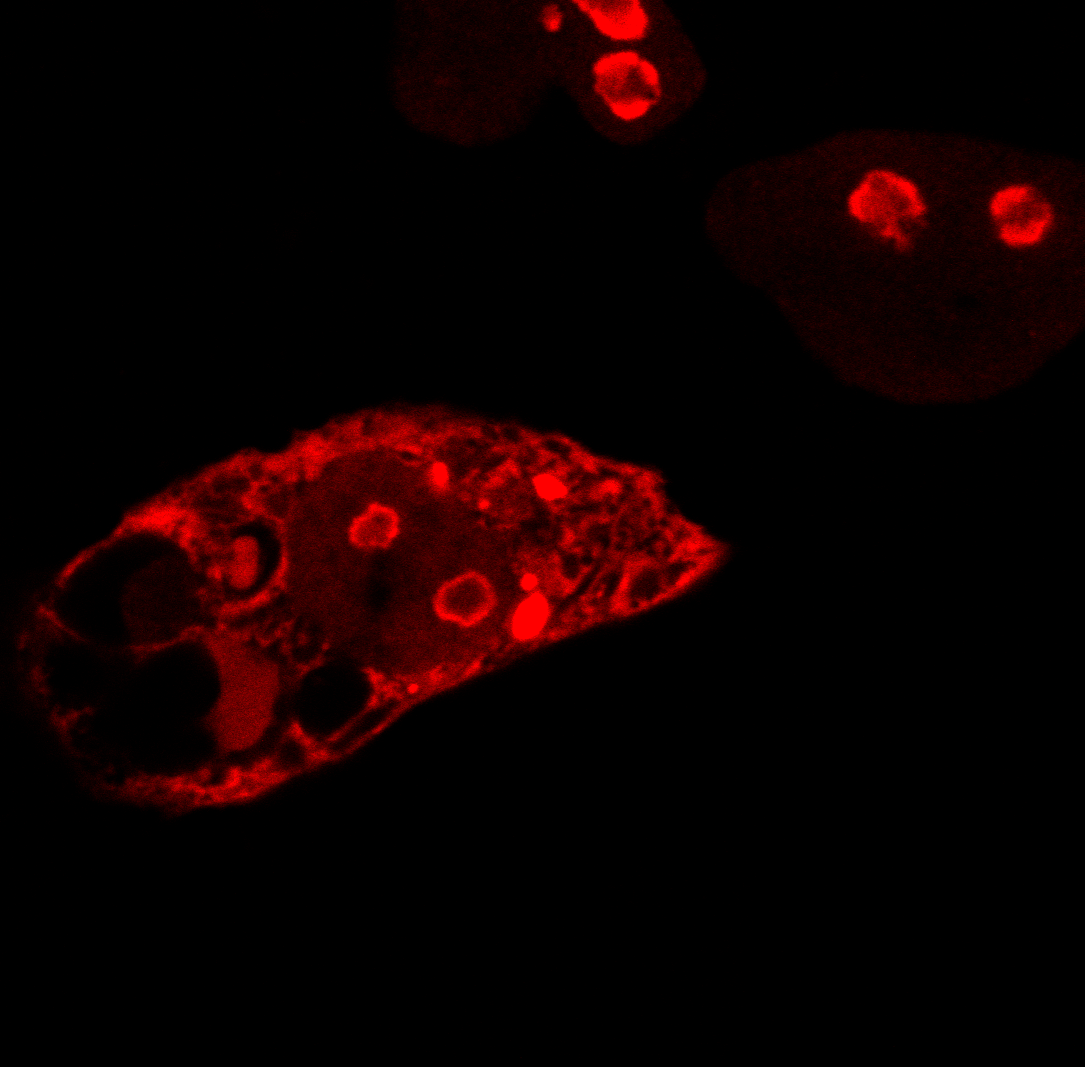

Supplement: S2 Data — (ZIP) [file ppat.1012014.s009.zip › A/A-1/siERK+rAd-Blank NPM1.tif]

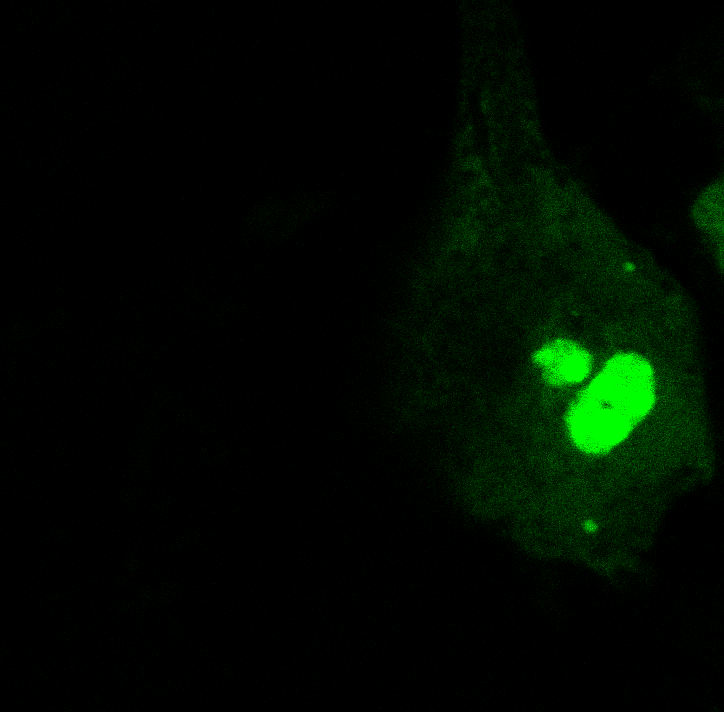

Supplement: S2 Data — (ZIP) [file ppat.1012014.s009.zip › A/A-1/siERK+rAd-Cap Cap.tif]

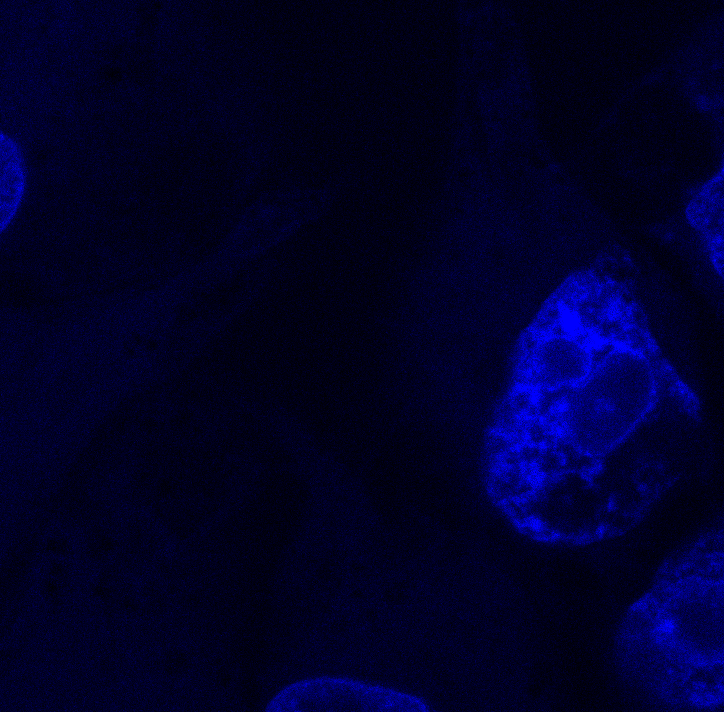

Supplement: S2 Data — (ZIP) [file ppat.1012014.s009.zip › A/A-1/siERK+rAd-Cap DAPI.tif]

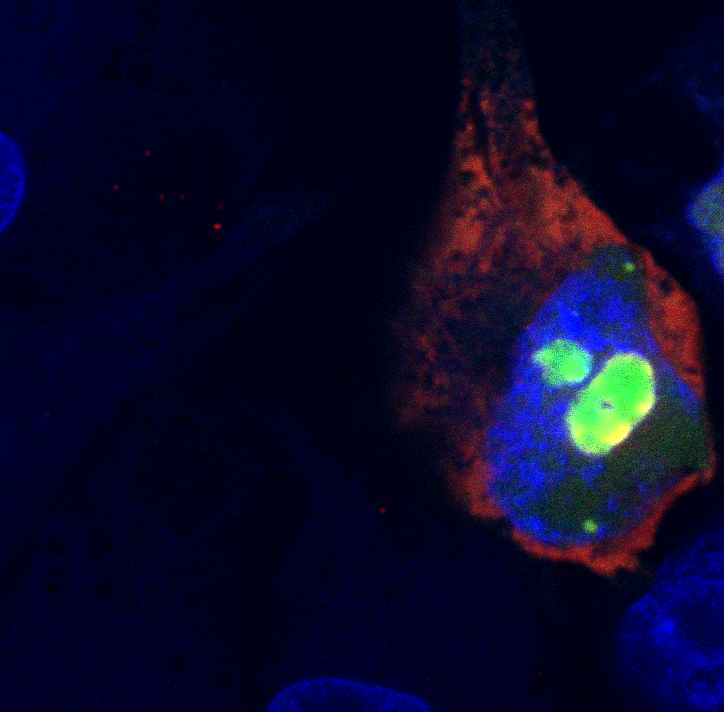

Supplement: S2 Data — (ZIP) [file ppat.1012014.s009.zip › A/A-1/siERK+rAd-Cap Merge.tif]

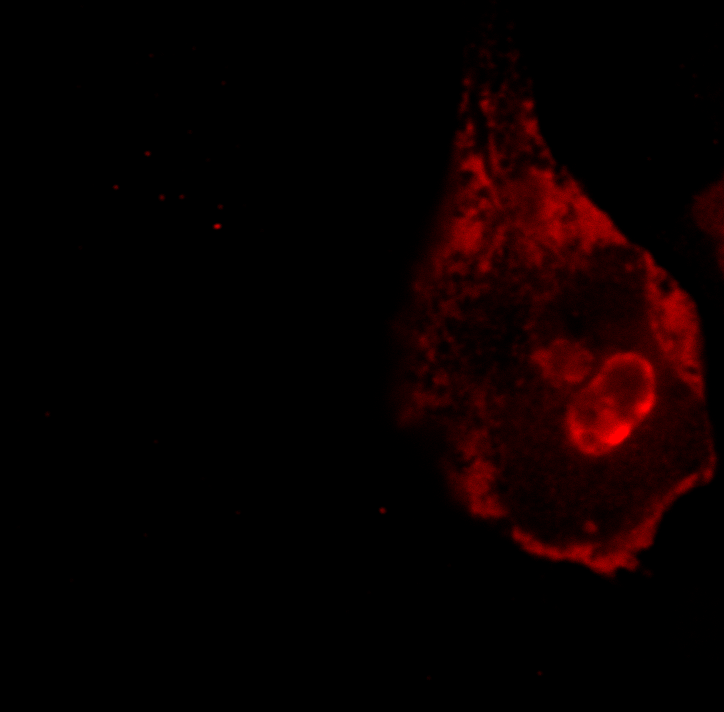

Supplement: S2 Data — (ZIP) [file ppat.1012014.s009.zip › A/A-1/siERK+rAd-Cap NPM1.tif]

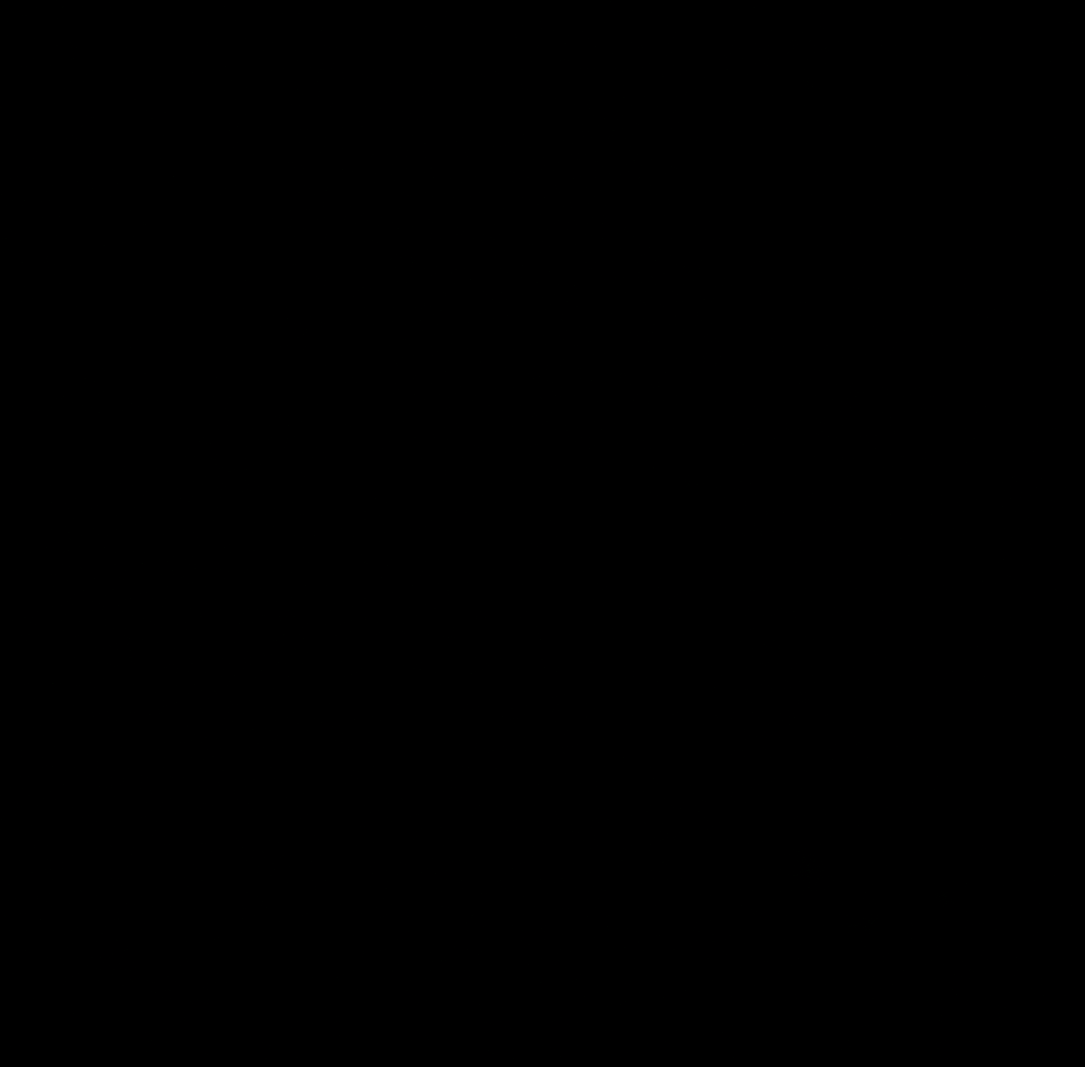

Supplement: S2 Data — (ZIP) [file ppat.1012014.s009.zip › A/A-1/siNC+rAd-Blank Cap.tif]

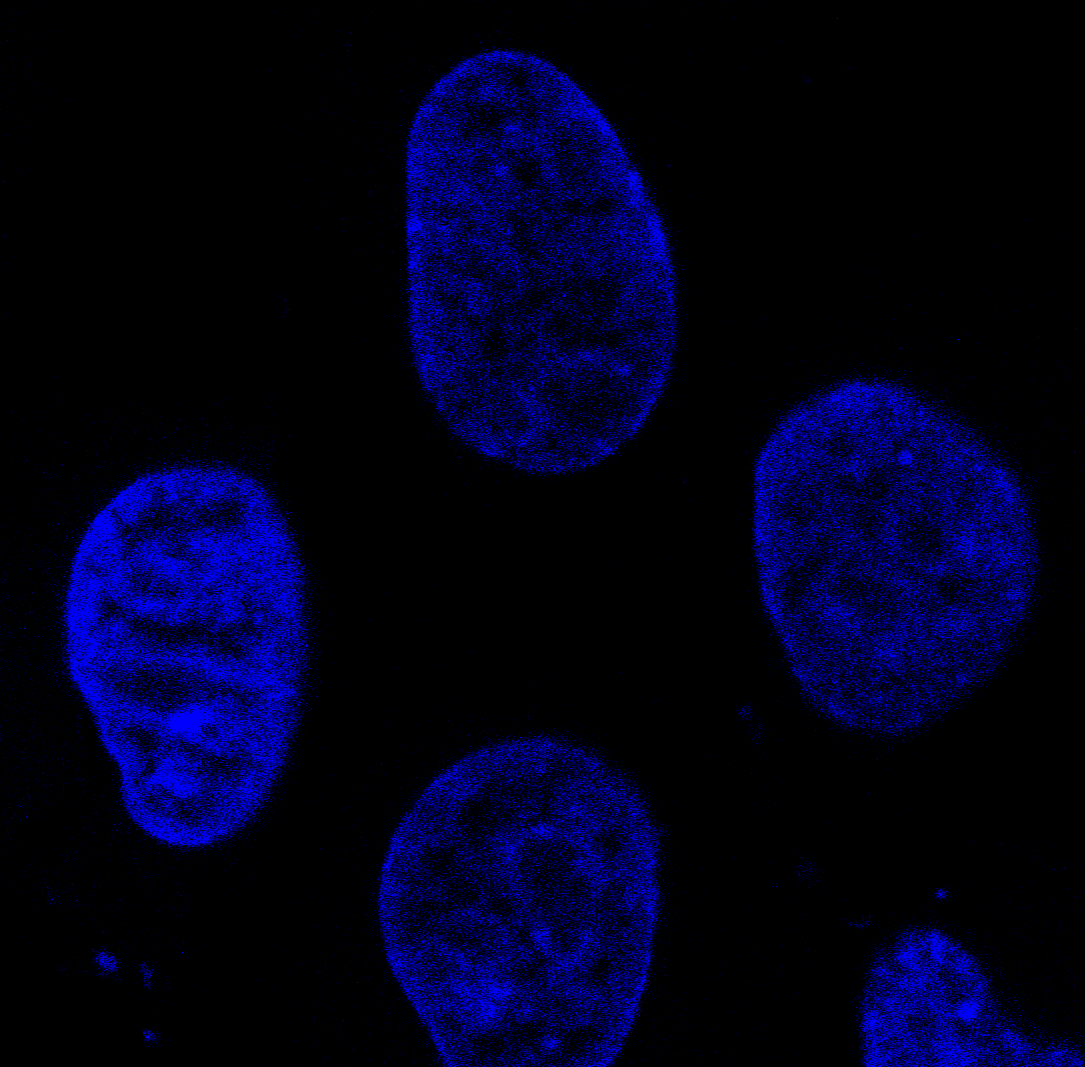

Supplement: S2 Data — (ZIP) [file ppat.1012014.s009.zip › A/A-1/siNC+rAd-Blank DAPI.tif]

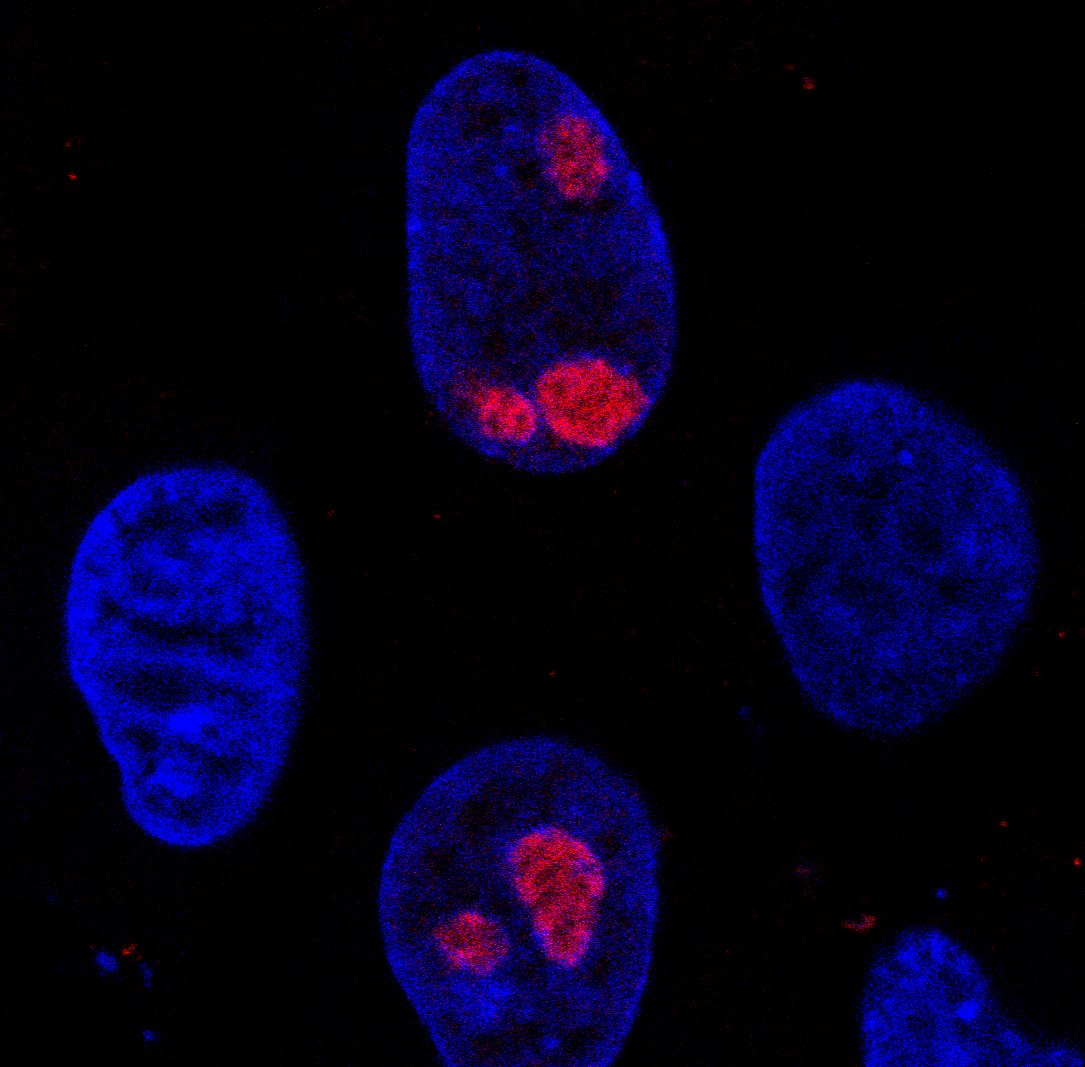

Supplement: S2 Data — (ZIP) [file ppat.1012014.s009.zip › A/A-1/siNC+rAd-Blank Merge.tif]

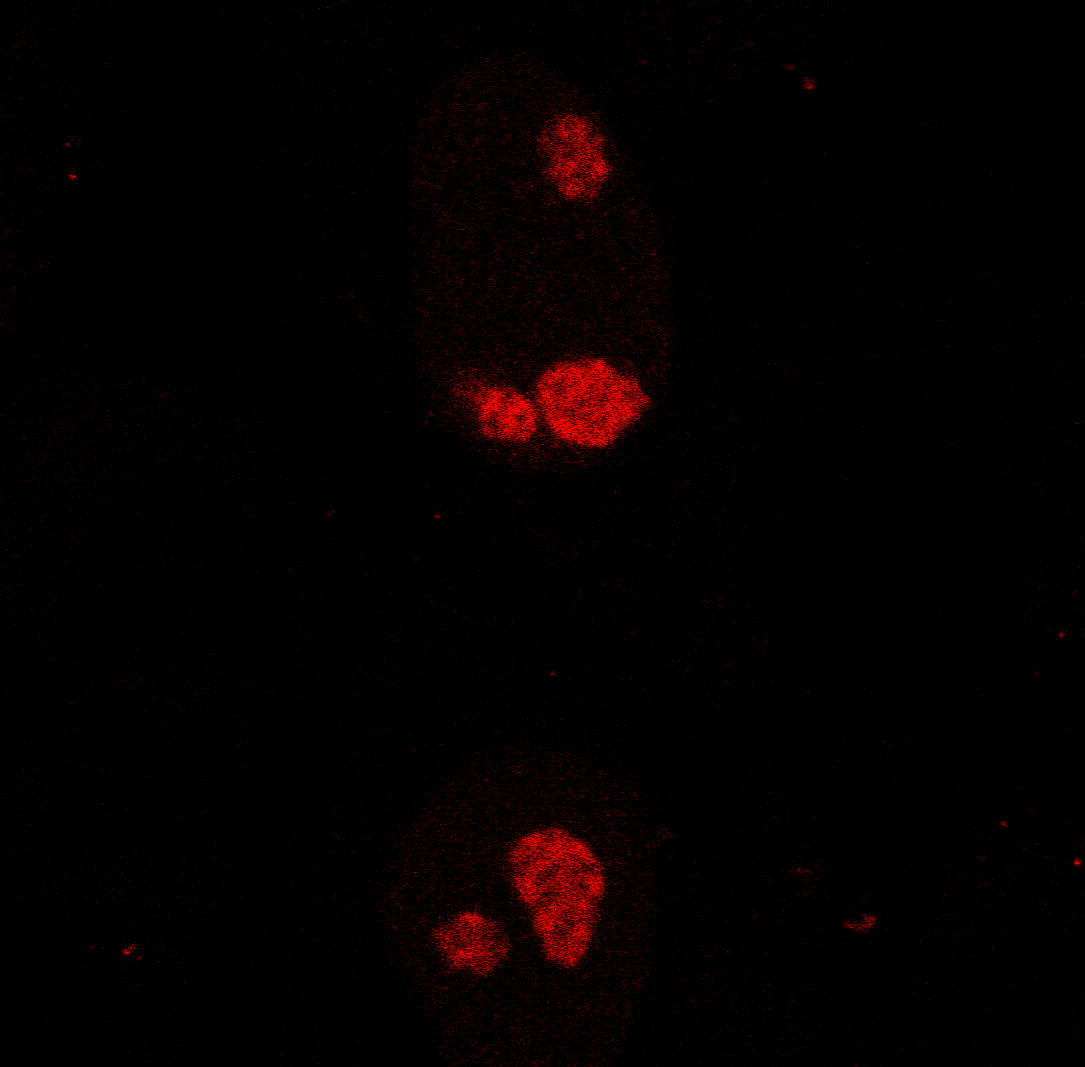

Supplement: S2 Data — (ZIP) [file ppat.1012014.s009.zip › A/A-1/siNC+rAd-Blank NPM1.tif]

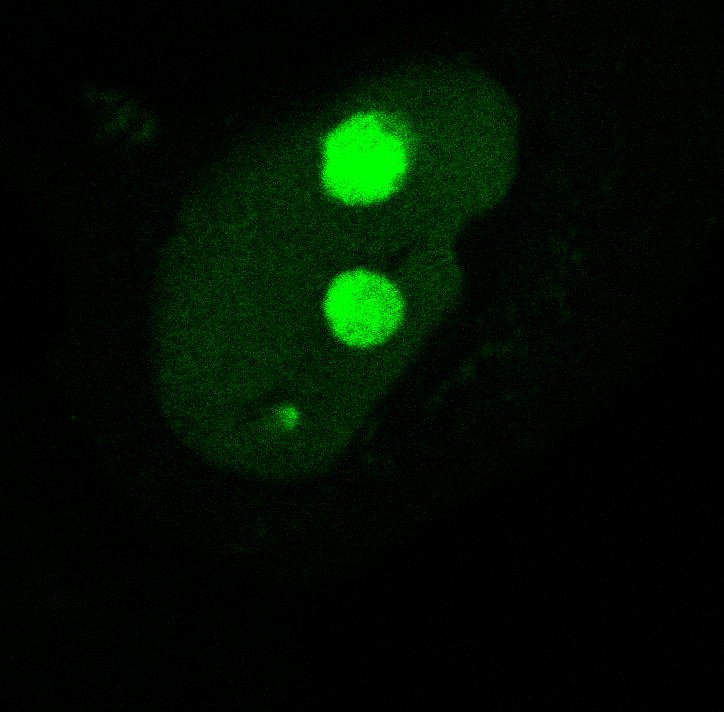

Supplement: S2 Data — (ZIP) [file ppat.1012014.s009.zip › A/A-1/siNC+rAd-Cap Cap.tif]

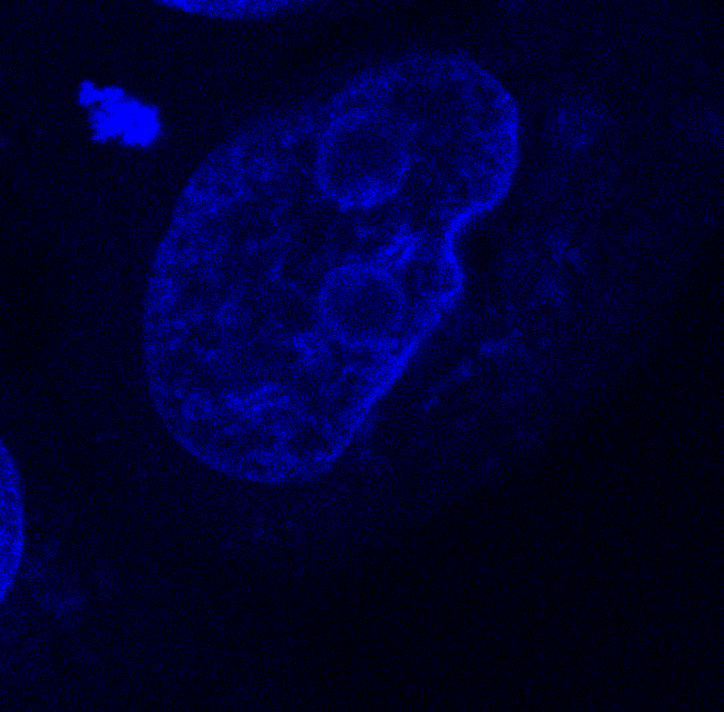

Supplement: S2 Data — (ZIP) [file ppat.1012014.s009.zip › A/A-1/siNC+rAd-Cap DAPI.tif]

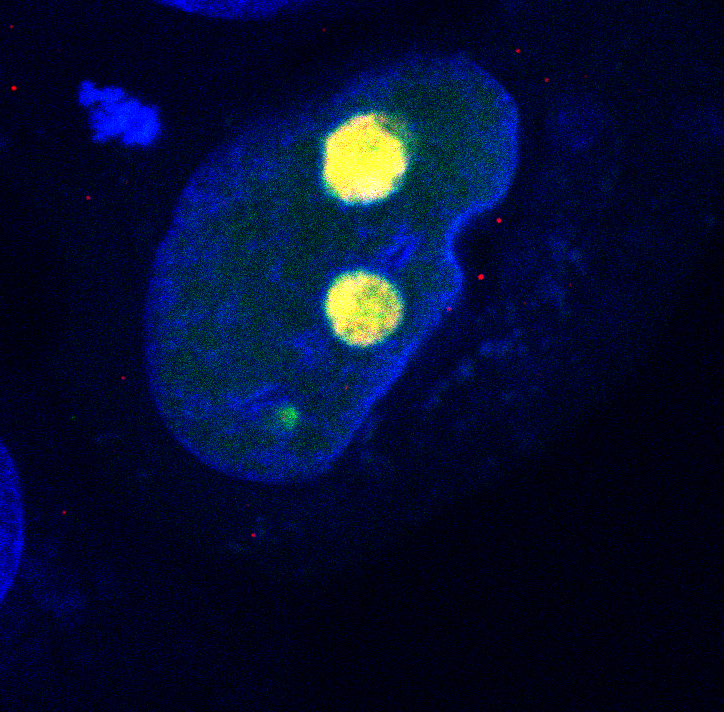

Supplement: S2 Data — (ZIP) [file ppat.1012014.s009.zip › A/A-1/siNC+rAd-Cap Merge.tif]

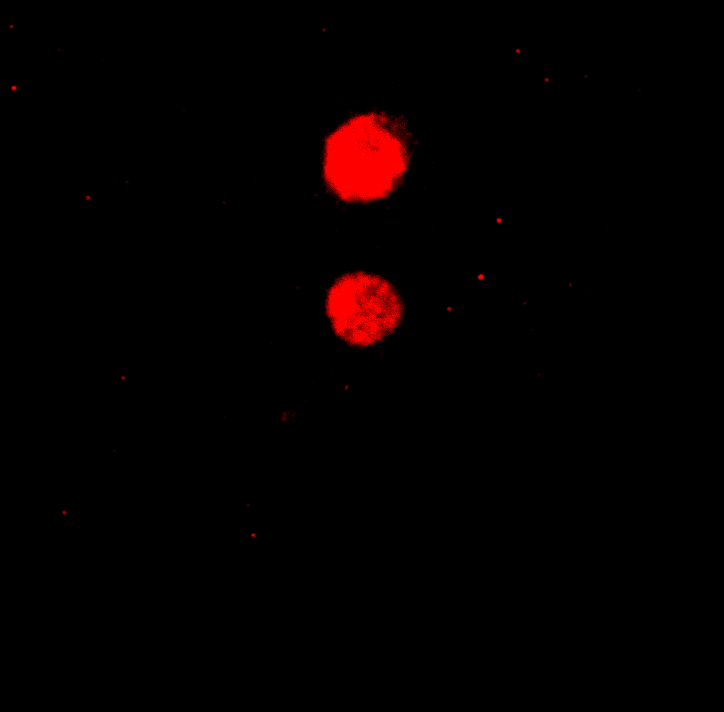

Supplement: S2 Data — (ZIP) [file ppat.1012014.s009.zip › A/A-1/siNC+rAd-Cap NPM1.tif]

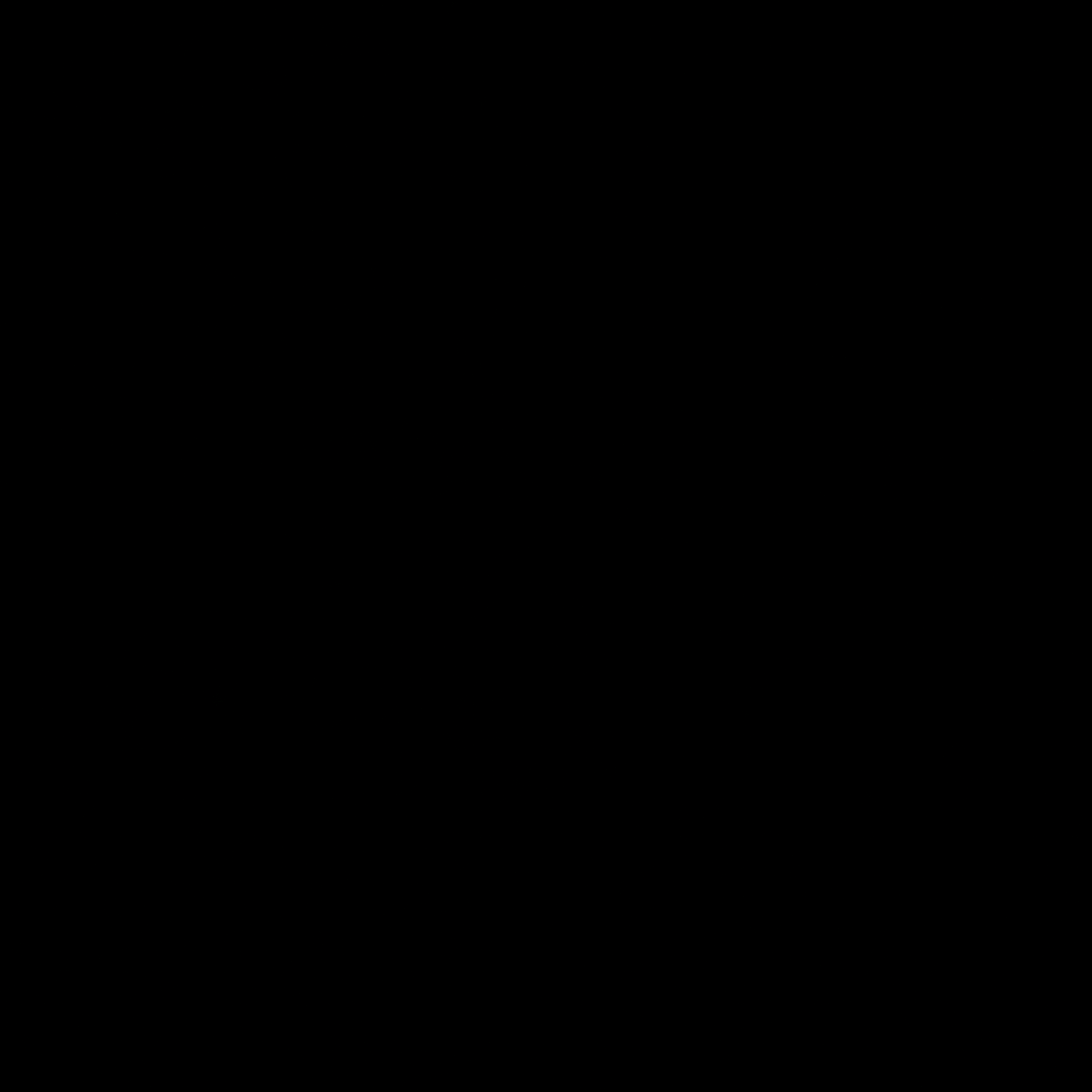

Supplement: S2 Data — (ZIP) [file ppat.1012014.s009.zip › A/A-2/siERK+rAd-Blank Cap.tif]

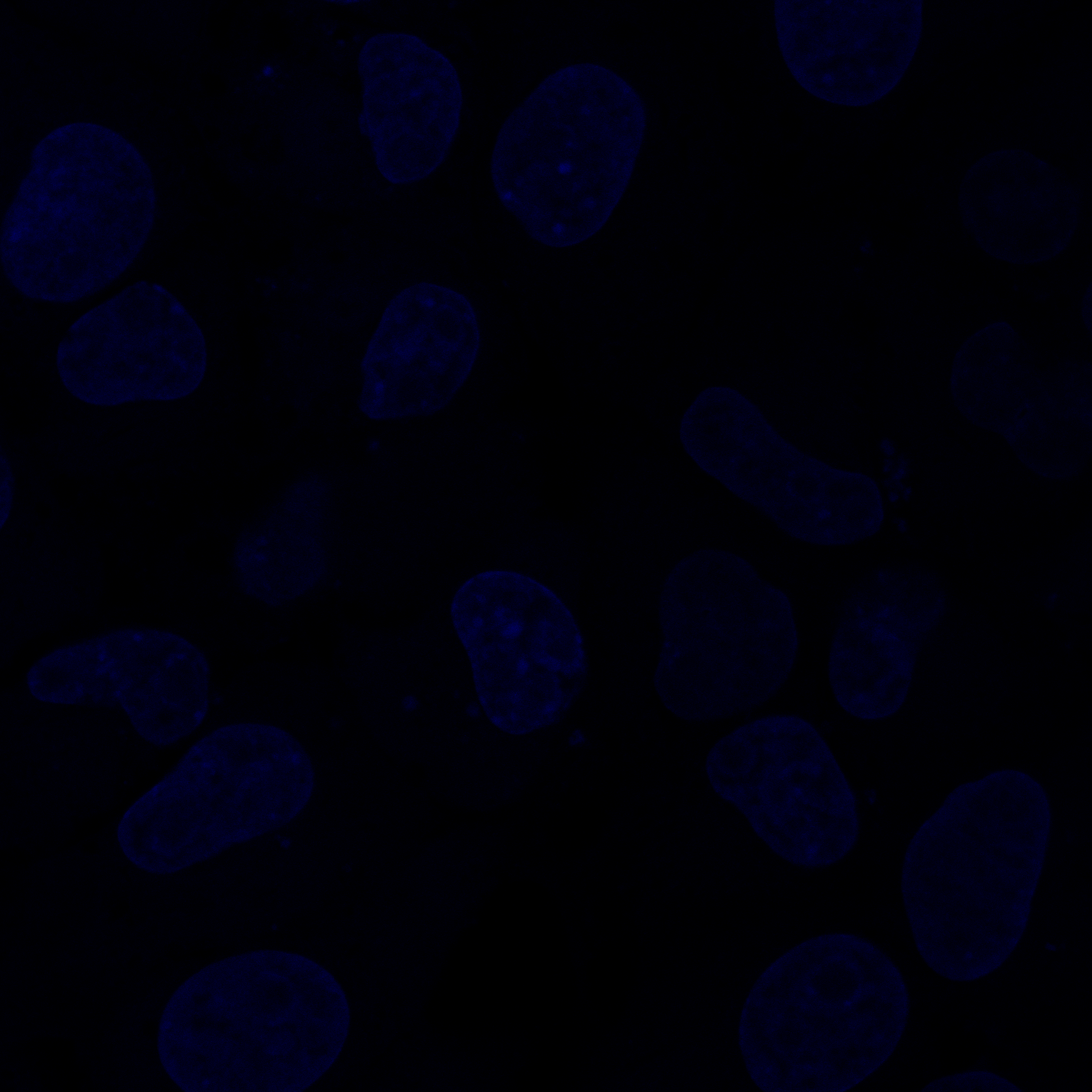

Supplement: S2 Data — (ZIP) [file ppat.1012014.s009.zip › A/A-2/siERK+rAd-Blank DAPI.tif]

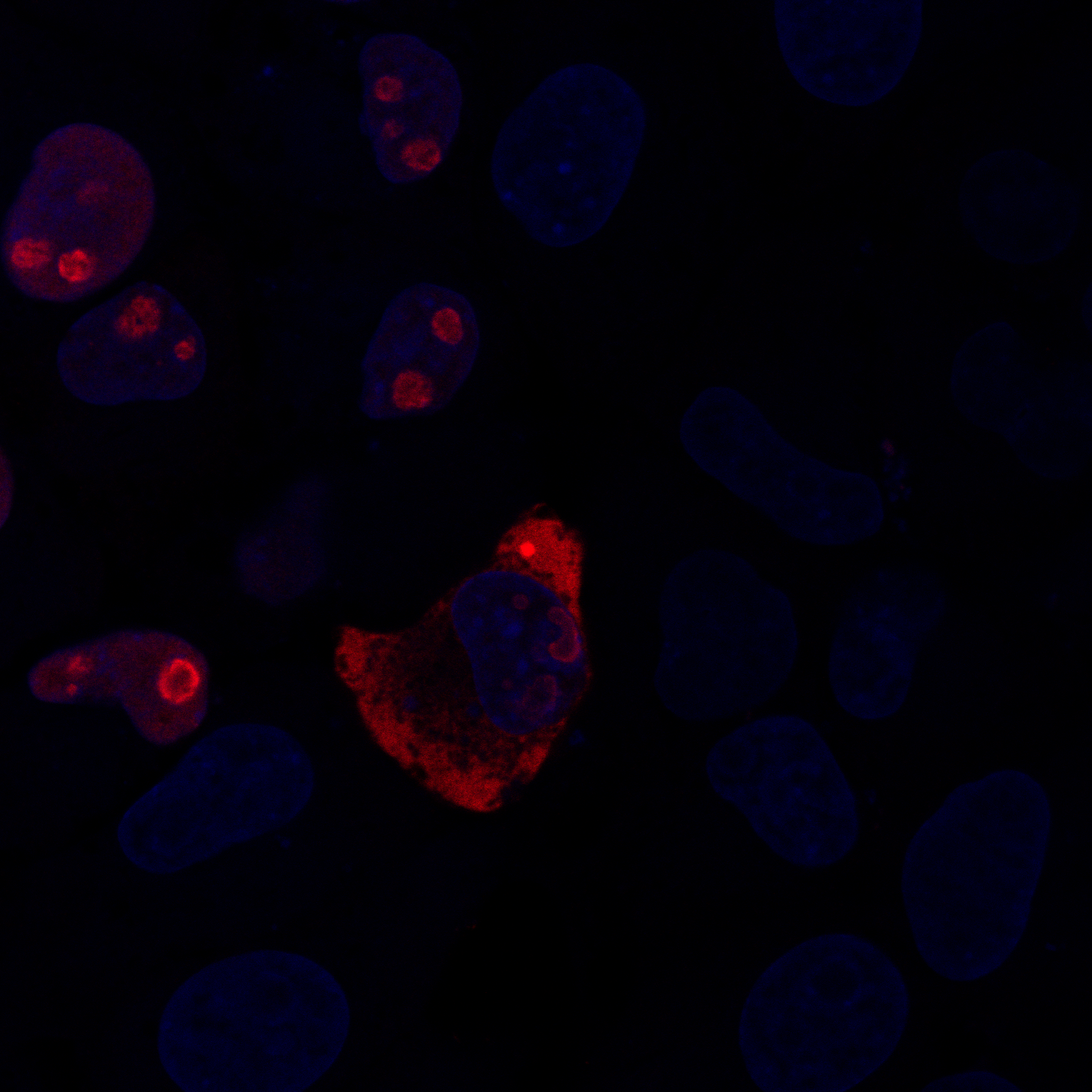

Supplement: S2 Data — (ZIP) [file ppat.1012014.s009.zip › A/A-2/siERK+rAd-Blank Merge.tif]

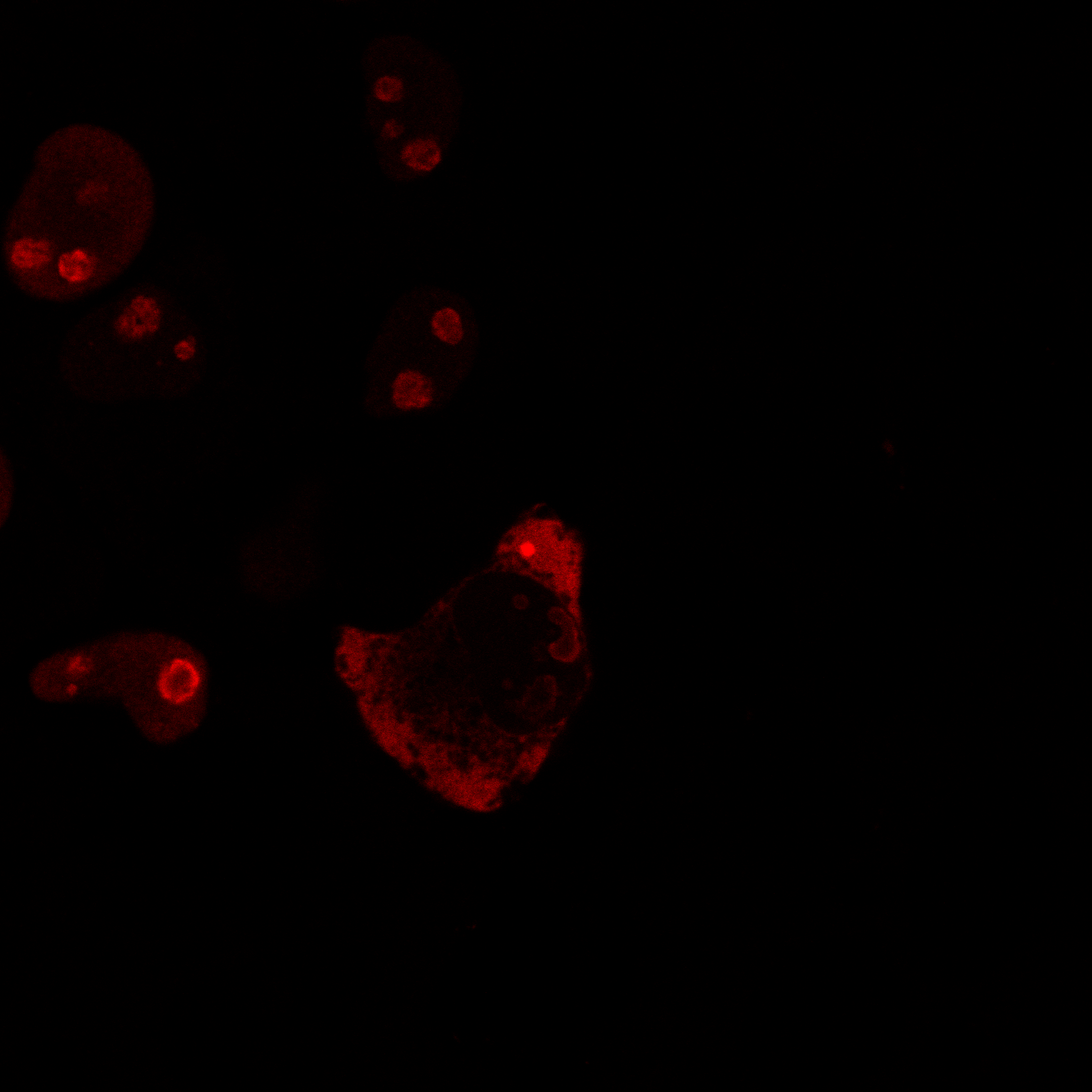

Supplement: S2 Data — (ZIP) [file ppat.1012014.s009.zip › A/A-2/siERK+rAd-Blank NPM1.tif]

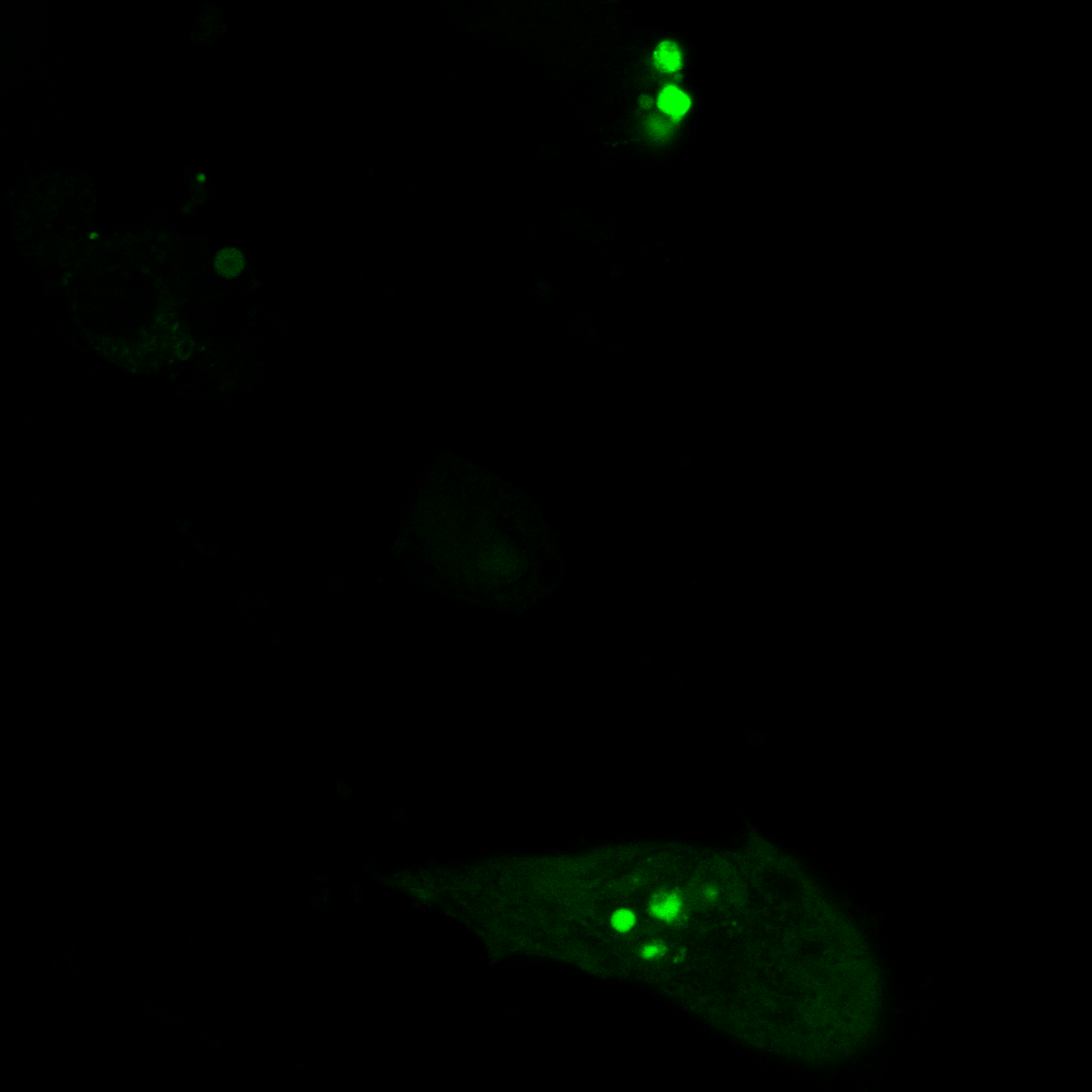

Supplement: S2 Data — (ZIP) [file ppat.1012014.s009.zip › A/A-2/siERK+rAd-Cap Cap.tif]

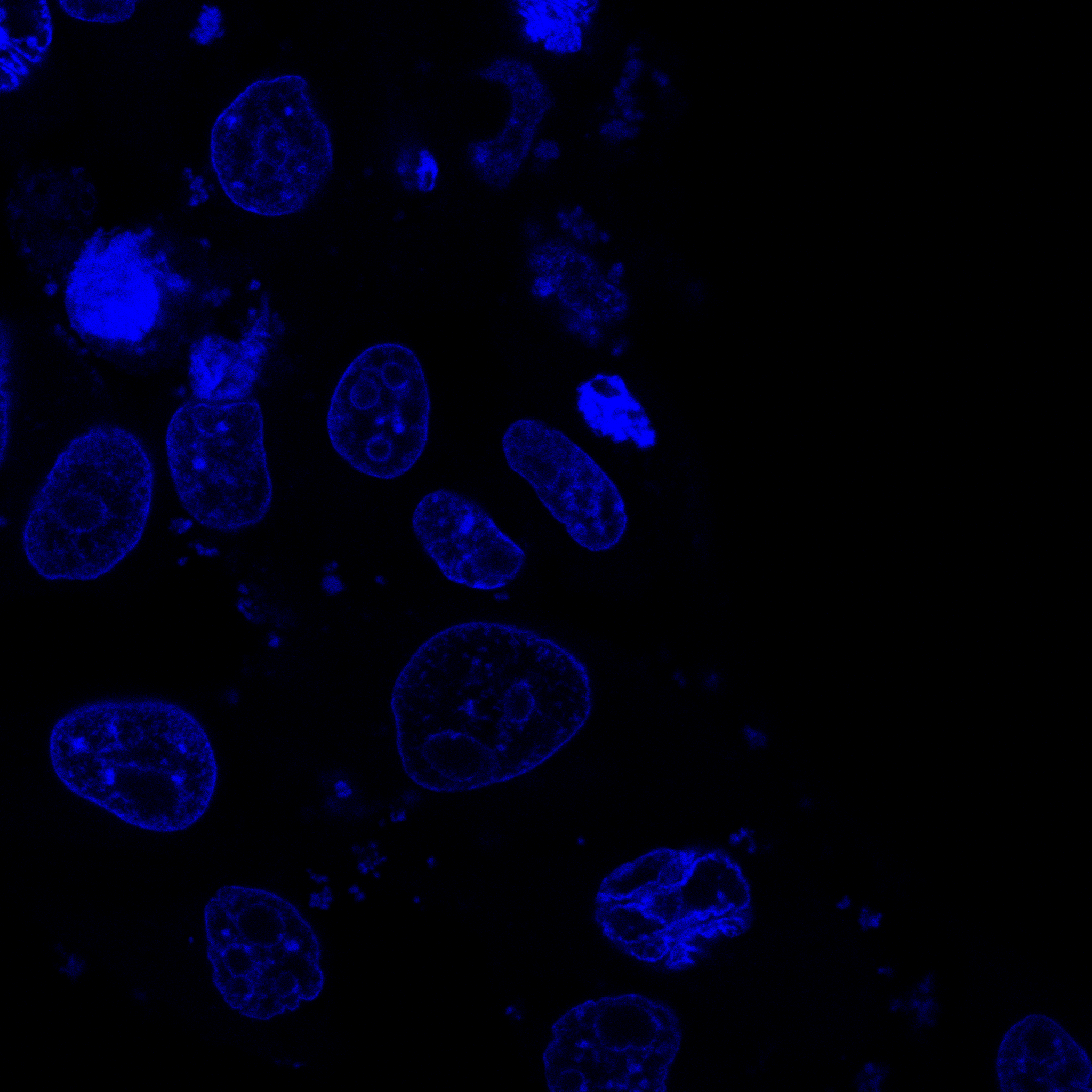

Supplement: S2 Data — (ZIP) [file ppat.1012014.s009.zip › A/A-2/siERK+rAd-Cap DAPI.tif]

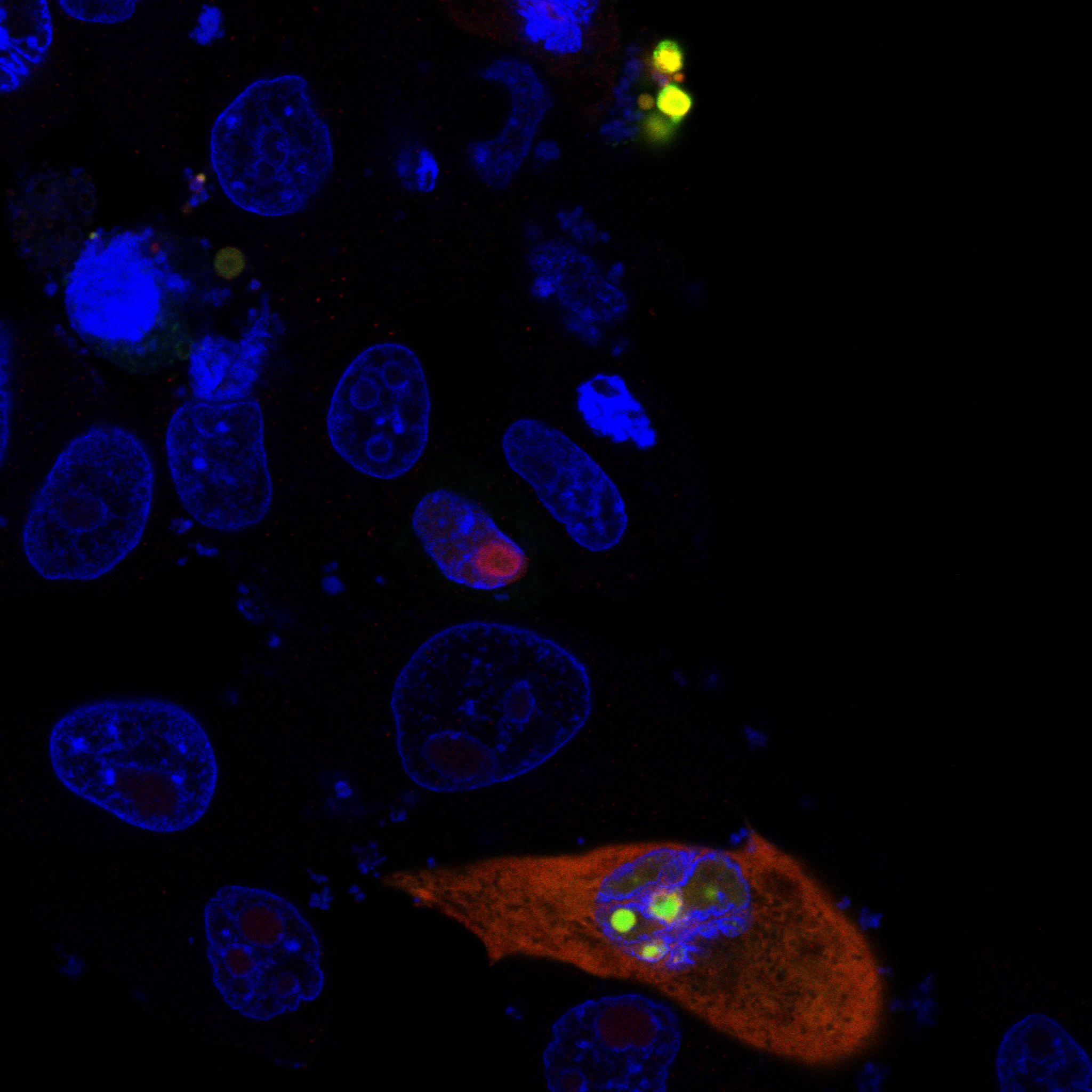

Supplement: S2 Data — (ZIP) [file ppat.1012014.s009.zip › A/A-2/siERK+rAd-Cap Merge.tif]

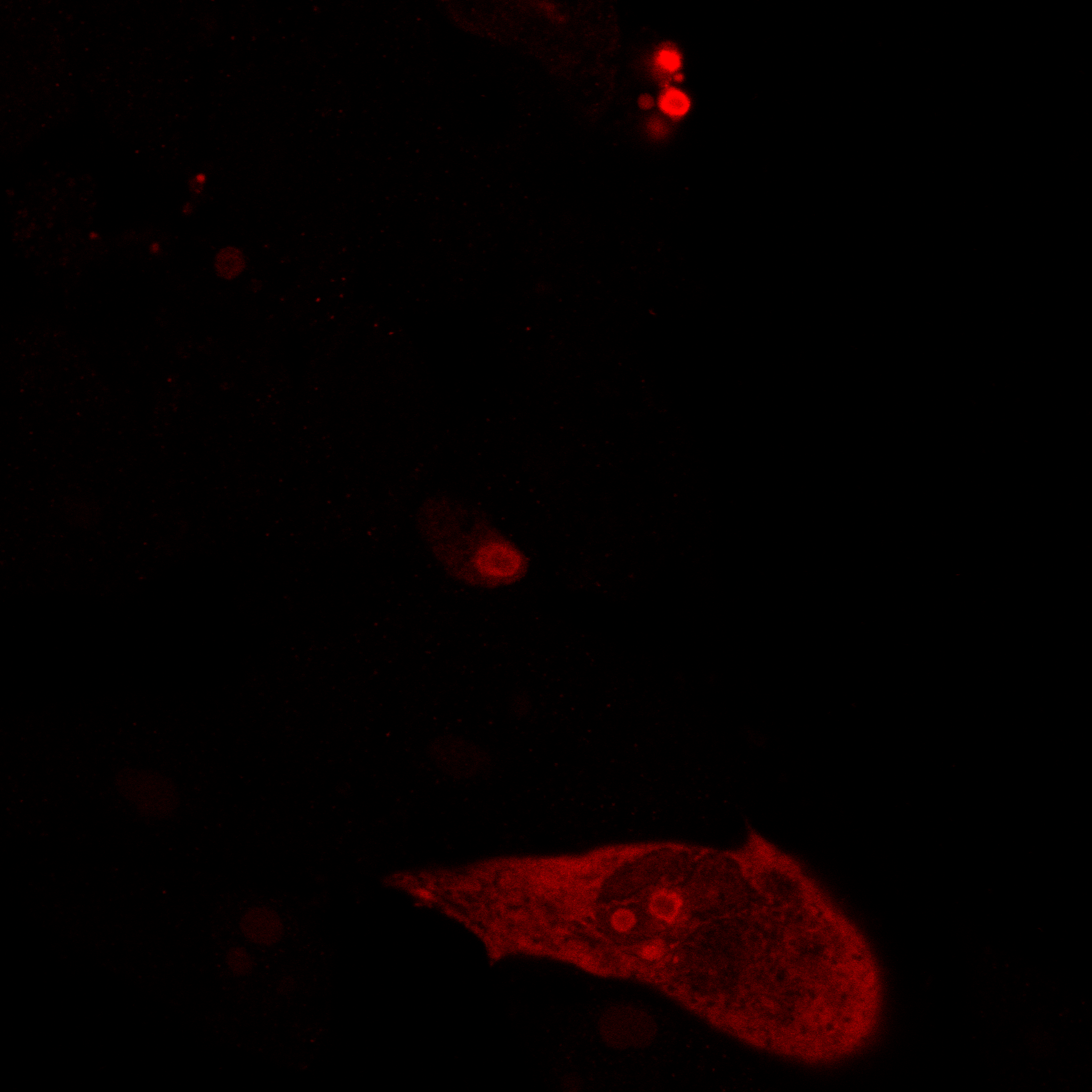

Supplement: S2 Data — (ZIP) [file ppat.1012014.s009.zip › A/A-2/siERK+rAd-Cap NPM1.tif]

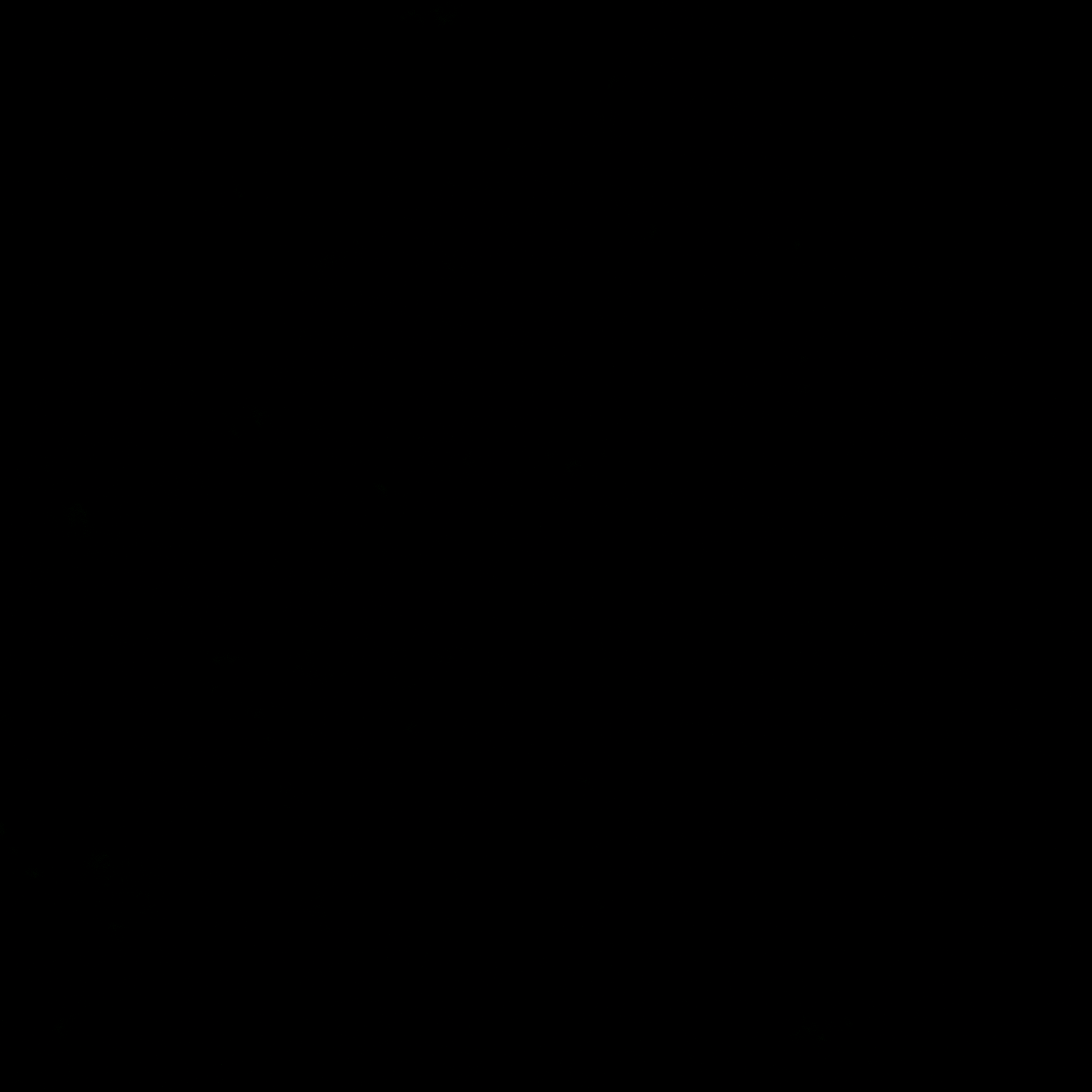

Supplement: S2 Data — (ZIP) [file ppat.1012014.s009.zip › A/A-2/siNC+rAd-Blank Cap.tif]

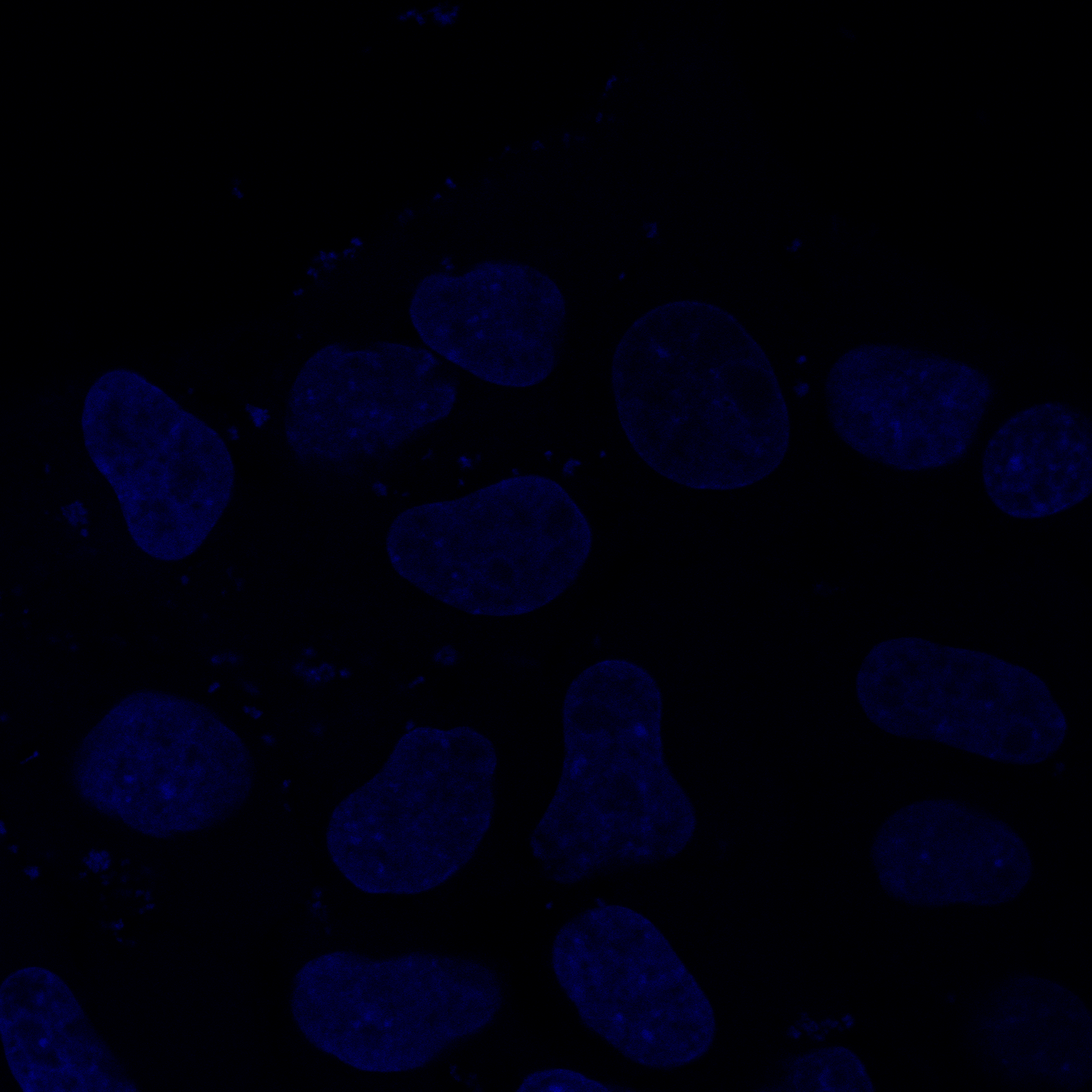

Supplement: S2 Data — (ZIP) [file ppat.1012014.s009.zip › A/A-2/siNC+rAd-Blank DAPI.tif]

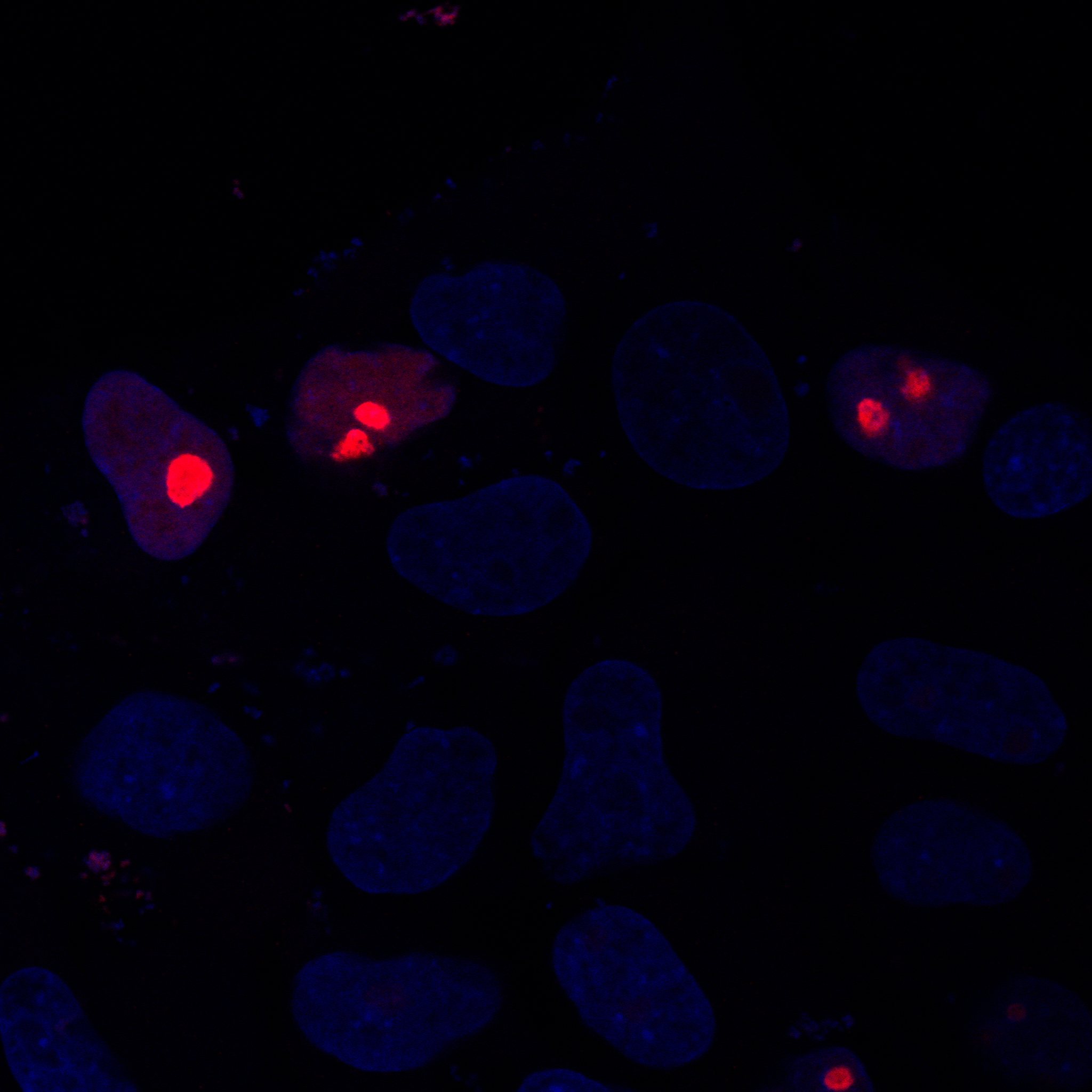

Supplement: S2 Data — (ZIP) [file ppat.1012014.s009.zip › A/A-2/siNC+rAd-Blank Merge.tif]

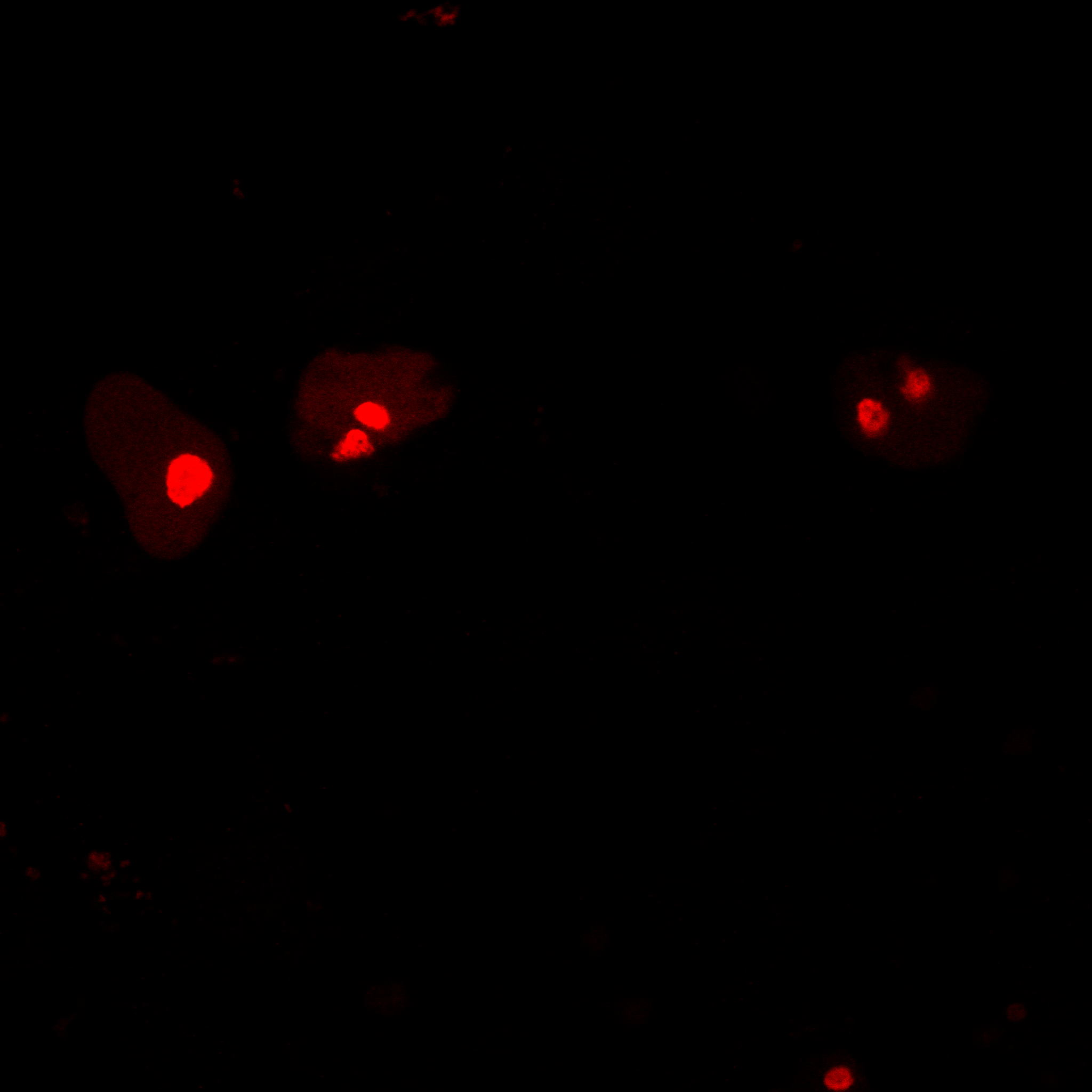

Supplement: S2 Data — (ZIP) [file ppat.1012014.s009.zip › A/A-2/siNC+rAd-Blank NPM1.tif]

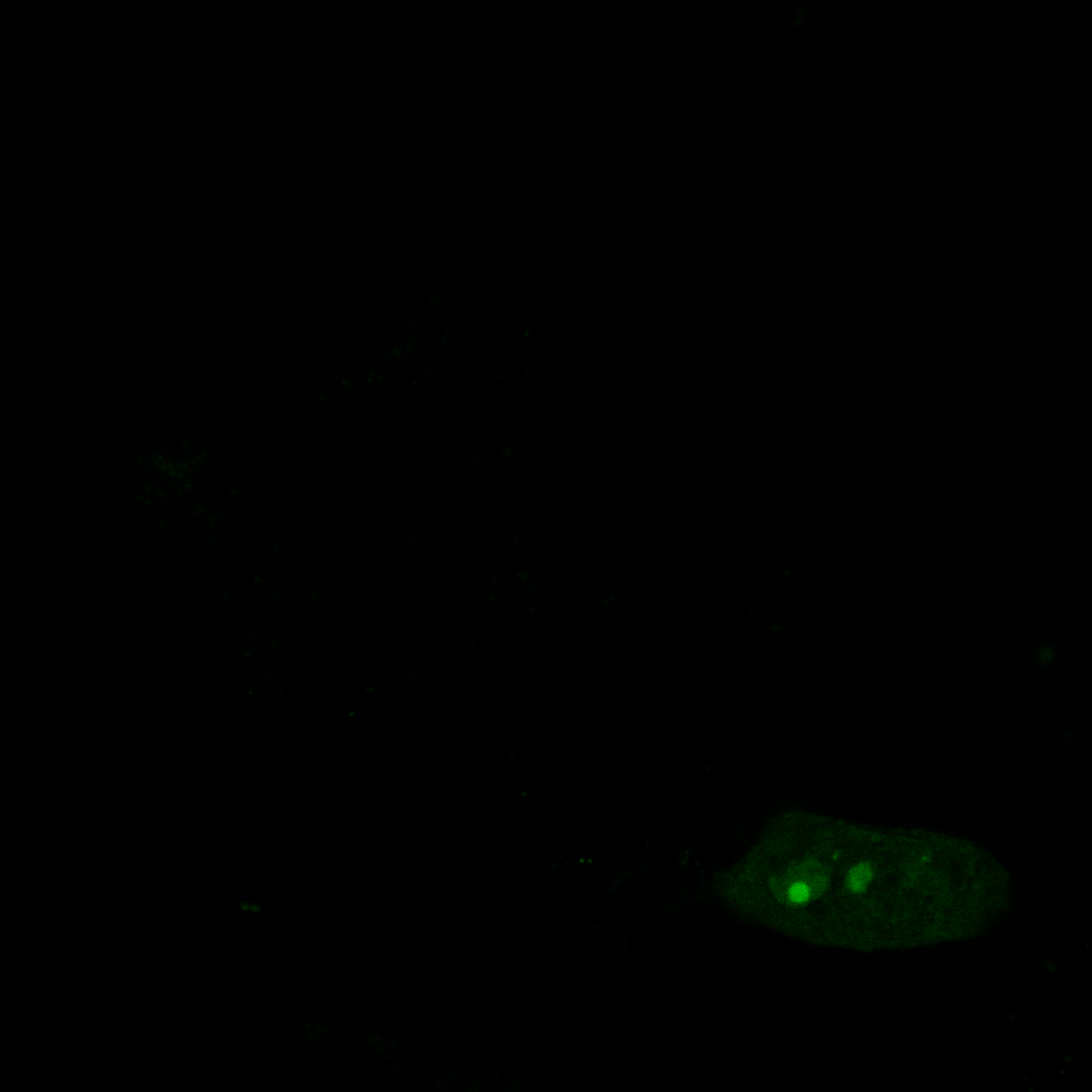

Supplement: S2 Data — (ZIP) [file ppat.1012014.s009.zip › A/A-2/siNC+rAd-Cap Cap.tif]

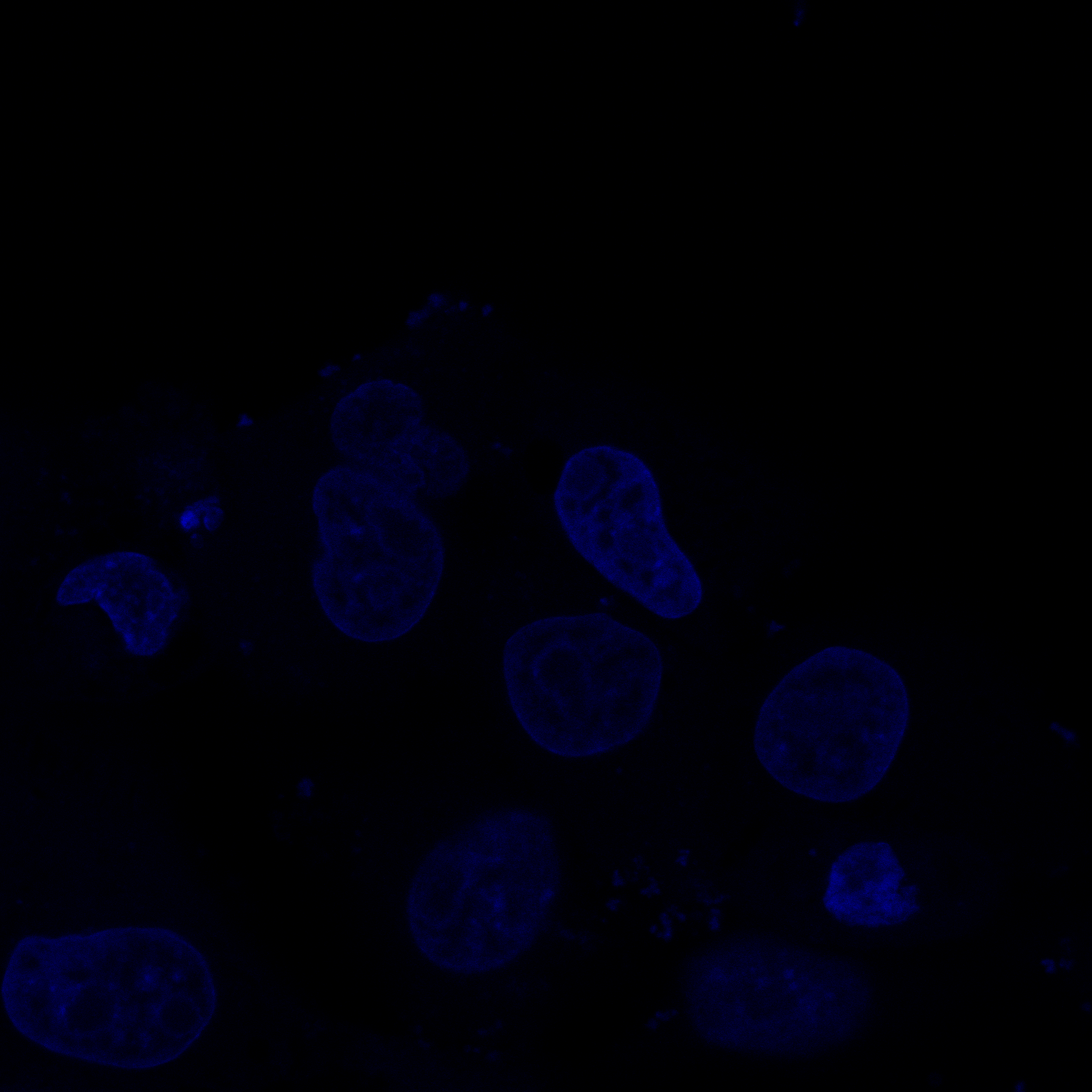

Supplement: S2 Data — (ZIP) [file ppat.1012014.s009.zip › A/A-2/siNC+rAd-Cap DAPI.tif]

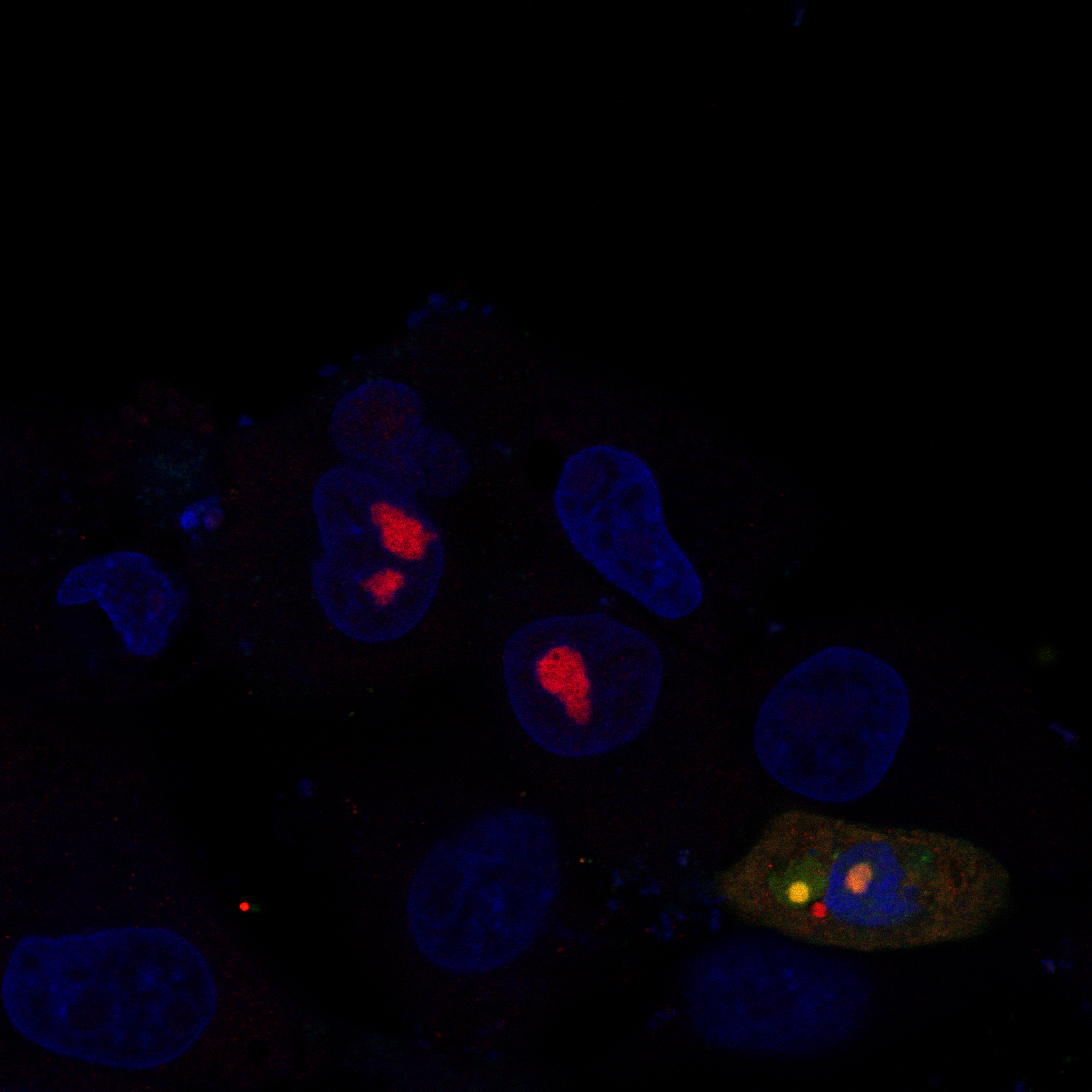

Supplement: S2 Data — (ZIP) [file ppat.1012014.s009.zip › A/A-2/siNC+rAd-Cap Merge.tif]

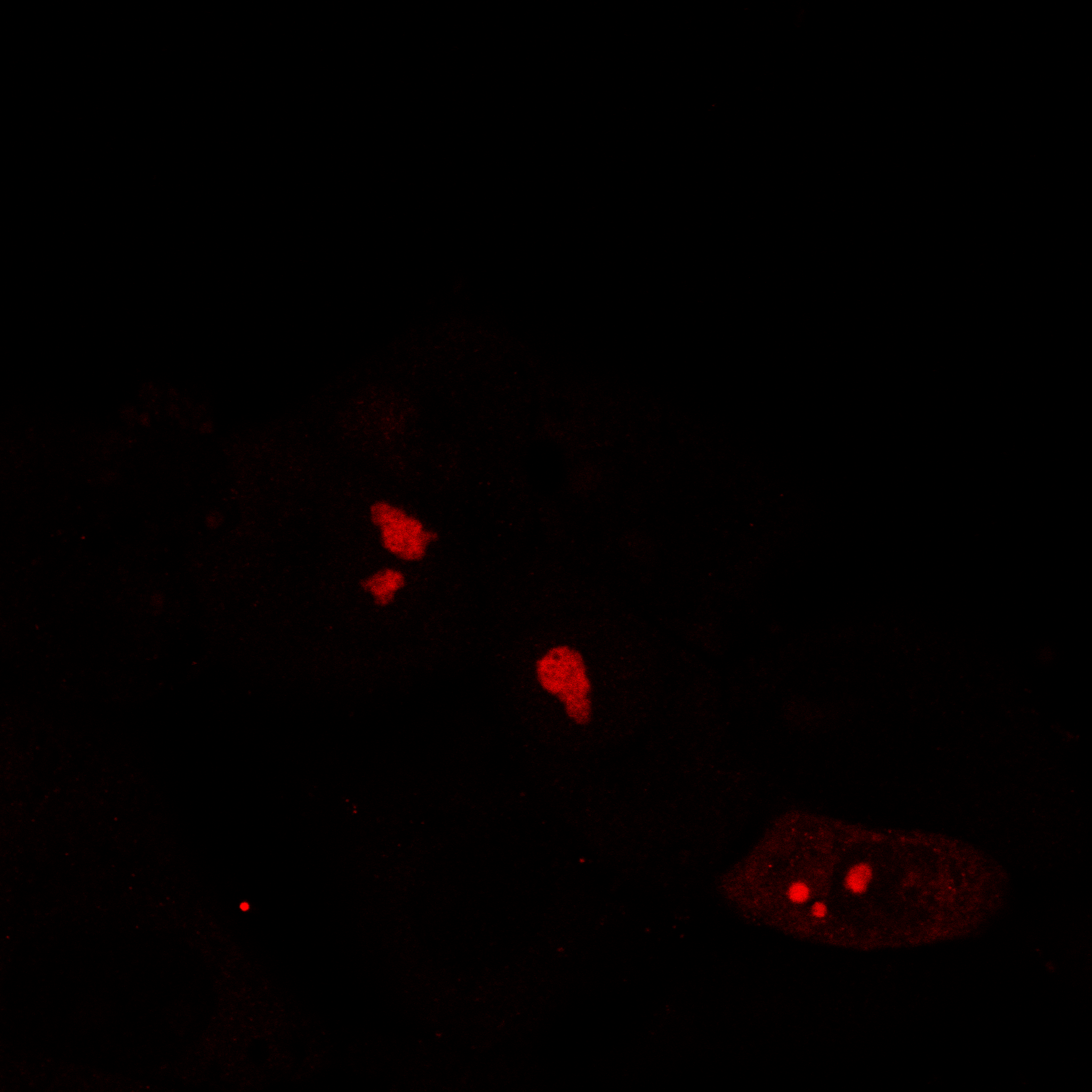

Supplement: S2 Data — (ZIP) [file ppat.1012014.s009.zip › A/A-2/siNC+rAd-Cap NPM1.tif]

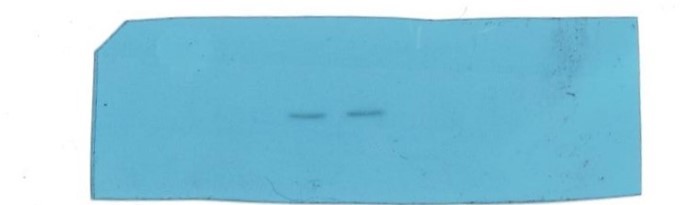

Supplement: S2 Data — (ZIP) [file ppat.1012014.s009.zip › B/B-1/Cytoplasm-Cap-1.jpg]

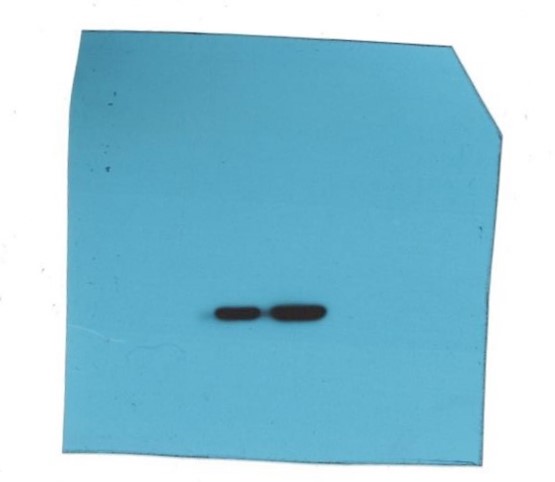

Supplement: S2 Data — (ZIP) [file ppat.1012014.s009.zip › B/B-1/Cytoplasm-NPM1-1.jpg]

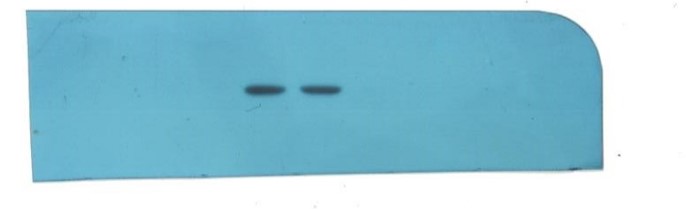

Supplement: S2 Data — (ZIP) [file ppat.1012014.s009.zip › B/B-1/Cytoplasm-β-actin-1.jpg]

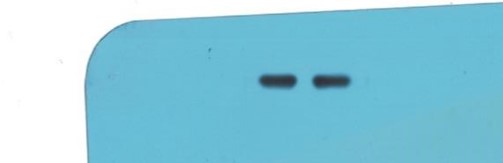

Supplement: S2 Data — (ZIP) [file ppat.1012014.s009.zip › B/B-1/Nuclear-Cap-1.jpg]

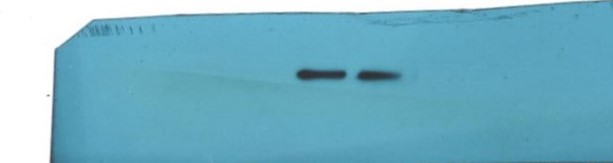

Supplement: S2 Data — (ZIP) [file ppat.1012014.s009.zip › B/B-1/Nuclear-LaminB-1.jpg]

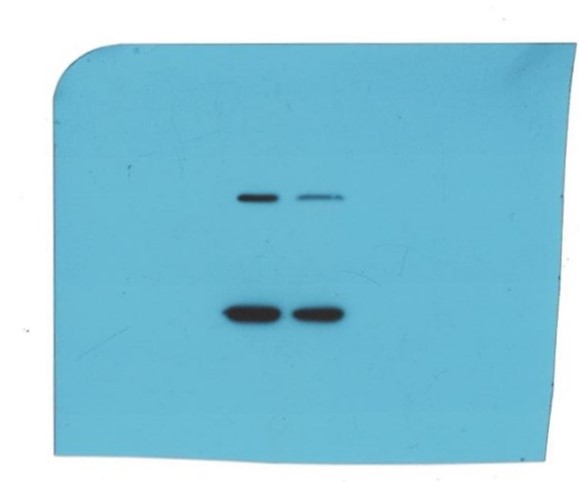

Supplement: S2 Data — (ZIP) [file ppat.1012014.s009.zip › B/B-1/Nuclear-NPM1-1.jpg]

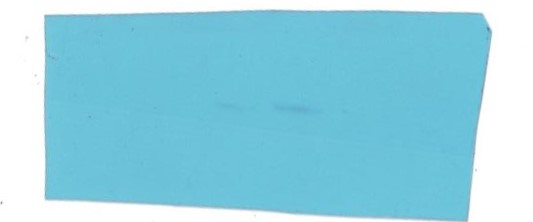

Supplement: S2 Data — (ZIP) [file ppat.1012014.s009.zip › B/B-2/Cap-Cytoplasm.jpg]

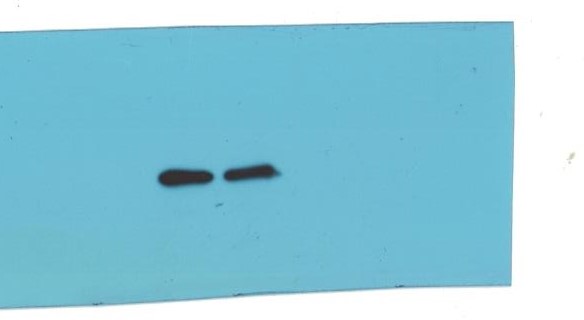

Supplement: S2 Data — (ZIP) [file ppat.1012014.s009.zip › B/B-2/Cap-Nuclear.jpg]

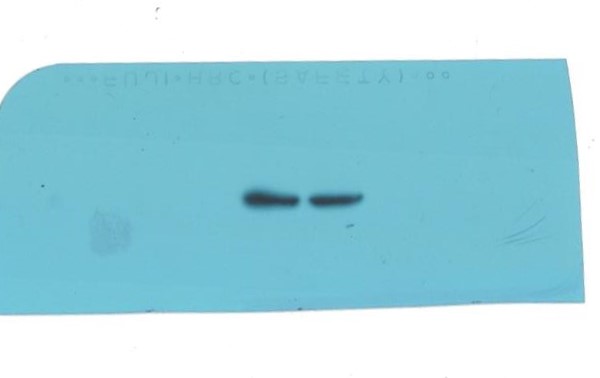

Supplement: S2 Data — (ZIP) [file ppat.1012014.s009.zip › B/B-2/F7-B-LaminB.jpg]

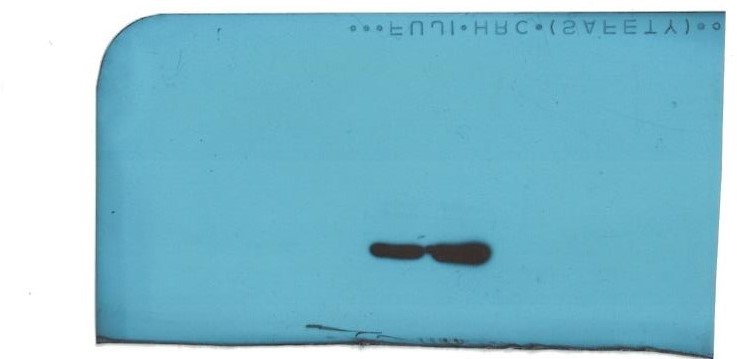

Supplement: S2 Data — (ZIP) [file ppat.1012014.s009.zip › B/B-2/NPM1-Cytoplasm.jpg]

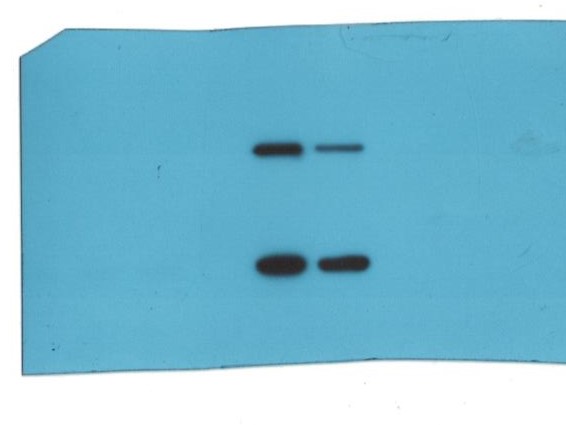

Supplement: S2 Data — (ZIP) [file ppat.1012014.s009.zip › B/B-2/NPM1-Nuclear.jpg]

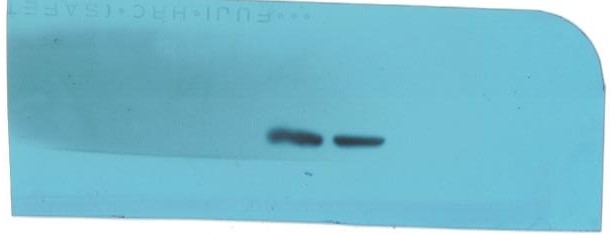

Supplement: S2 Data — (ZIP) [file ppat.1012014.s009.zip › B/B-2/β-actin.jpg]

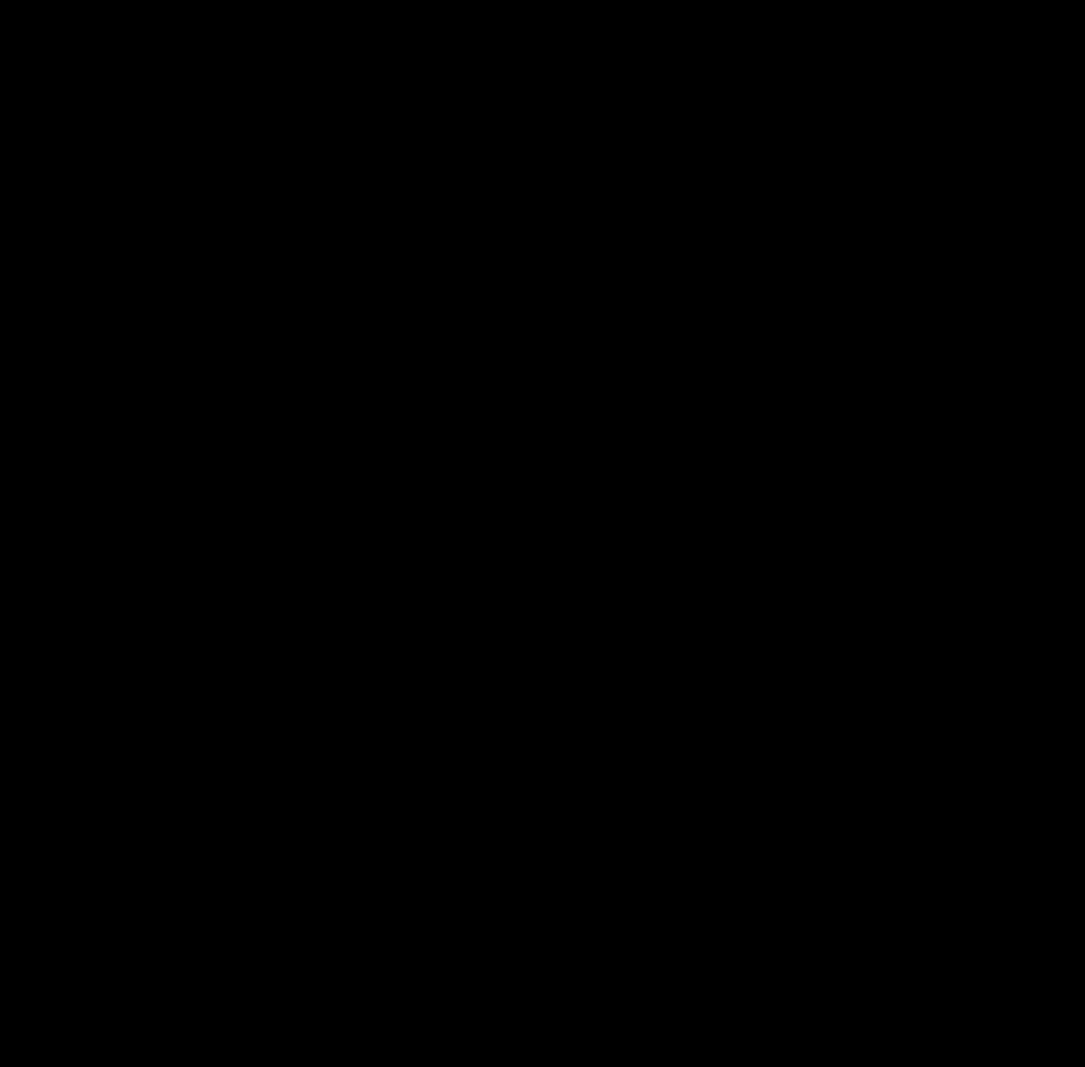

Supplement: S2 Data — (ZIP) [file ppat.1012014.s009.zip › C/C-1/siERK+Mock Cap.tif]

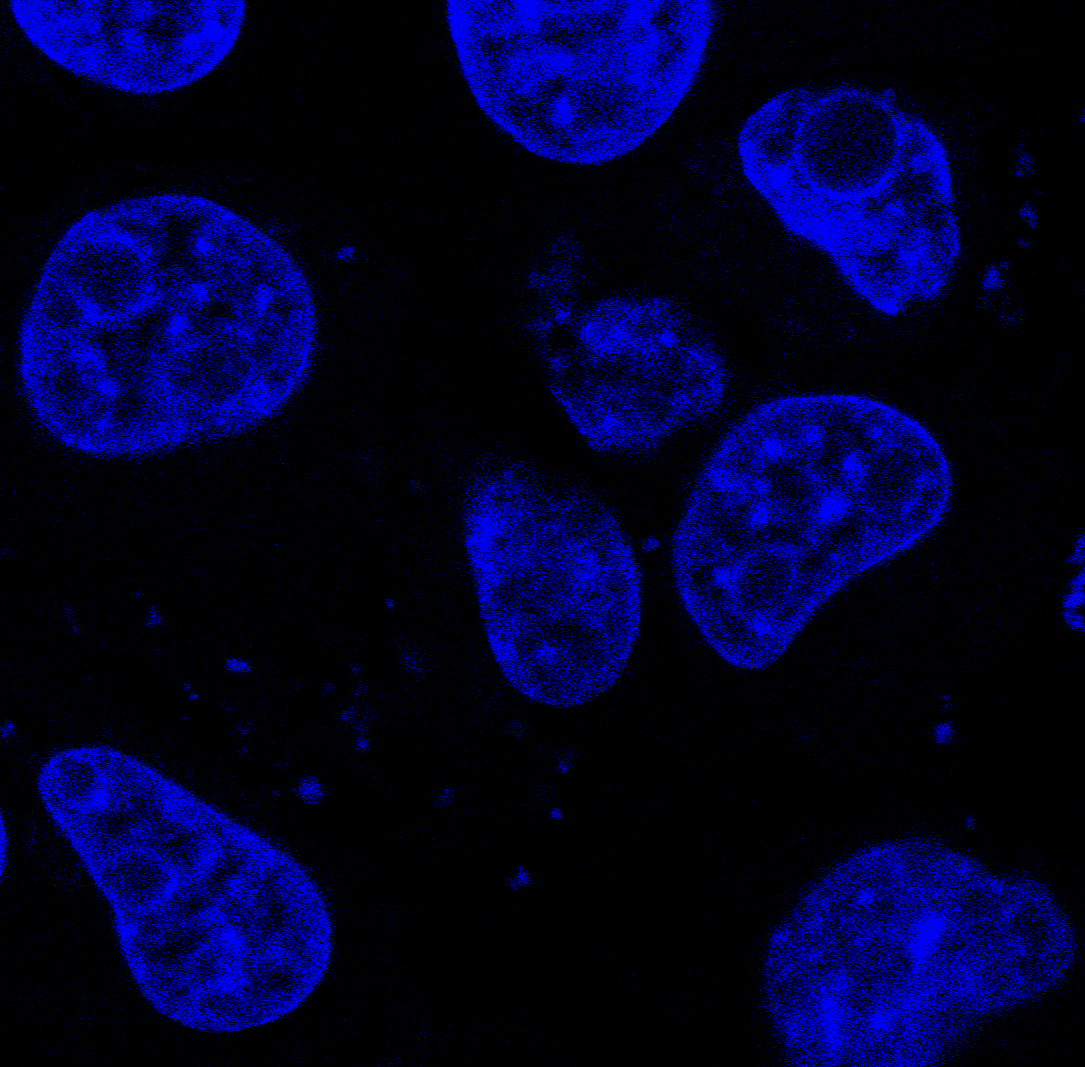

Supplement: S2 Data — (ZIP) [file ppat.1012014.s009.zip › C/C-1/siERK+Mock DAPI.tif]

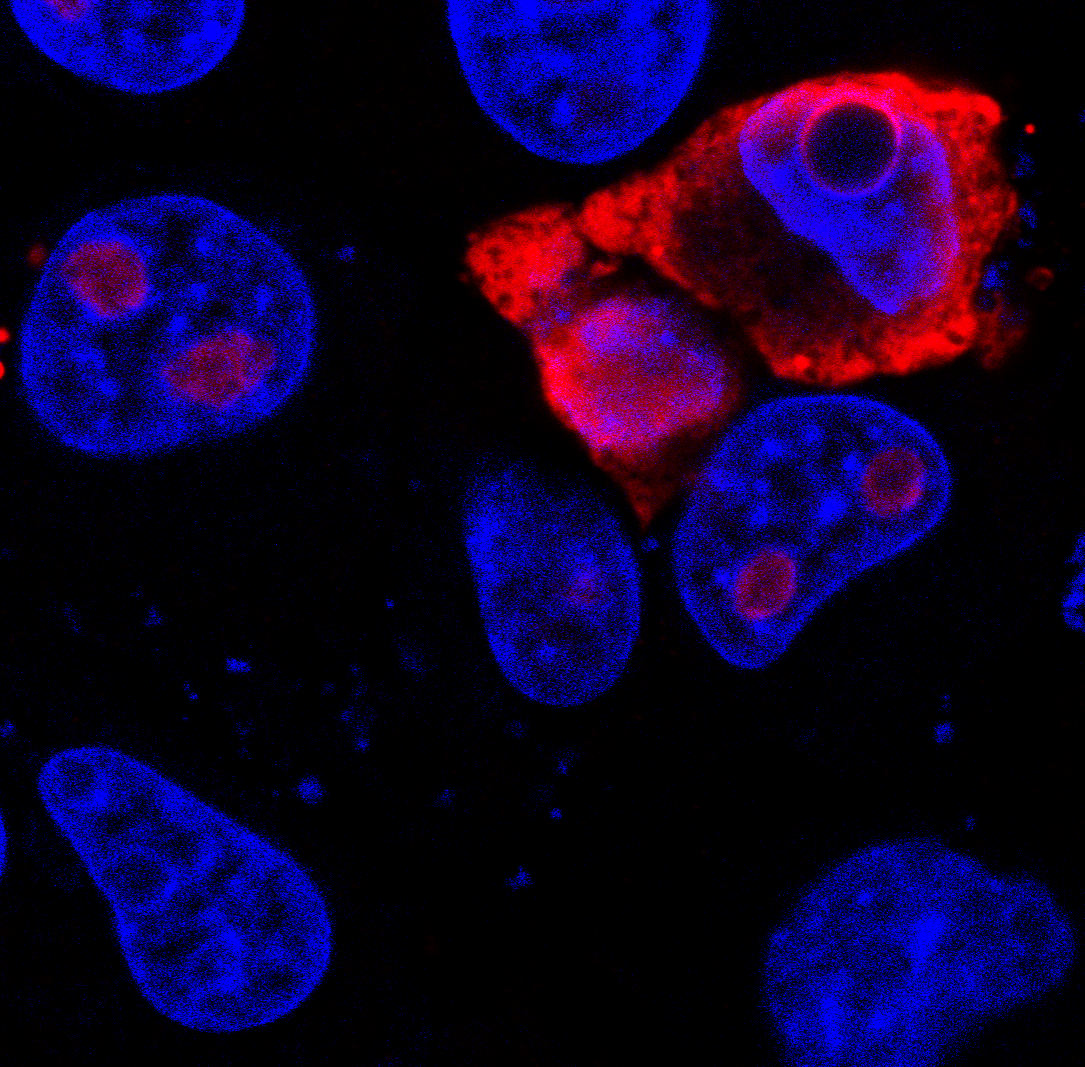

Supplement: S2 Data — (ZIP) [file ppat.1012014.s009.zip › C/C-1/siERK+Mock Merge.tif]

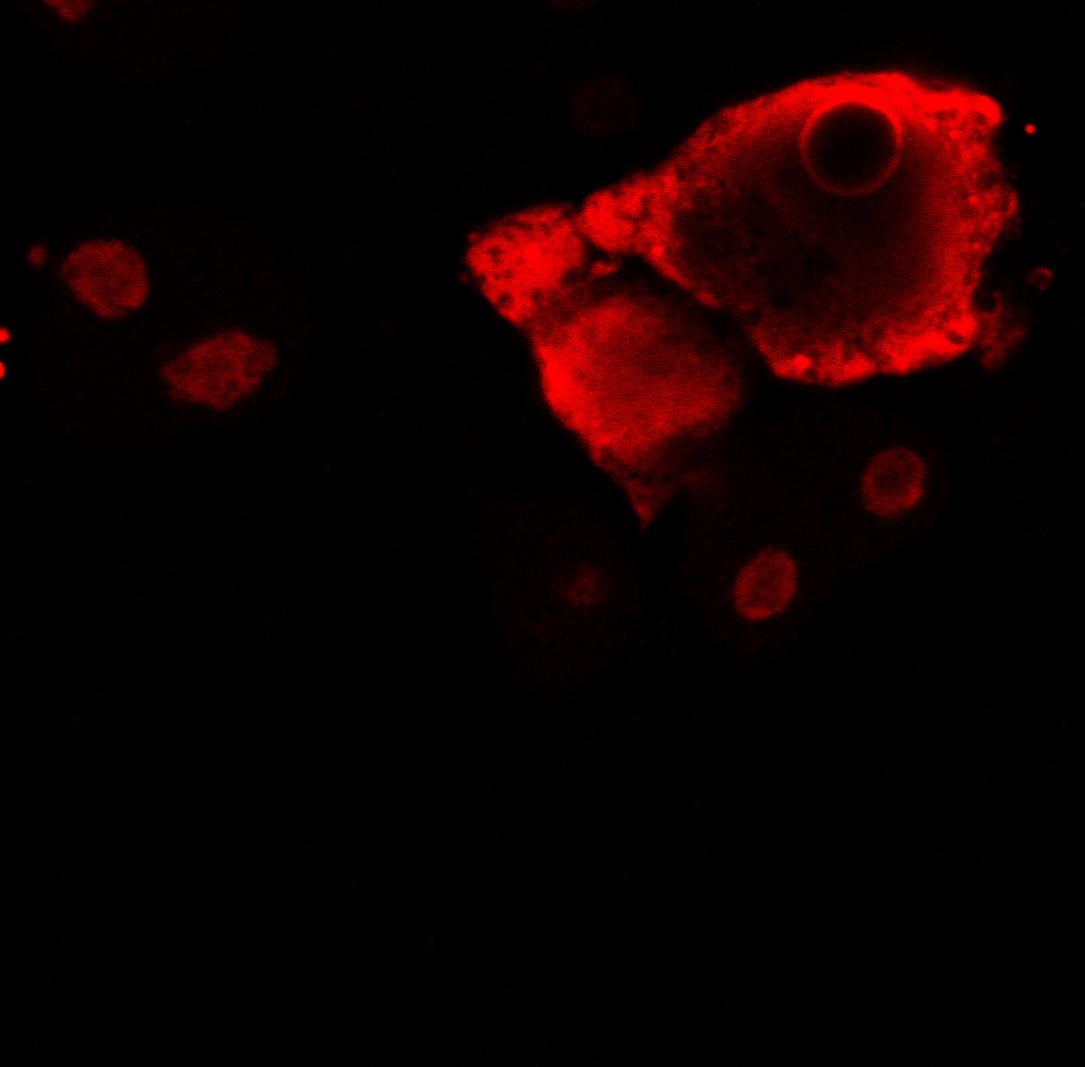

Supplement: S2 Data — (ZIP) [file ppat.1012014.s009.zip › C/C-1/siERK+Mock NPM1.tif]

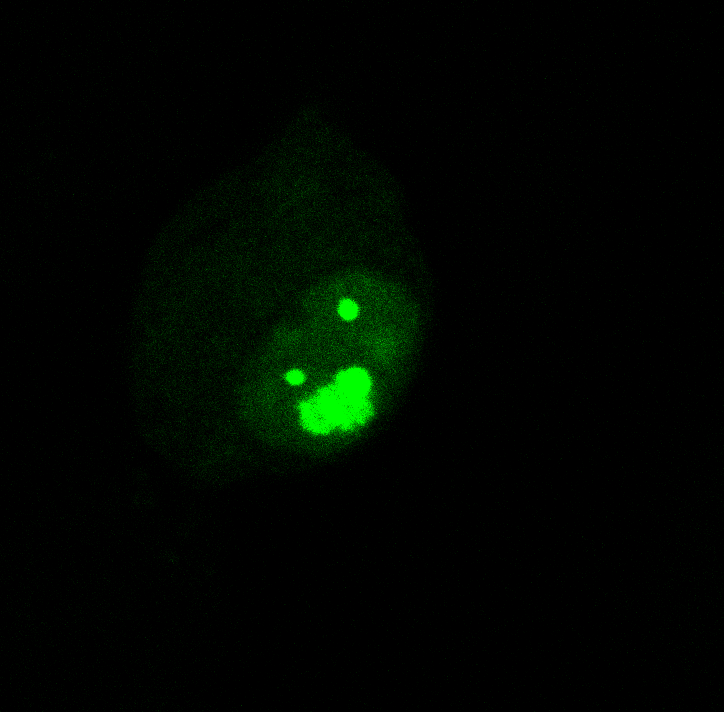

Supplement: S2 Data — (ZIP) [file ppat.1012014.s009.zip › C/C-1/siERK+PCV2 Cap.tif]

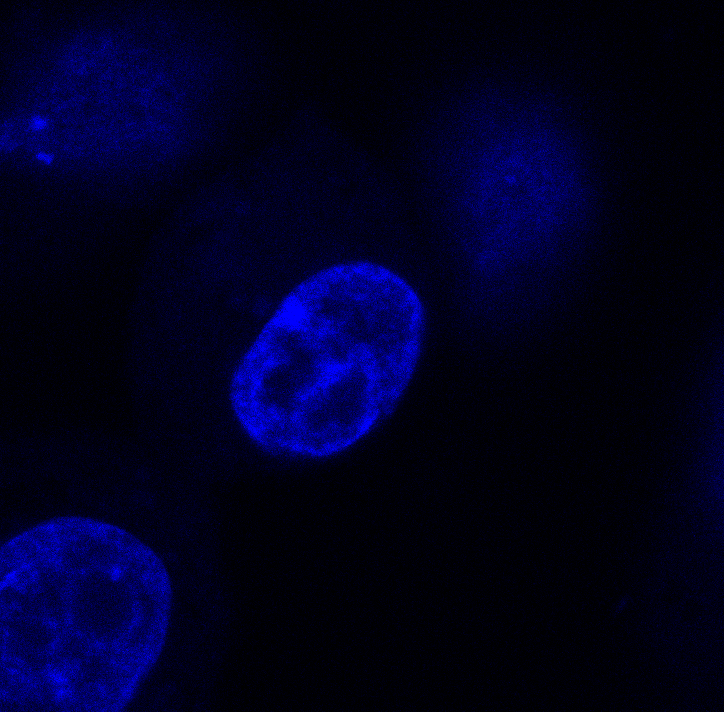

Supplement: S2 Data — (ZIP) [file ppat.1012014.s009.zip › C/C-1/siERK+PCV2 DAPI.tif]

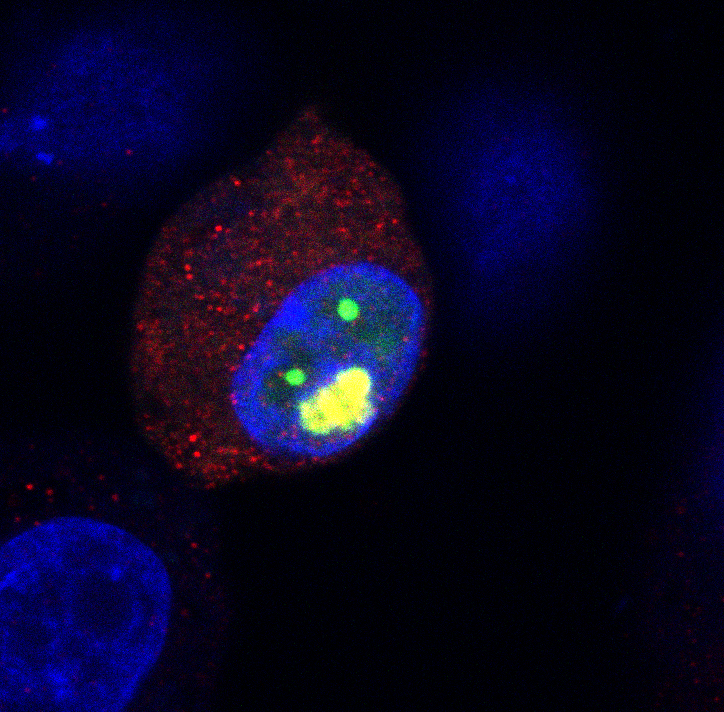

Supplement: S2 Data — (ZIP) [file ppat.1012014.s009.zip › C/C-1/siERK+PCV2 Merge.tif]

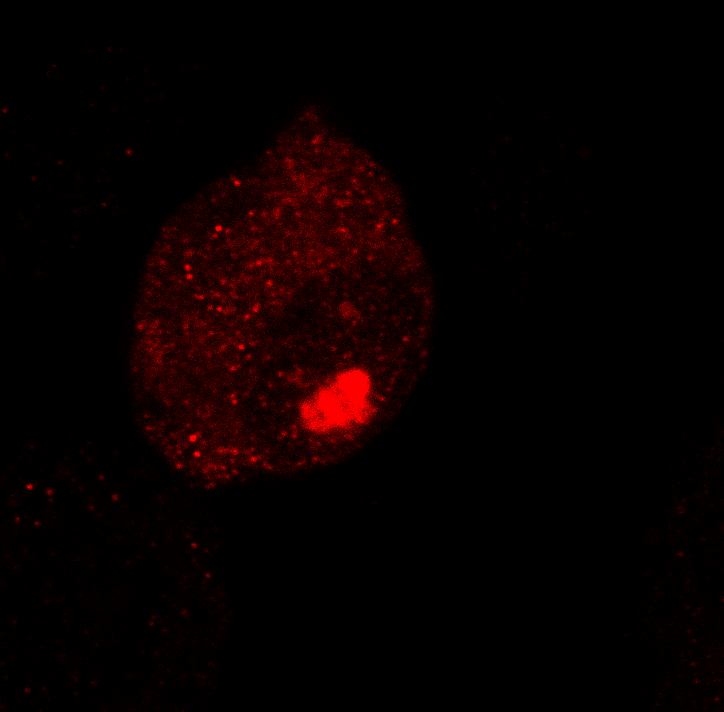

Supplement: S2 Data — (ZIP) [file ppat.1012014.s009.zip › C/C-1/siERK+PCV2 NPM1.tif]

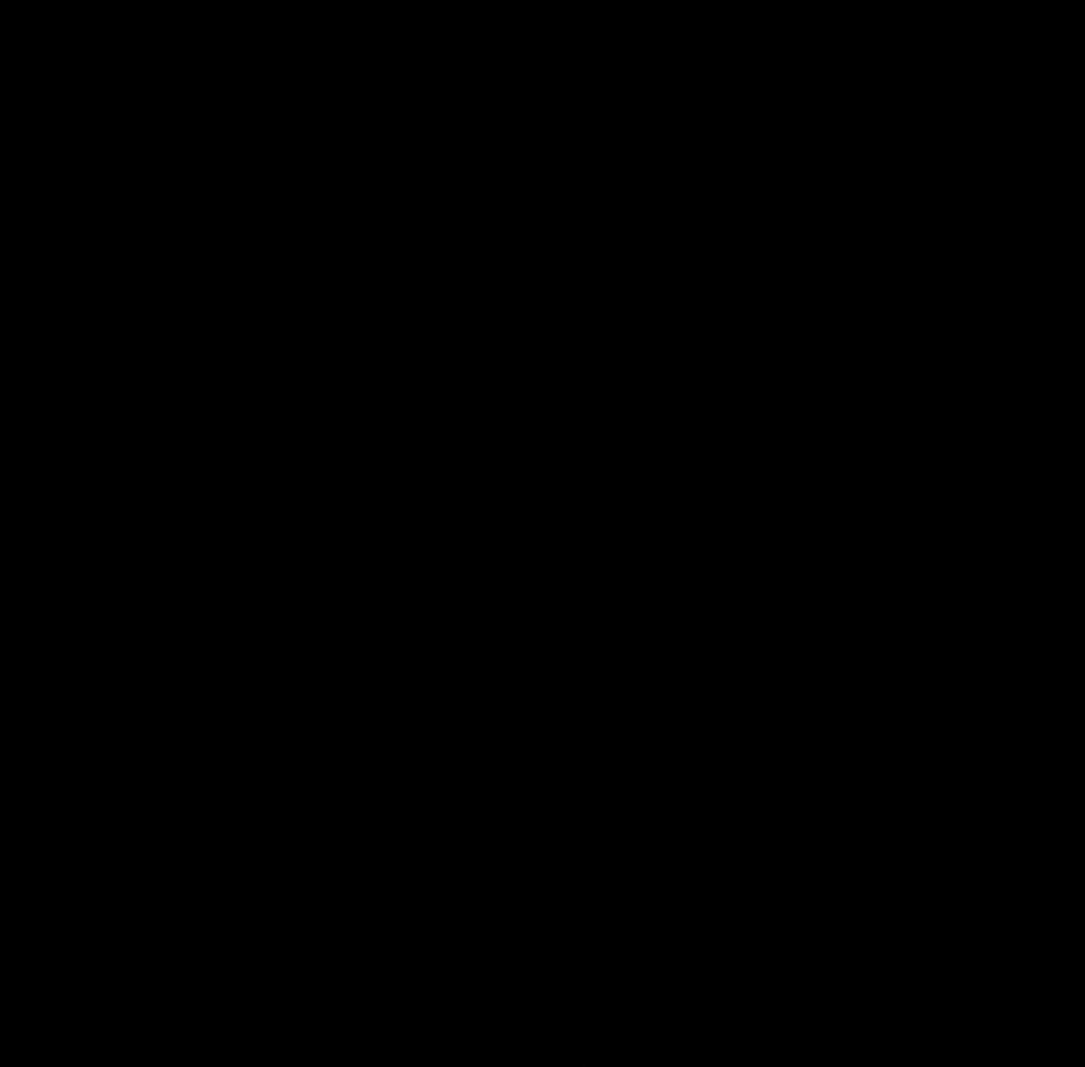

Supplement: S2 Data — (ZIP) [file ppat.1012014.s009.zip › C/C-1/siNC+Mock Cap.tif]

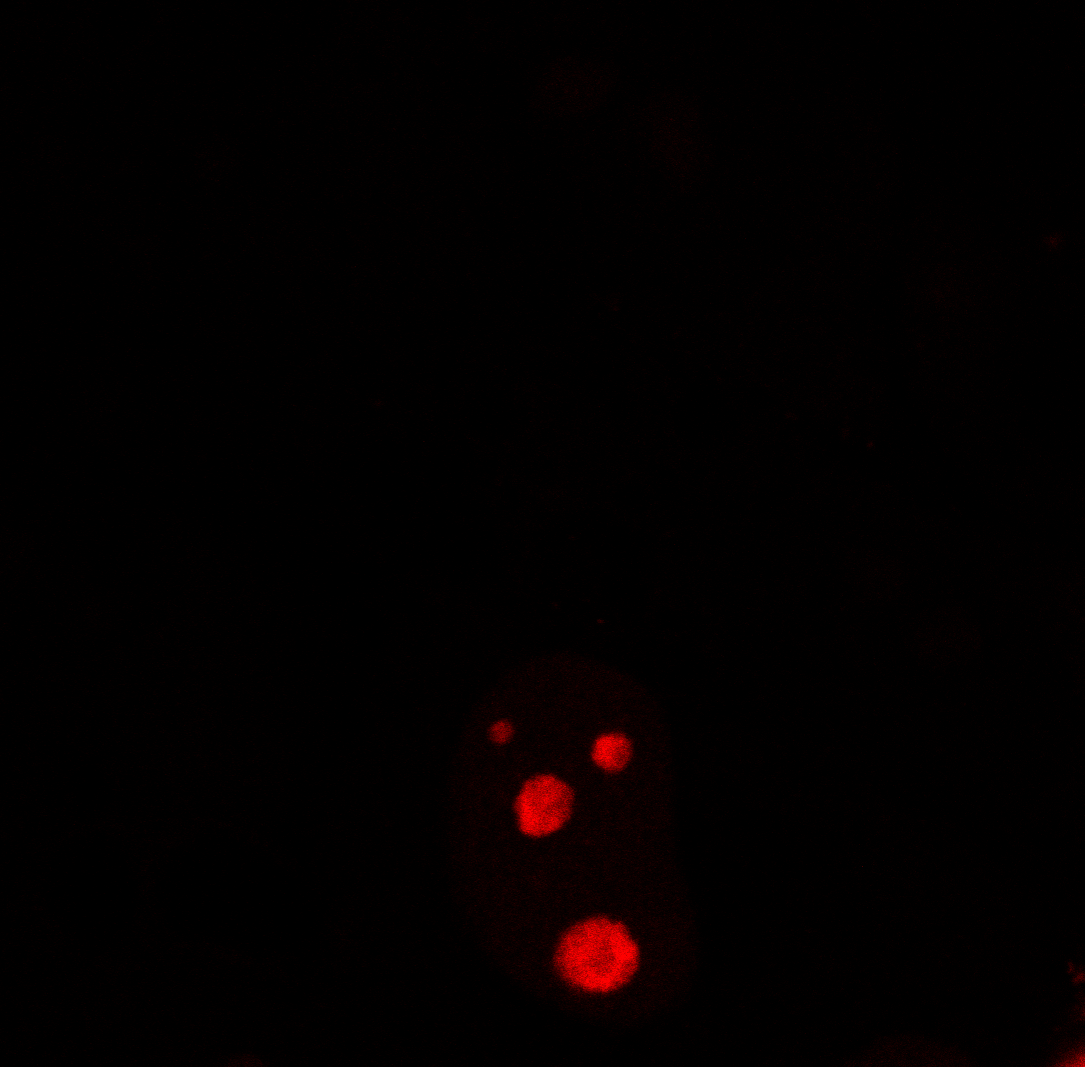

Supplement: S2 Data — (ZIP) [file ppat.1012014.s009.zip › C/C-1/siNC+Mock CapNPM1.tif]

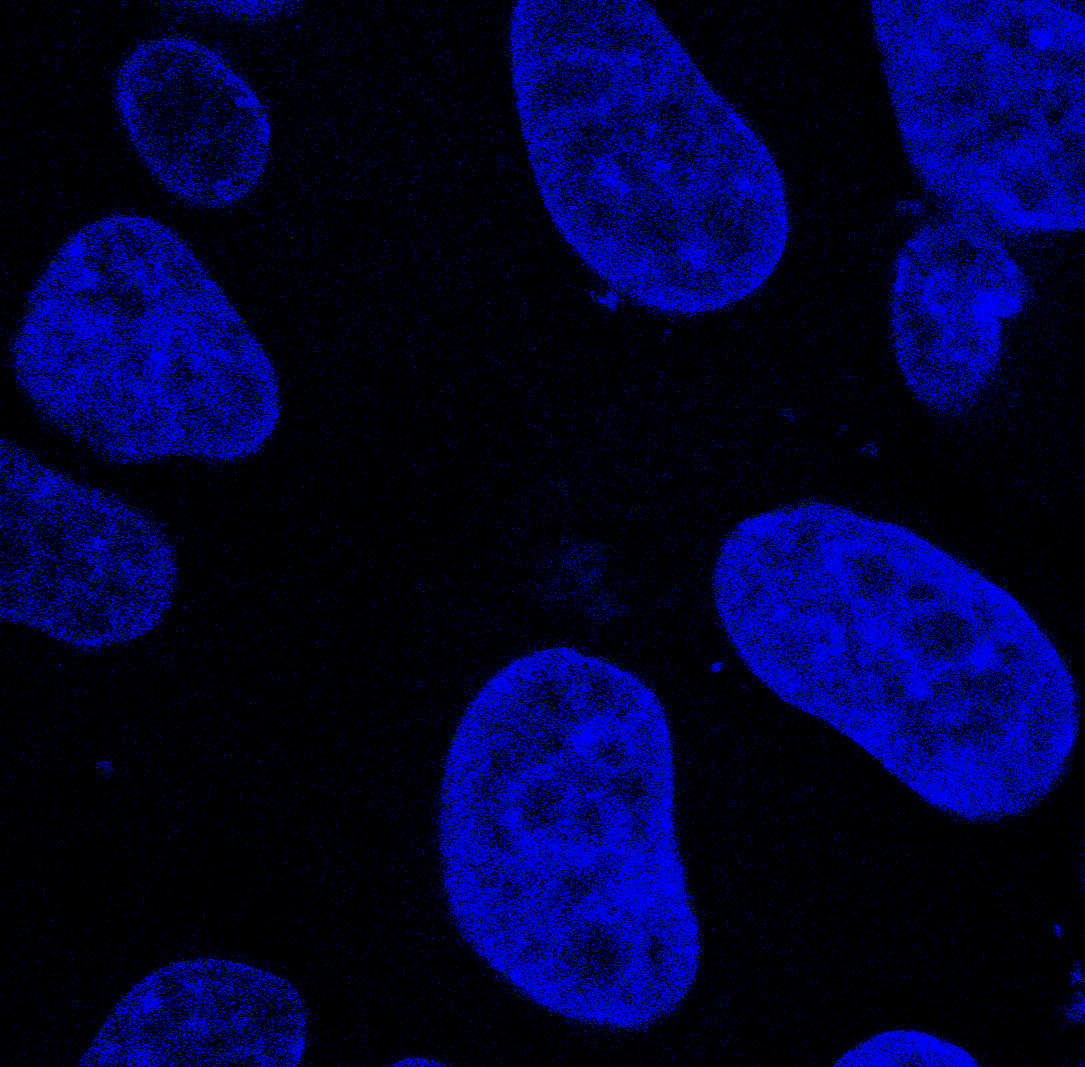

Supplement: S2 Data — (ZIP) [file ppat.1012014.s009.zip › C/C-1/siNC+Mock DAPI.tif]

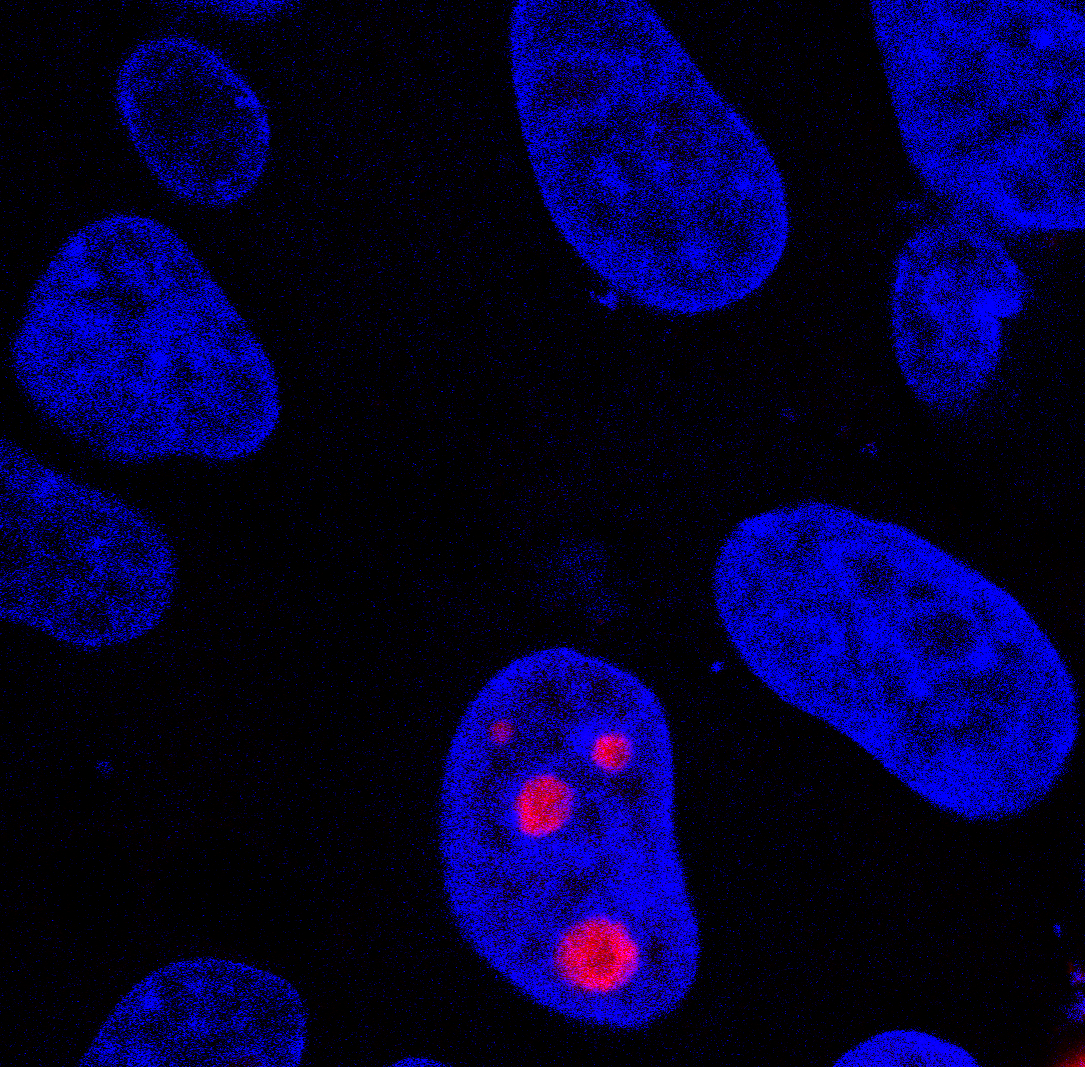

Supplement: S2 Data — (ZIP) [file ppat.1012014.s009.zip › C/C-1/siNC+Mock Merge.tif]

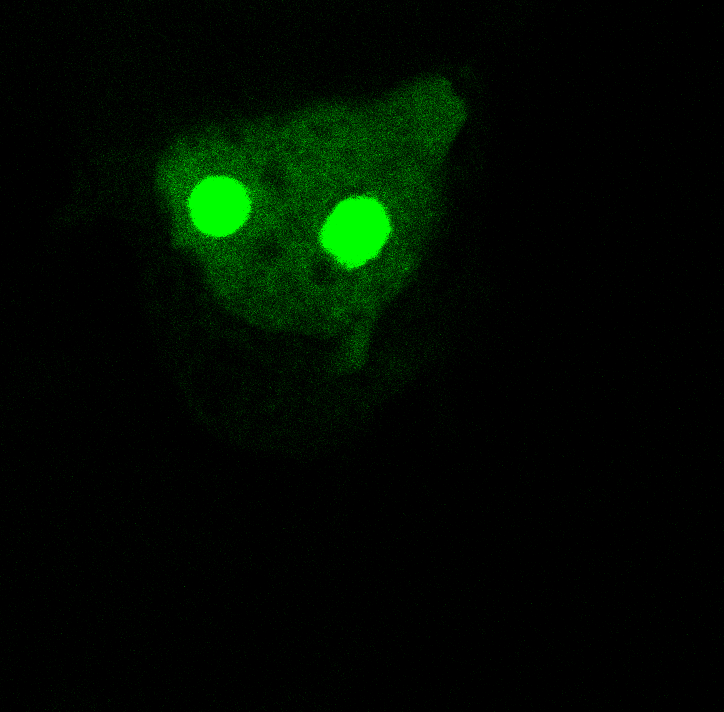

Supplement: S2 Data — (ZIP) [file ppat.1012014.s009.zip › C/C-1/siNC+PCV2 Cap.tif]

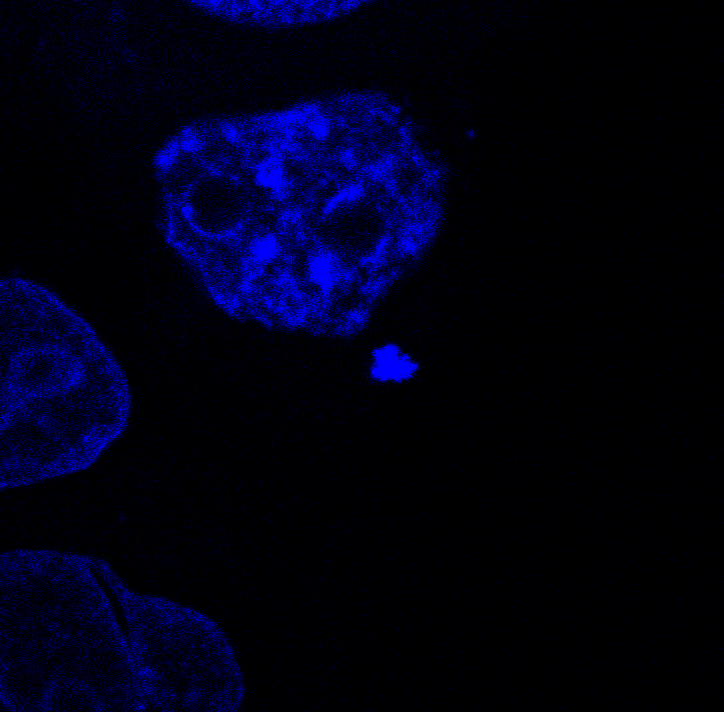

Supplement: S2 Data — (ZIP) [file ppat.1012014.s009.zip › C/C-1/siNC+PCV2 DAPI.tif]

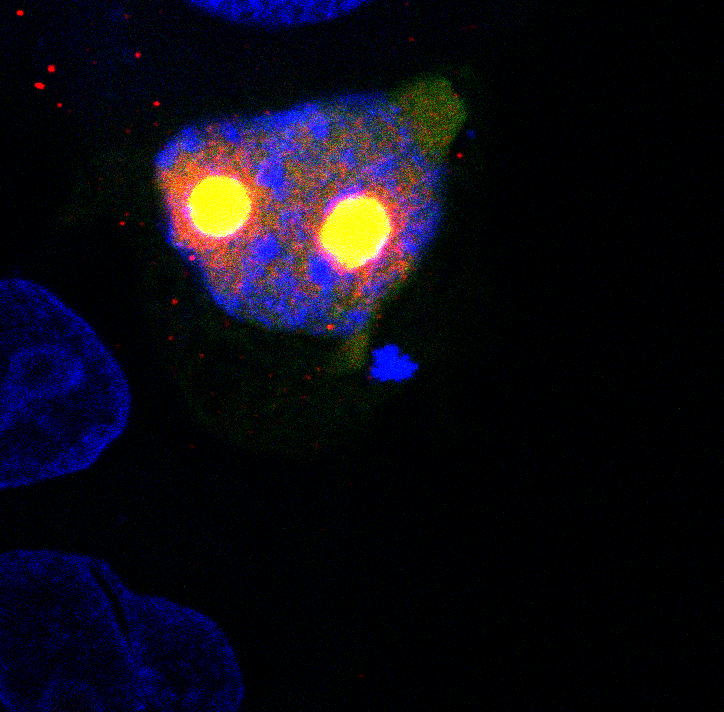

Supplement: S2 Data — (ZIP) [file ppat.1012014.s009.zip › C/C-1/siNC+PCV2 Merge.tif]

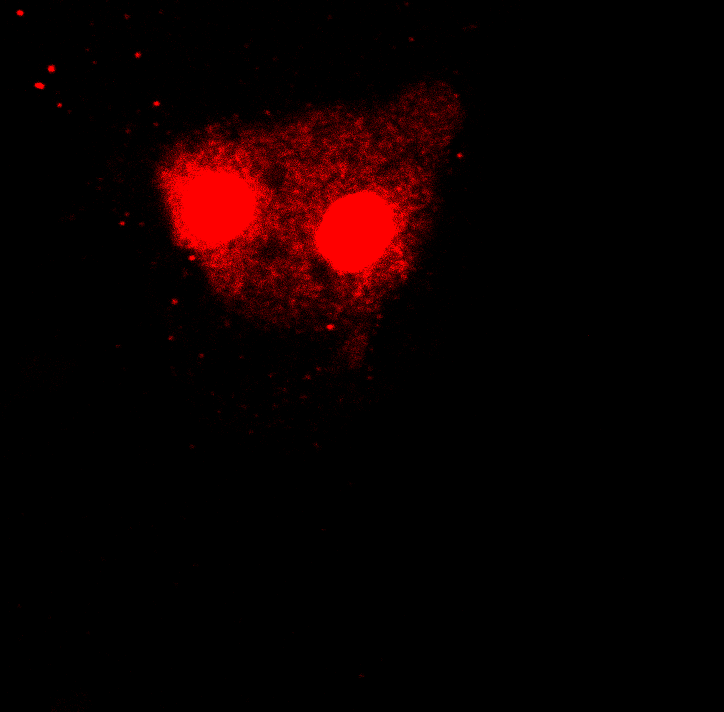

Supplement: S2 Data — (ZIP) [file ppat.1012014.s009.zip › C/C-1/siNC+PCV2 NPM1.tif]

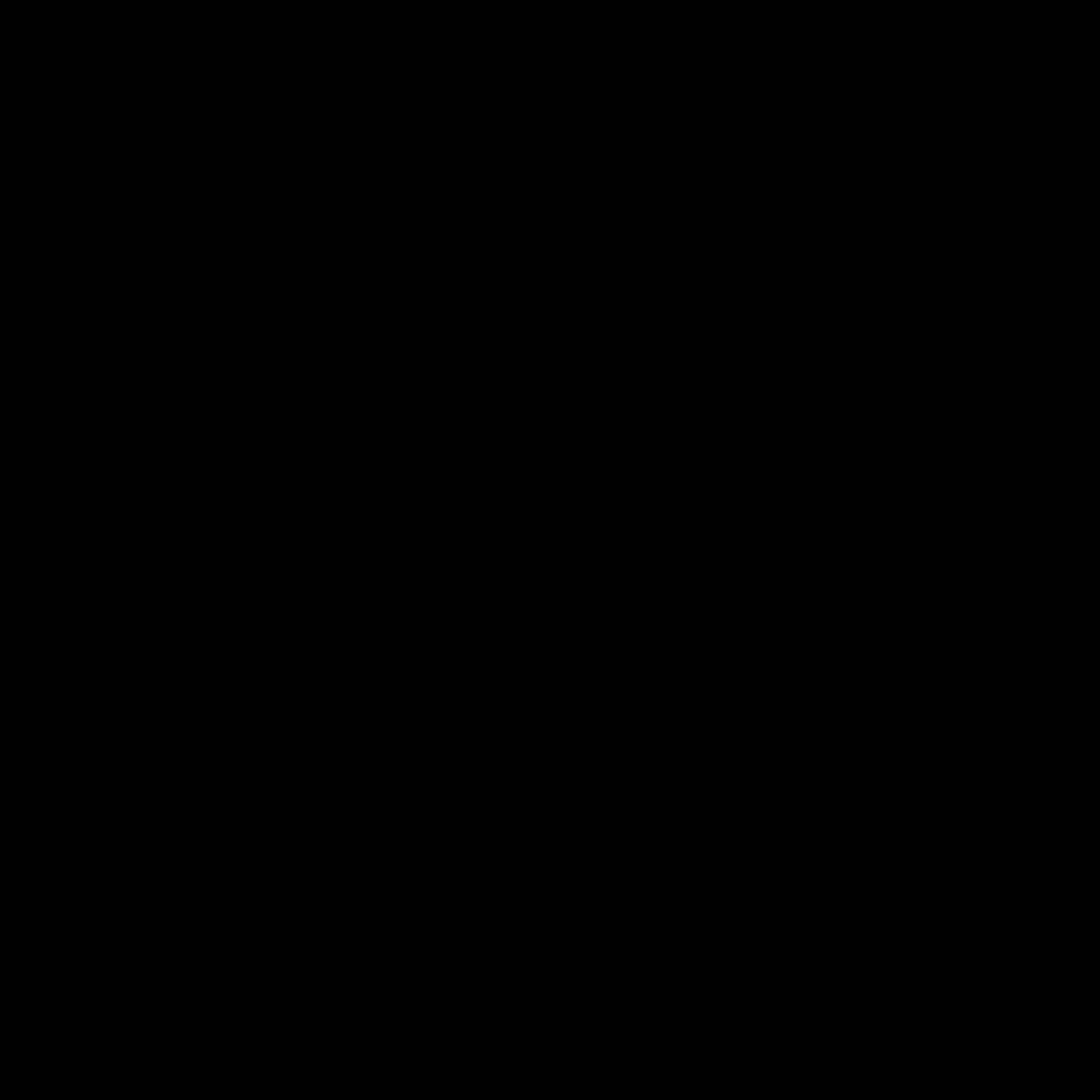

Supplement: S2 Data — (ZIP) [file ppat.1012014.s009.zip › C/C-2/siERK+Mock Cap.tif]

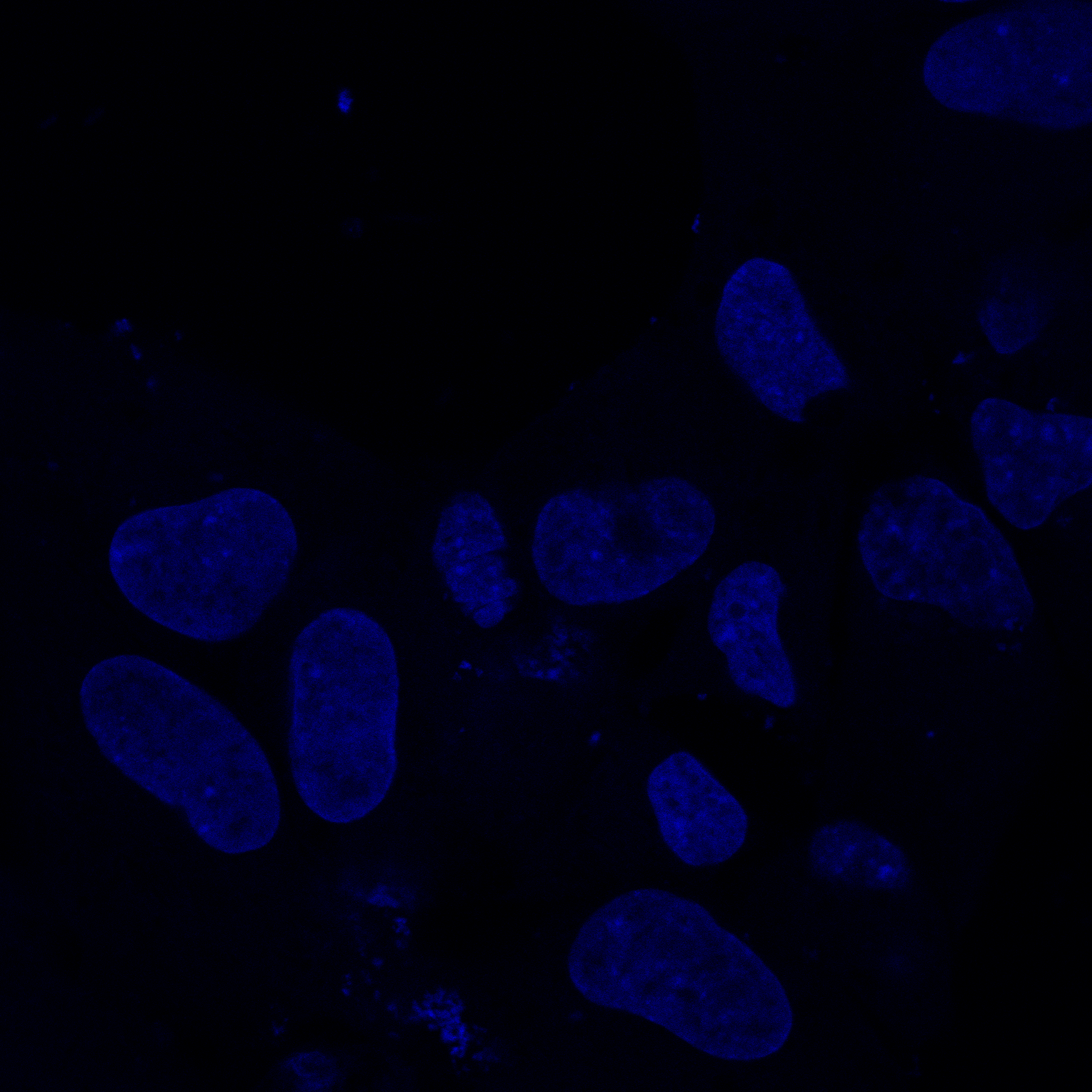

Supplement: S2 Data — (ZIP) [file ppat.1012014.s009.zip › C/C-2/siERK+Mock DAPI.tif]

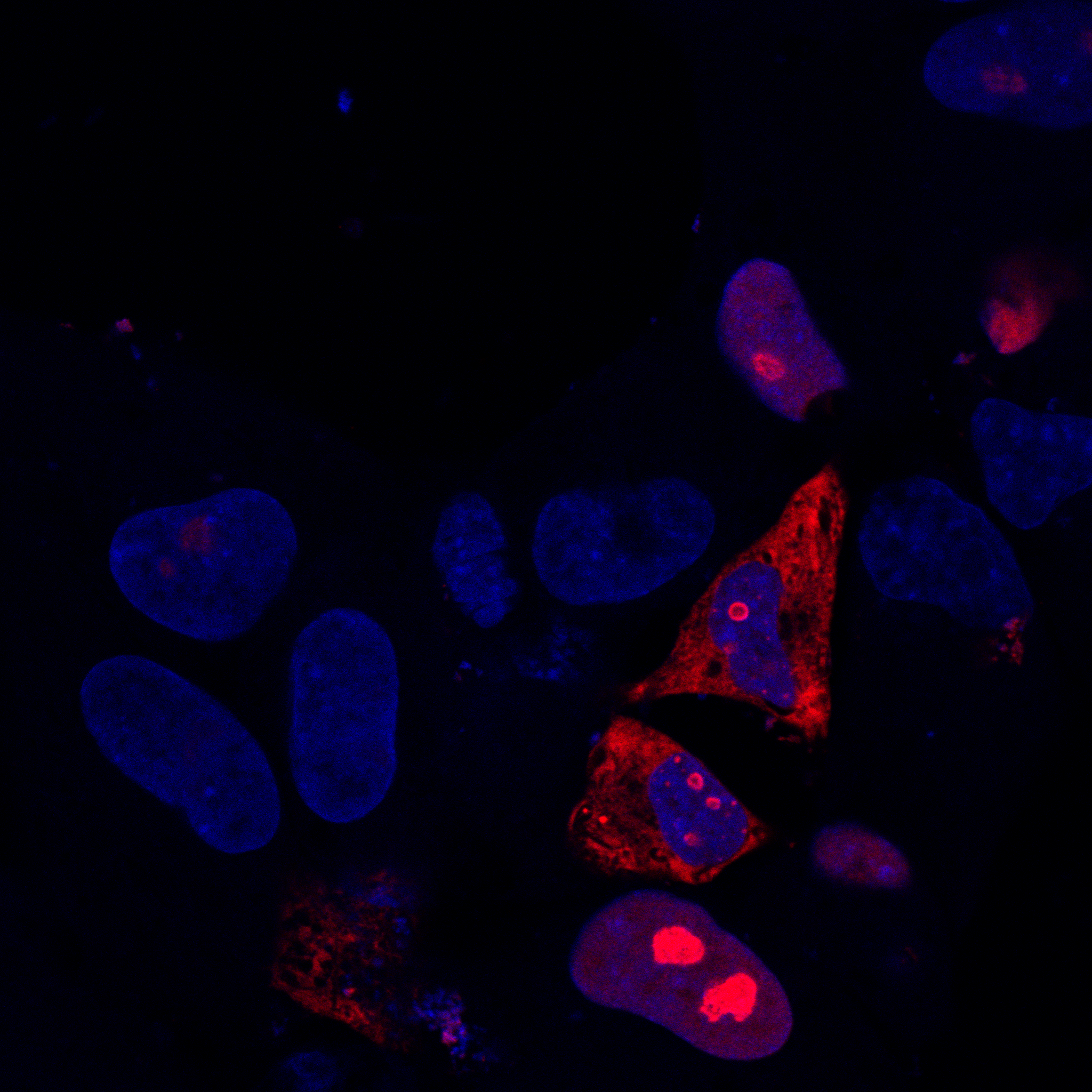

Supplement: S2 Data — (ZIP) [file ppat.1012014.s009.zip › C/C-2/siERK+Mock Merge.tif]

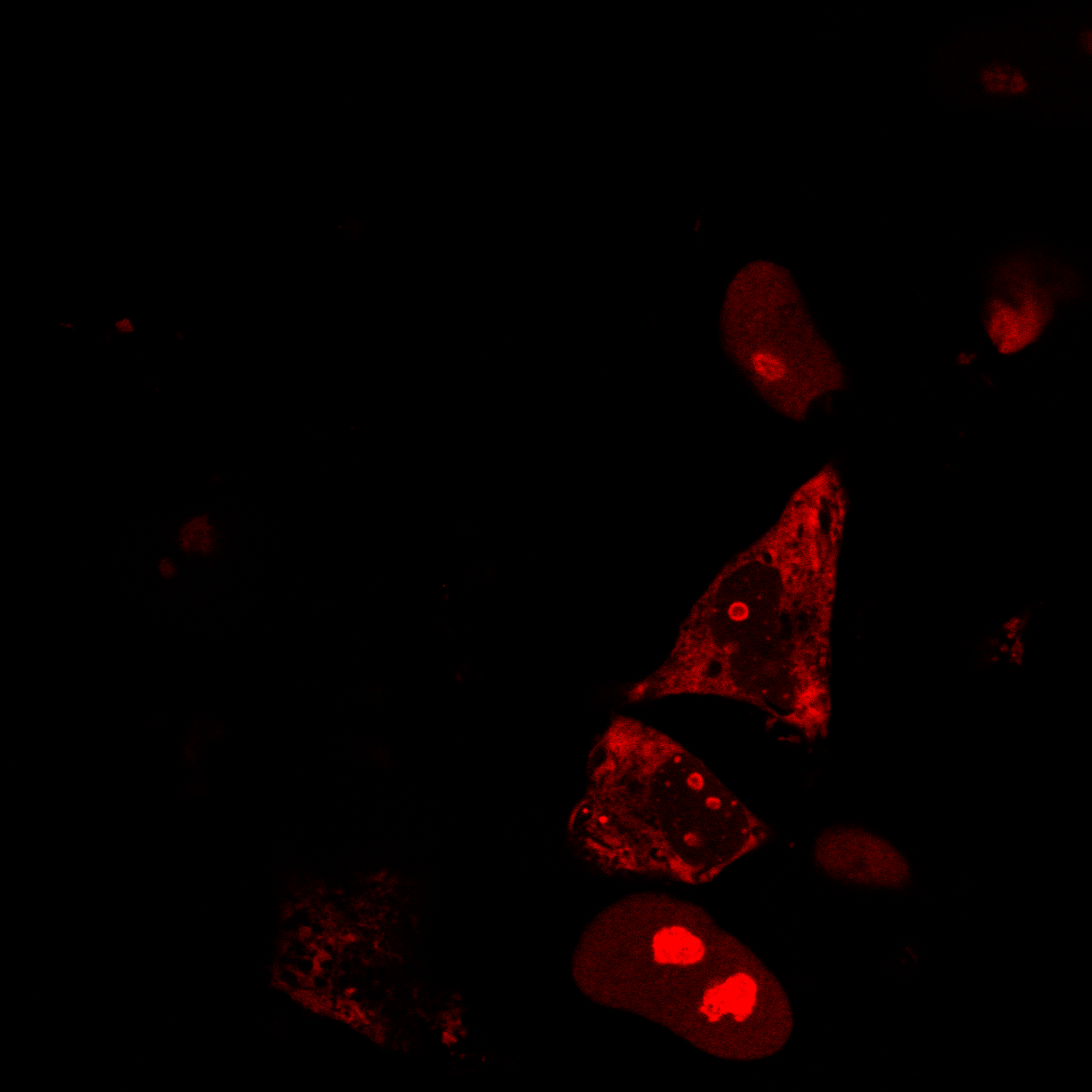

Supplement: S2 Data — (ZIP) [file ppat.1012014.s009.zip › C/C-2/siERK+Mock NPM1.tif]

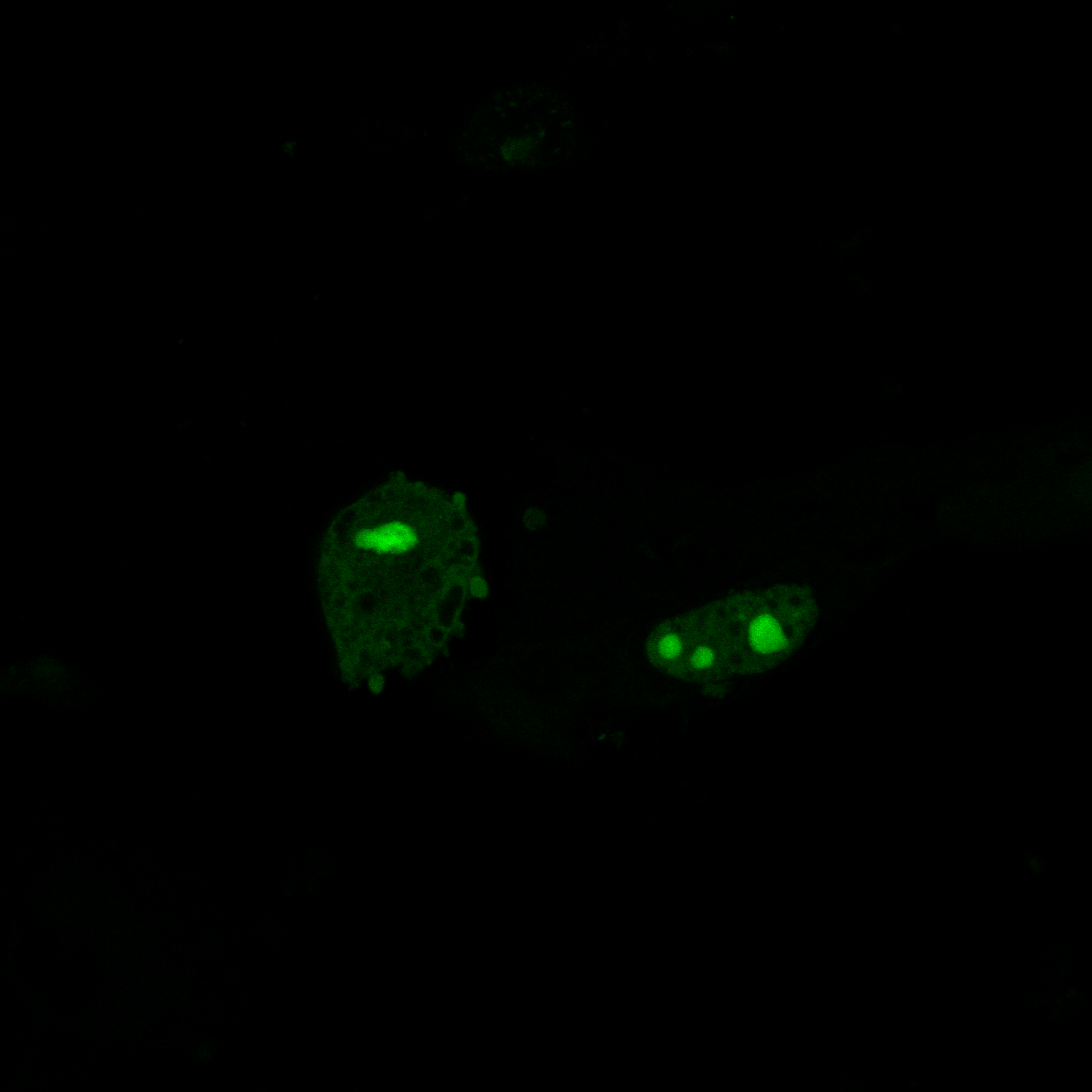

Supplement: S2 Data — (ZIP) [file ppat.1012014.s009.zip › C/C-2/siERK+PCV2 Cap.tif]

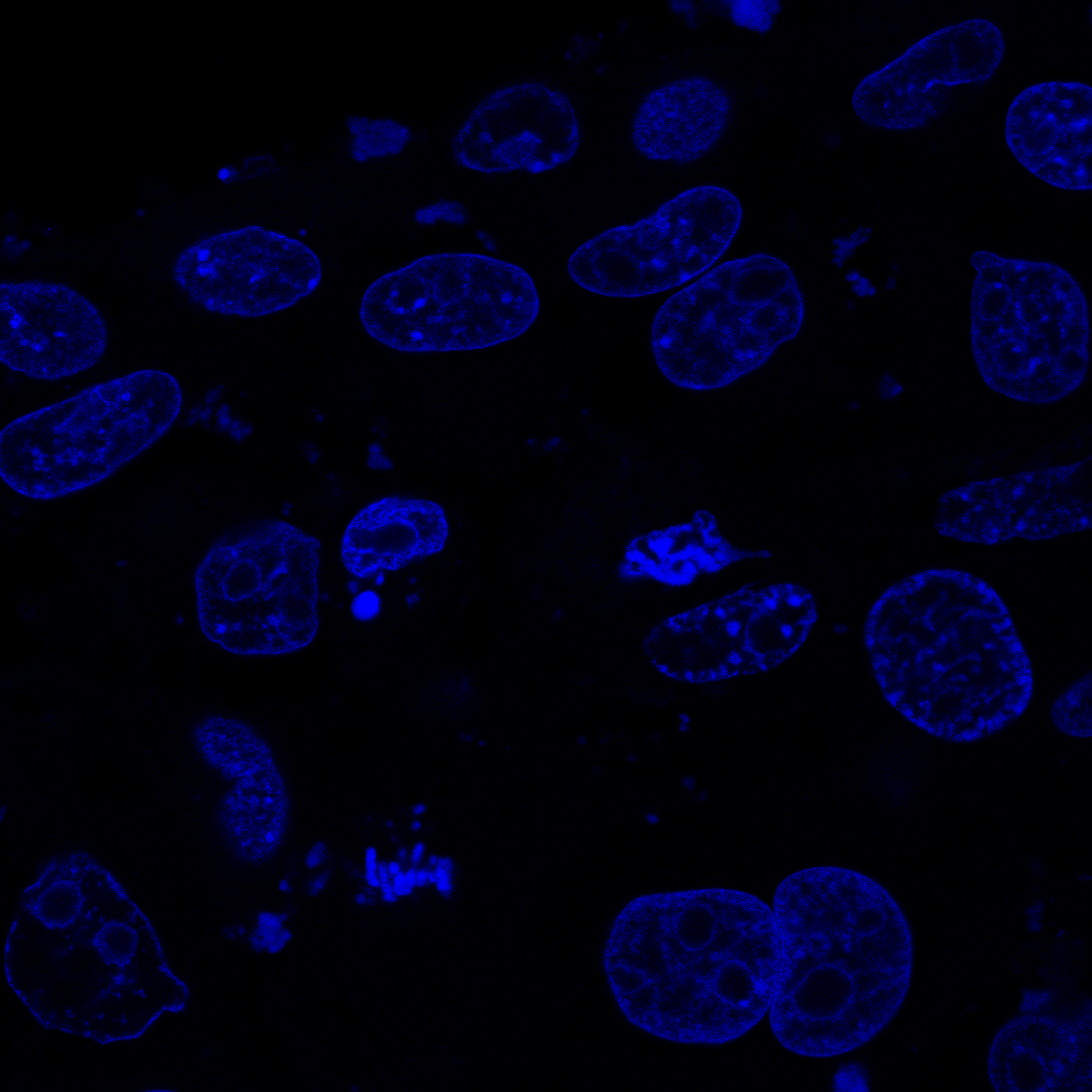

Supplement: S2 Data — (ZIP) [file ppat.1012014.s009.zip › C/C-2/siERK+PCV2 DAPI.tif]

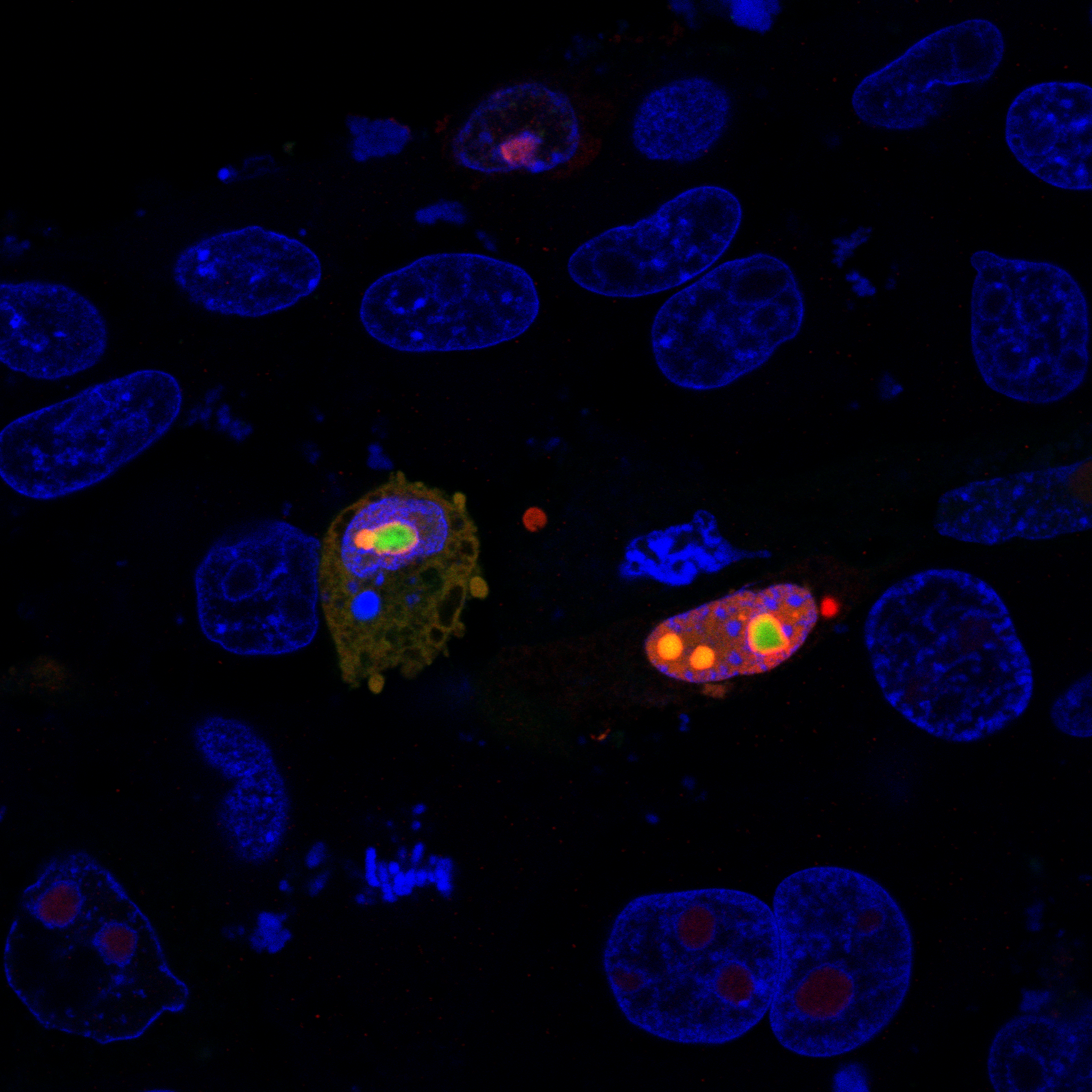

Supplement: S2 Data — (ZIP) [file ppat.1012014.s009.zip › C/C-2/siERK+PCV2 Merge.tif]

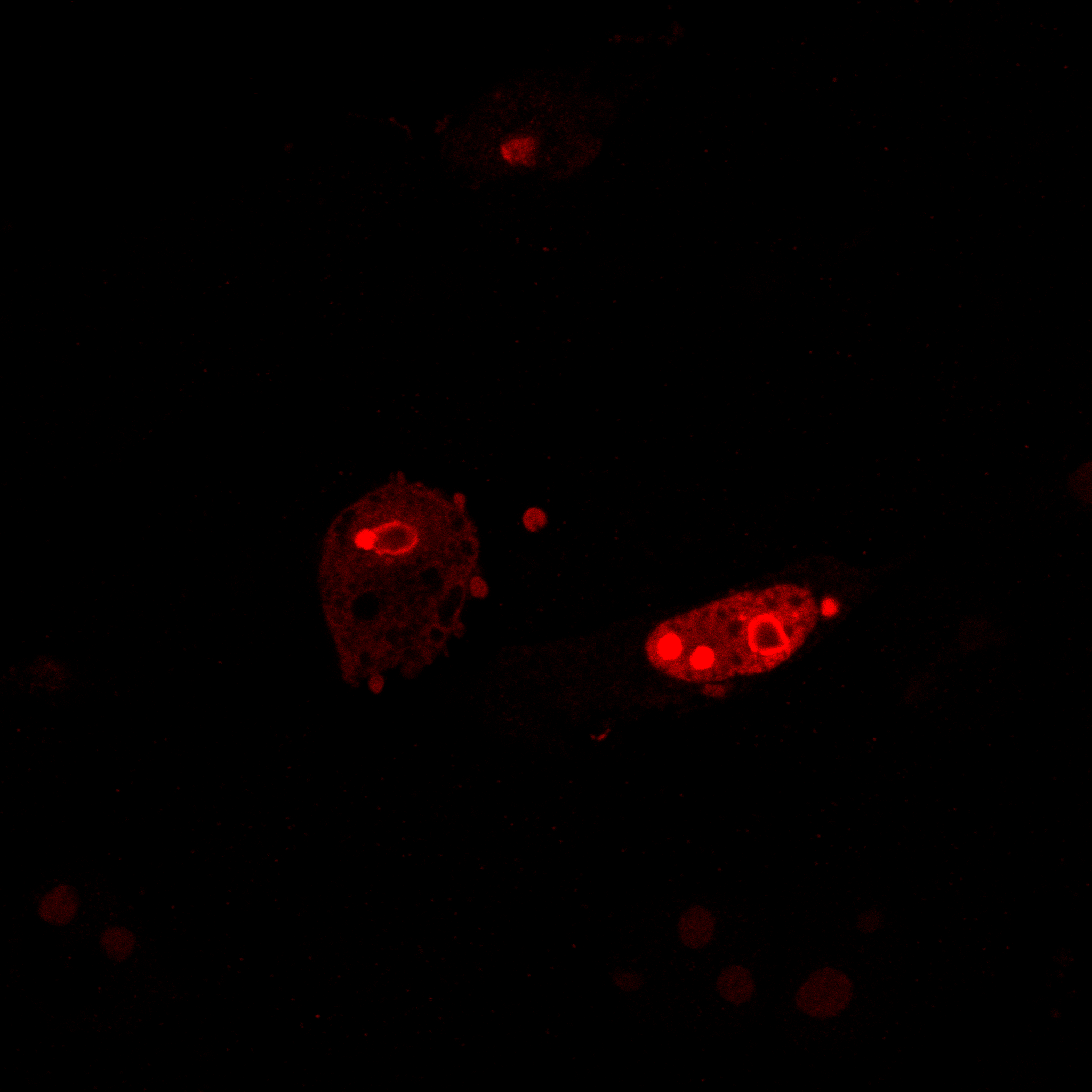

Supplement: S2 Data — (ZIP) [file ppat.1012014.s009.zip › C/C-2/siERK+PCV2 NPM1.tif]

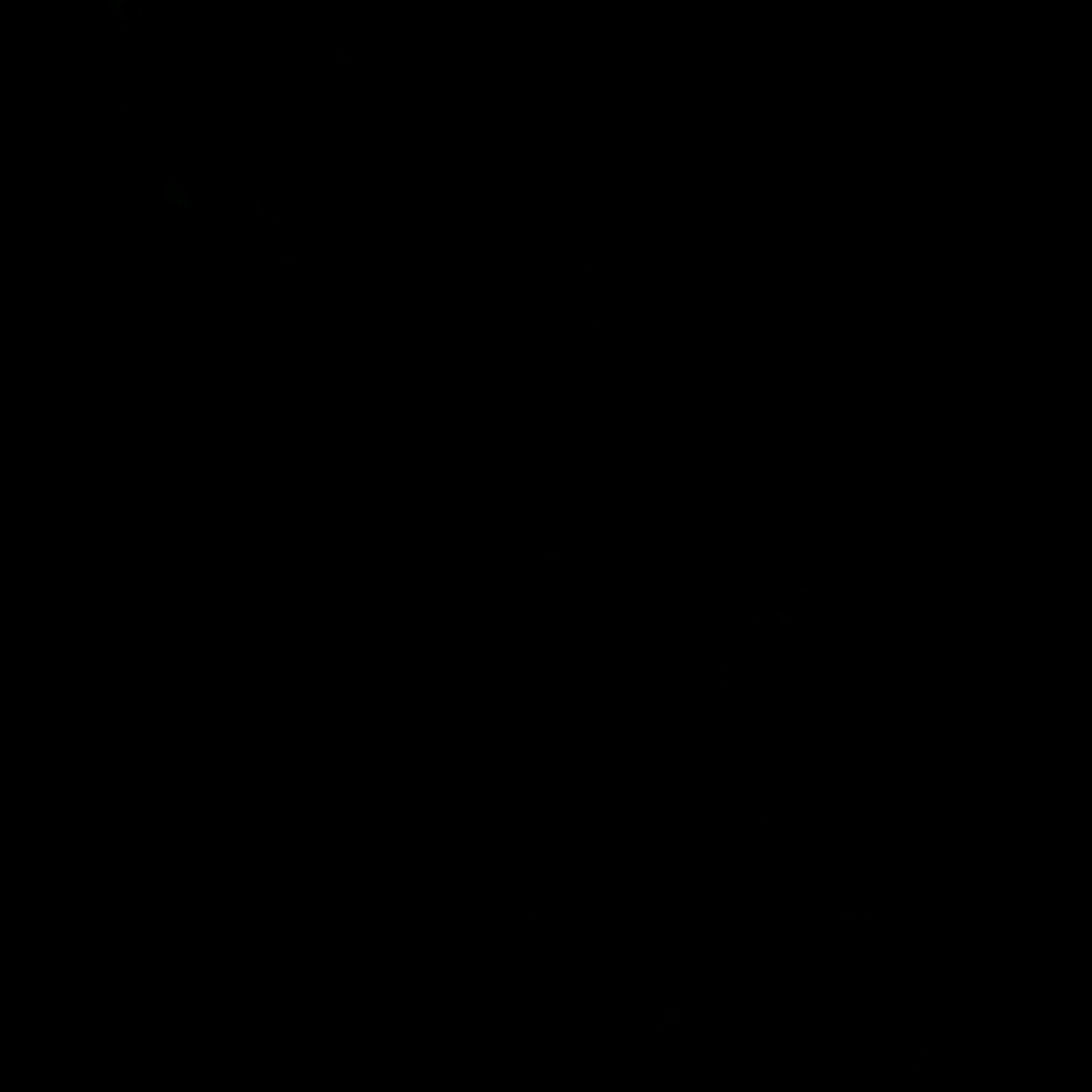

Supplement: S2 Data — (ZIP) [file ppat.1012014.s009.zip › C/C-2/siNC+Mock Cap.tif]

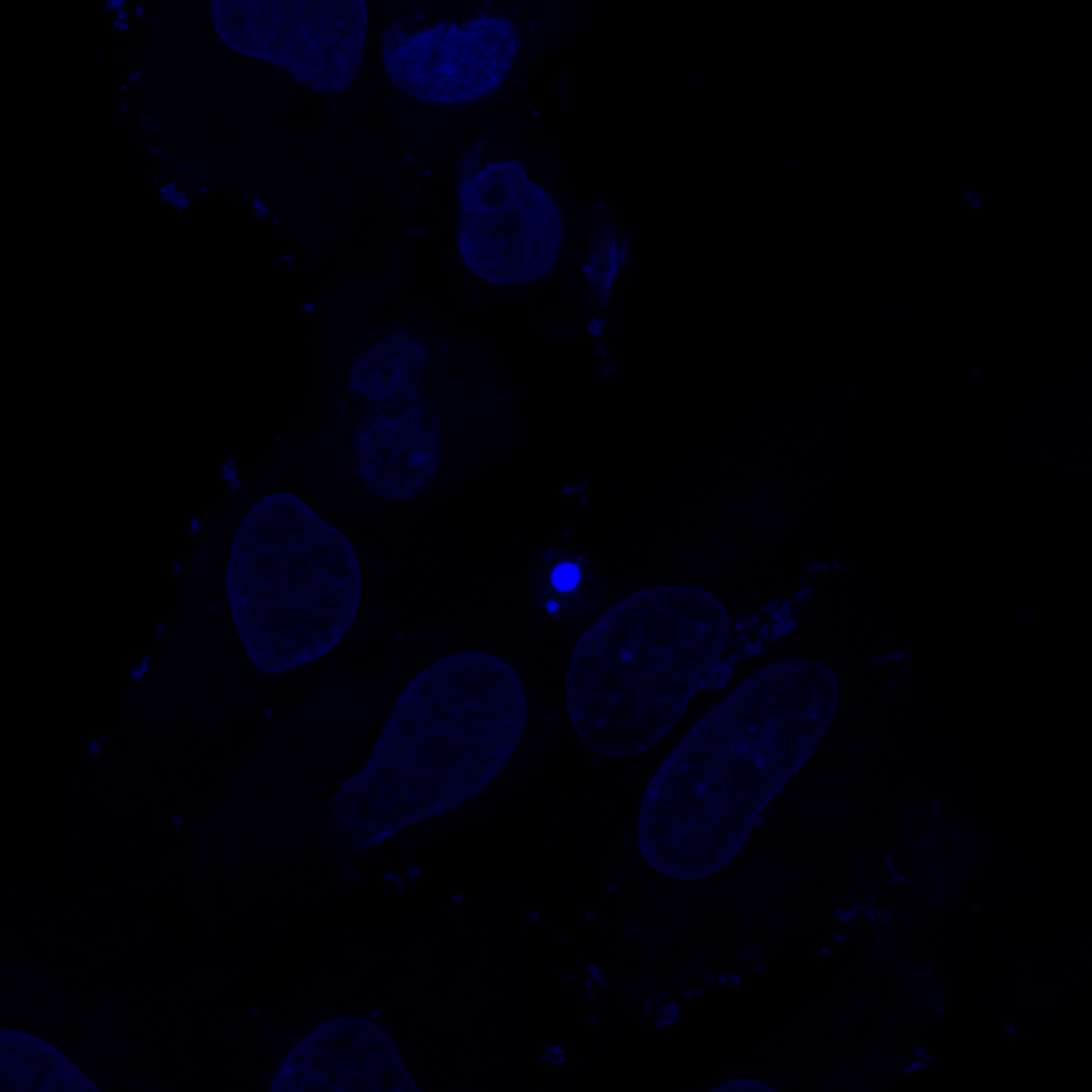

Supplement: S2 Data — (ZIP) [file ppat.1012014.s009.zip › C/C-2/siNC+Mock DAPI.tif]

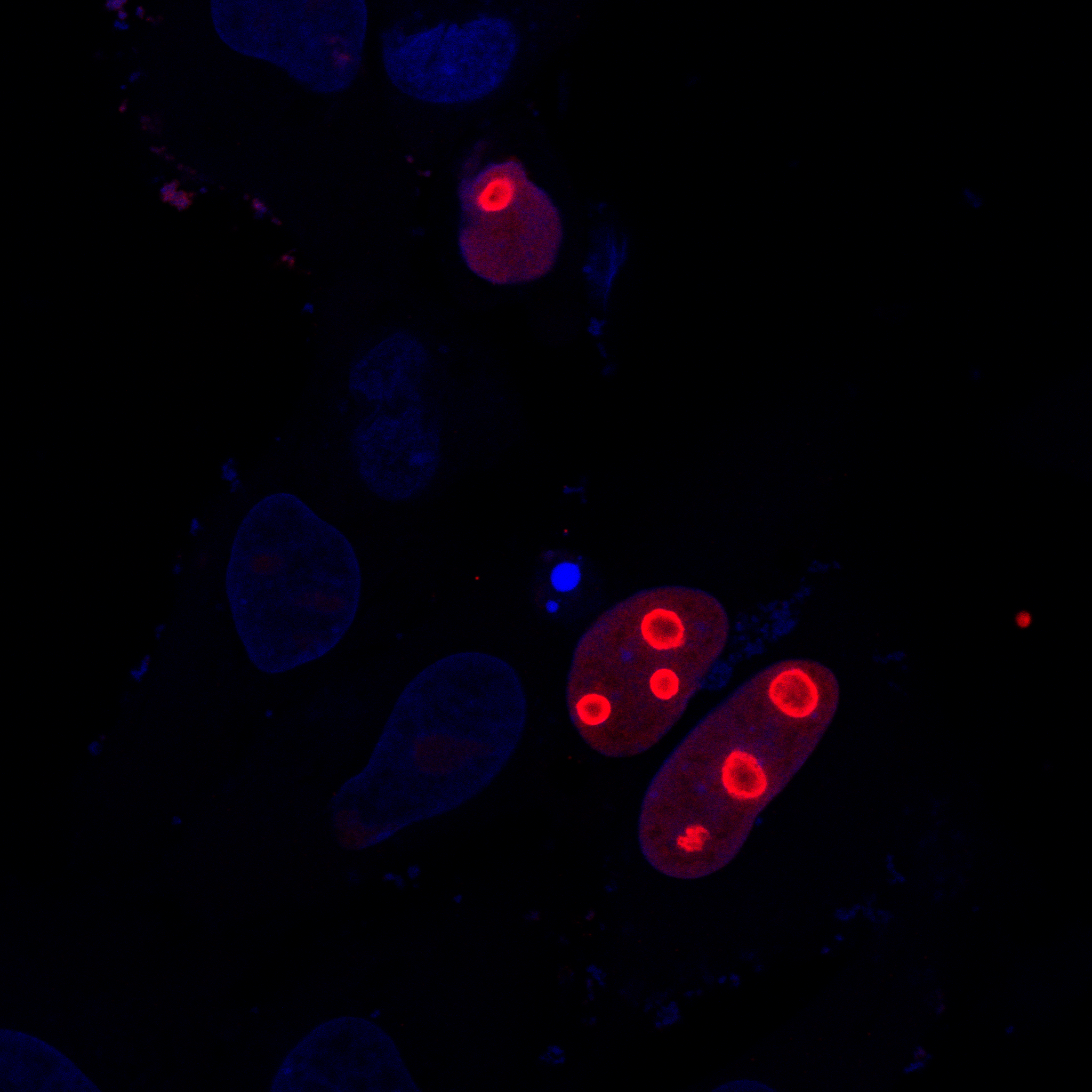

Supplement: S2 Data — (ZIP) [file ppat.1012014.s009.zip › C/C-2/siNC+Mock Merge.tif]

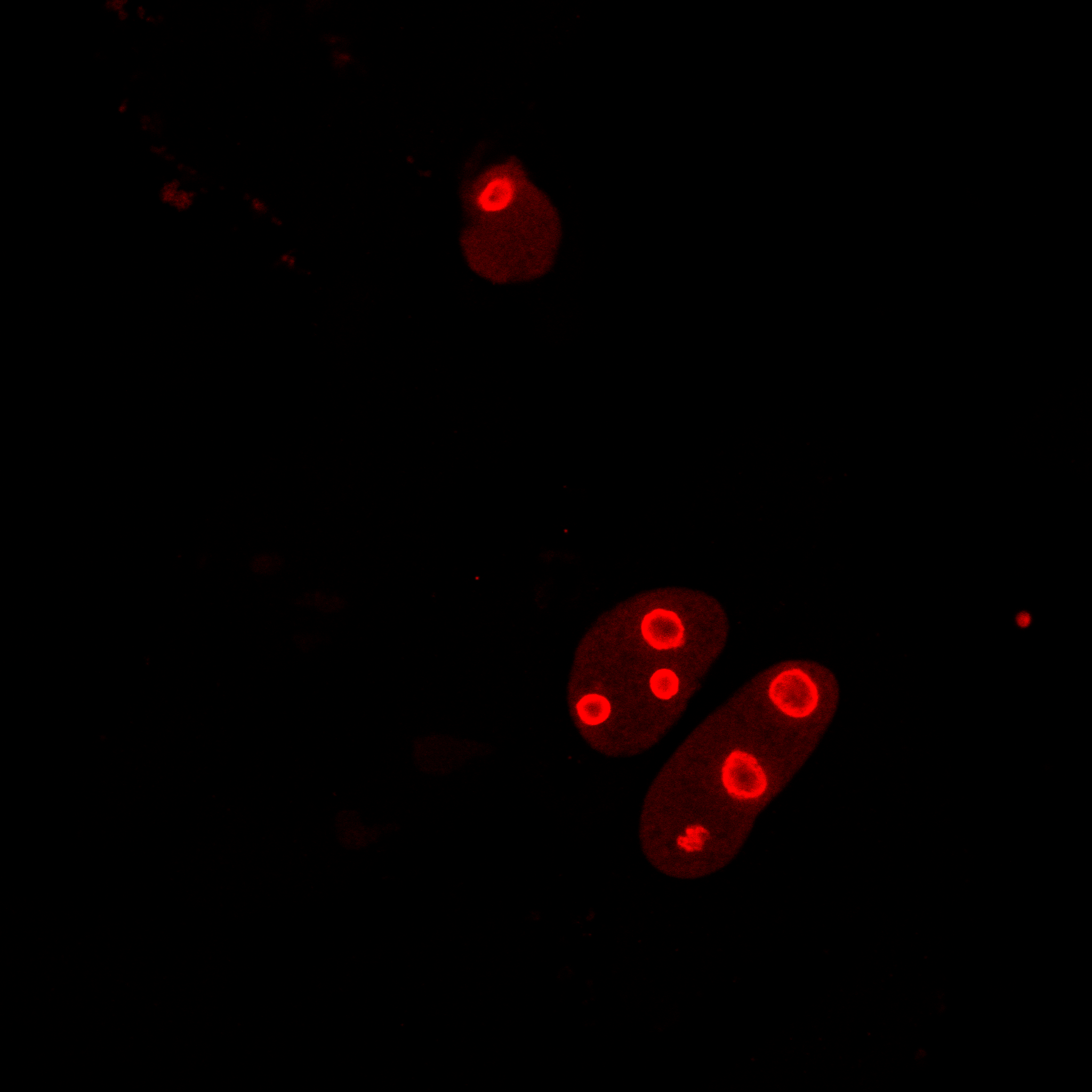

Supplement: S2 Data — (ZIP) [file ppat.1012014.s009.zip › C/C-2/siNC+Mock NPM1.tif]

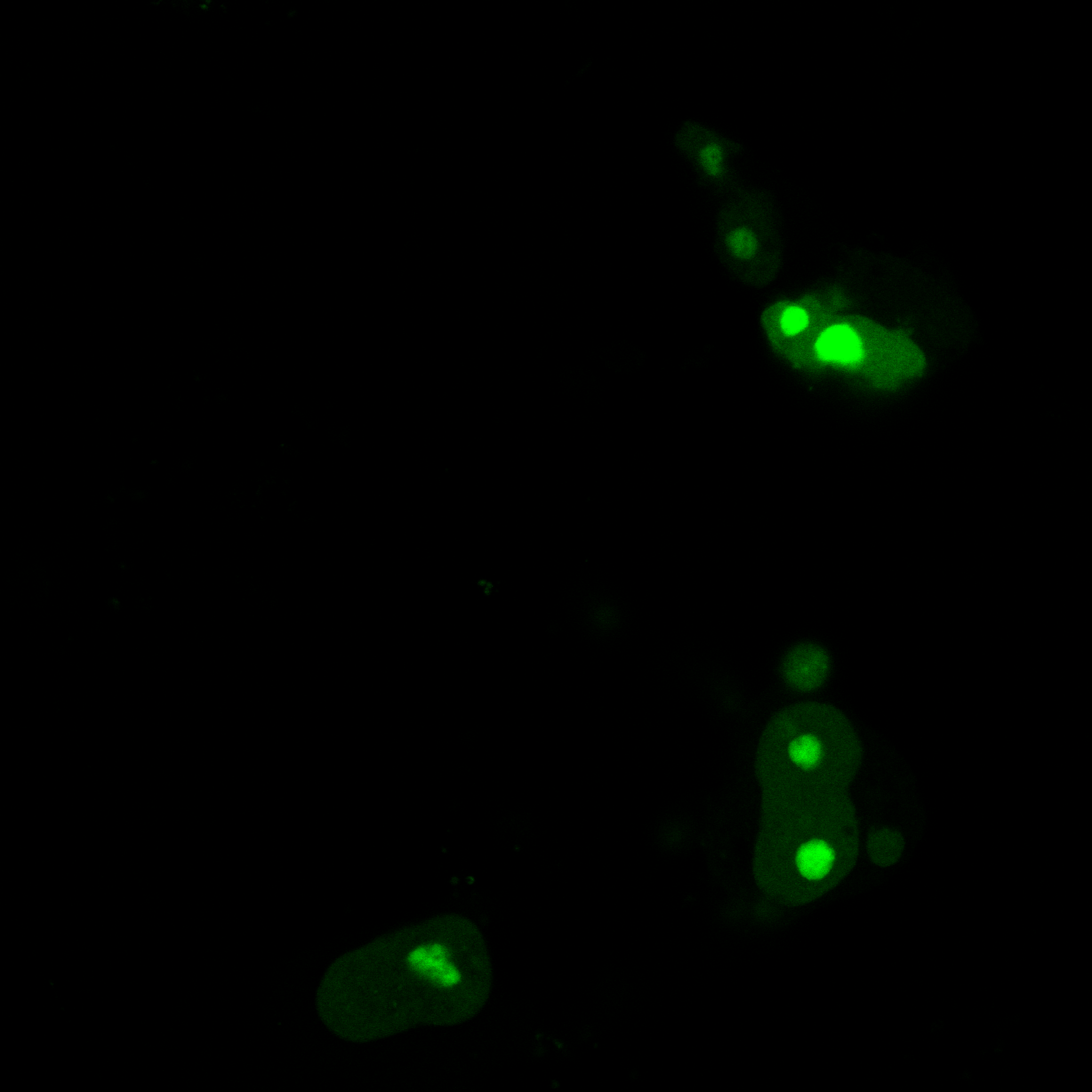

Supplement: S2 Data — (ZIP) [file ppat.1012014.s009.zip › C/C-2/siNC+PCV2 Cap.tif]

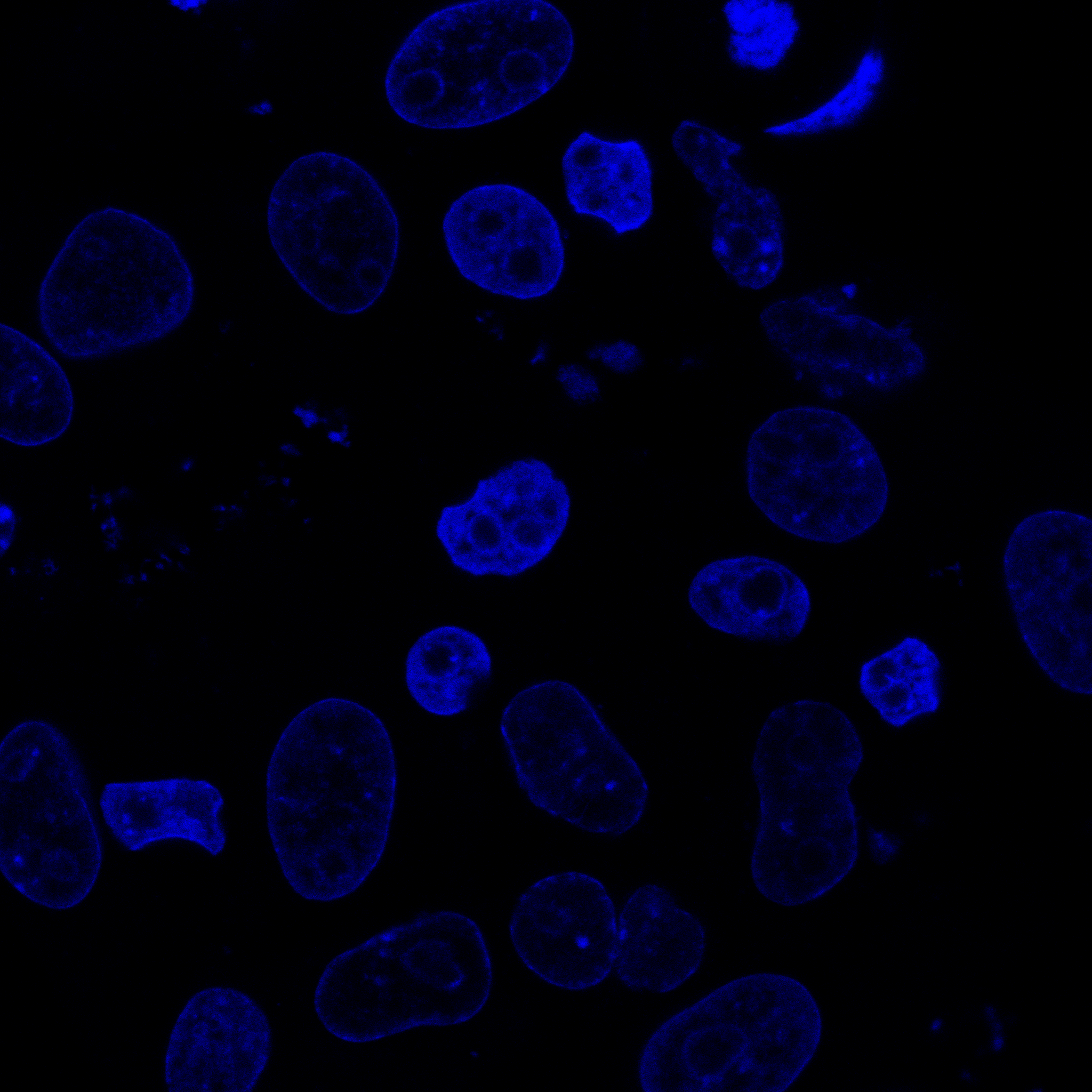

Supplement: S2 Data — (ZIP) [file ppat.1012014.s009.zip › C/C-2/siNC+PCV2 DAPI.tif]

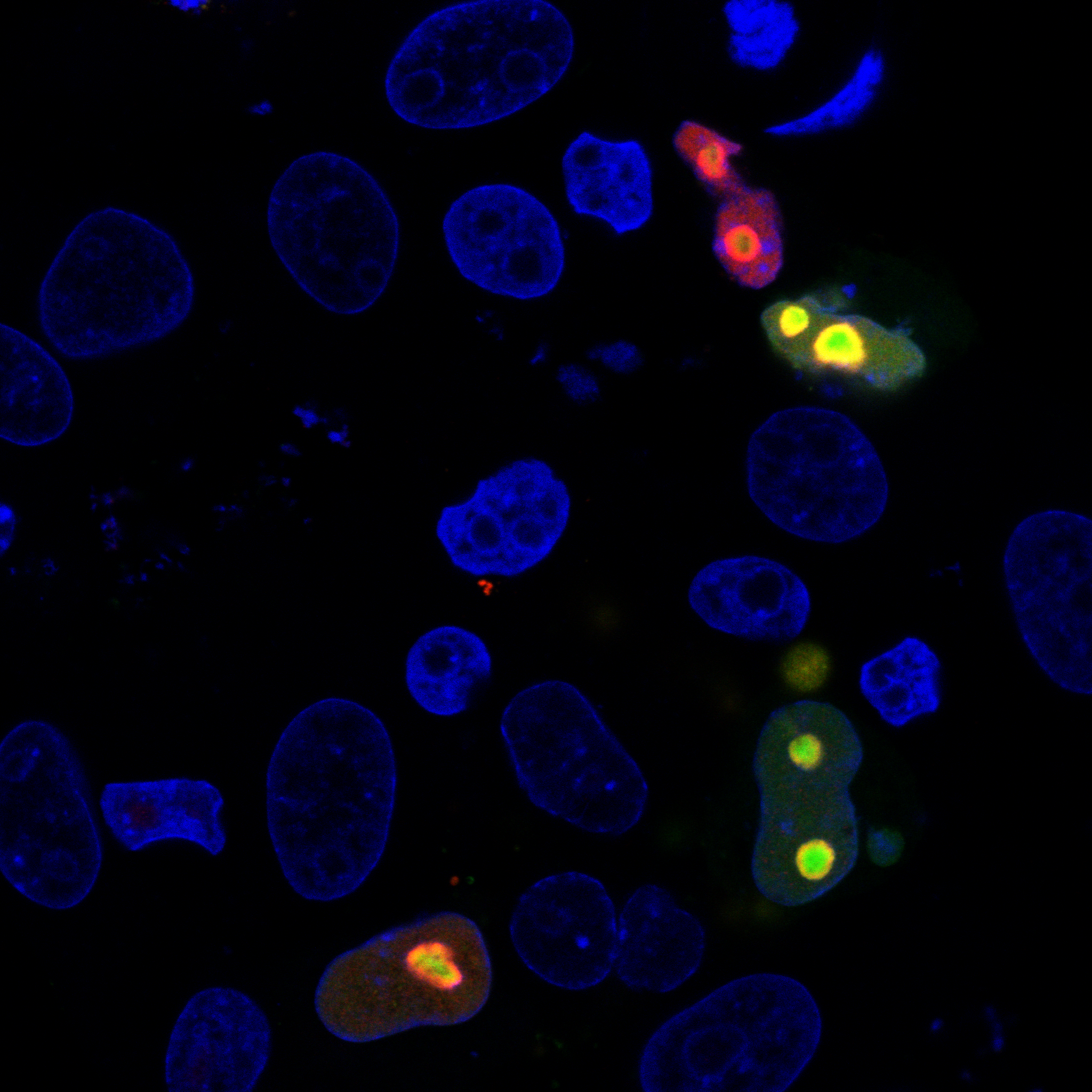

Supplement: S2 Data — (ZIP) [file ppat.1012014.s009.zip › C/C-2/siNC+PCV2 Merge.tif]

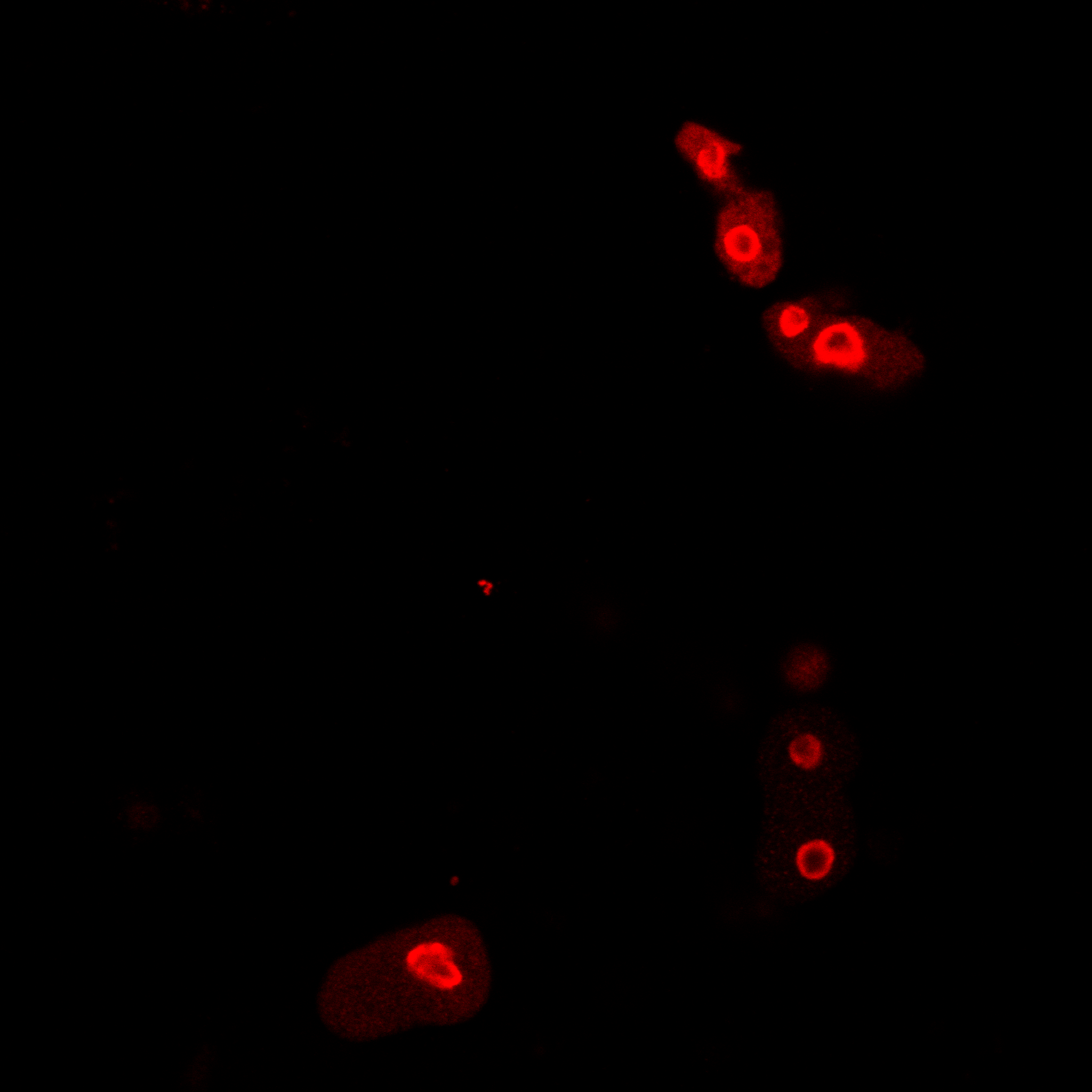

Supplement: S2 Data — (ZIP) [file ppat.1012014.s009.zip › C/C-2/siNC+PCV2 NPM1.tif]

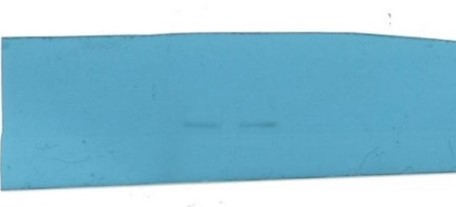

Supplement: S2 Data — (ZIP) [file ppat.1012014.s009.zip › D/D-1/Cytoplasm-Cap-1.jpg]

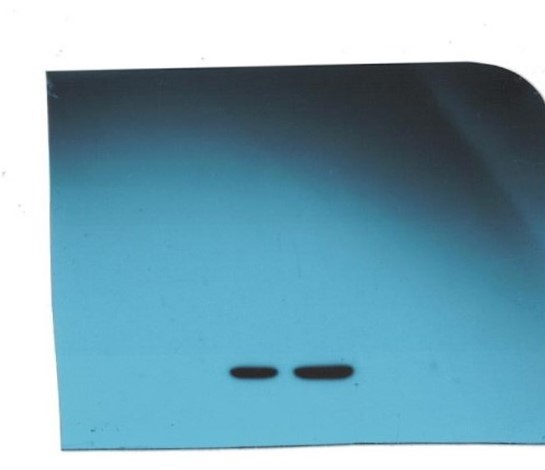

Supplement: S2 Data — (ZIP) [file ppat.1012014.s009.zip › D/D-1/Cytoplasm-NPM1-1.jpg]

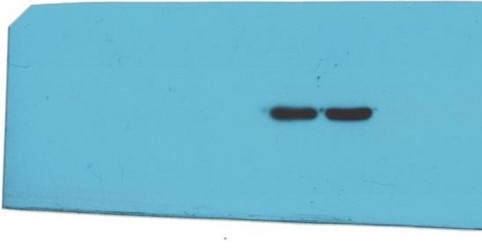

Supplement: S2 Data — (ZIP) [file ppat.1012014.s009.zip › D/D-1/Cytoplasm-β-actin-1.jpg]

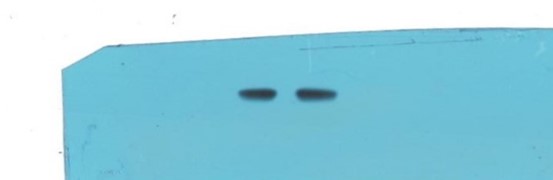

Supplement: S2 Data — (ZIP) [file ppat.1012014.s009.zip › D/D-1/Nuclear-Cap-1.jpg]

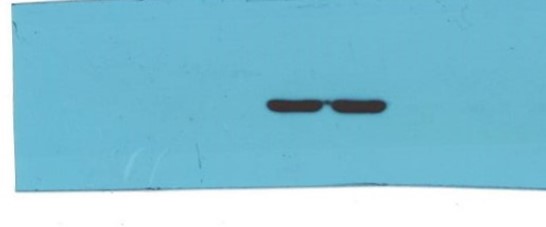

Supplement: S2 Data — (ZIP) [file ppat.1012014.s009.zip › D/D-1/Nuclear-Lamin B-1.jpg]

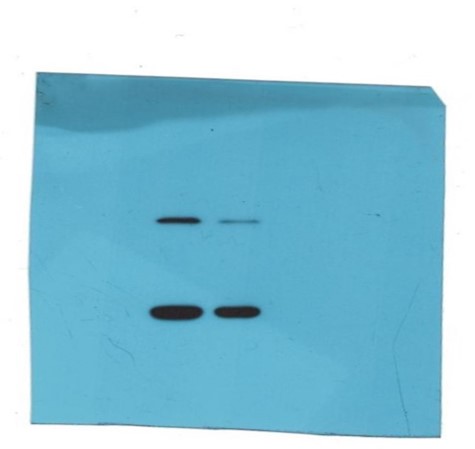

Supplement: S2 Data — (ZIP) [file ppat.1012014.s009.zip › D/D-1/Nuclear-NPM1-1.jpg]

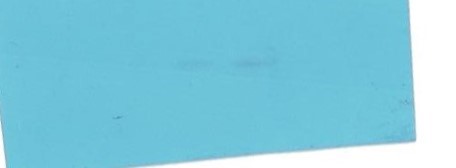

Supplement: S2 Data — (ZIP) [file ppat.1012014.s009.zip › D/D-2/Cap-Cytoplasm.jpg]

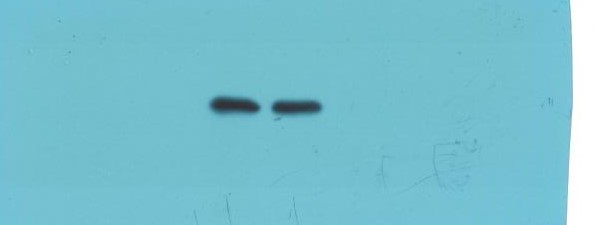

Supplement: S2 Data — (ZIP) [file ppat.1012014.s009.zip › D/D-2/Cap-nuclear.jpg]

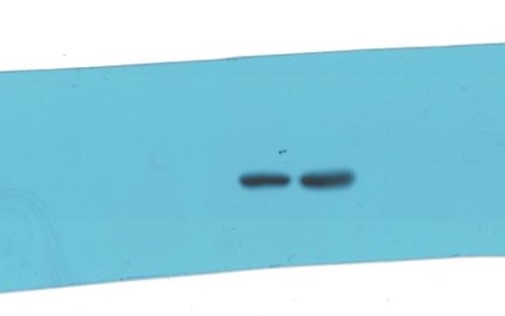

Supplement: S2 Data — (ZIP) [file ppat.1012014.s009.zip › D/D-2/F7-D-LaminB.jpg]

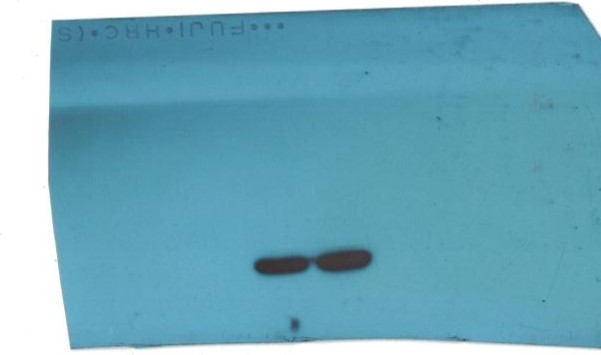

Supplement: S2 Data — (ZIP) [file ppat.1012014.s009.zip › D/D-2/NPM1-Cytoplasm.jpg]

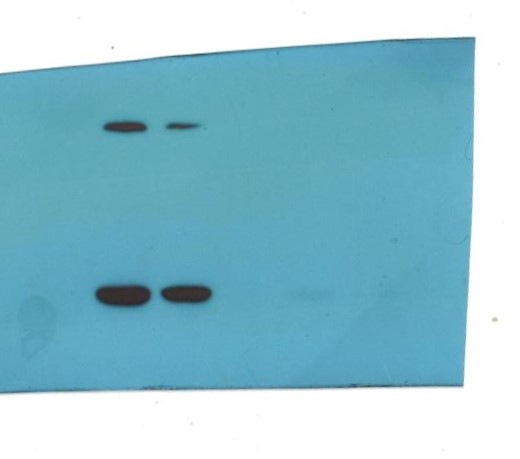

Supplement: S2 Data — (ZIP) [file ppat.1012014.s009.zip › D/D-2/NPM1-Nuclear.jpg]

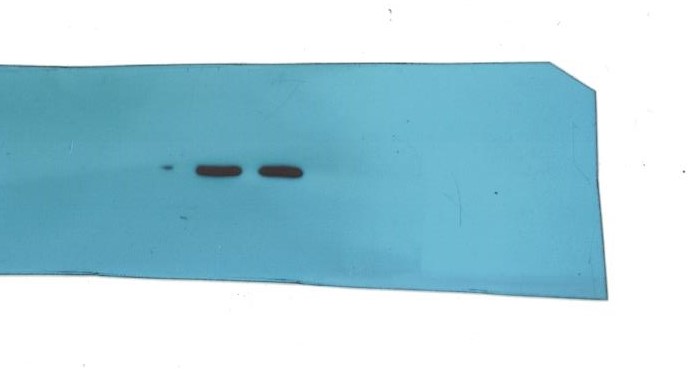

Supplement: S2 Data — (ZIP) [file ppat.1012014.s009.zip › D/D-2/β-actin.jpg]

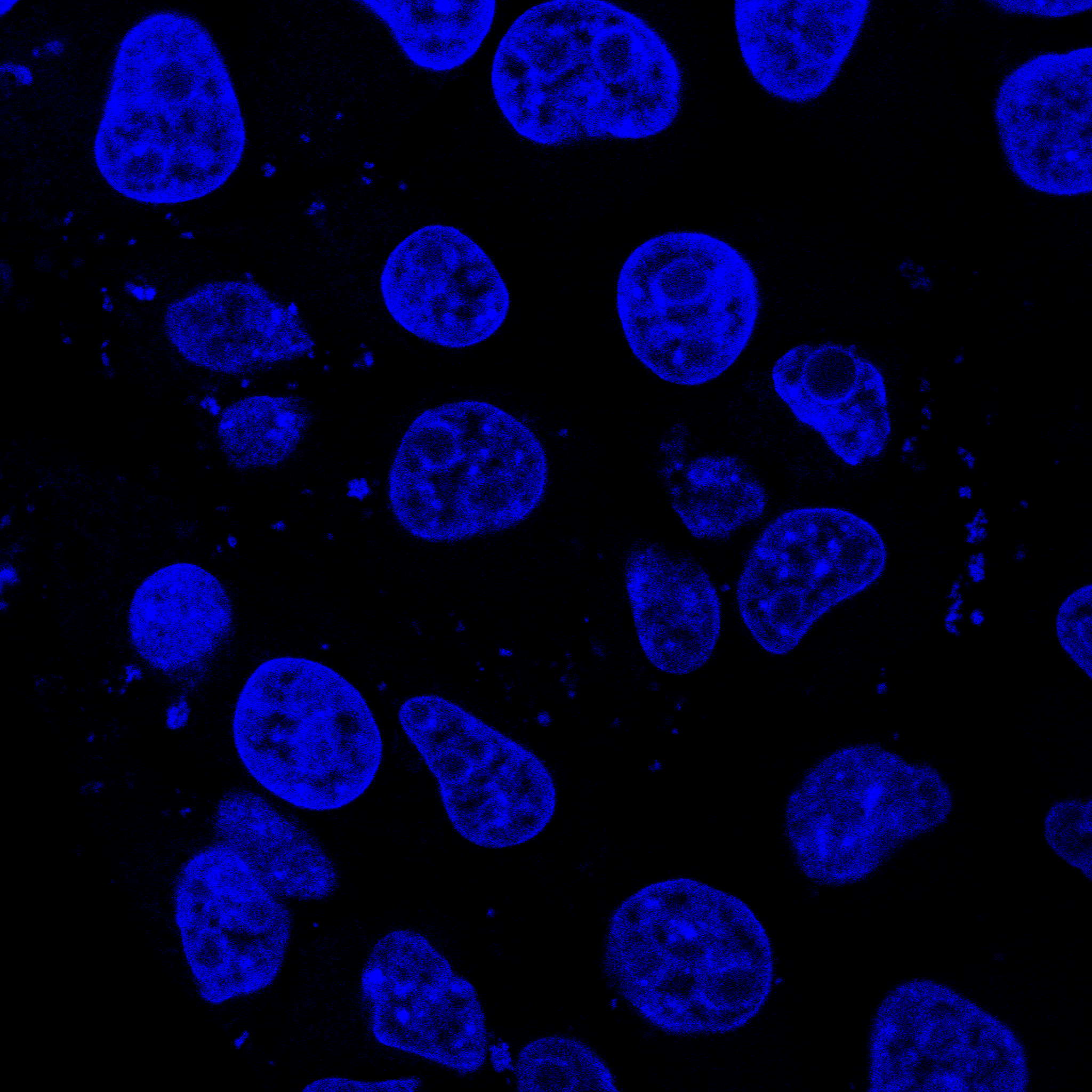

Supplement: S4 Data — (ZIP) [file ppat.1012014.s011.zip › A/A-1/siERK+rAd-Blank DAPI.tif]

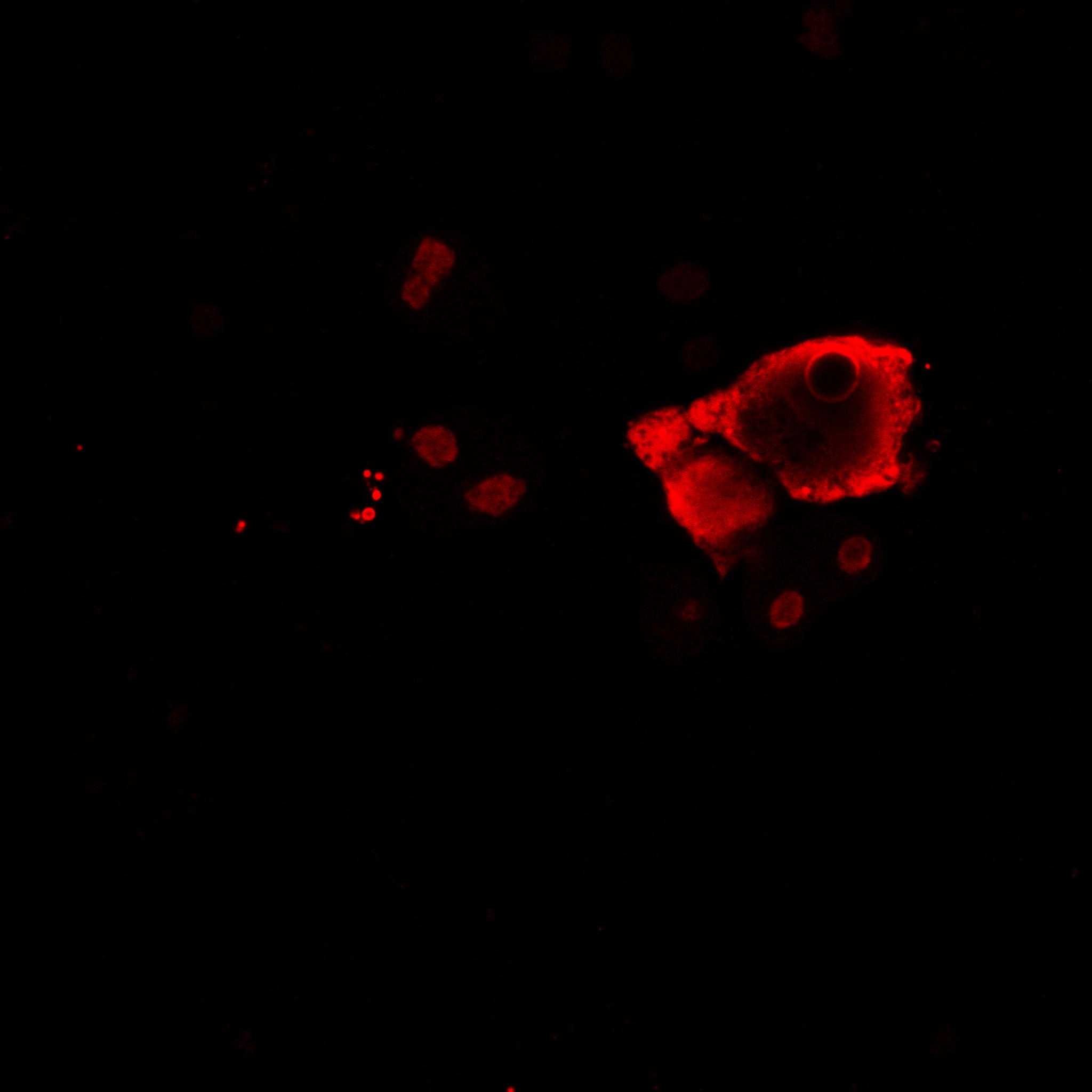

Supplement: S4 Data — (ZIP) [file ppat.1012014.s011.zip › A/A-1/siERK+rAd-Blank NPM1.tif]

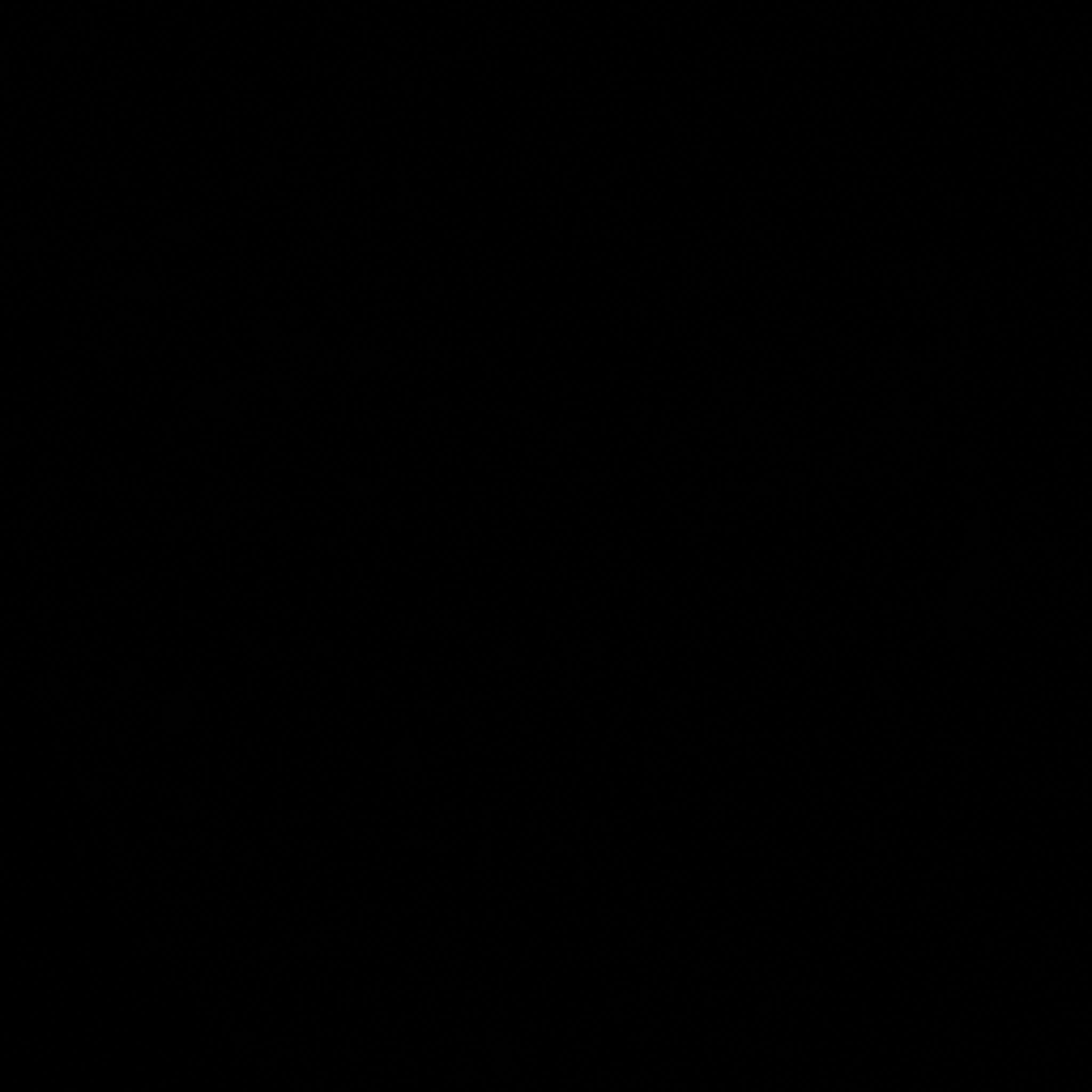

Supplement: S4 Data — (ZIP) [file ppat.1012014.s011.zip › A/A-1/siERK+rAd-Blank Cap.tif]

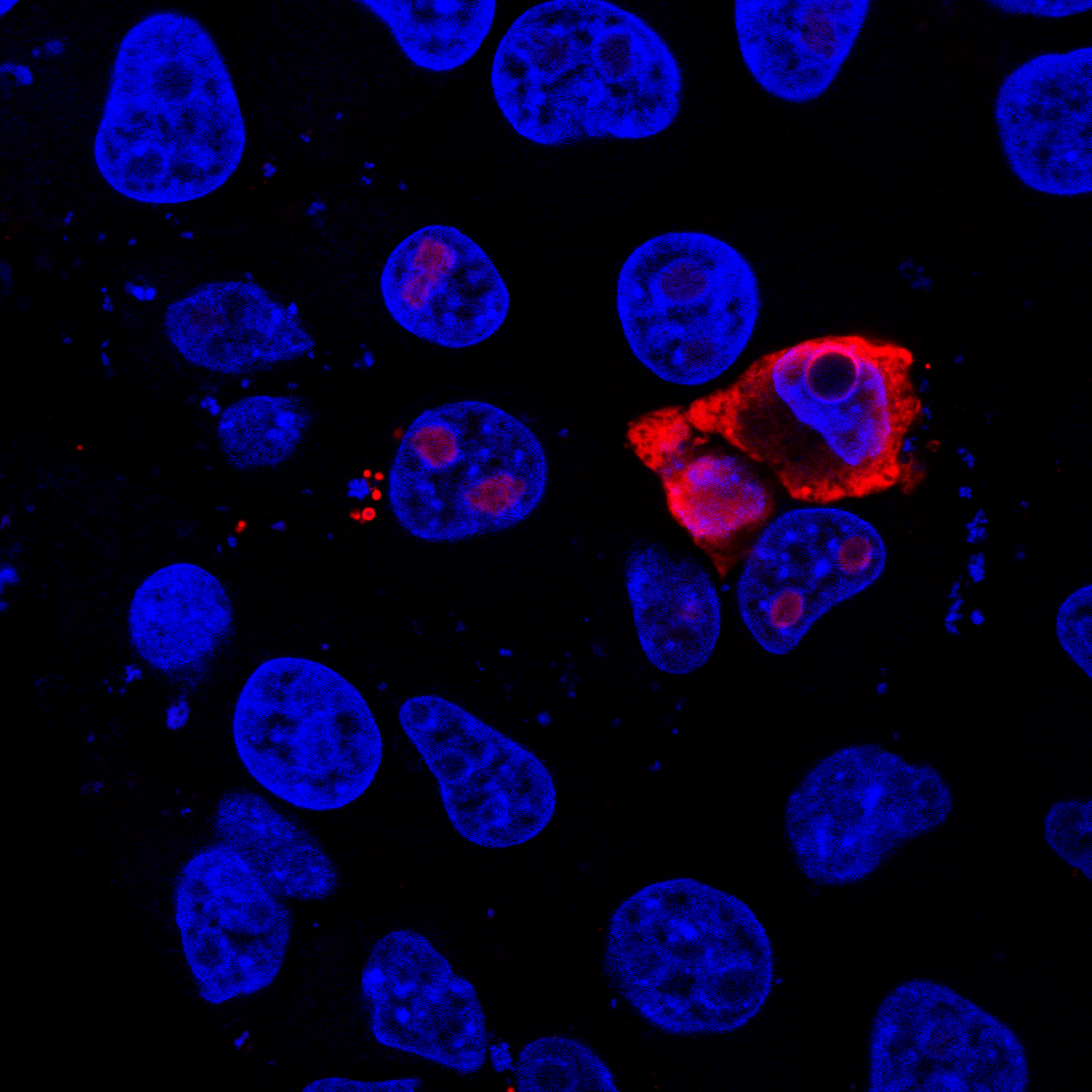

Supplement: S4 Data — (ZIP) [file ppat.1012014.s011.zip › A/A-1/siERK+rAd-Blank Merge.tif]

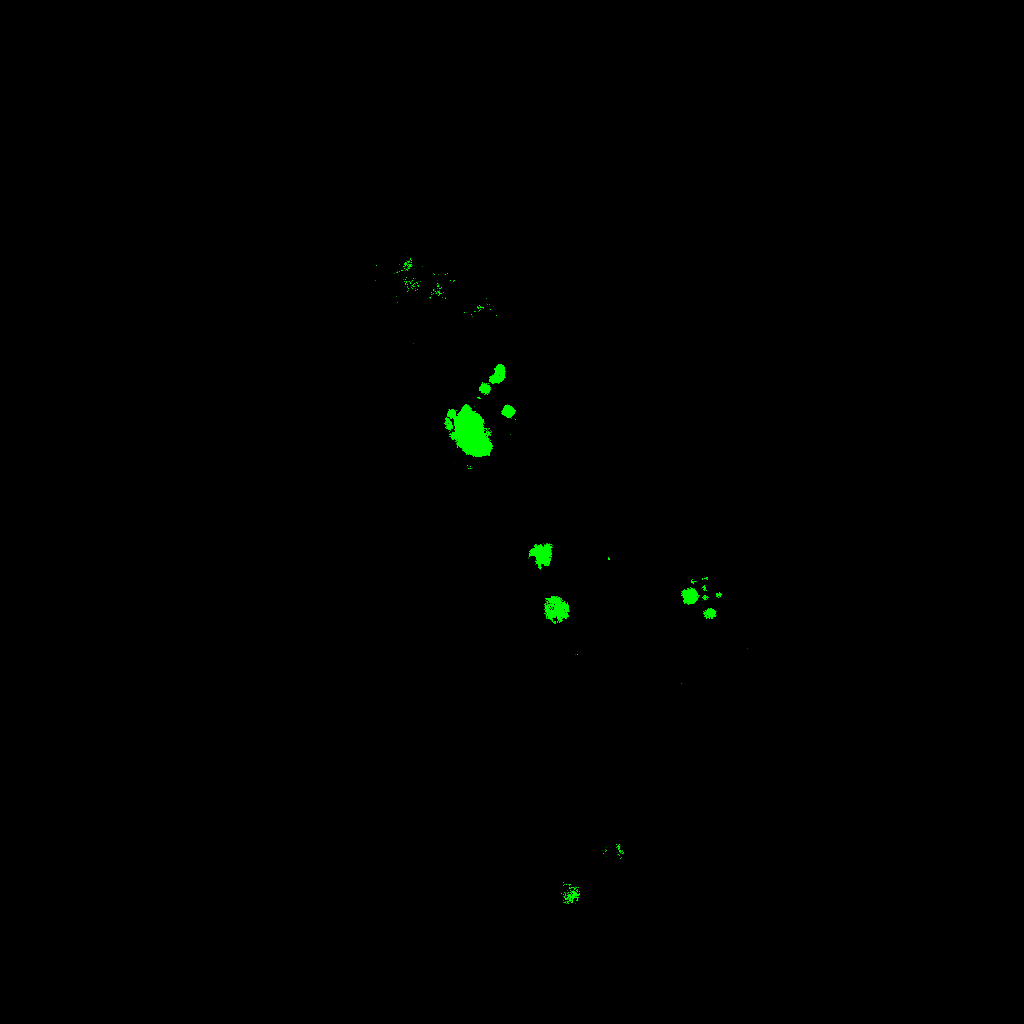

Supplement: S4 Data — (ZIP) [file ppat.1012014.s011.zip › A/A-1/siERK+rAd-Cap Cap.tif]
