# Supplementary figures and images for: Porcine circovirus type 2 infection promotes the SUMOylation of nucleophosmin-1 to facilitate the viral circular single-stranded DNA replication (part 2 of 2)
Source: PLoS Pathog. 2024 Feb 23;20(2):e1012014. doi: 10.1371/journal.ppat.1012014 (PMC10917307; doi:10.1371/journal.ppat.1012014)

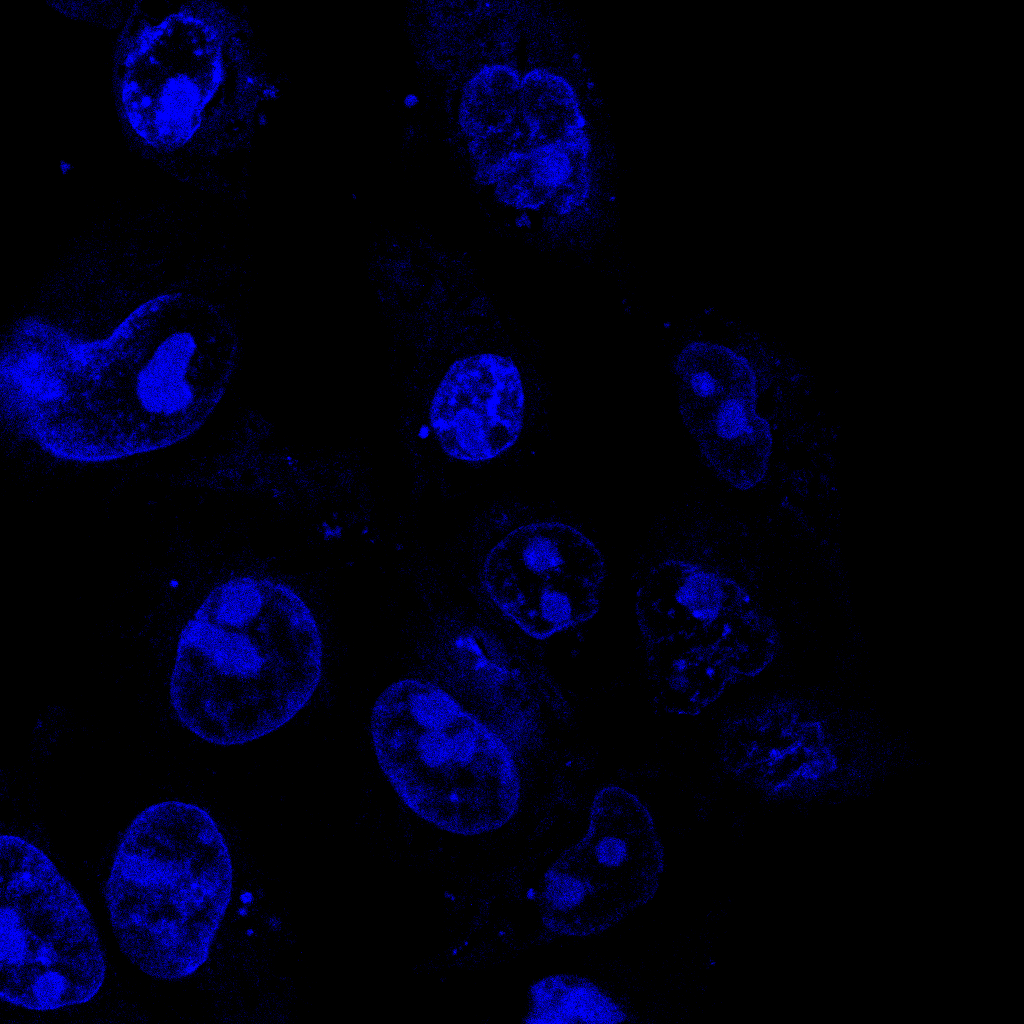

Supplement: S4 Data — (ZIP) [file ppat.1012014.s011.zip › A/A-1/siERK+rAd-Cap DAPI.tif]

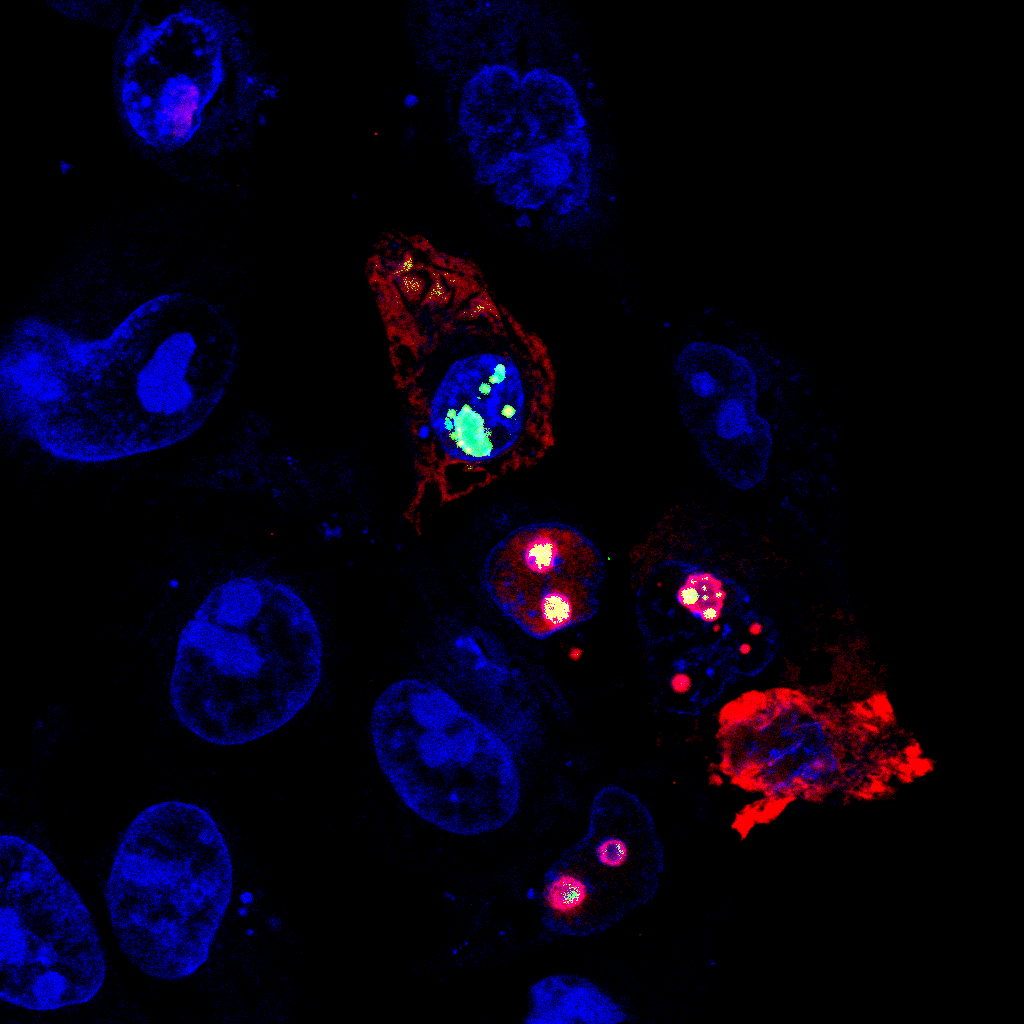

Supplement: S4 Data — (ZIP) [file ppat.1012014.s011.zip › A/A-1/siERK+rAd-Cap Merge.tif]

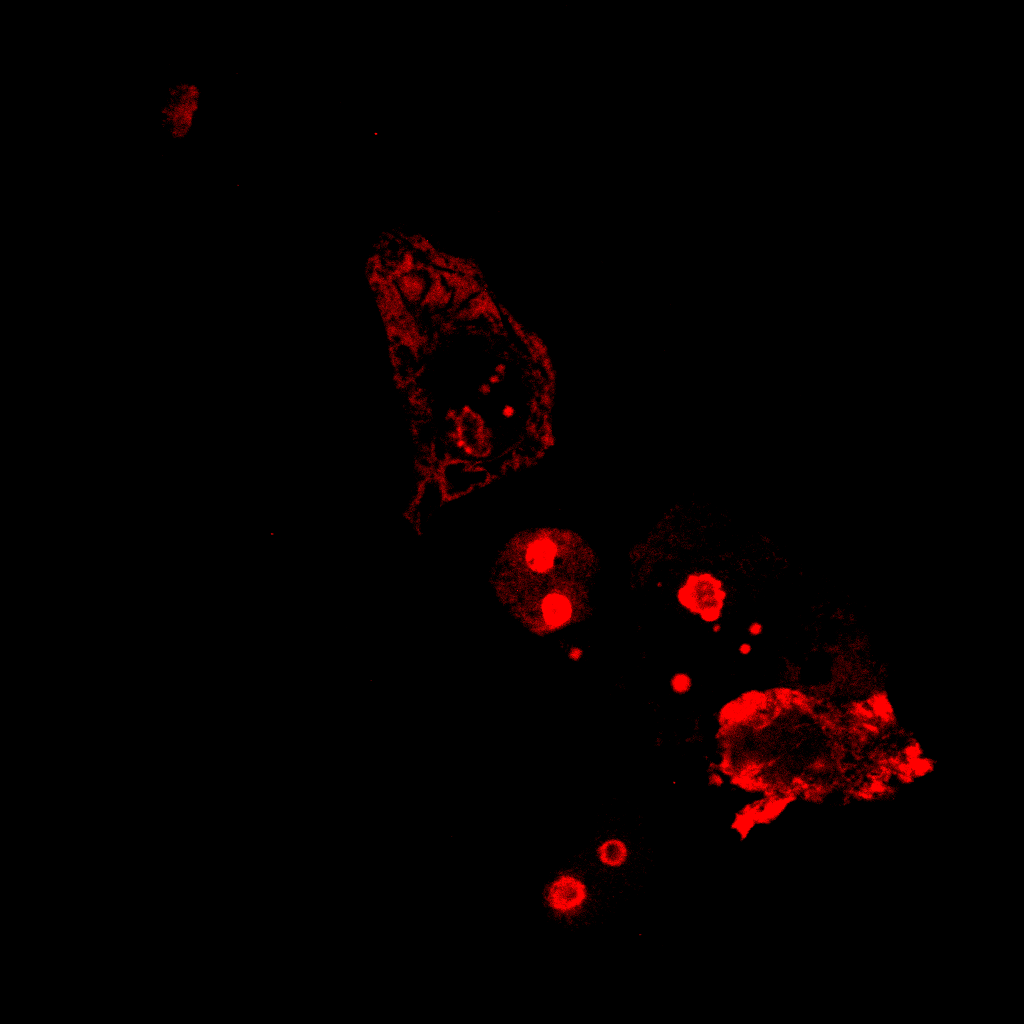

Supplement: S4 Data — (ZIP) [file ppat.1012014.s011.zip › A/A-1/siERK+rAd-Cap NPM1.tif]

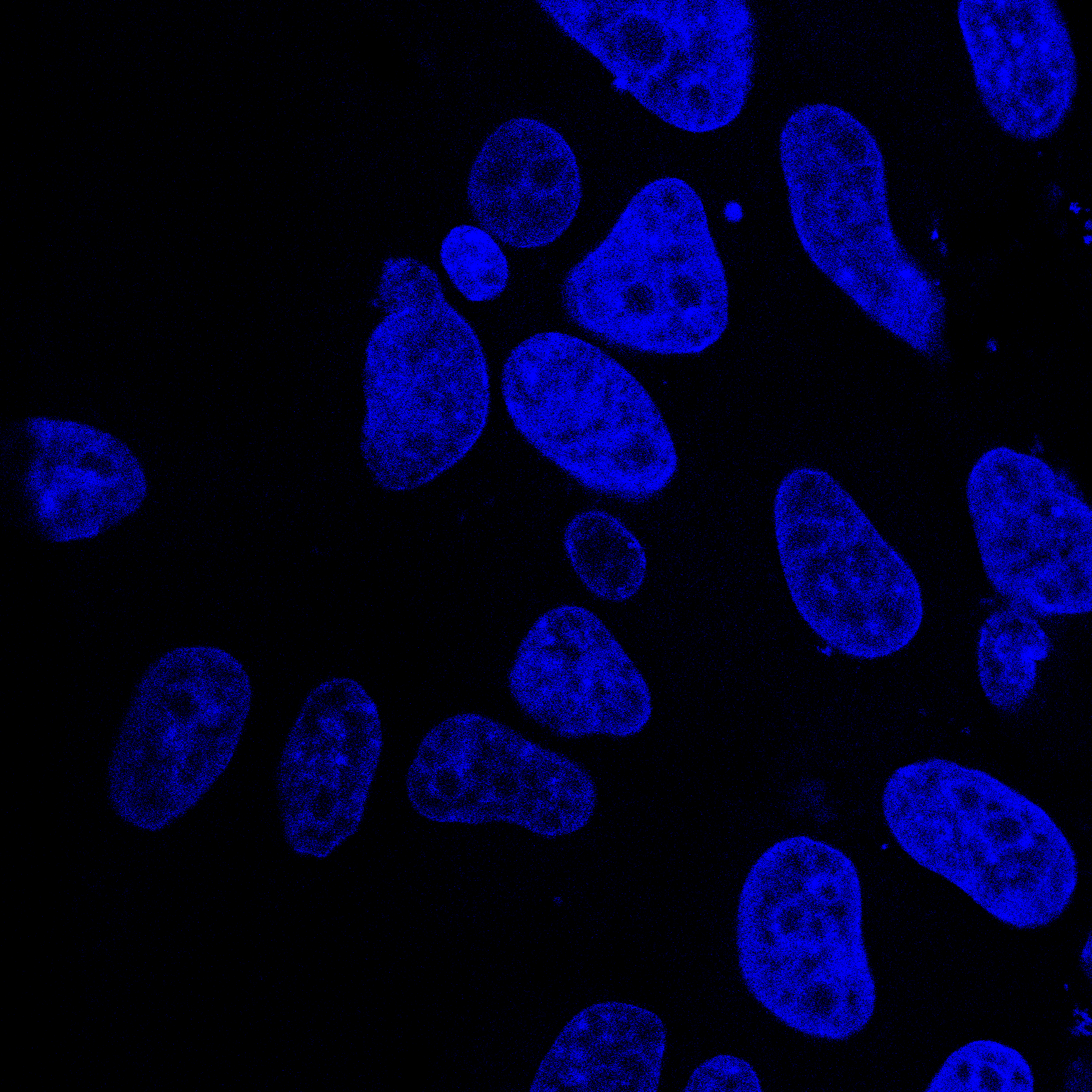

Supplement: S4 Data — (ZIP) [file ppat.1012014.s011.zip › A/A-1/siNC+rAd-Blank DAPI.tif]

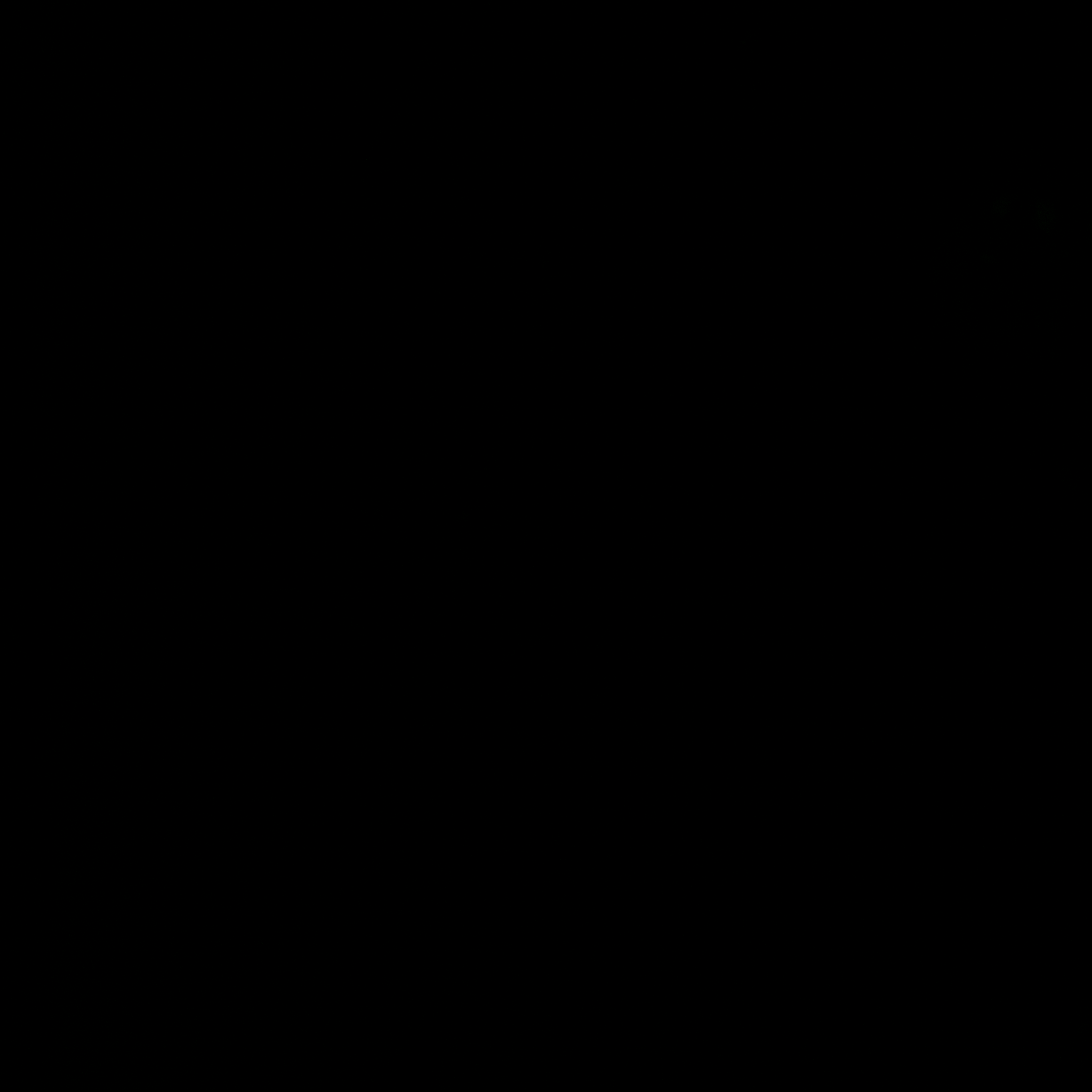

Supplement: S4 Data — (ZIP) [file ppat.1012014.s011.zip › A/A-1/siNC+rAd-Blank Cap.tif]

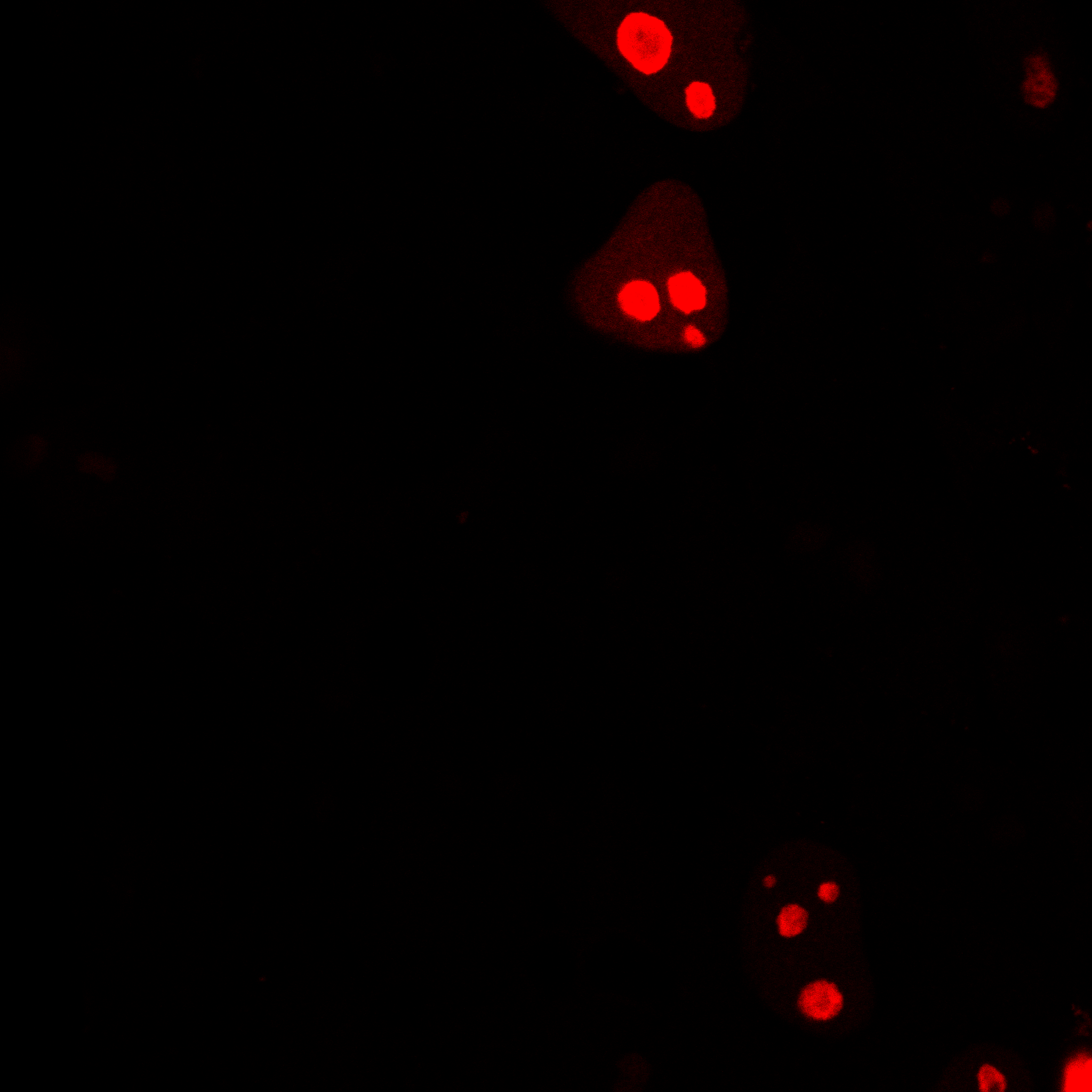

Supplement: S4 Data — (ZIP) [file ppat.1012014.s011.zip › A/A-1/siNC+rAd-Blank NPM1.tif]

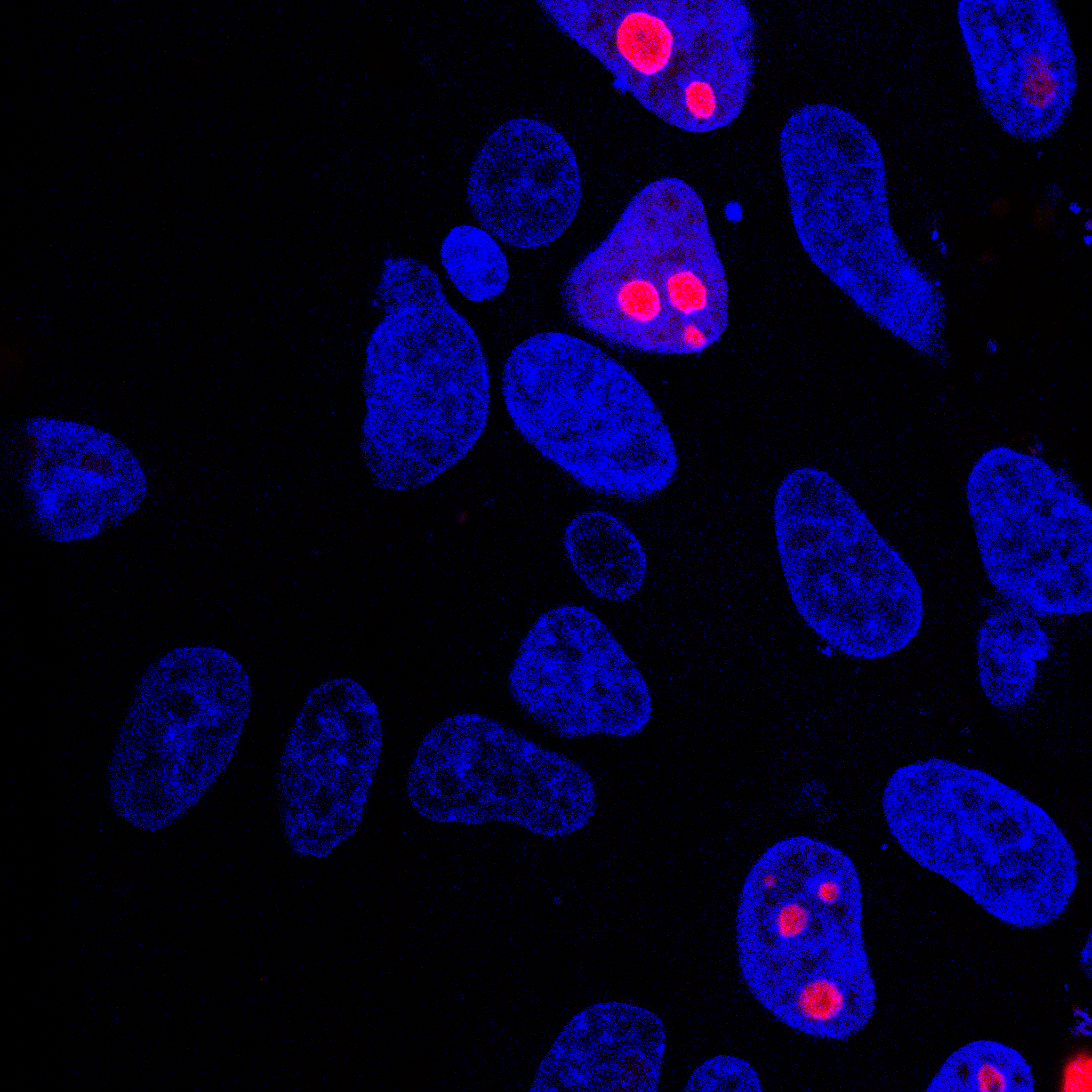

Supplement: S4 Data — (ZIP) [file ppat.1012014.s011.zip › A/A-1/siNC+rAd-BlankMerge.tif]

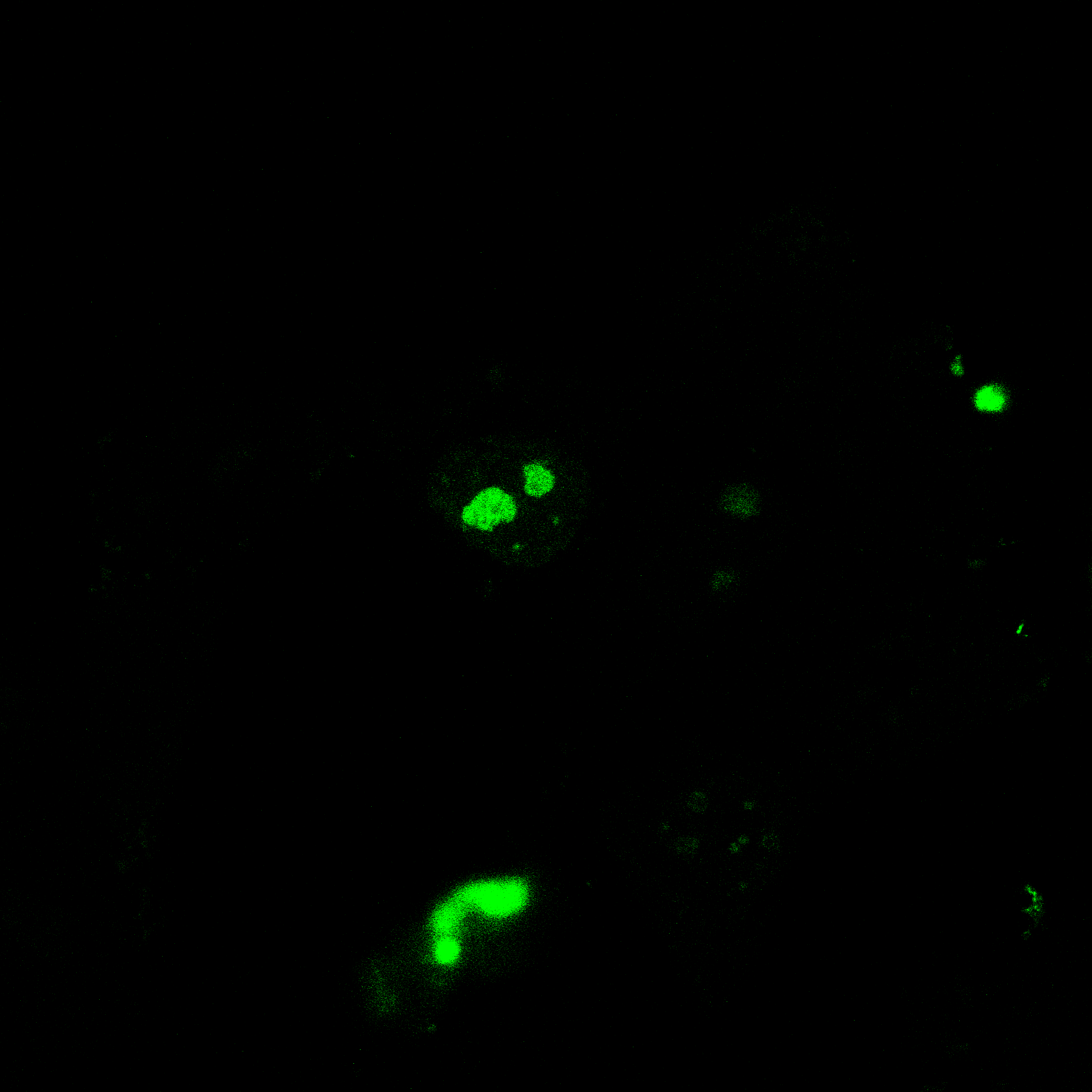

Supplement: S4 Data — (ZIP) [file ppat.1012014.s011.zip › A/A-1/siNC+rAd-Cap Cap.tif]

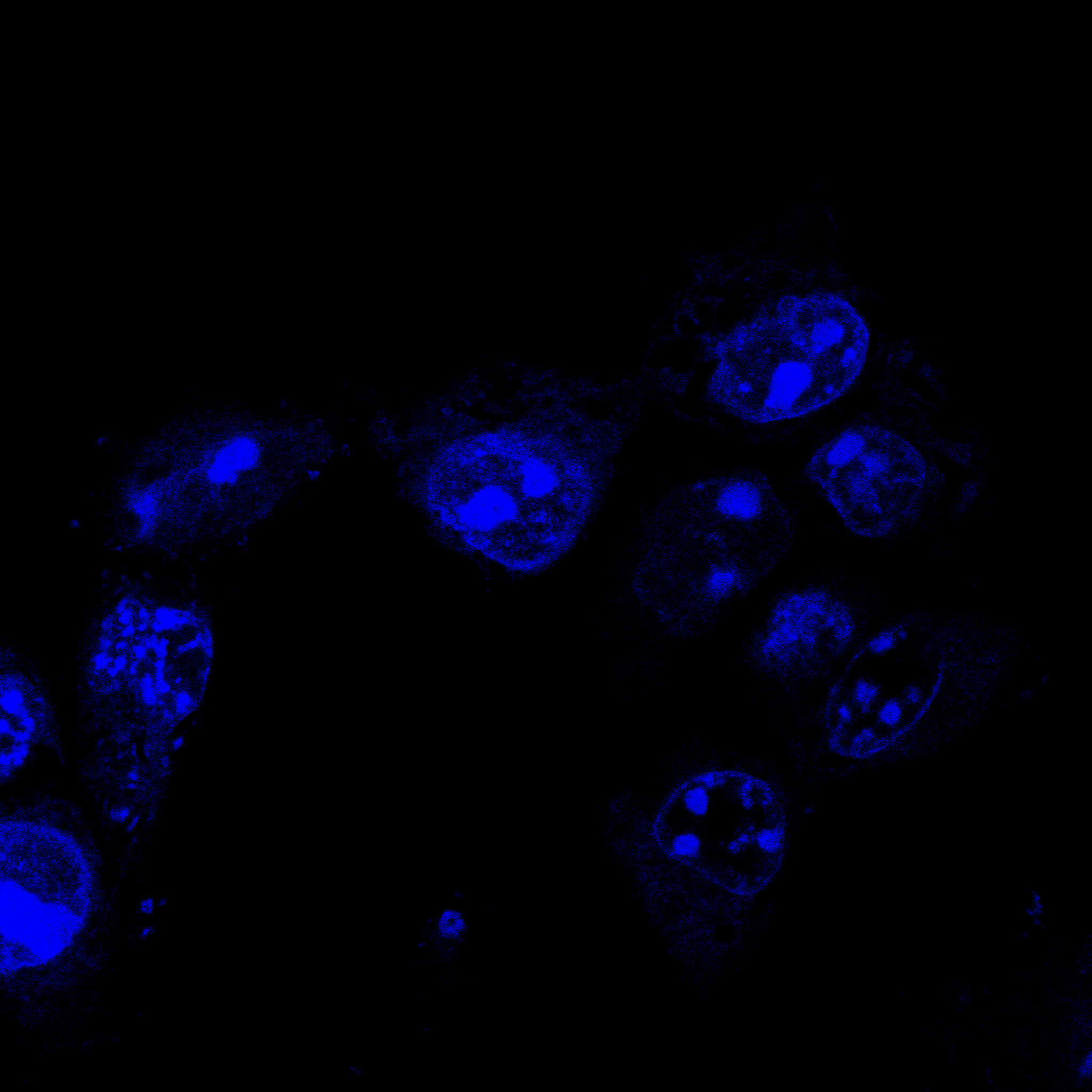

Supplement: S4 Data — (ZIP) [file ppat.1012014.s011.zip › A/A-1/siNC+rAd-Cap DAPI.tif]

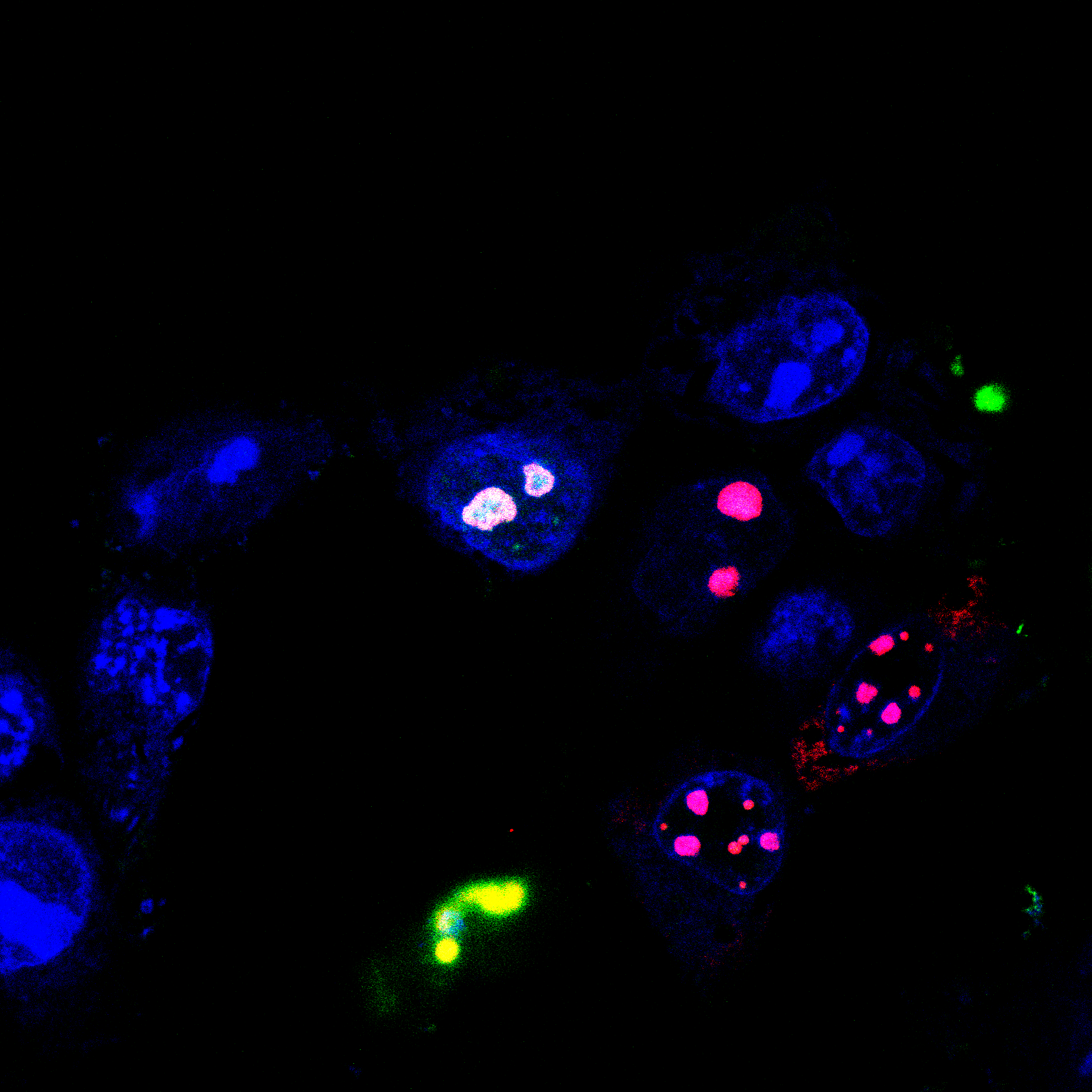

Supplement: S4 Data — (ZIP) [file ppat.1012014.s011.zip › A/A-1/siNC+rAd-Cap Merge.tif]

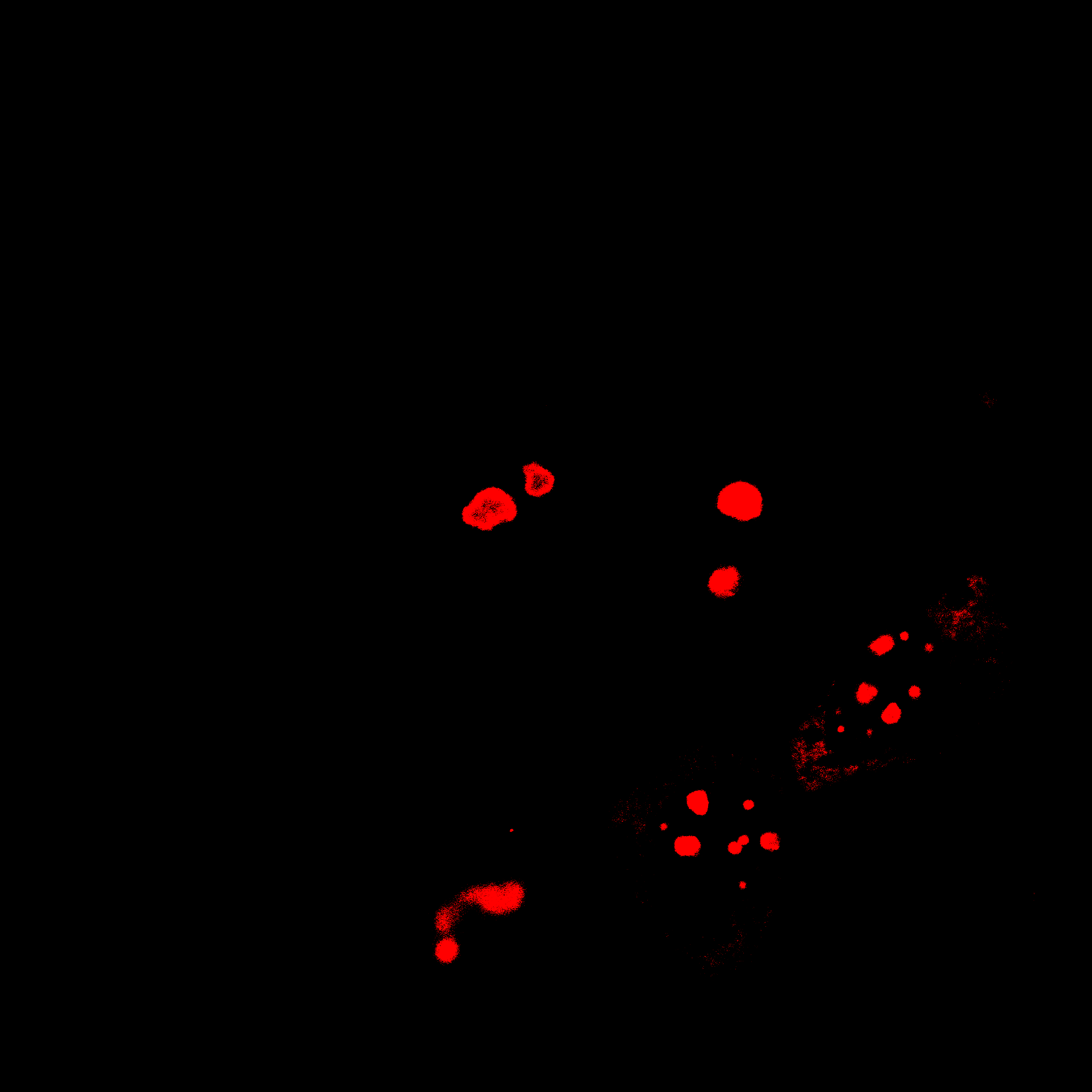

Supplement: S4 Data — (ZIP) [file ppat.1012014.s011.zip › A/A-1/siNC+rAd-Cap NPM1.tif]

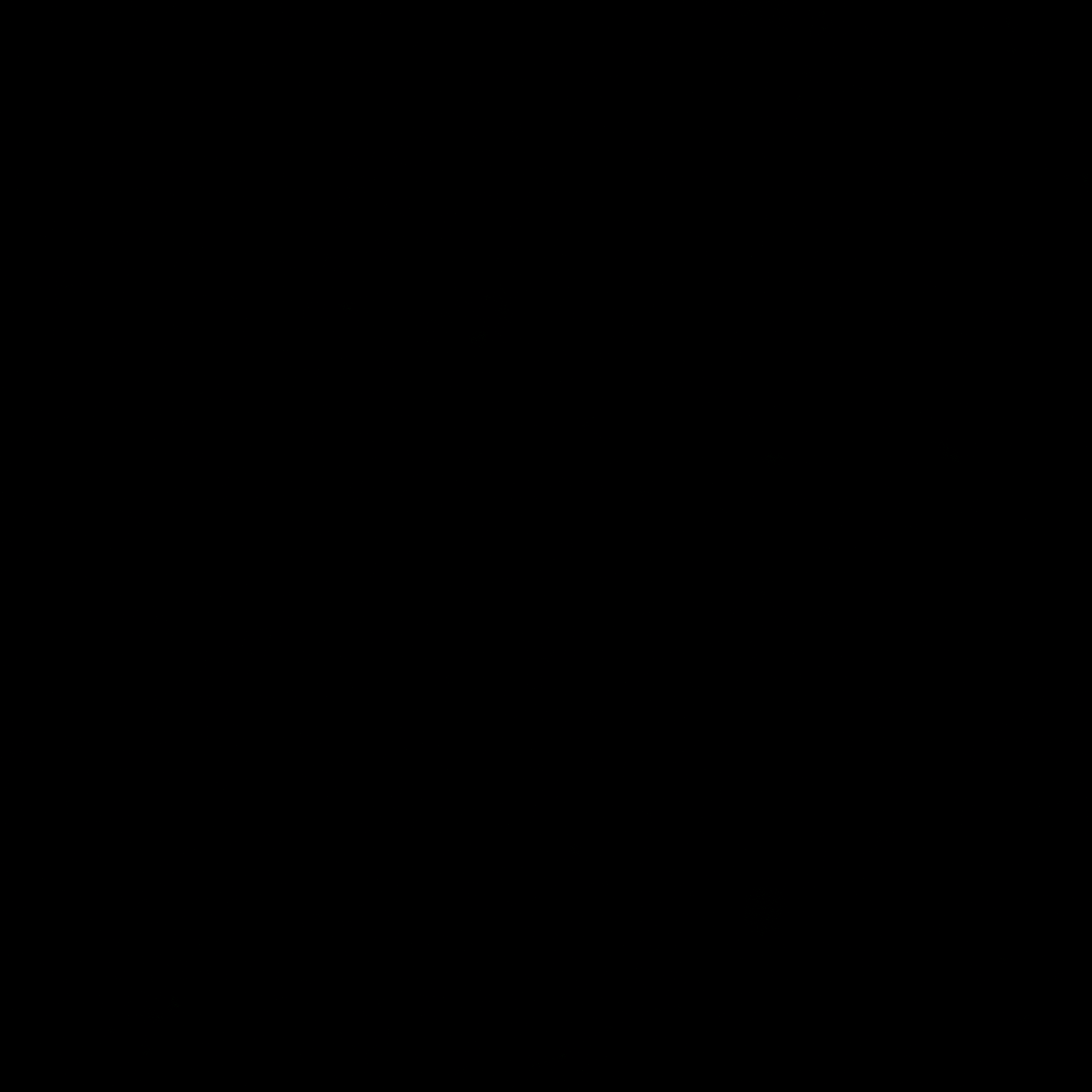

Supplement: S4 Data — (ZIP) [file ppat.1012014.s011.zip › A/A-2/siERK+rAd-Blank Cap.tif]

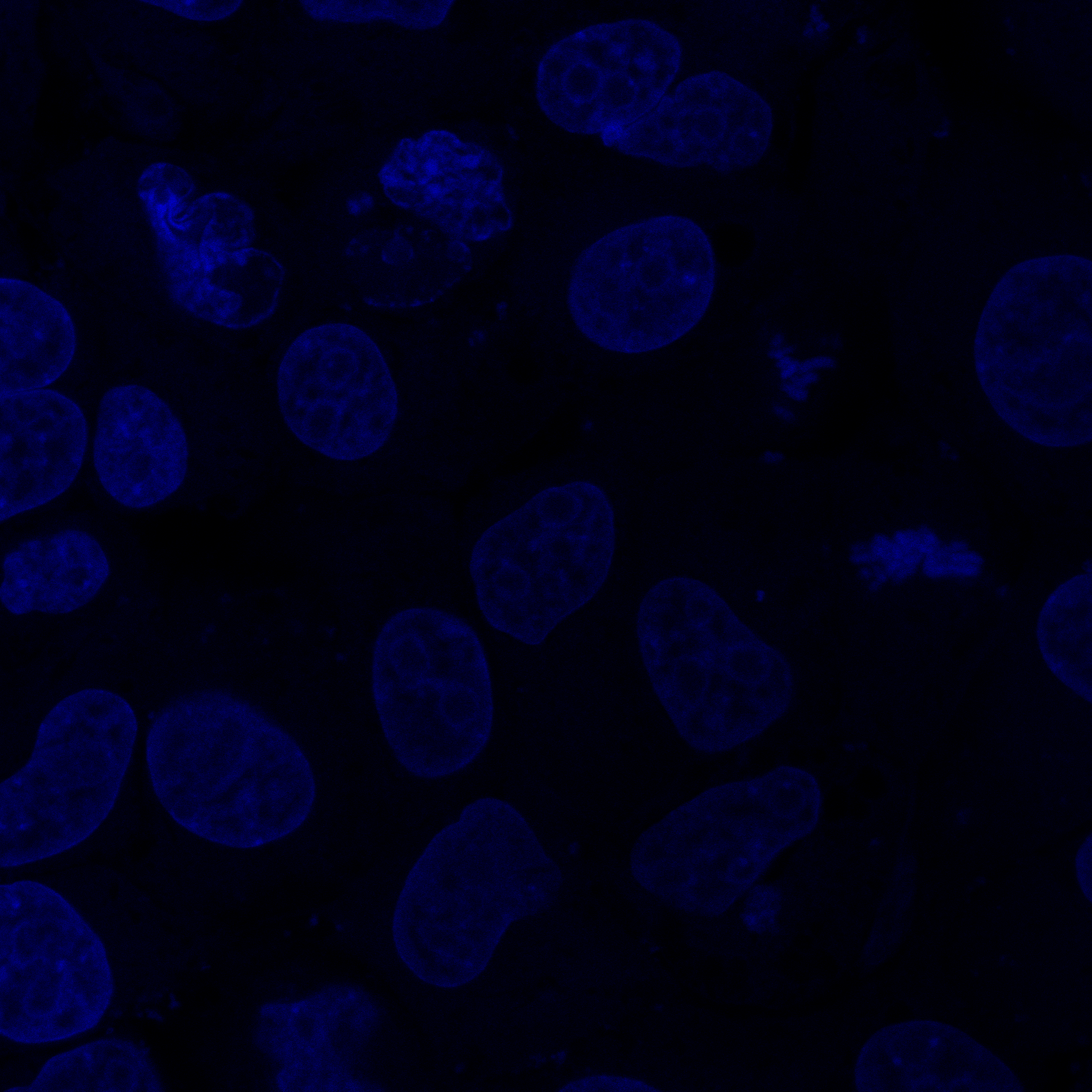

Supplement: S4 Data — (ZIP) [file ppat.1012014.s011.zip › A/A-2/siERK+rAd-Blank DAPI.tif]

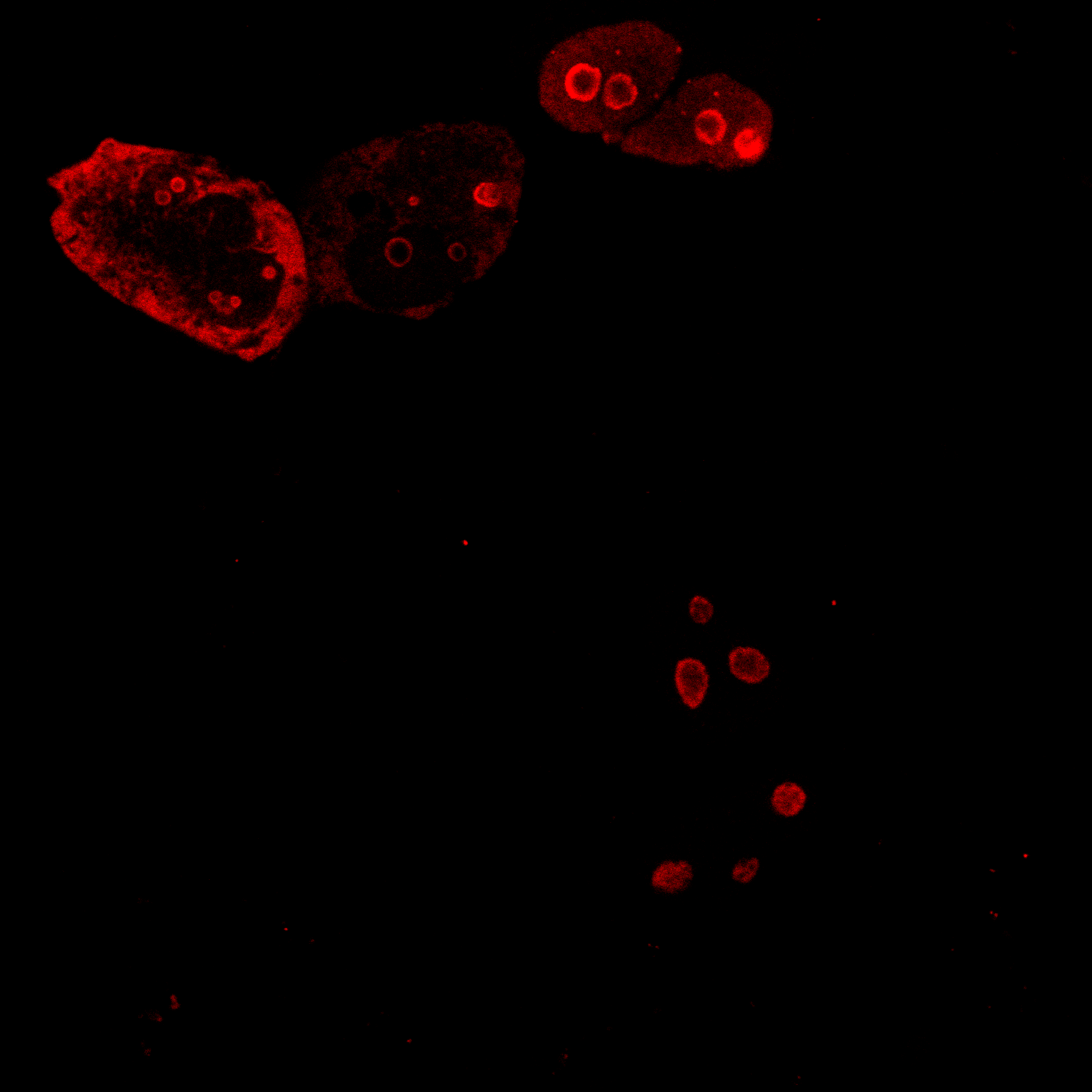

Supplement: S4 Data — (ZIP) [file ppat.1012014.s011.zip › A/A-2/siERK+rAd-Blank NPM1.tif]

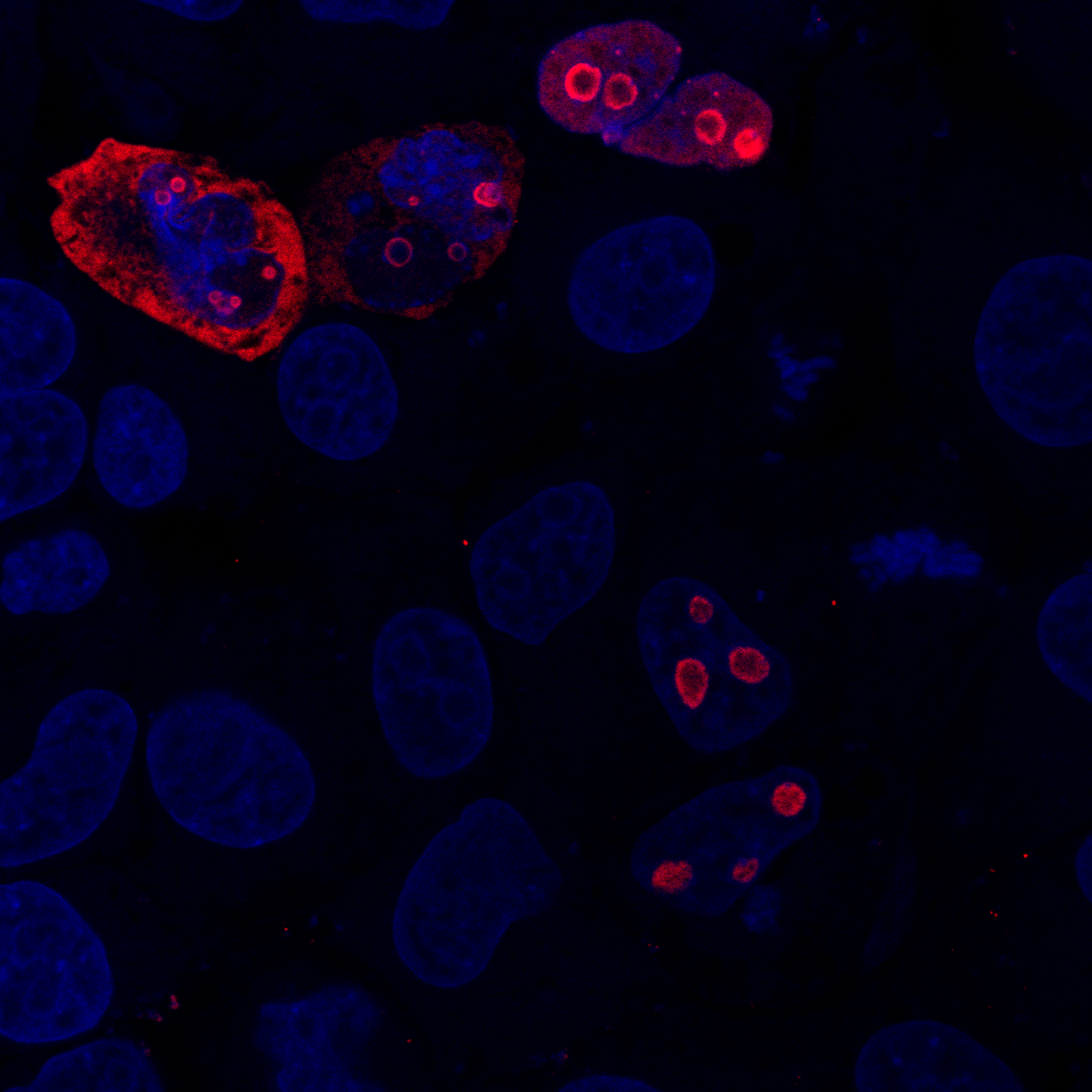

Supplement: S4 Data — (ZIP) [file ppat.1012014.s011.zip › A/A-2/siERK+rAd-Blank Merge.tif]

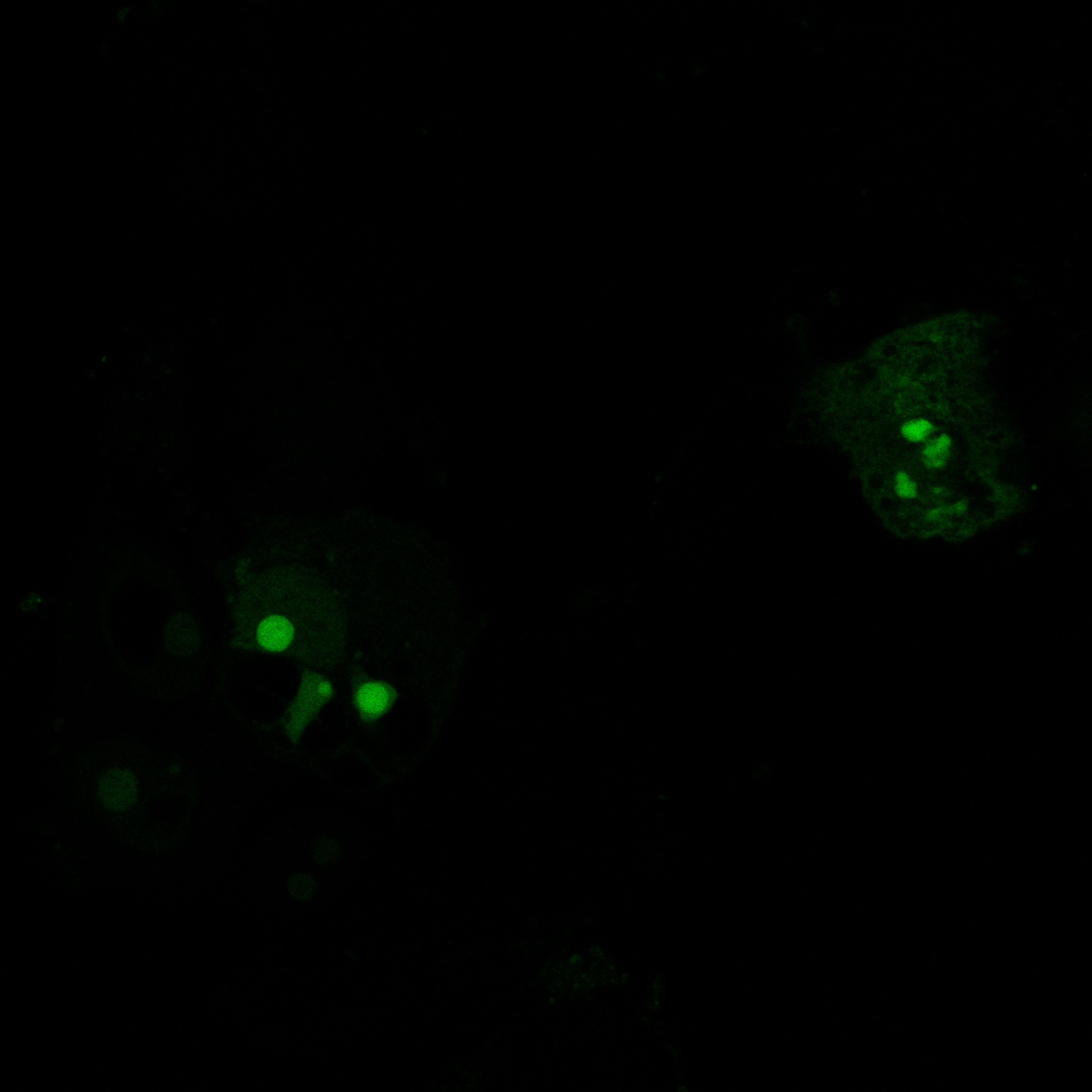

Supplement: S4 Data — (ZIP) [file ppat.1012014.s011.zip › A/A-2/siERK+rAd-Cap Cap.tif]

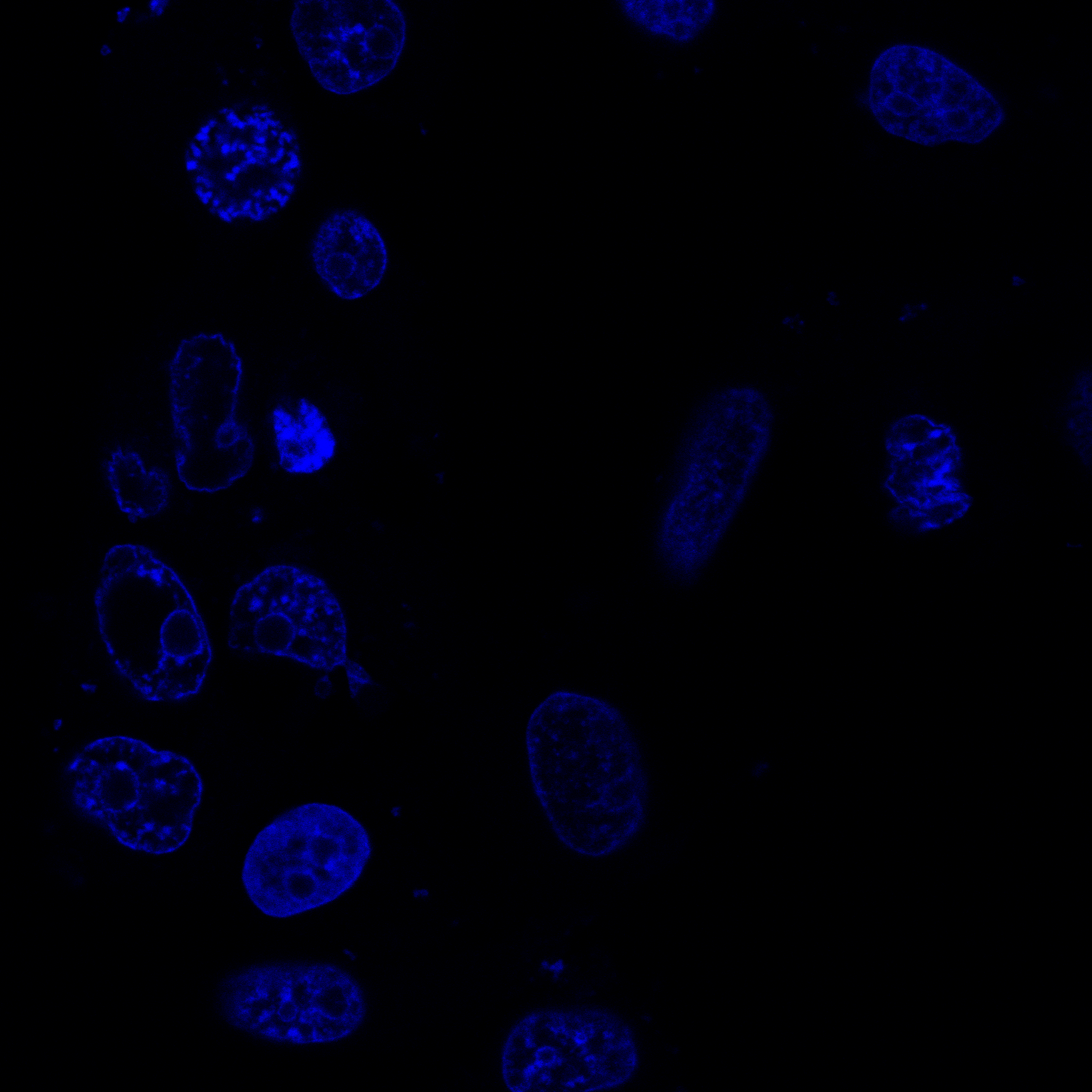

Supplement: S4 Data — (ZIP) [file ppat.1012014.s011.zip › A/A-2/siERK+rAd-Cap DAPI.tif]

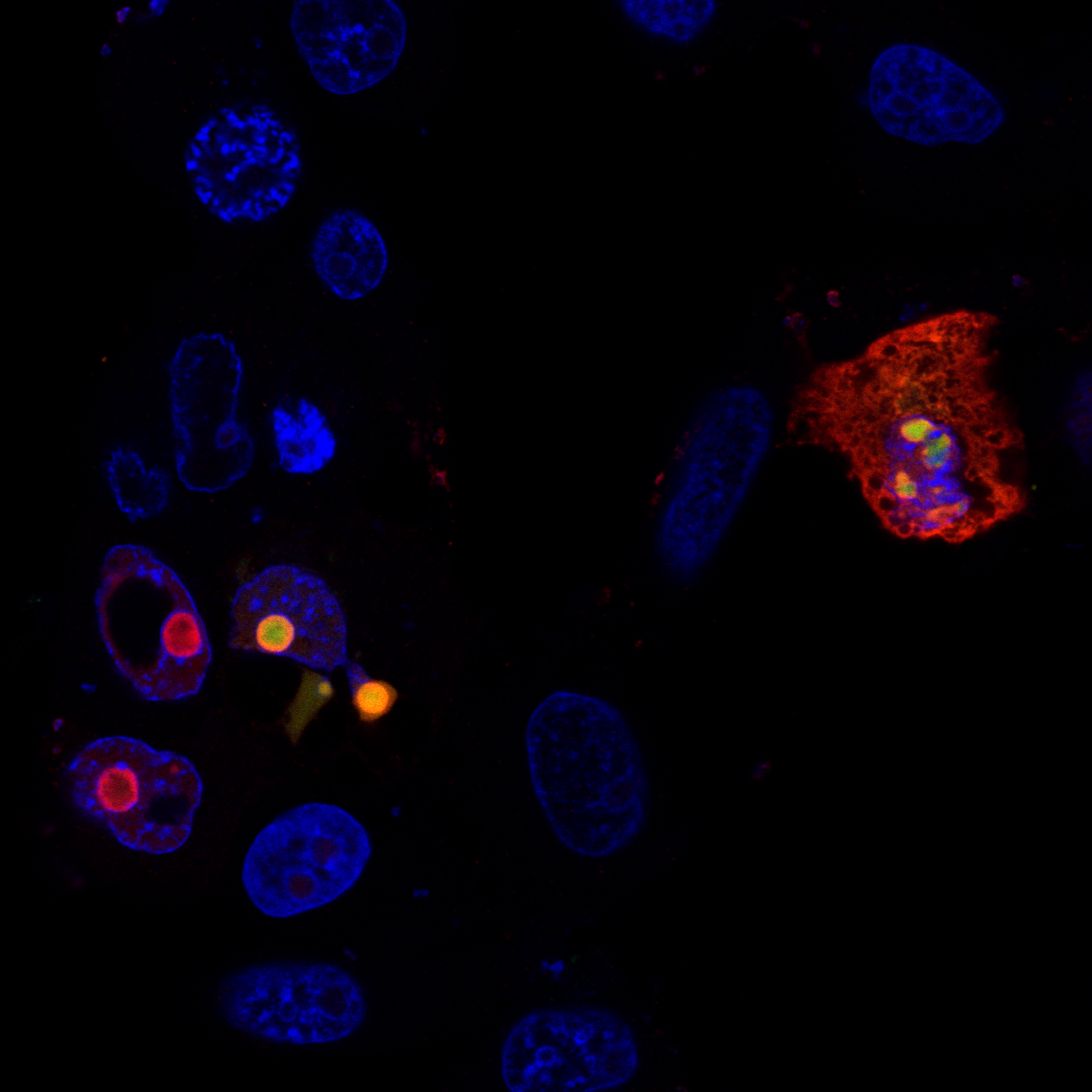

Supplement: S4 Data — (ZIP) [file ppat.1012014.s011.zip › A/A-2/siERK+rAd-Cap Merge.tif]

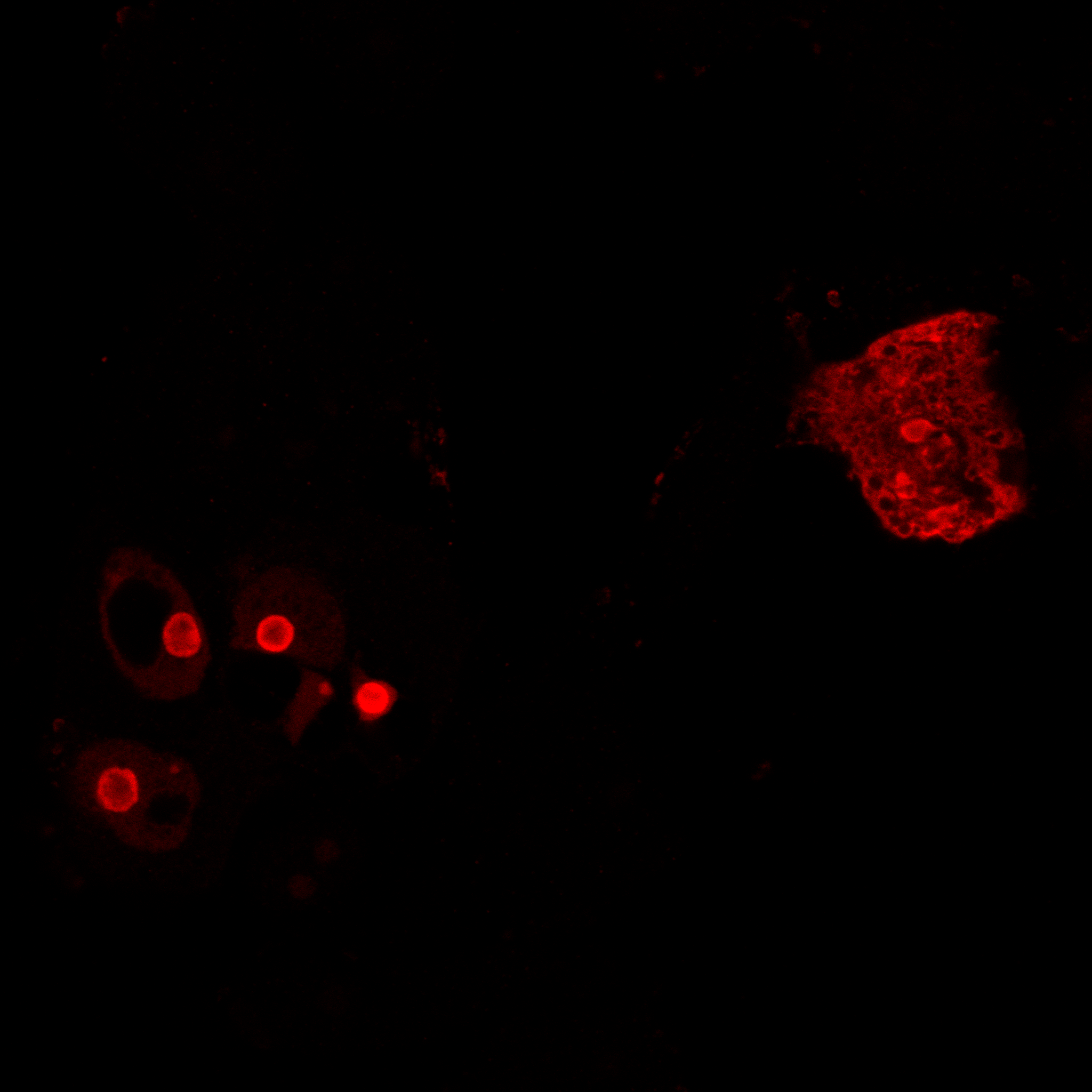

Supplement: S4 Data — (ZIP) [file ppat.1012014.s011.zip › A/A-2/siERK+rAd-Cap NPM1.tif]

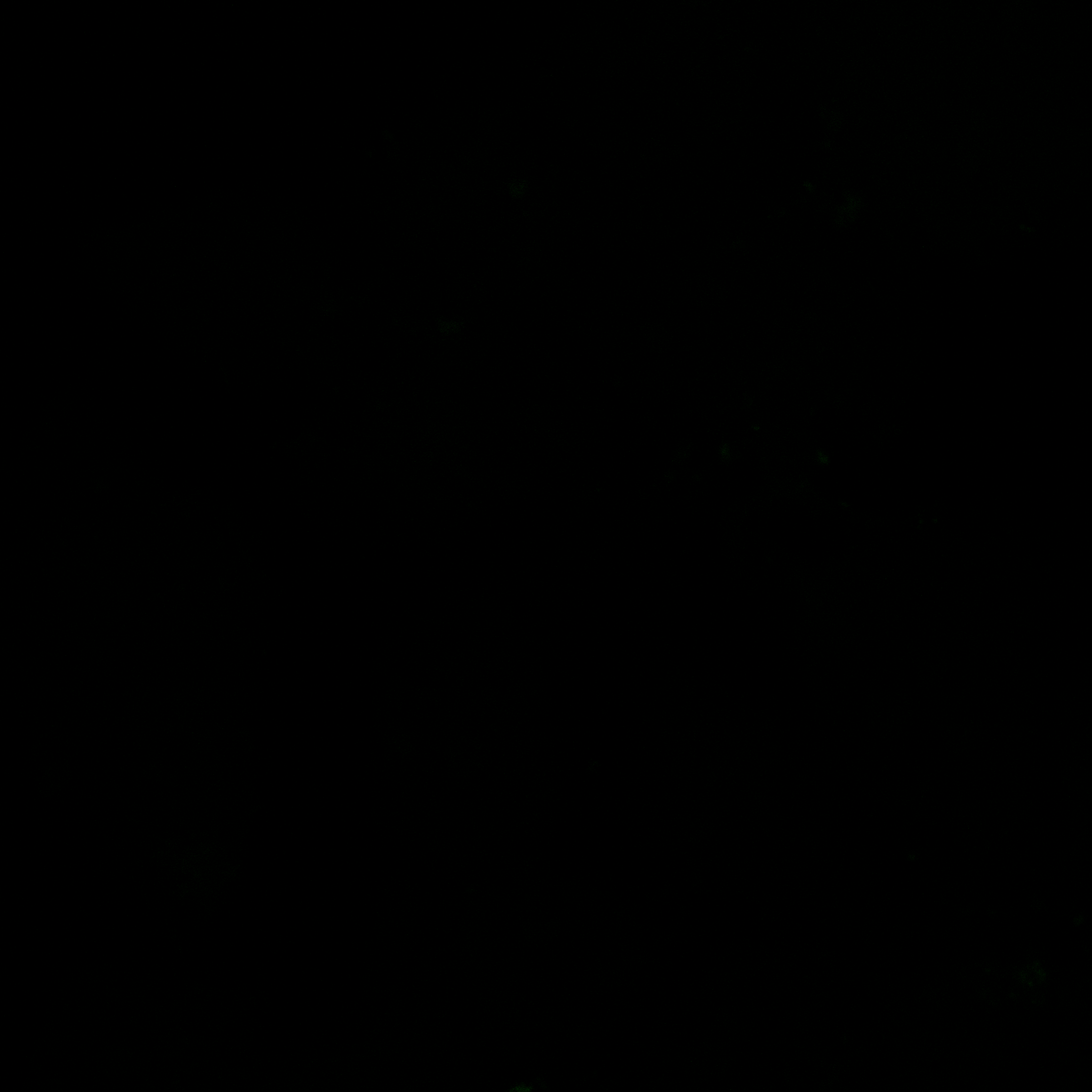

Supplement: S4 Data — (ZIP) [file ppat.1012014.s011.zip › A/A-2/siNC+rAd-Blank Cap.tif]

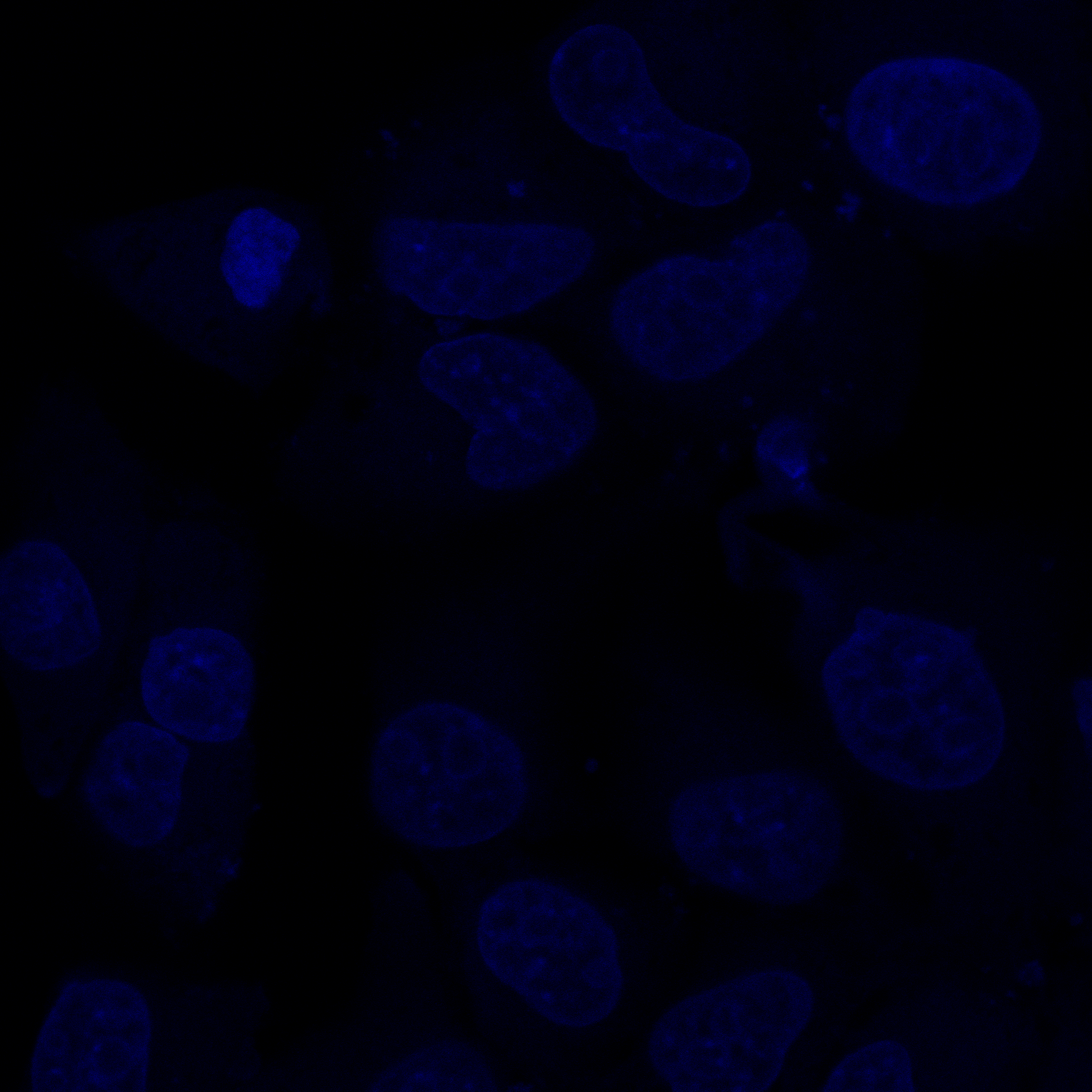

Supplement: S4 Data — (ZIP) [file ppat.1012014.s011.zip › A/A-2/siNC+rAd-Blank DAPI.tif]

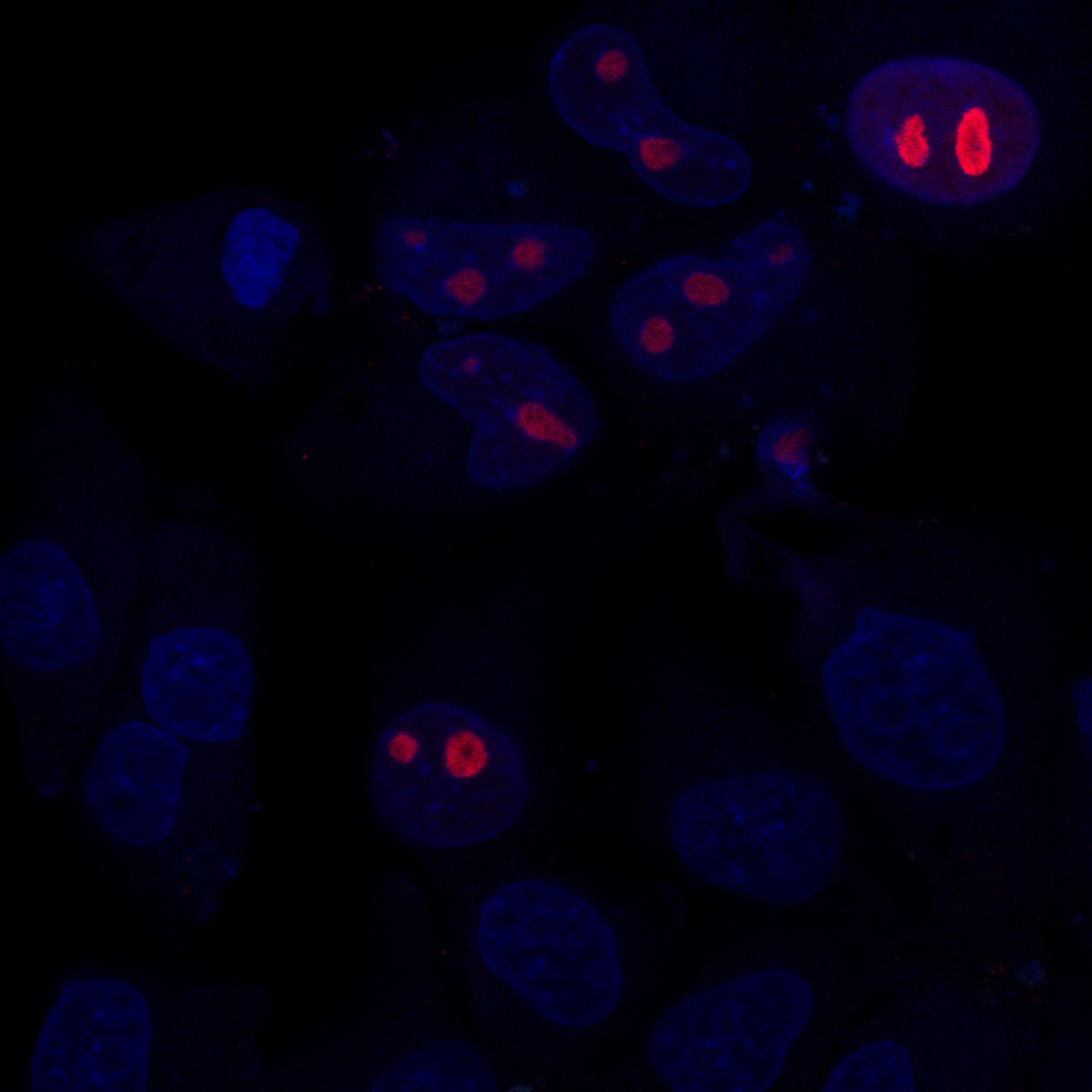

Supplement: S4 Data — (ZIP) [file ppat.1012014.s011.zip › A/A-2/siNC+rAd-Blank Merge.tif]

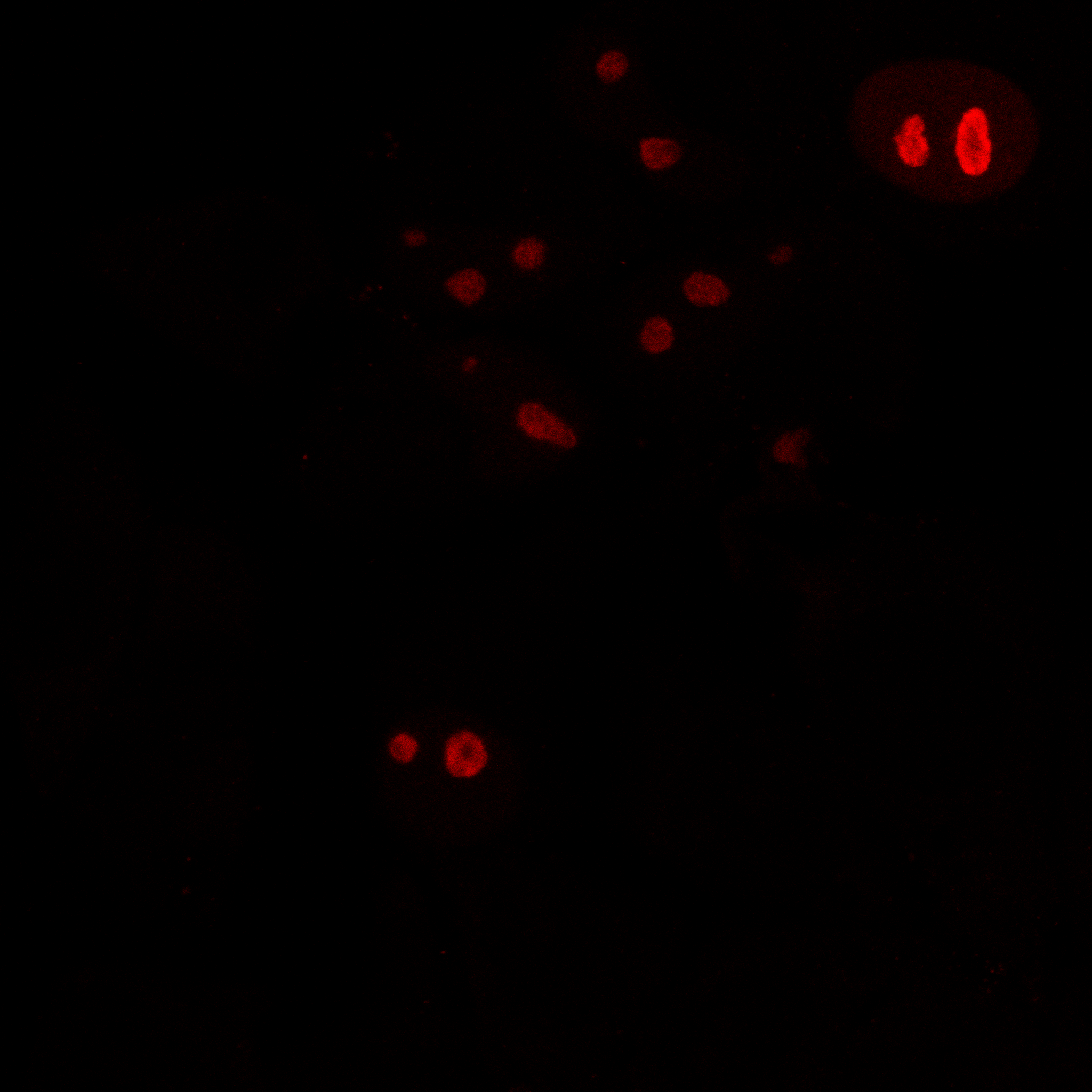

Supplement: S4 Data — (ZIP) [file ppat.1012014.s011.zip › A/A-2/siNC+rAd-Blank NPM1.tif]

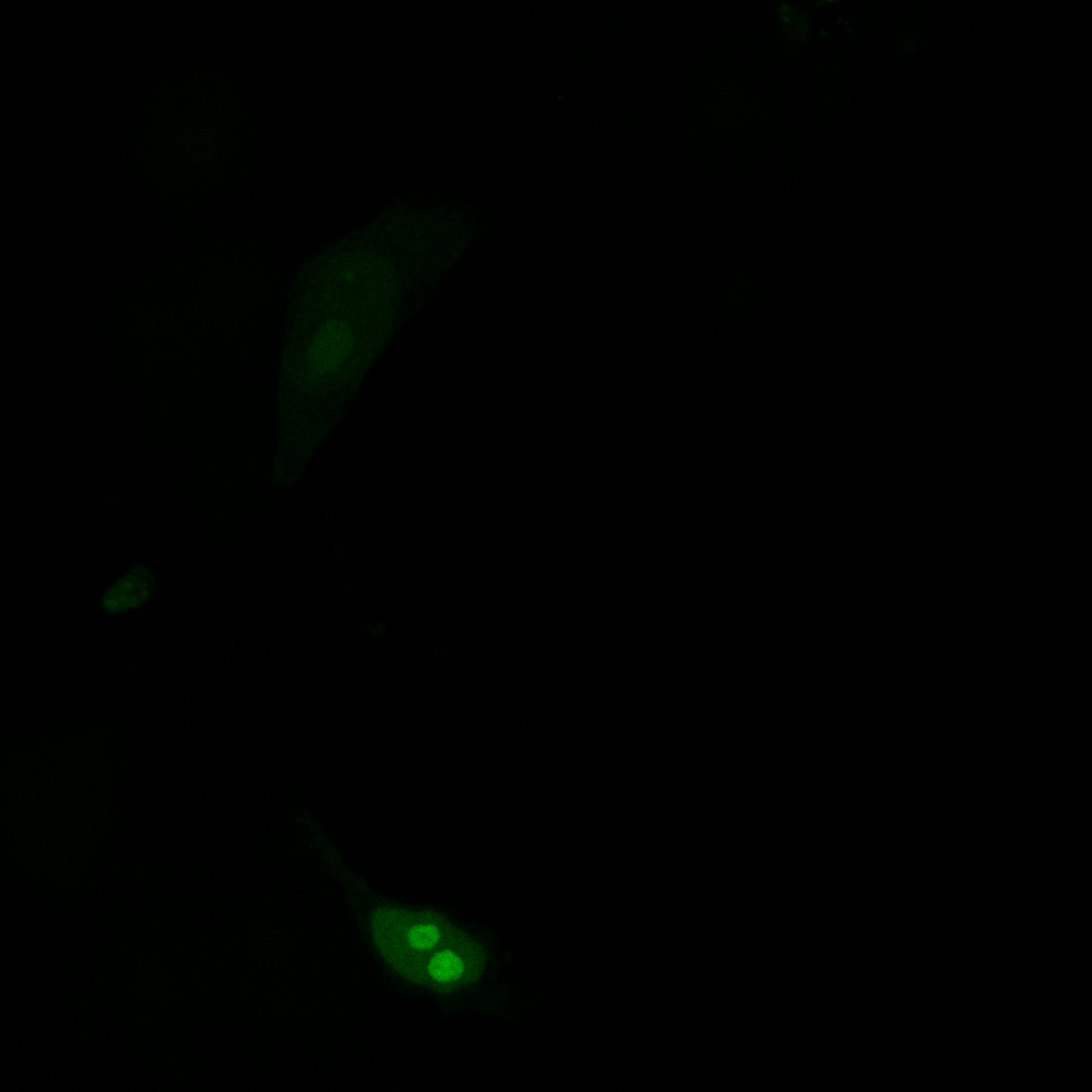

Supplement: S4 Data — (ZIP) [file ppat.1012014.s011.zip › A/A-2/siNC+rAd-Cap Cap.tif]

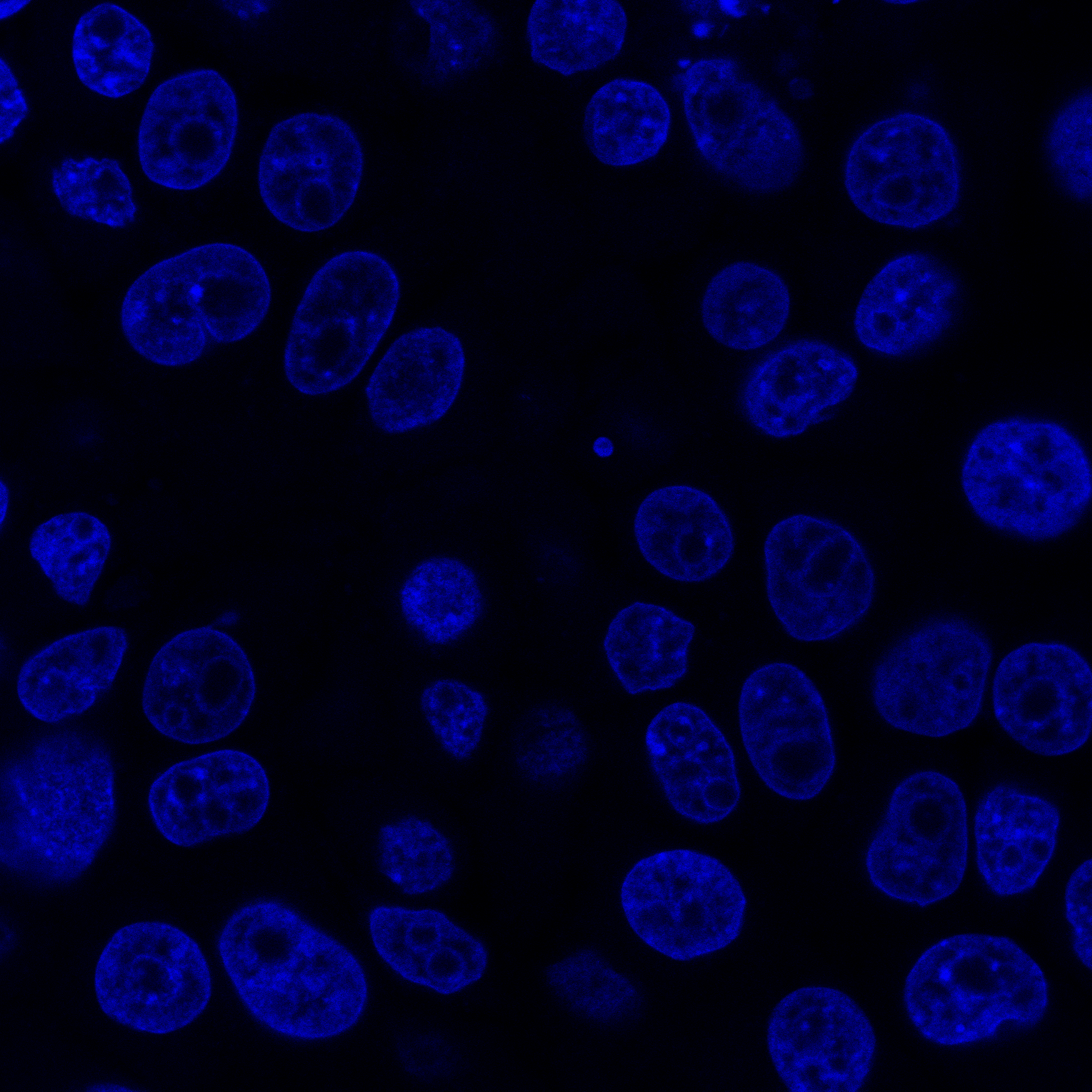

Supplement: S4 Data — (ZIP) [file ppat.1012014.s011.zip › A/A-2/siNC+rAd-Cap DAPI.tif]

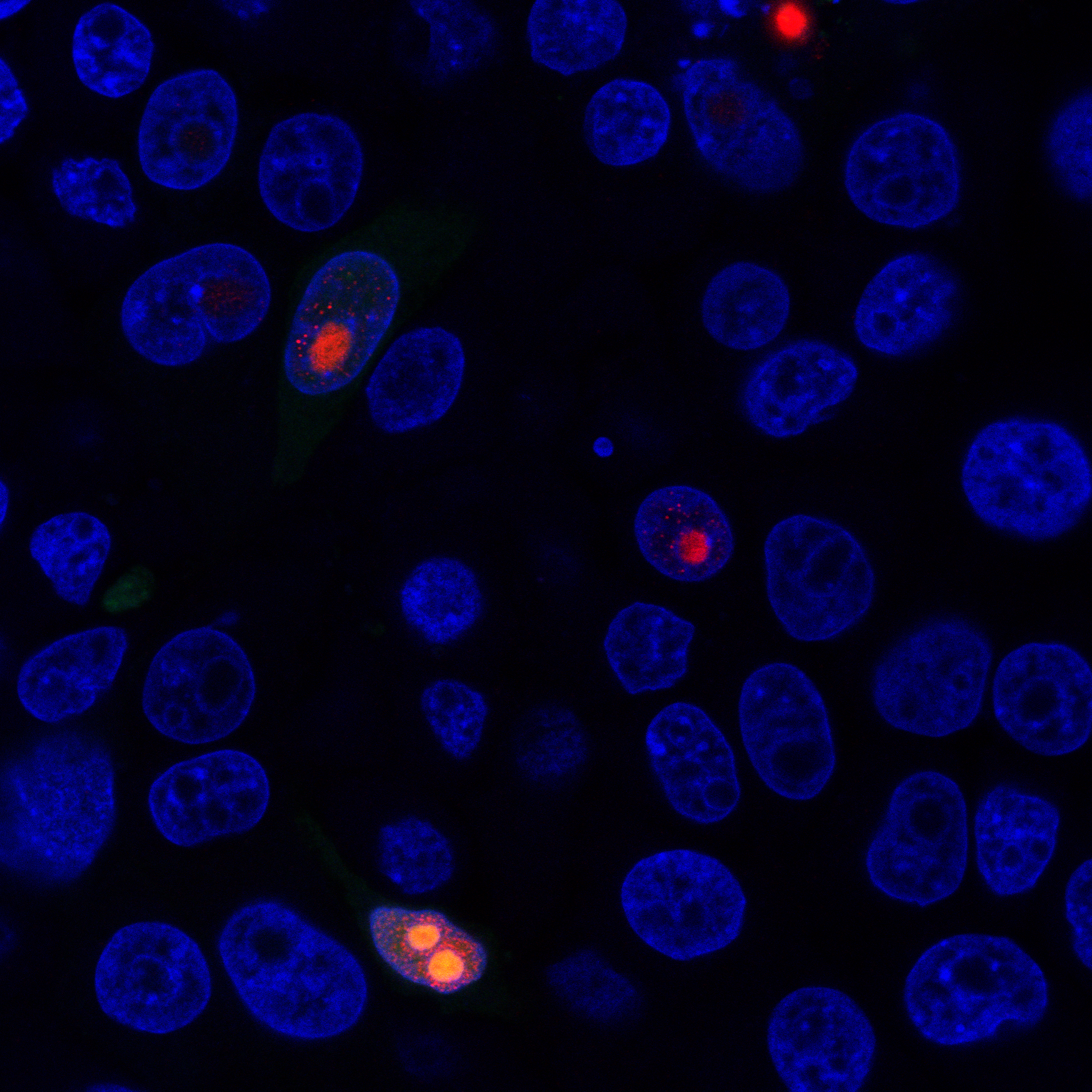

Supplement: S4 Data — (ZIP) [file ppat.1012014.s011.zip › A/A-2/siNC+rAd-Cap Merge.tif]

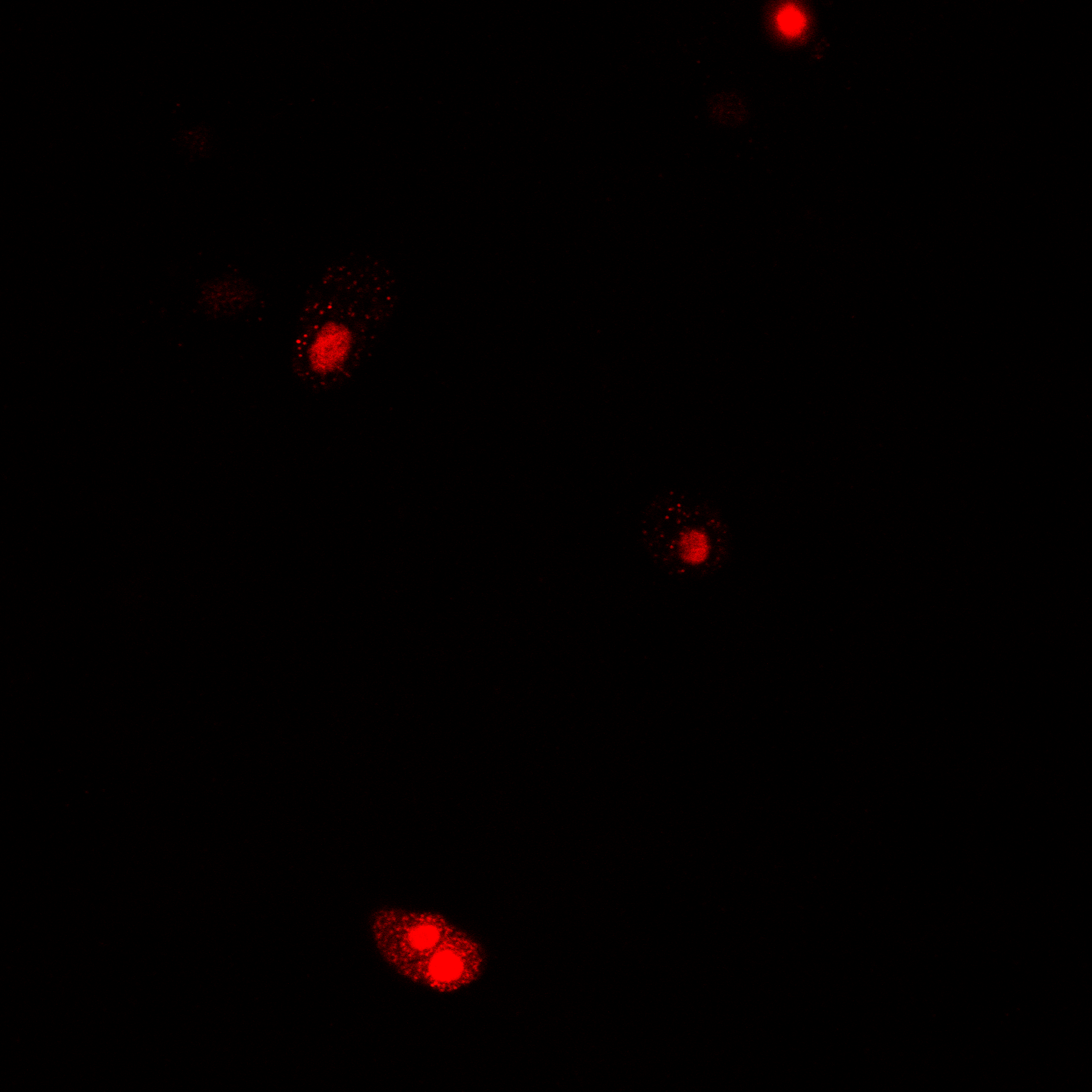

Supplement: S4 Data — (ZIP) [file ppat.1012014.s011.zip › A/A-2/siNC+rAd-Cap NPM1.tif]

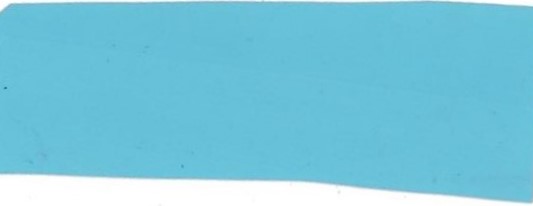

Supplement: S4 Data — (ZIP) [file ppat.1012014.s011.zip › B/1/Cytoplasm-Cap-1.jpg]

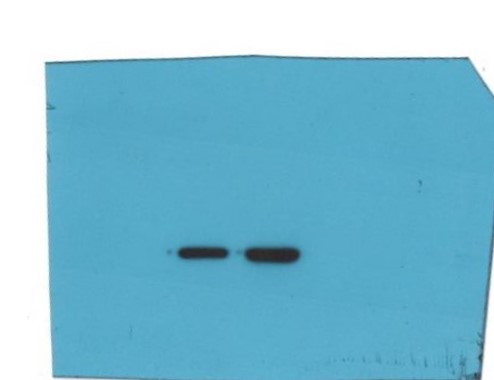

Supplement: S4 Data — (ZIP) [file ppat.1012014.s011.zip › B/1/Cytoplasm-NPM1-1.jpg]

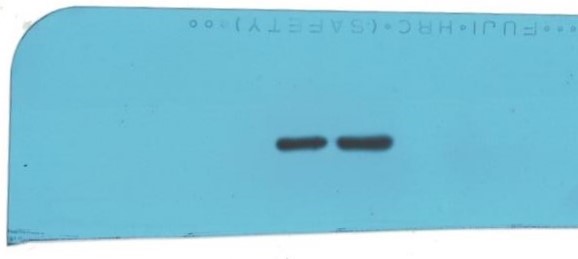

Supplement: S4 Data — (ZIP) [file ppat.1012014.s011.zip › B/1/Cytoplasm-β-actin-1.jpg]

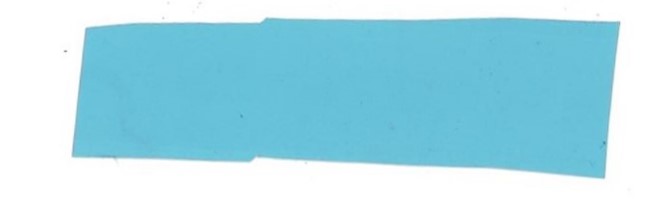

Supplement: S4 Data — (ZIP) [file ppat.1012014.s011.zip › B/1/Nuclear-Cap-1.jpg]

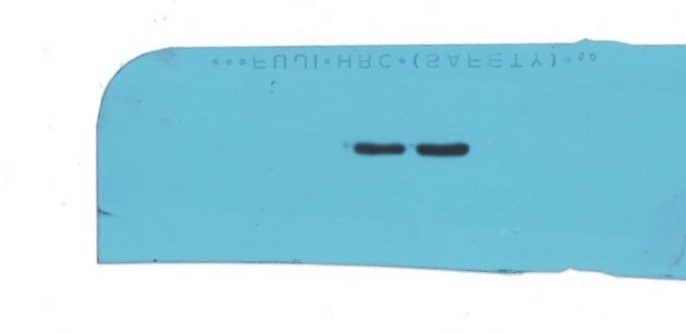

Supplement: S4 Data — (ZIP) [file ppat.1012014.s011.zip › B/1/Nuclear-Lamin B-1.jpg]

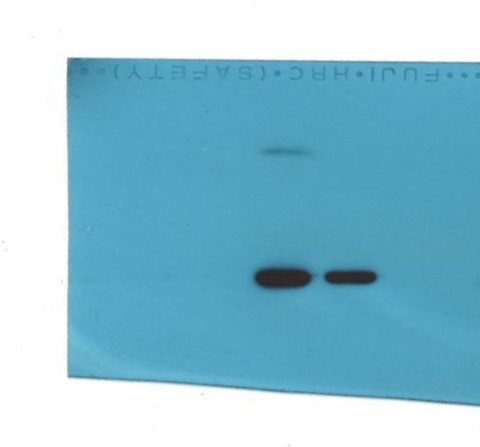

Supplement: S4 Data — (ZIP) [file ppat.1012014.s011.zip › B/1/Nuclear-NPM1-1.jpg]

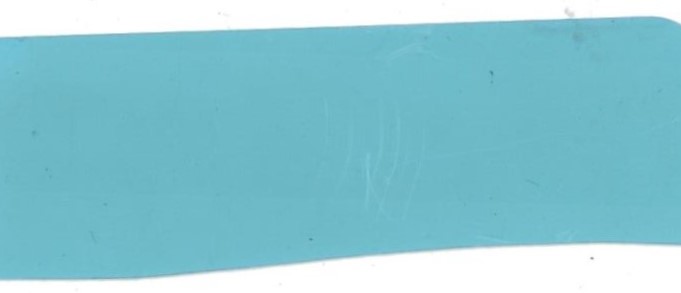

Supplement: S4 Data — (ZIP) [file ppat.1012014.s011.zip › B/2/Cap(Cytoplasm).jpg]

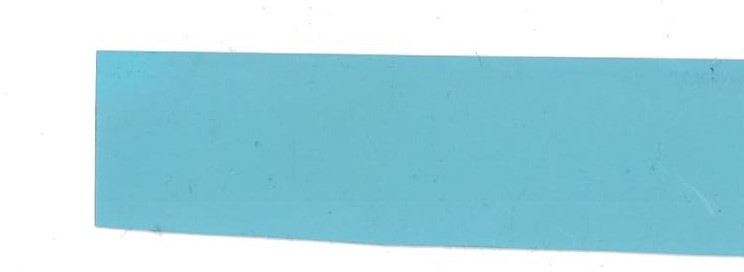

Supplement: S4 Data — (ZIP) [file ppat.1012014.s011.zip › B/2/Cap(Nuclear).jpg]

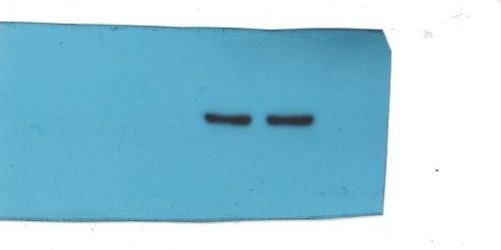

Supplement: S4 Data — (ZIP) [file ppat.1012014.s011.zip › B/2/Lamin B.jpg]

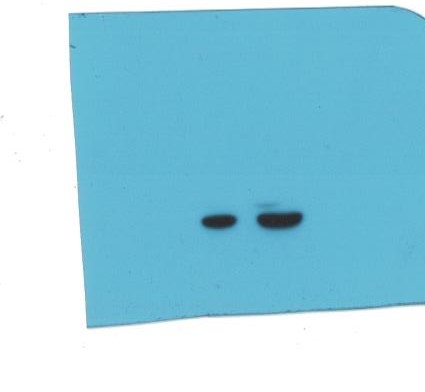

Supplement: S4 Data — (ZIP) [file ppat.1012014.s011.zip › B/2/NPM1-Cytoplasm.jpg]

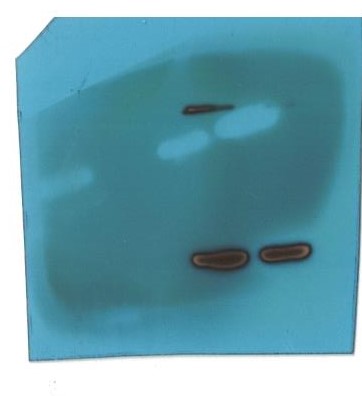

Supplement: S4 Data — (ZIP) [file ppat.1012014.s011.zip › B/2/NPM1-Nuclear.jpeg]

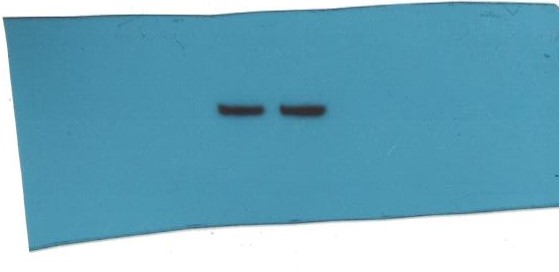

Supplement: S4 Data — (ZIP) [file ppat.1012014.s011.zip › B/2/β-actin.jpg]

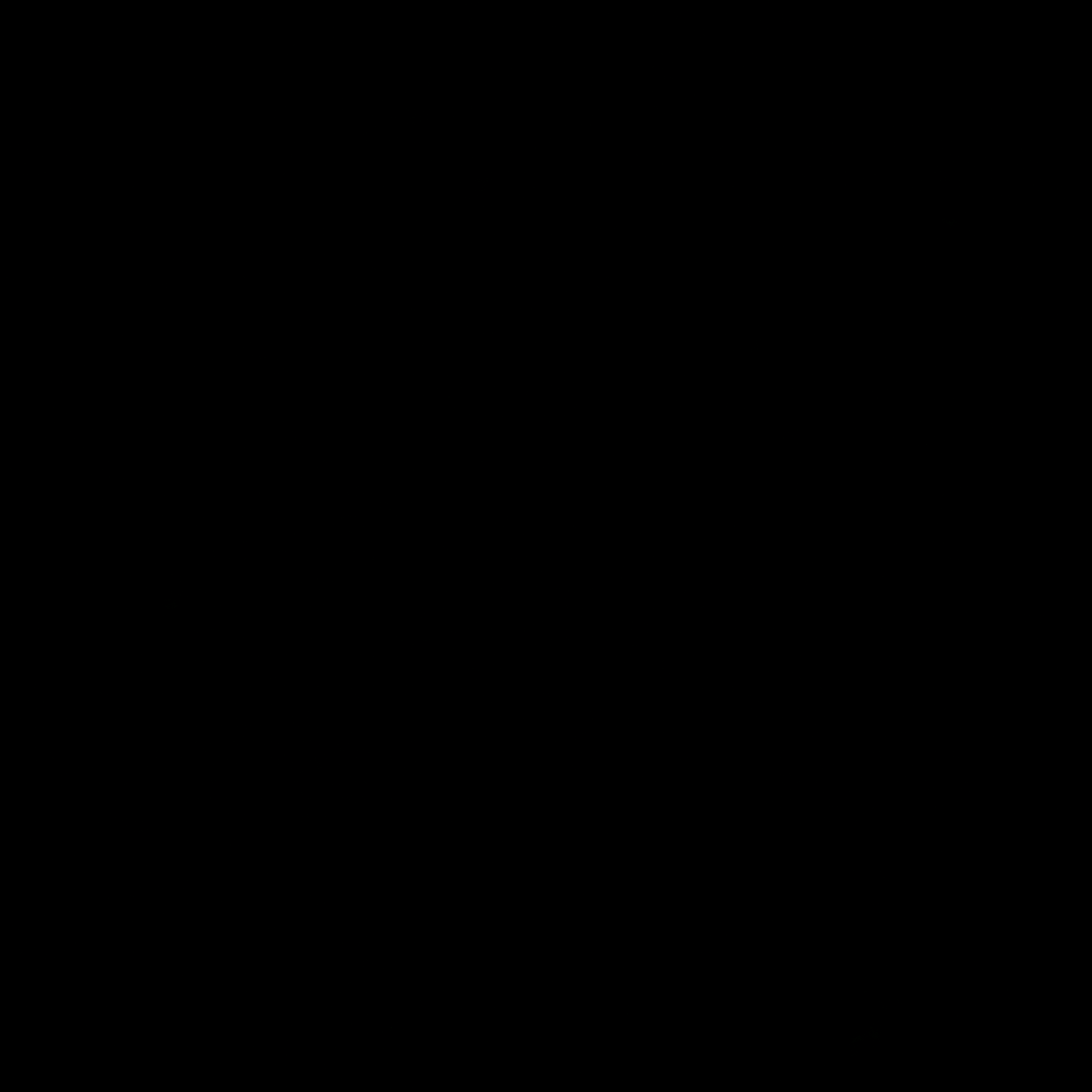

Supplement: S5 Data — (ZIP) [file ppat.1012014.s012.zip › C/C-1/siERK+Mock Cap.tif]

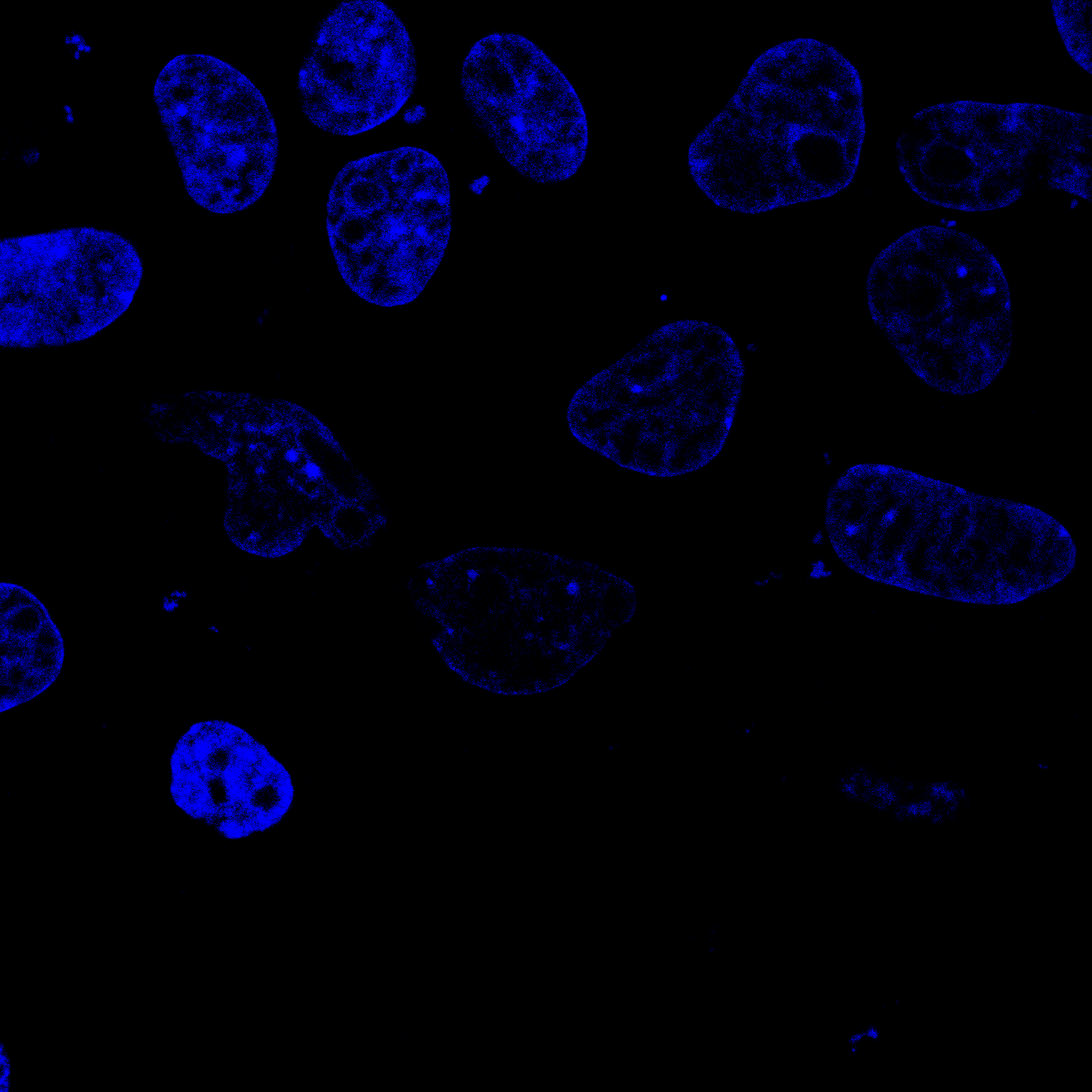

Supplement: S5 Data — (ZIP) [file ppat.1012014.s012.zip › C/C-1/siERK+Mock DAPI.tif]

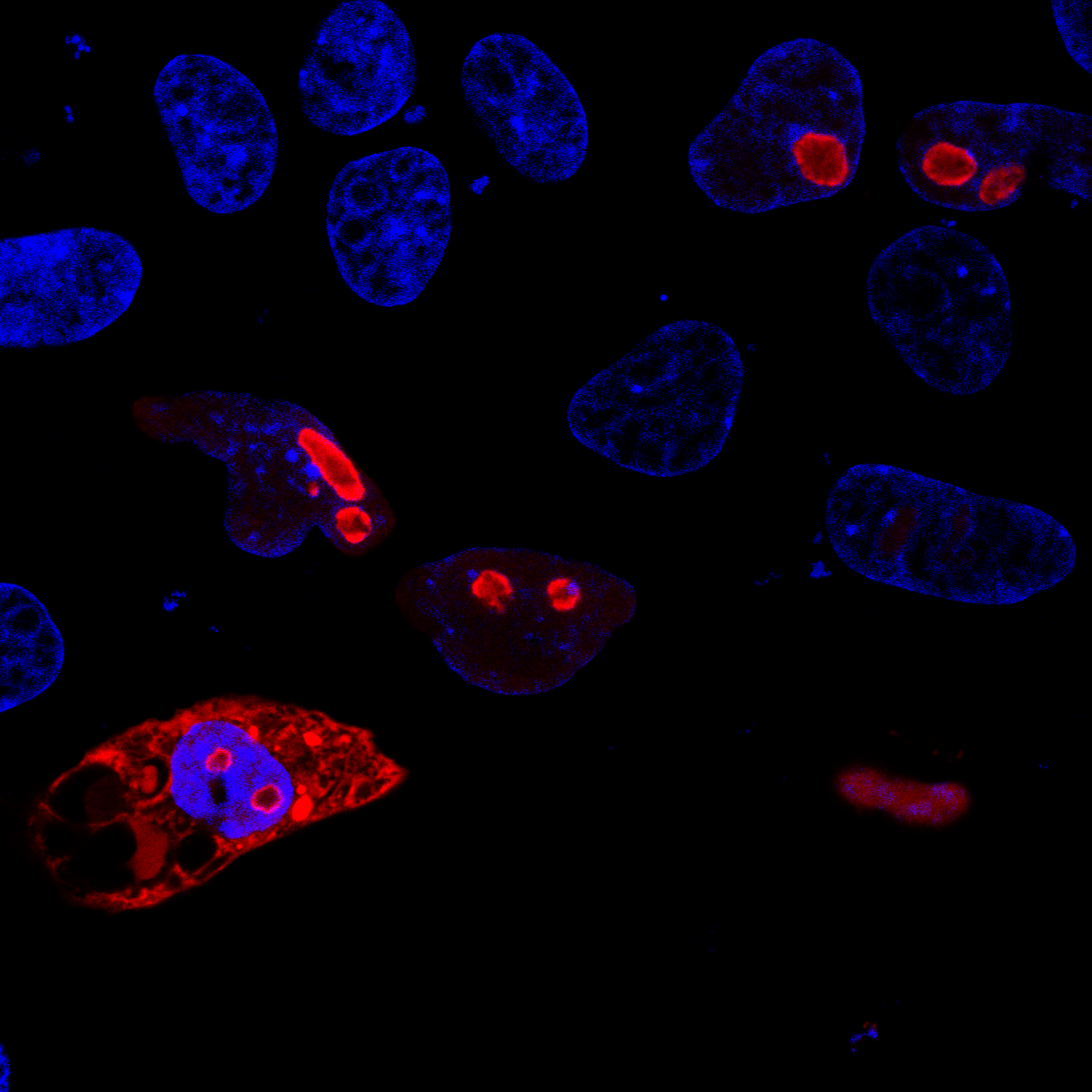

Supplement: S5 Data — (ZIP) [file ppat.1012014.s012.zip › C/C-1/siERK+Mock Merge.tif]

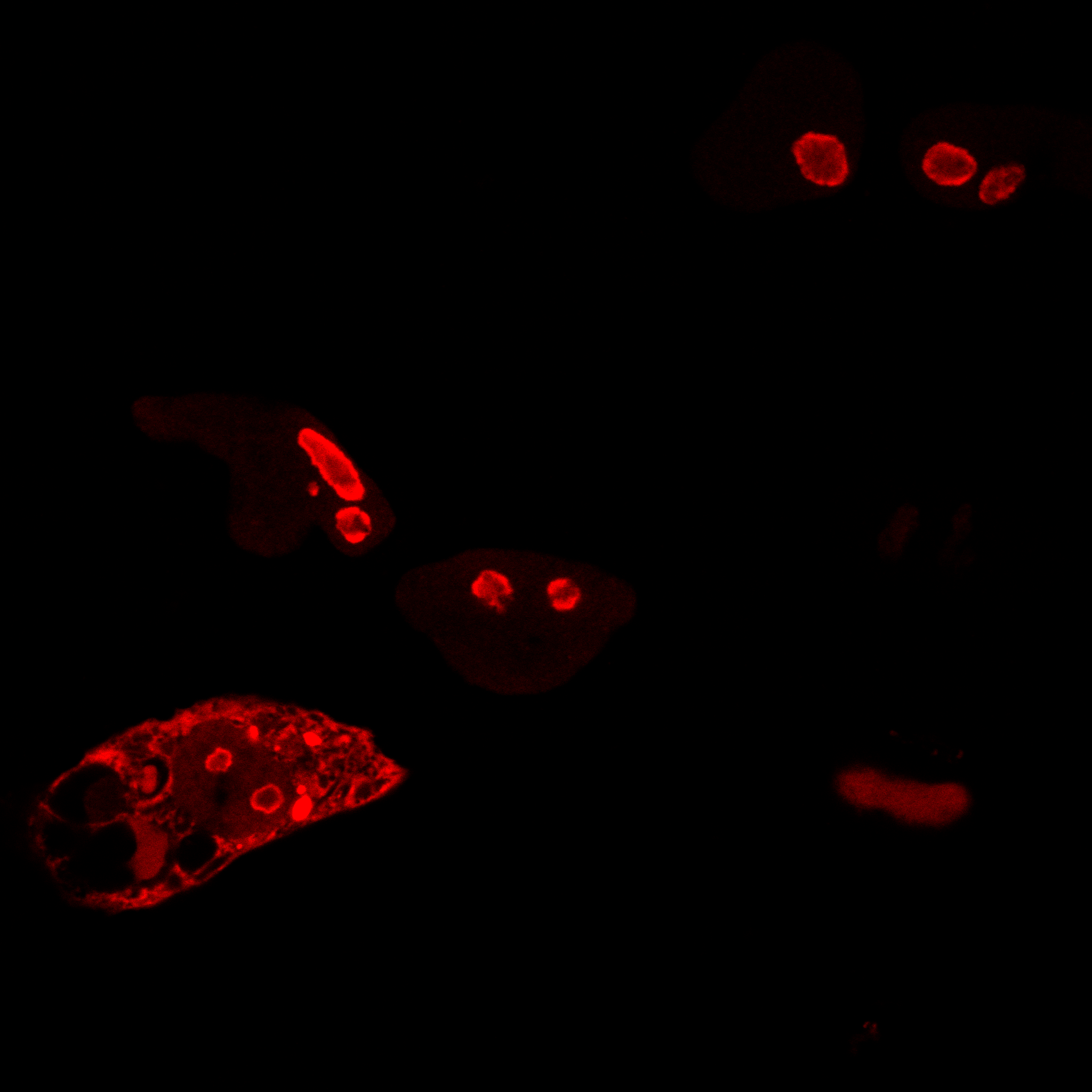

Supplement: S5 Data — (ZIP) [file ppat.1012014.s012.zip › C/C-1/siERK+Mock NPM1.tif]

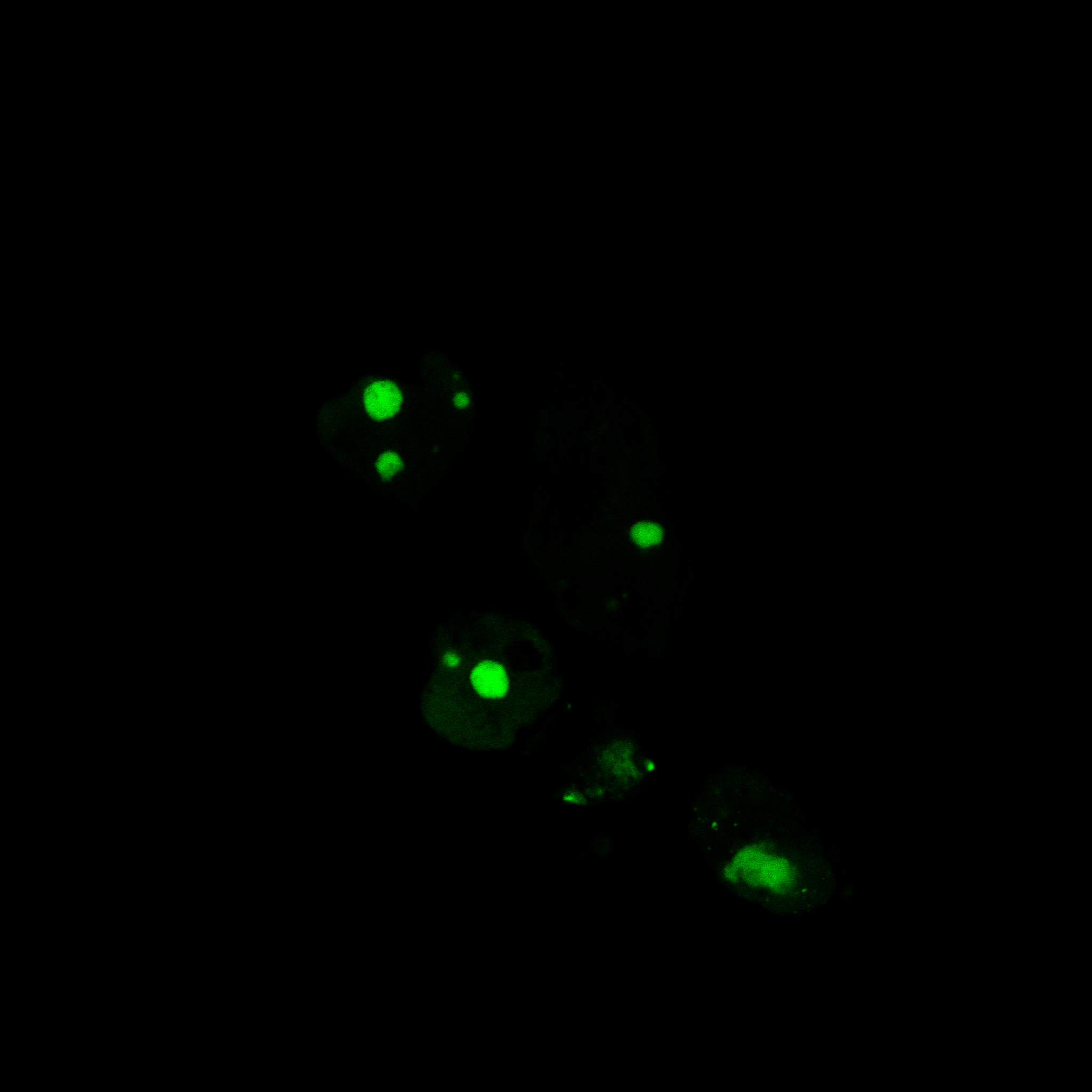

Supplement: S5 Data — (ZIP) [file ppat.1012014.s012.zip › C/C-1/siERK+PCV2 Cap.tif]

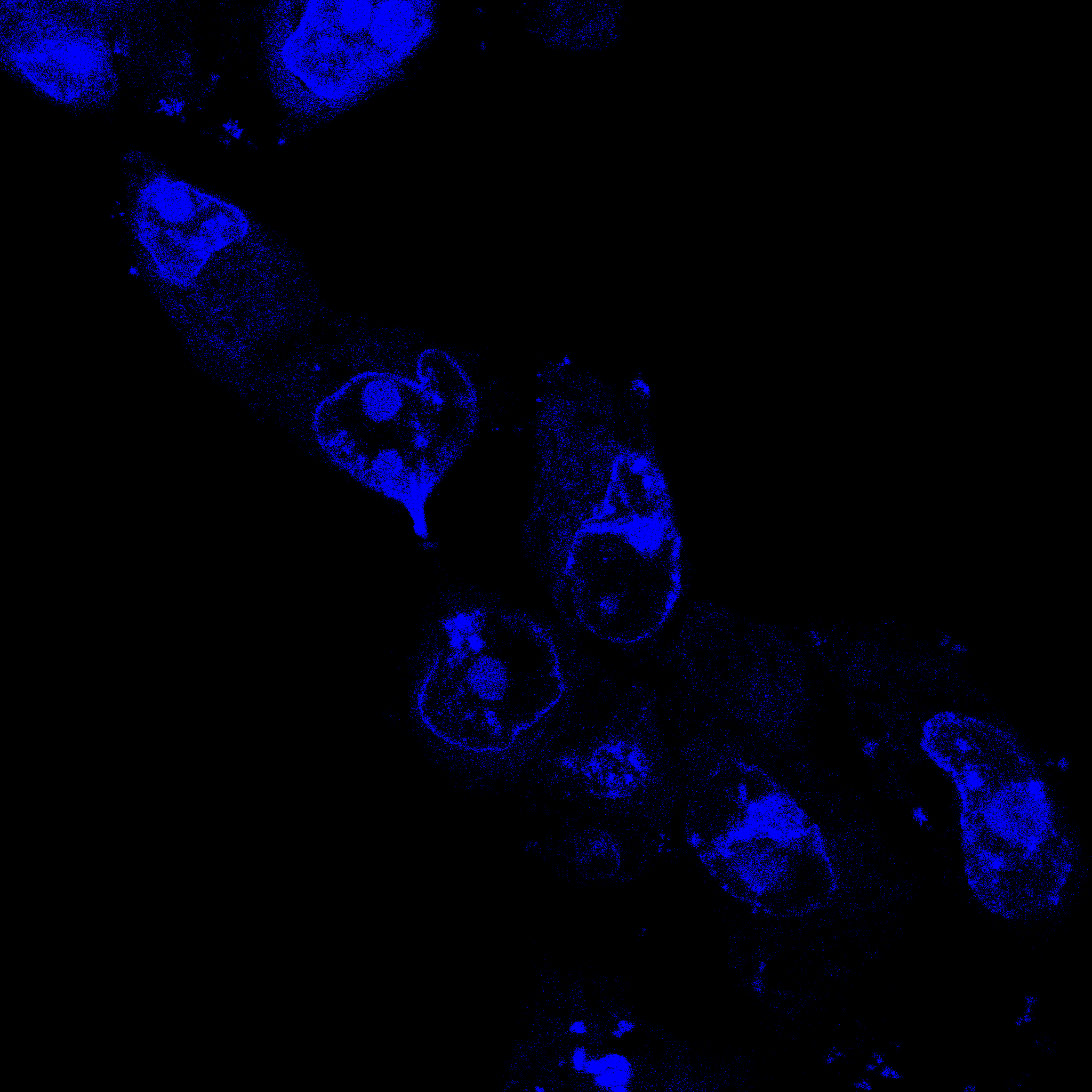

Supplement: S5 Data — (ZIP) [file ppat.1012014.s012.zip › C/C-1/siERK+PCV2 DAPI.tif]

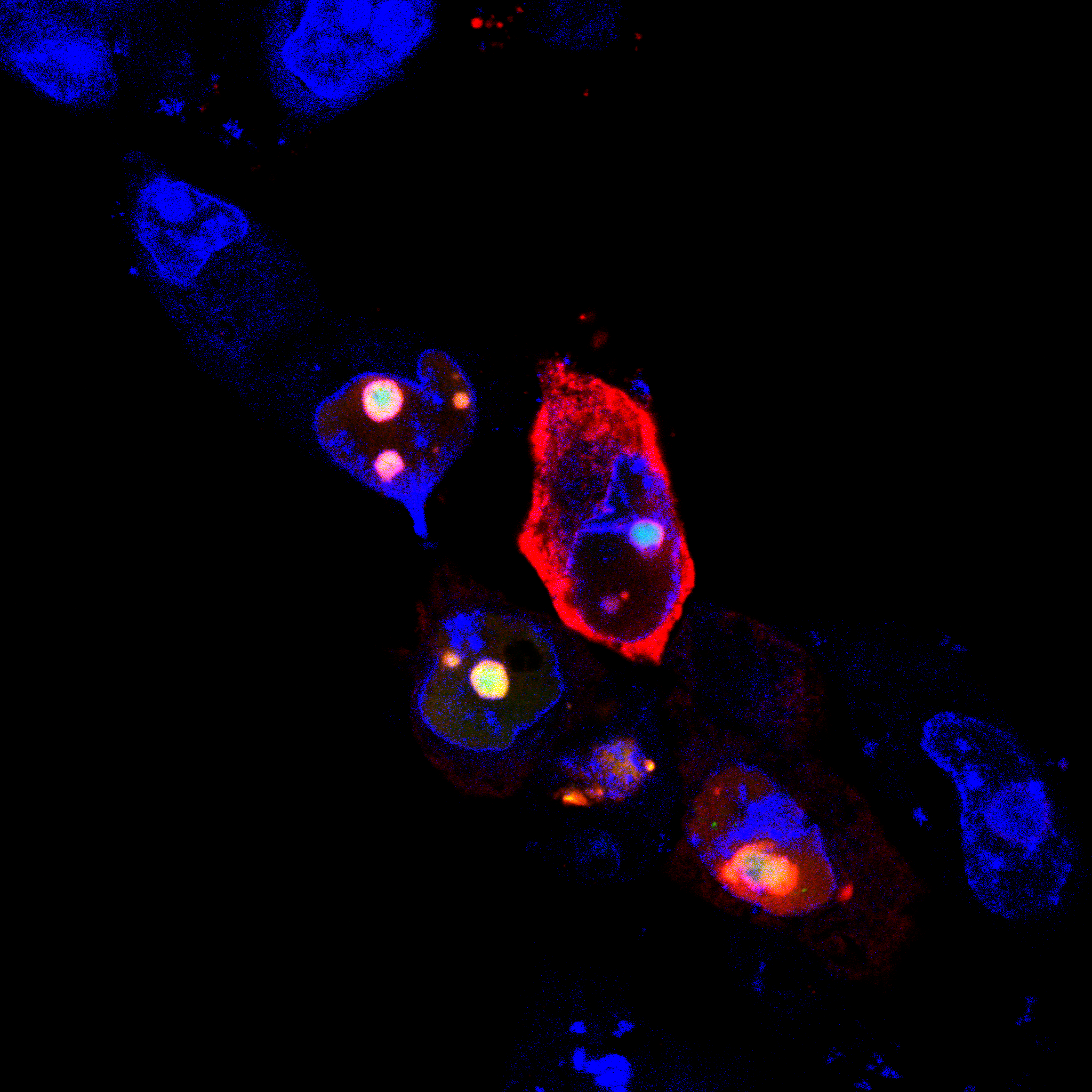

Supplement: S5 Data — (ZIP) [file ppat.1012014.s012.zip › C/C-1/siERK+PCV2 Merge.tif]

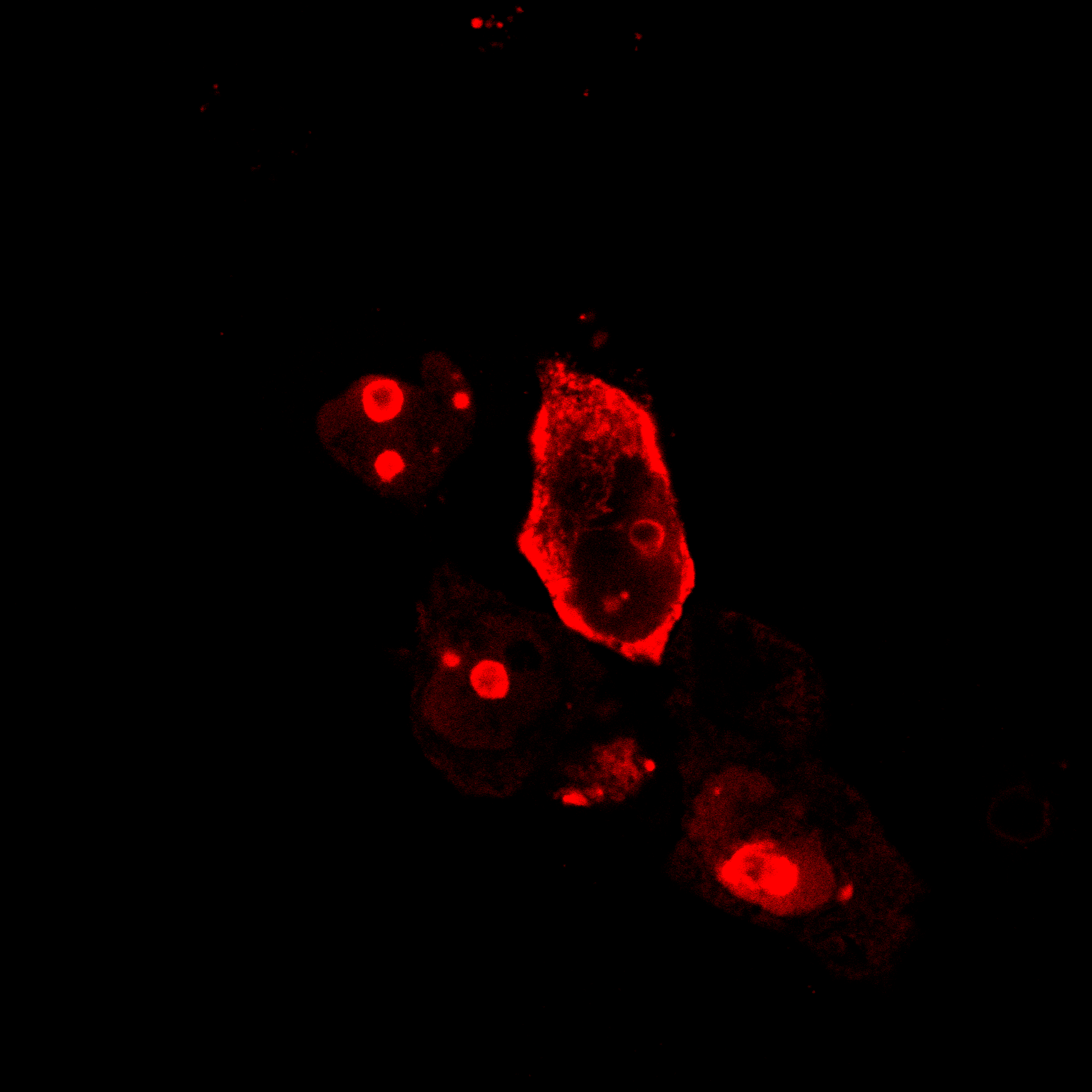

Supplement: S5 Data — (ZIP) [file ppat.1012014.s012.zip › C/C-1/siERK+PCV2 NPM1.tif]

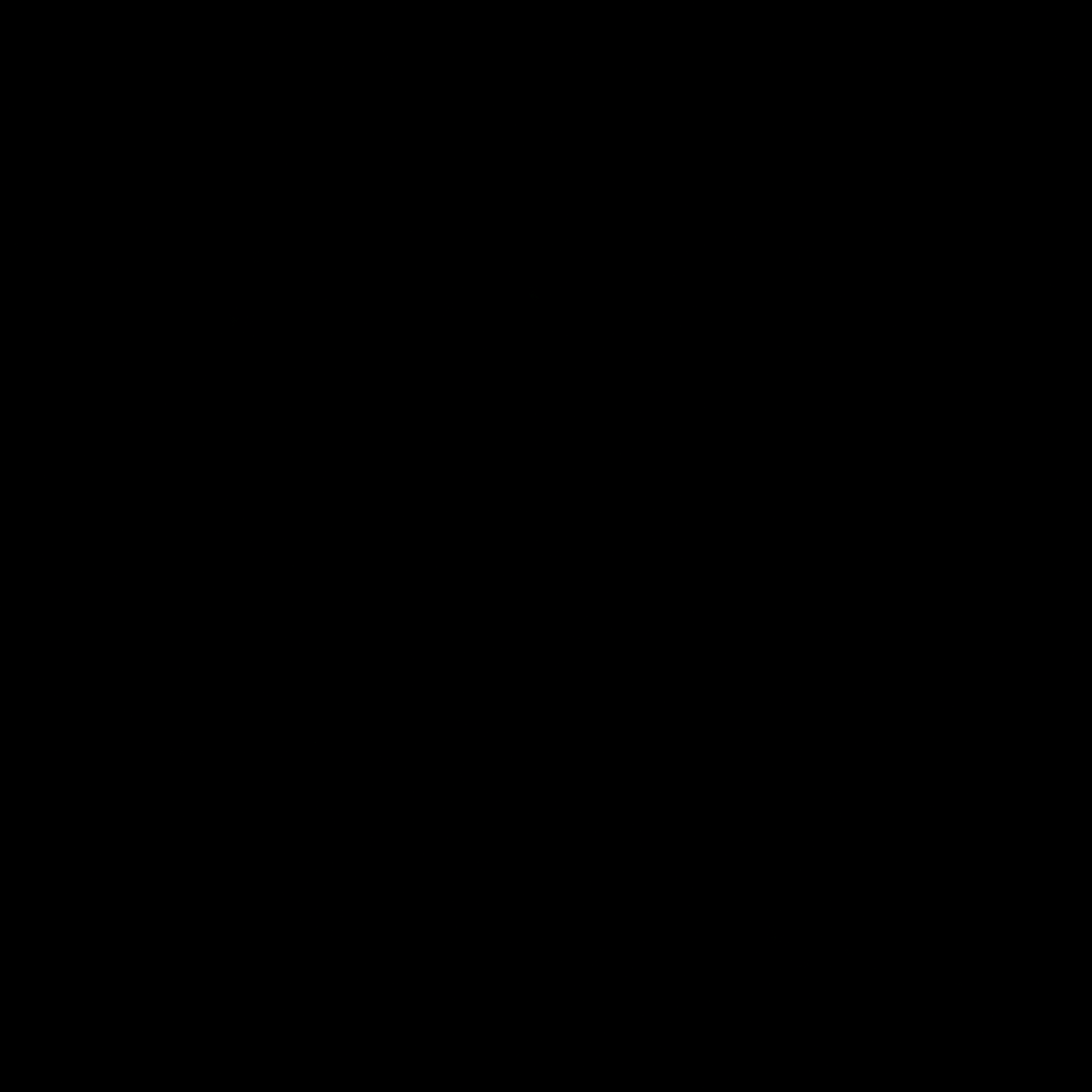

Supplement: S5 Data — (ZIP) [file ppat.1012014.s012.zip › C/C-1/siNC+Mock Cap.tif]

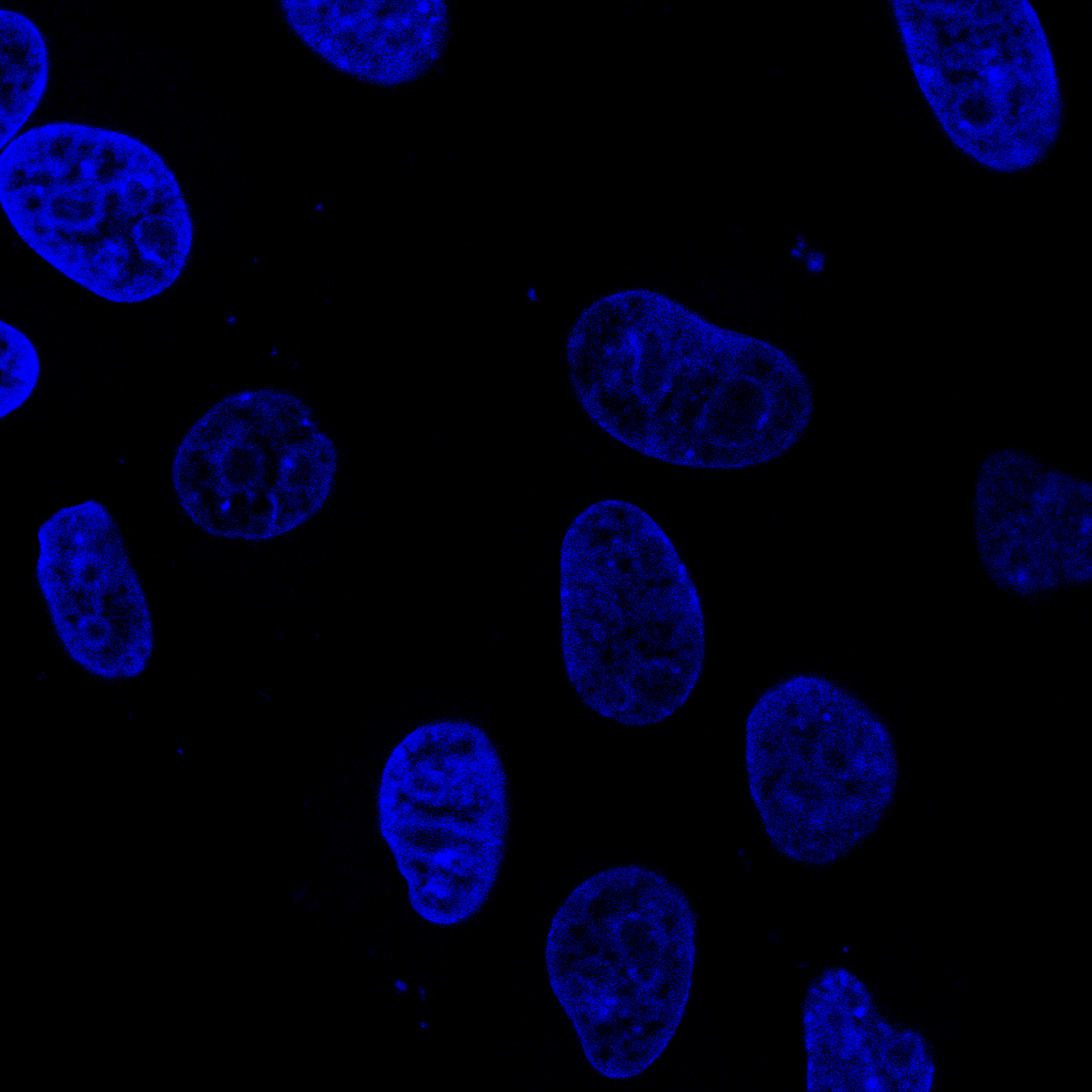

Supplement: S5 Data — (ZIP) [file ppat.1012014.s012.zip › C/C-1/siNC+Mock DAPI.tif]

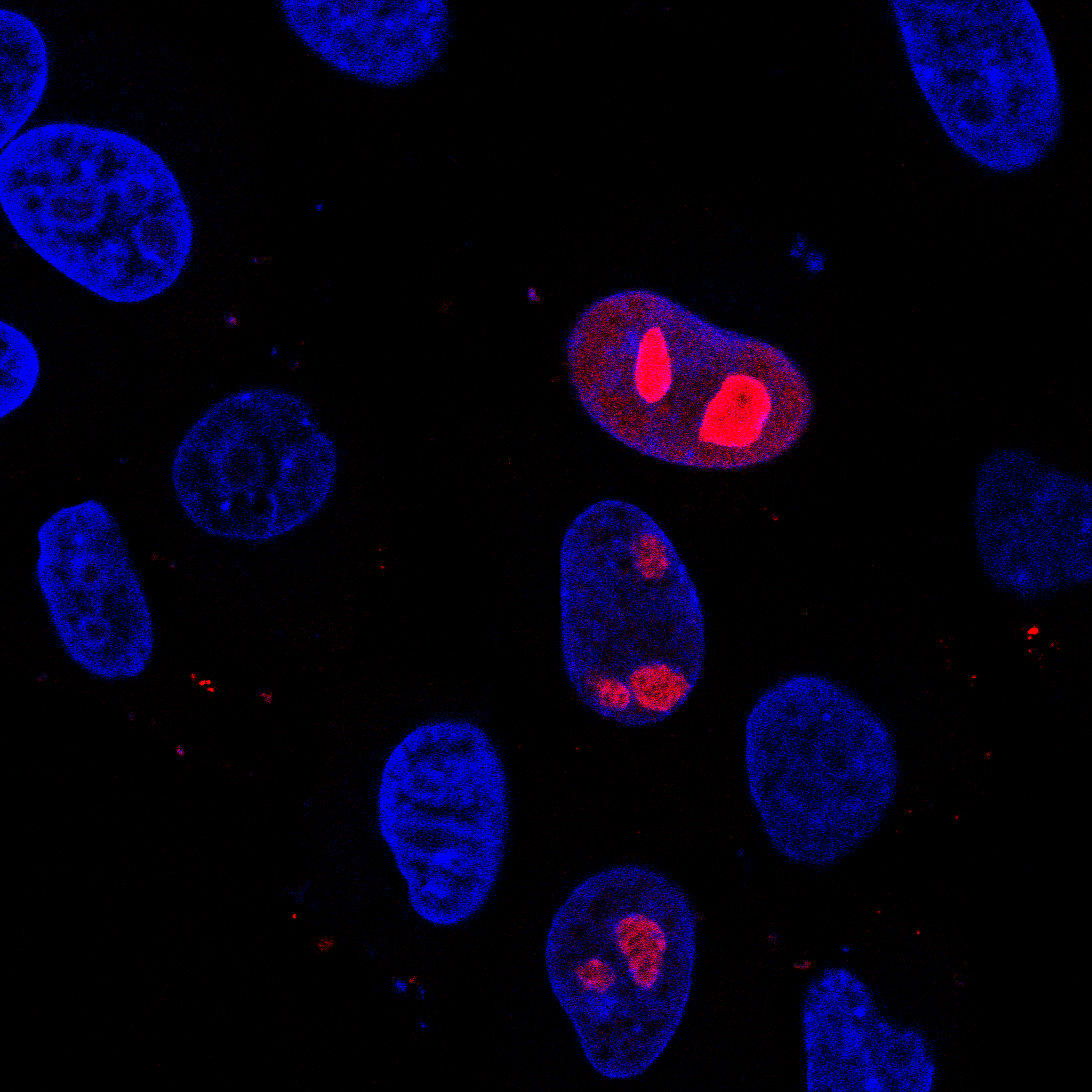

Supplement: S5 Data — (ZIP) [file ppat.1012014.s012.zip › C/C-1/siNC+Mock Merge.tif]

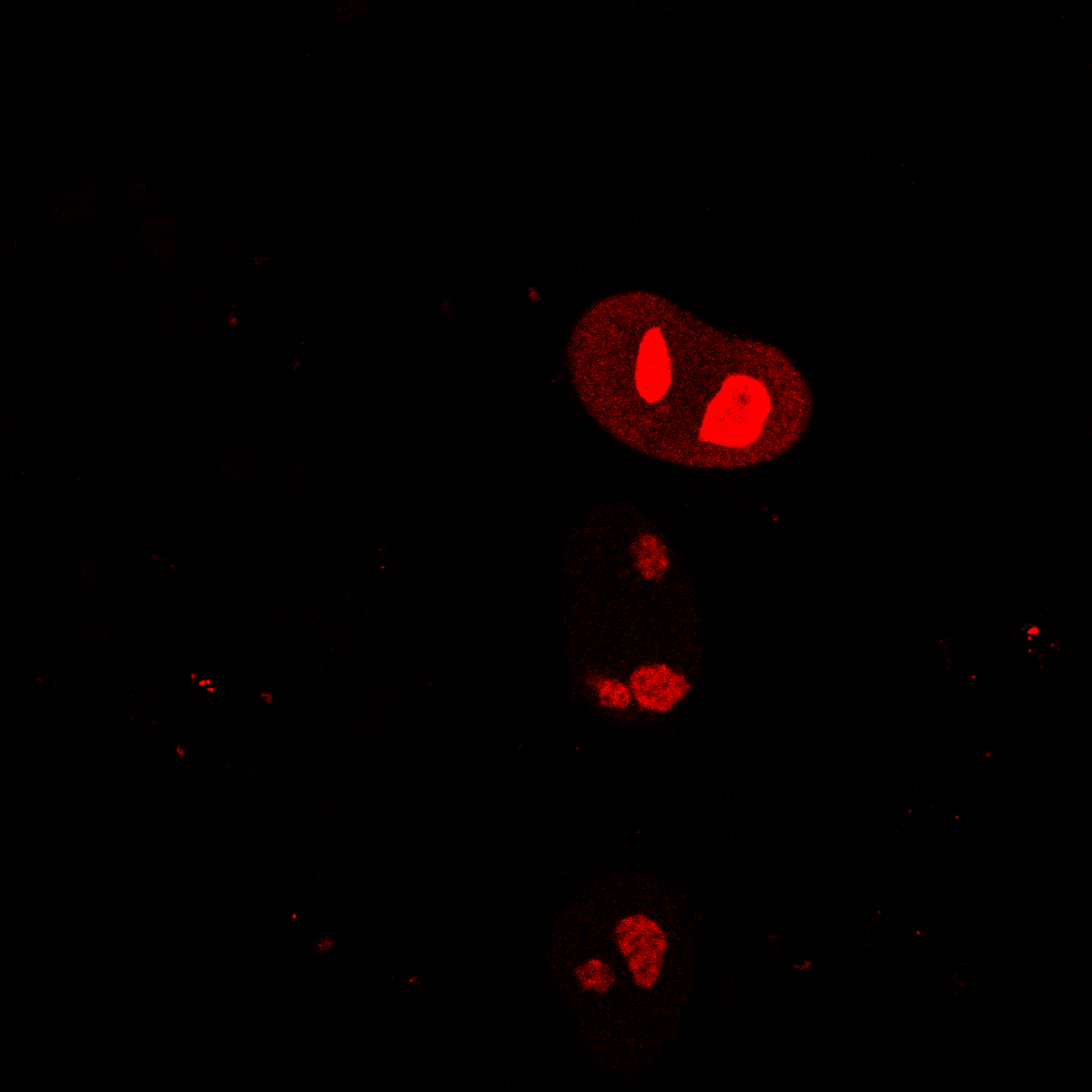

Supplement: S5 Data — (ZIP) [file ppat.1012014.s012.zip › C/C-1/siNC+Mock NPM1.tif]

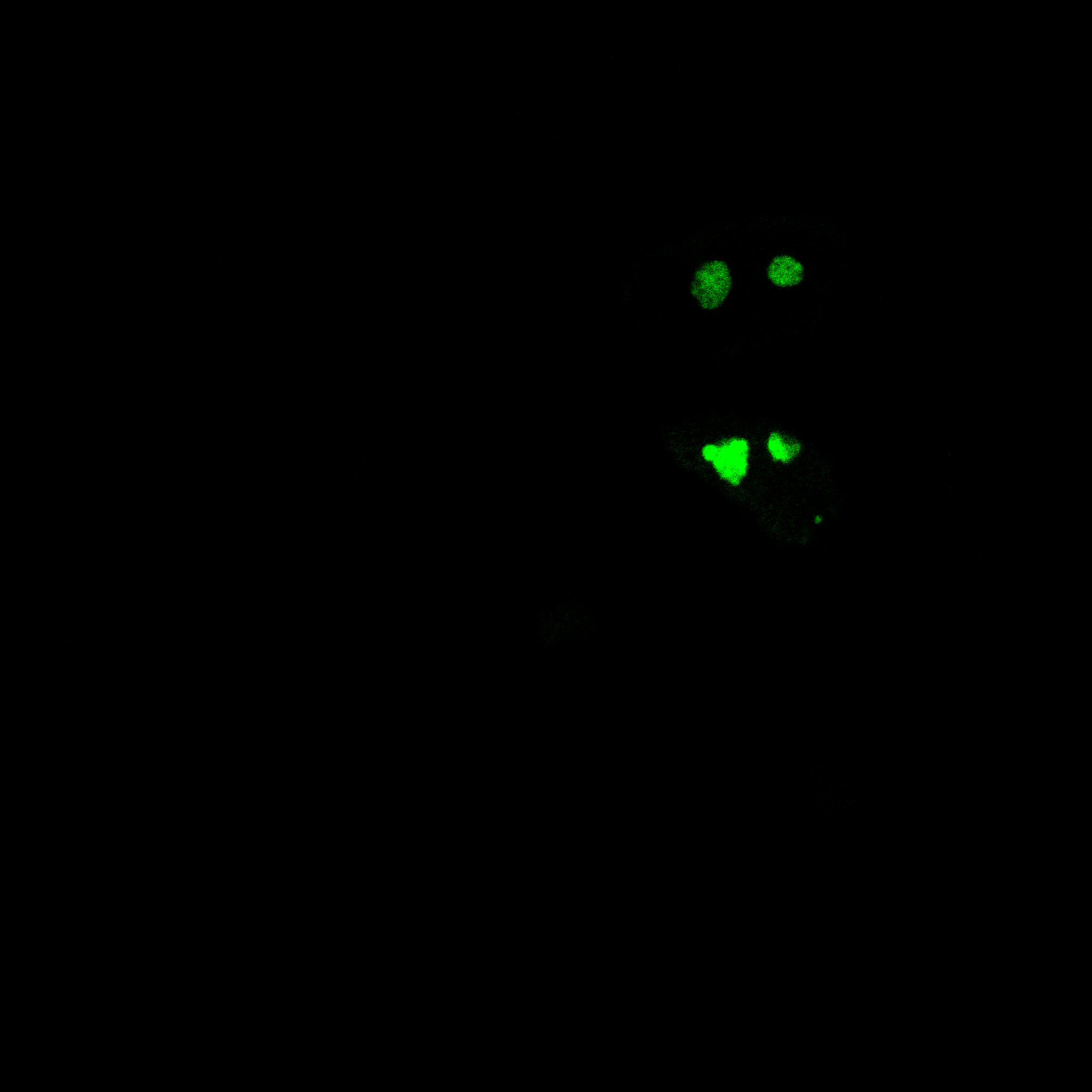

Supplement: S5 Data — (ZIP) [file ppat.1012014.s012.zip › C/C-1/siNC+PCV2 Cap.tif]

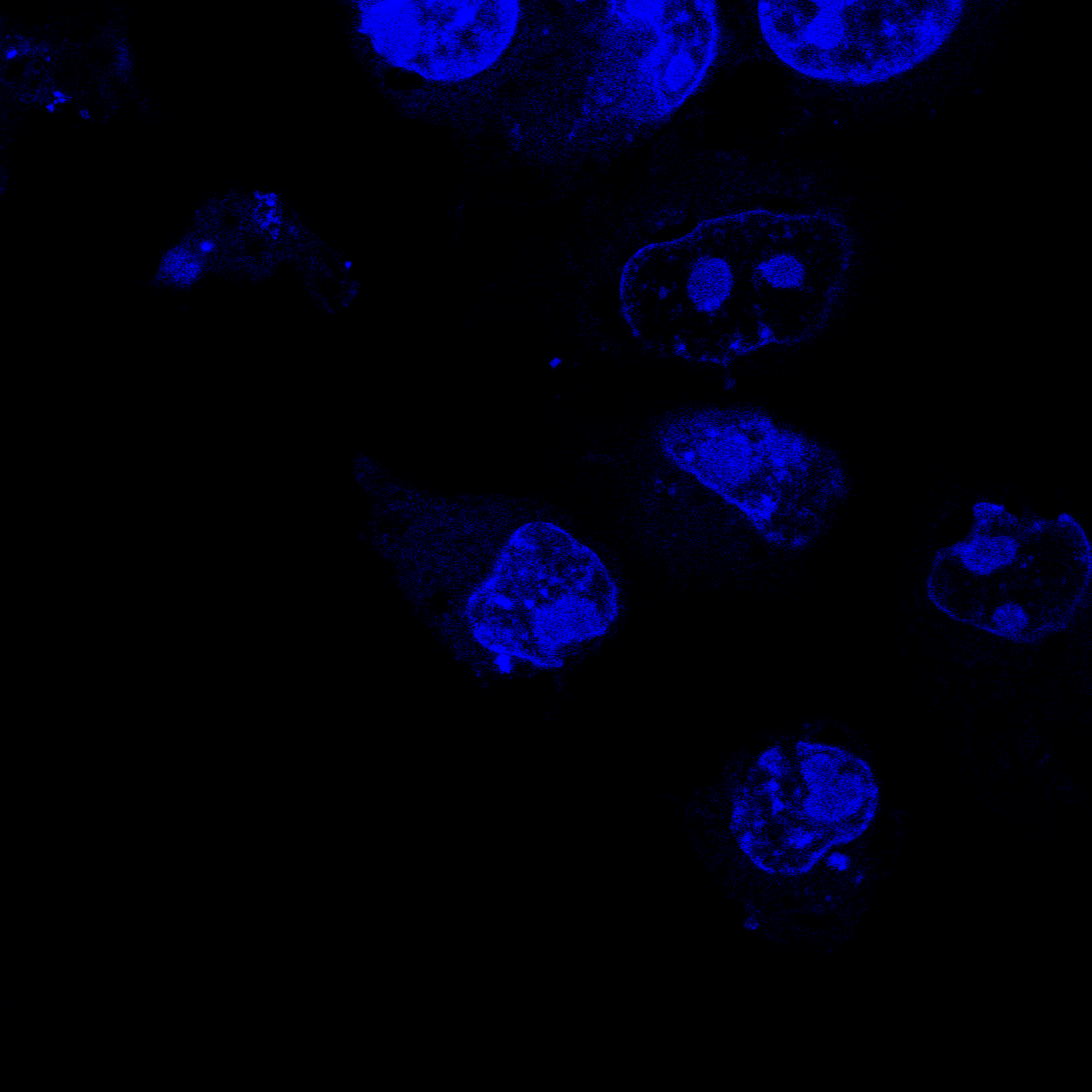

Supplement: S5 Data — (ZIP) [file ppat.1012014.s012.zip › C/C-1/siNC+PCV2 DAPI.tif]

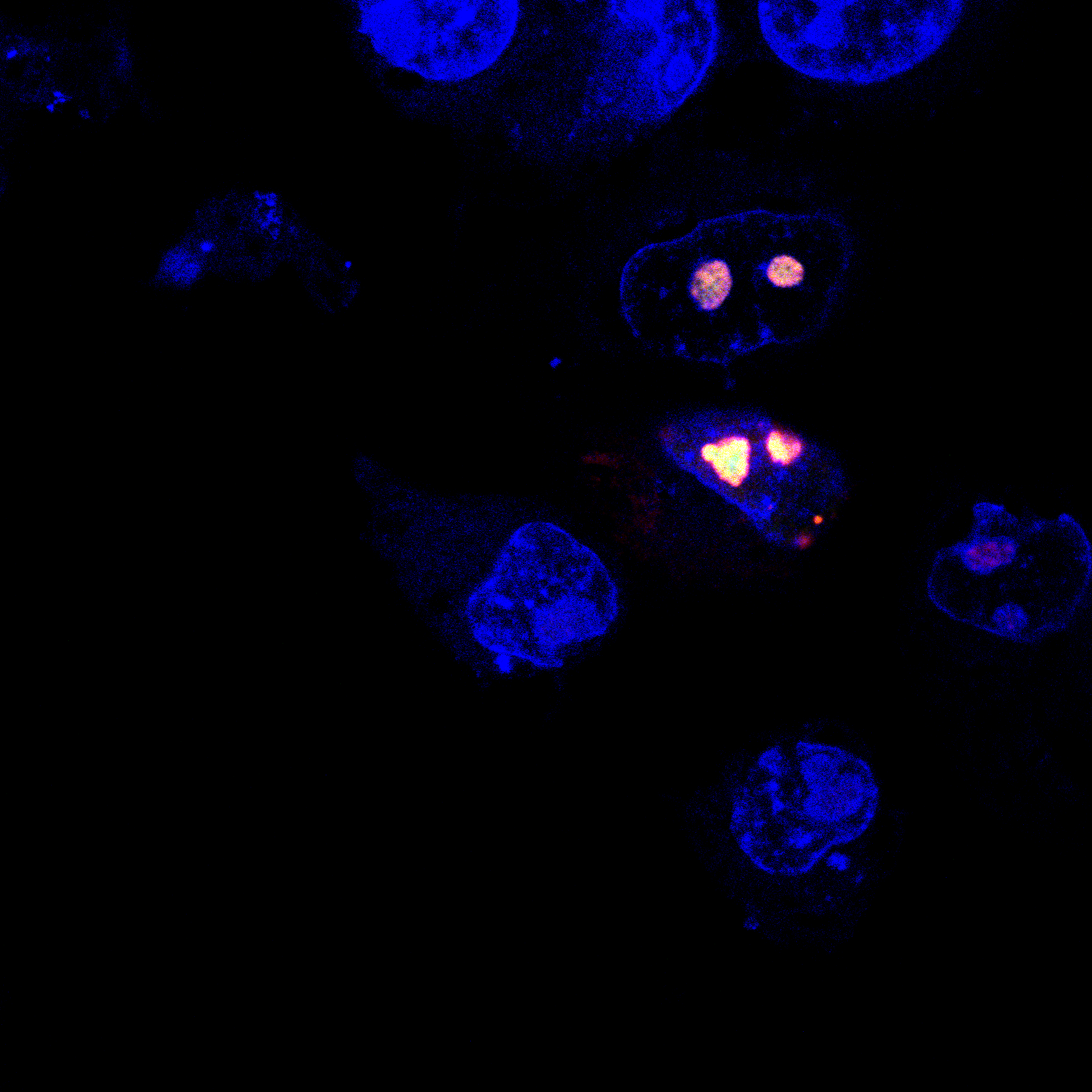

Supplement: S5 Data — (ZIP) [file ppat.1012014.s012.zip › C/C-1/siNC+PCV2 Merge.tif]

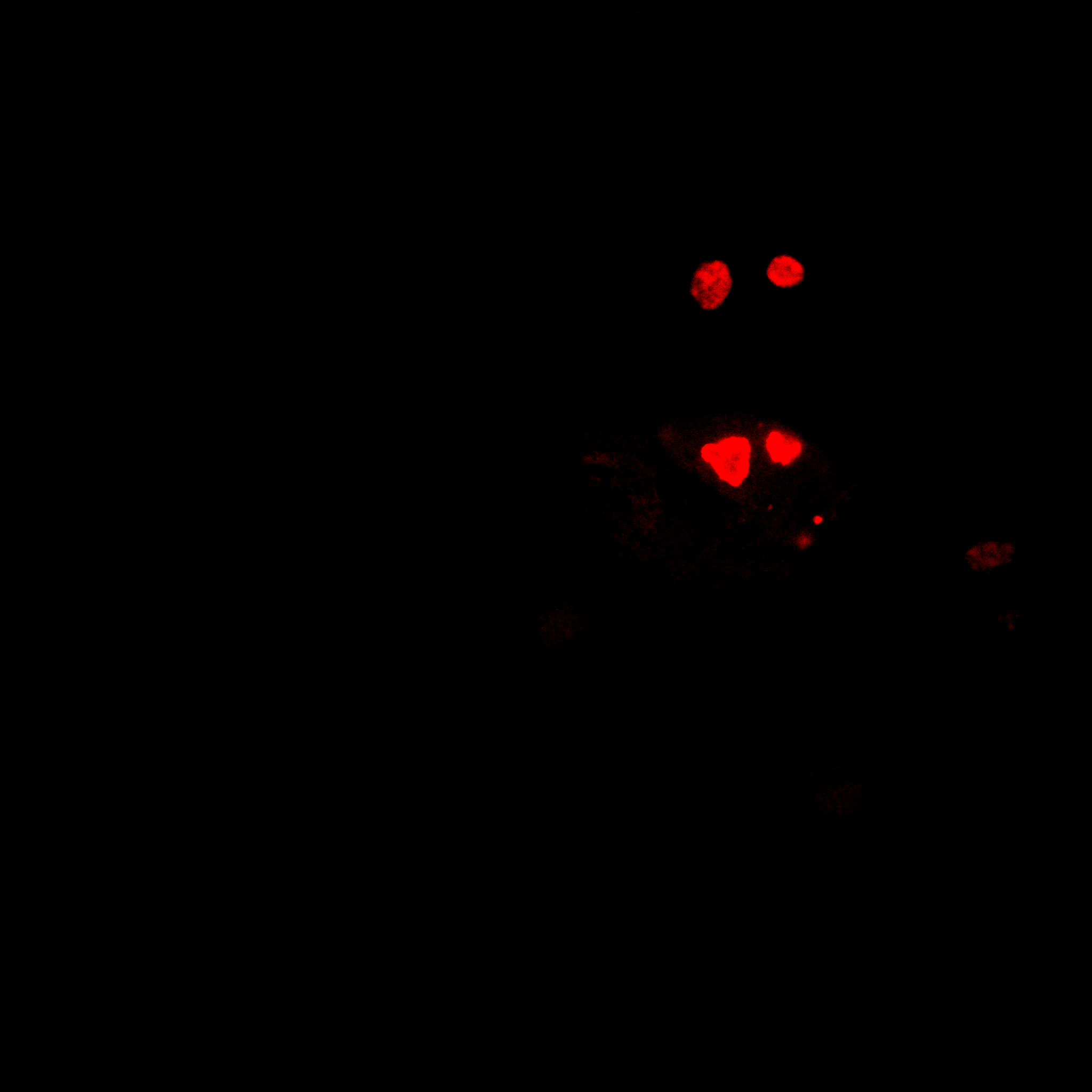

Supplement: S5 Data — (ZIP) [file ppat.1012014.s012.zip › C/C-1/siNC+PCV2 NPM1.tif]

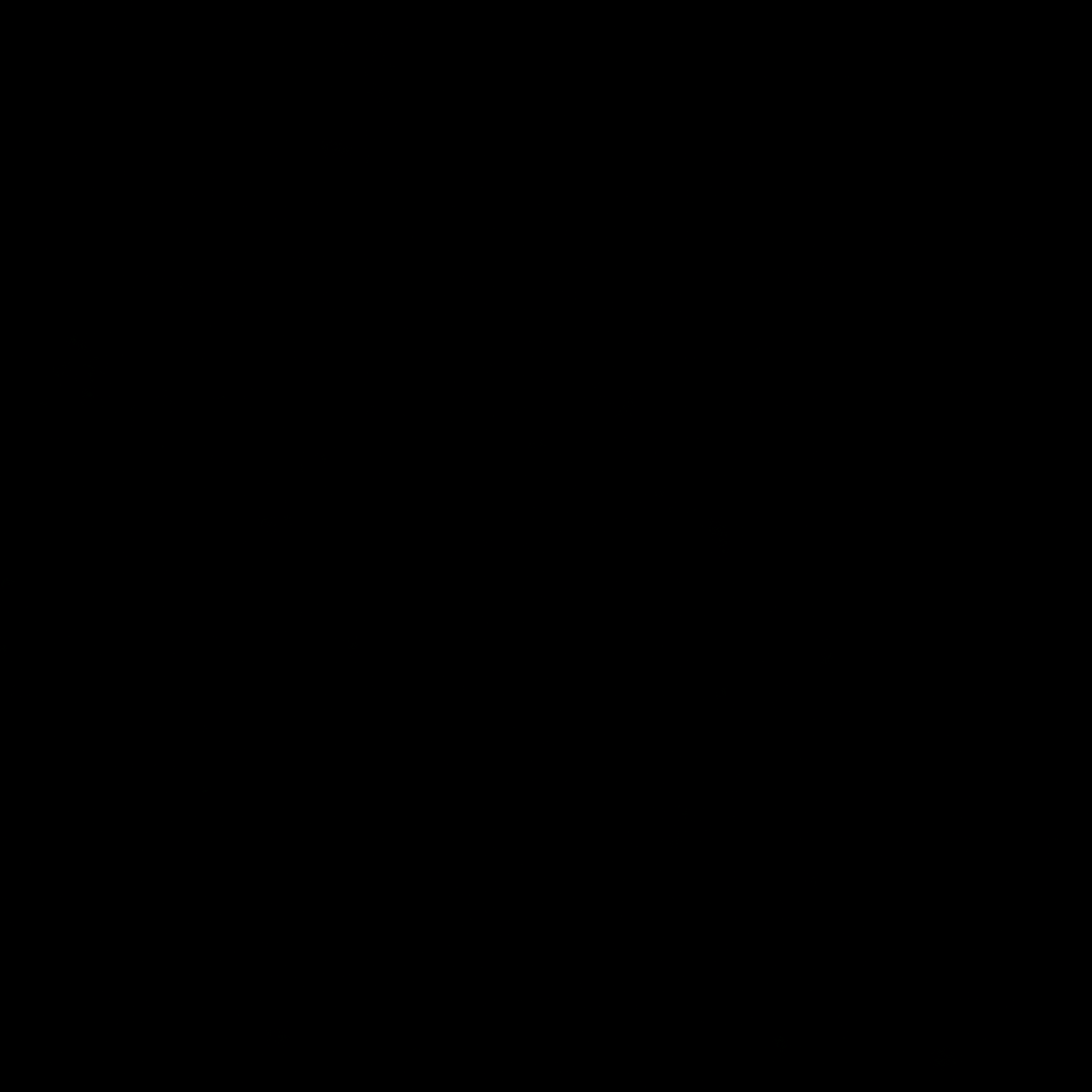

Supplement: S5 Data — (ZIP) [file ppat.1012014.s012.zip › C/C-2/siERK+Mock Cap.tif]

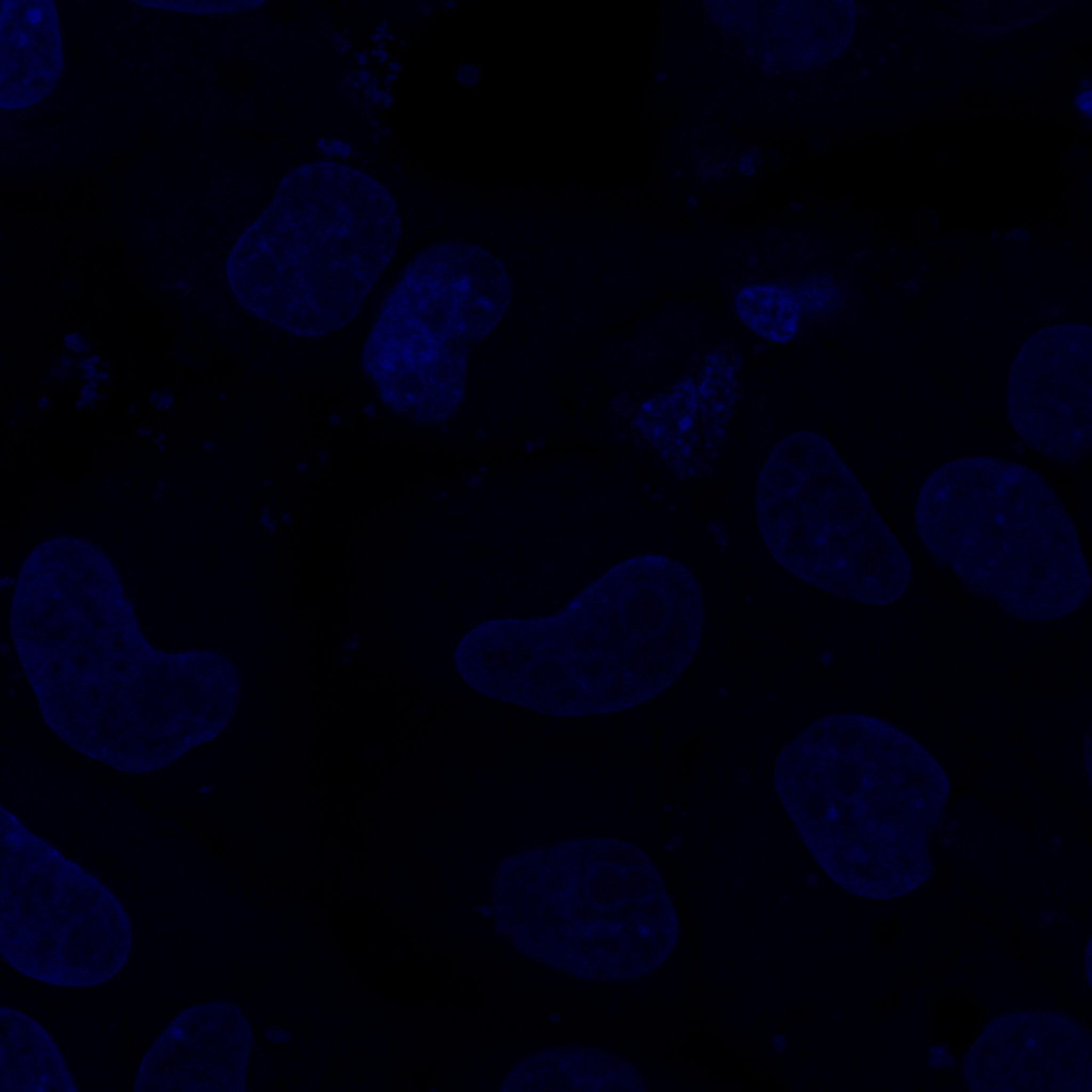

Supplement: S5 Data — (ZIP) [file ppat.1012014.s012.zip › C/C-2/siERK+Mock DAPI.tif]

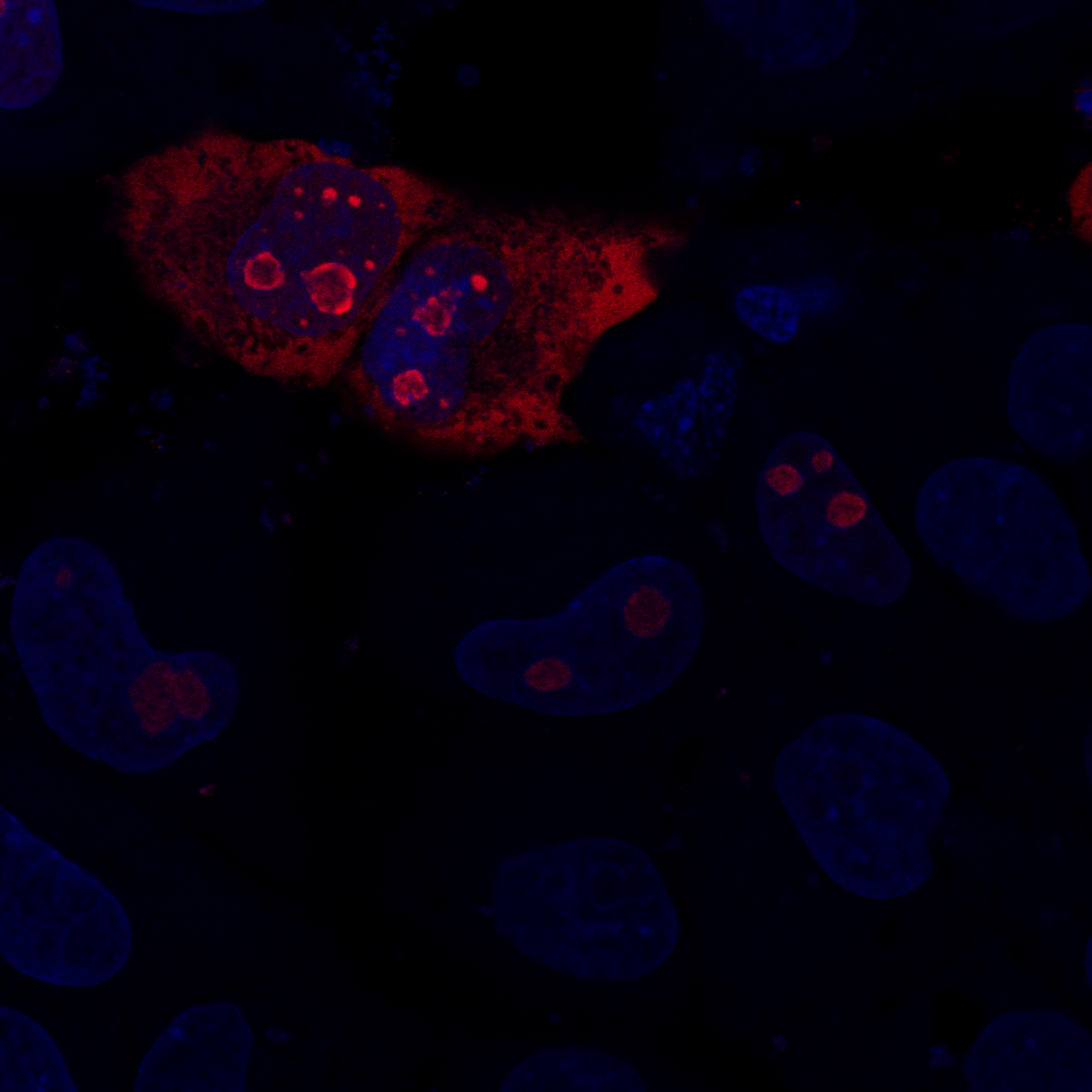

Supplement: S5 Data — (ZIP) [file ppat.1012014.s012.zip › C/C-2/siERK+Mock Merge.tif]

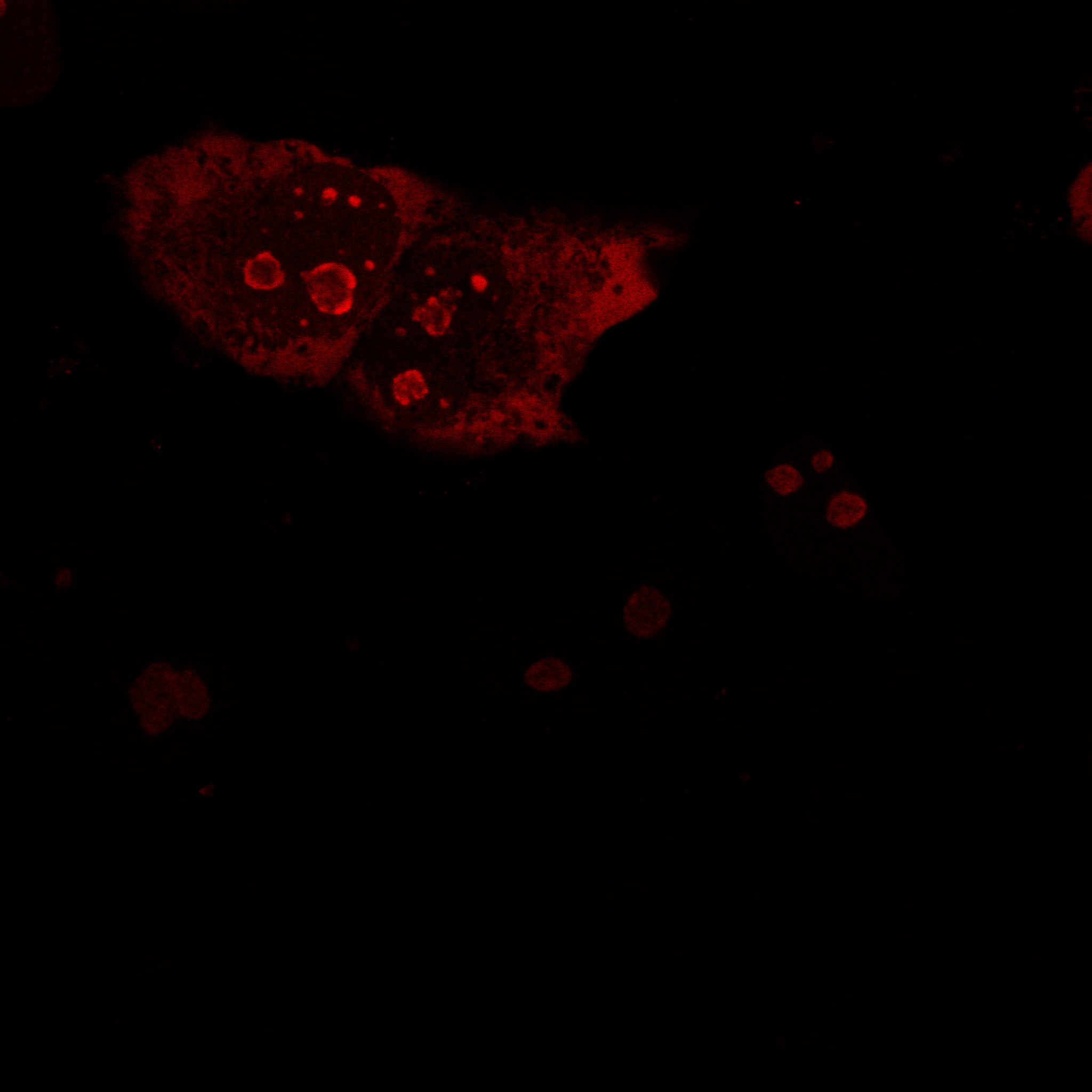

Supplement: S5 Data — (ZIP) [file ppat.1012014.s012.zip › C/C-2/siERK+Mock NPM1.tif]

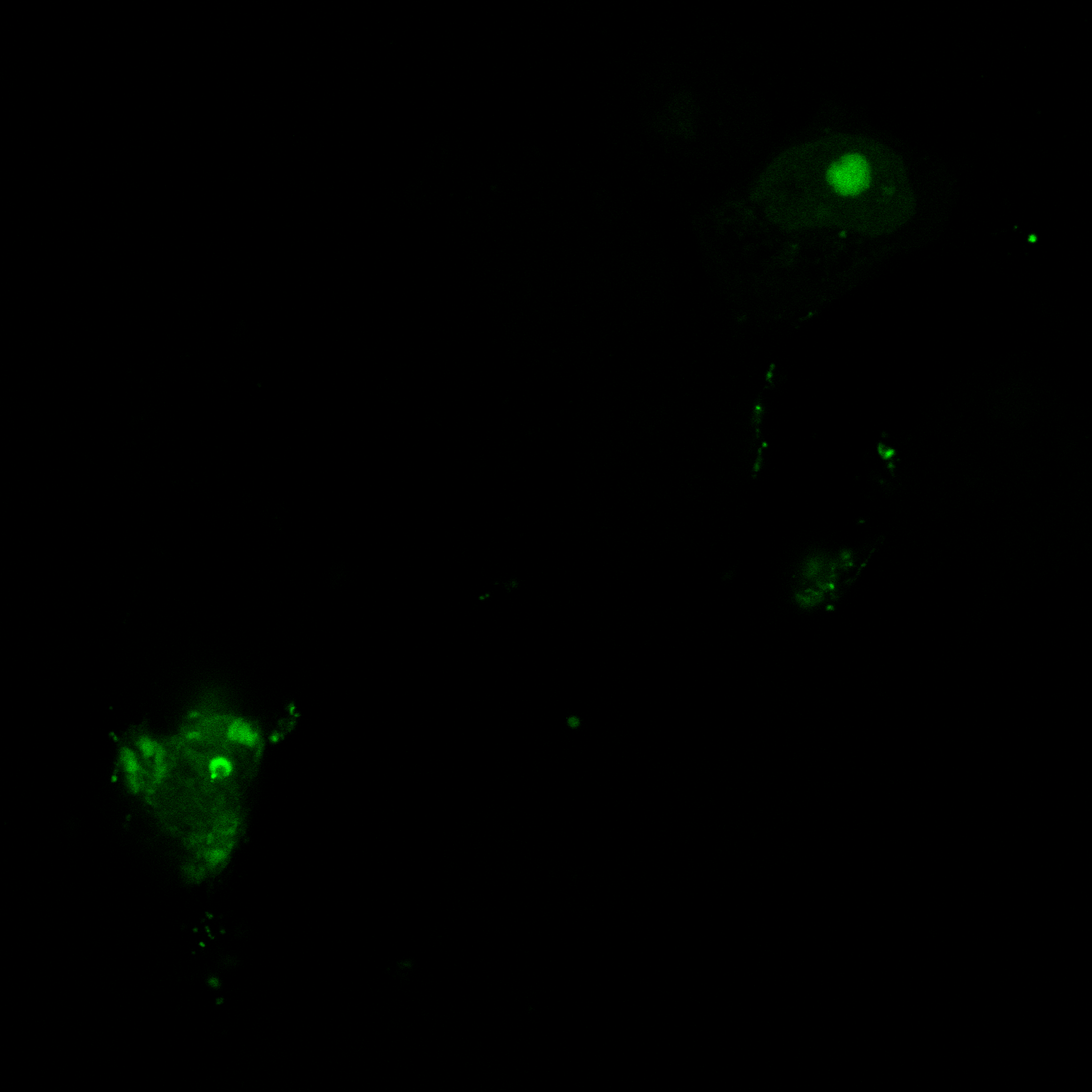

Supplement: S5 Data — (ZIP) [file ppat.1012014.s012.zip › C/C-2/siERK+PCV2 Cap.tif]

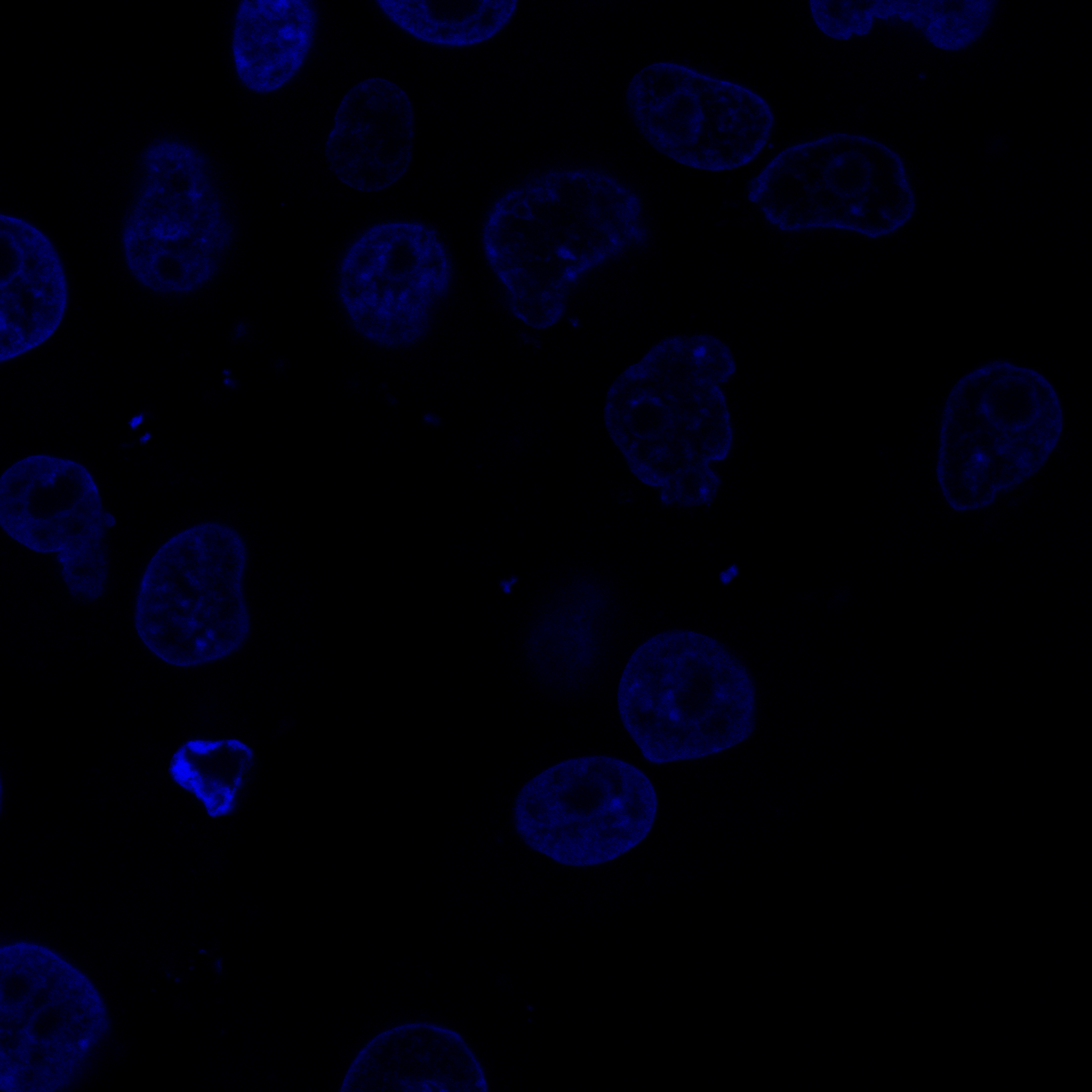

Supplement: S5 Data — (ZIP) [file ppat.1012014.s012.zip › C/C-2/siERK+PCV2 DAPI.tif]

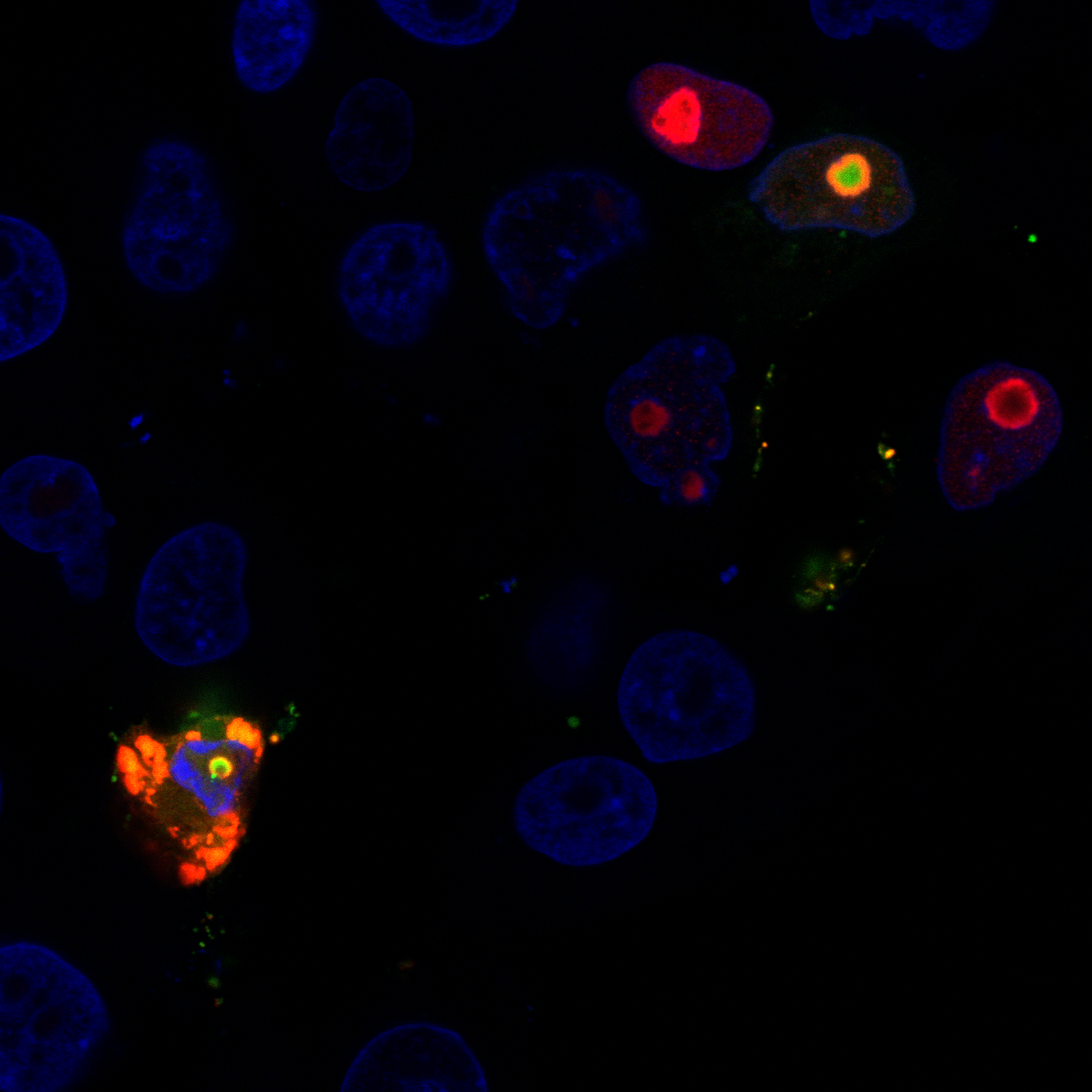

Supplement: S5 Data — (ZIP) [file ppat.1012014.s012.zip › C/C-2/siERK+PCV2 Merge.tif]

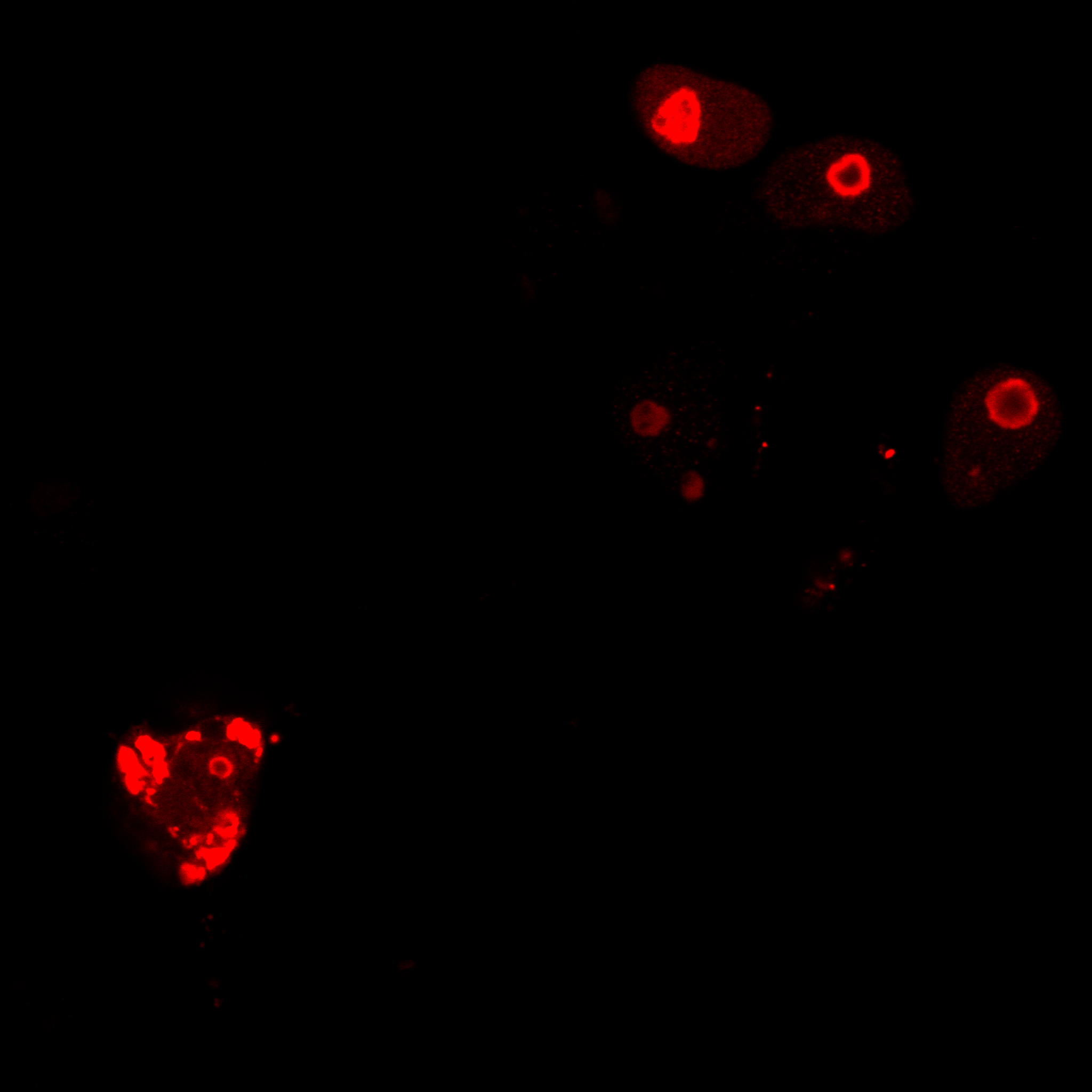

Supplement: S5 Data — (ZIP) [file ppat.1012014.s012.zip › C/C-2/siERK+PCV2 NPM1.tif]

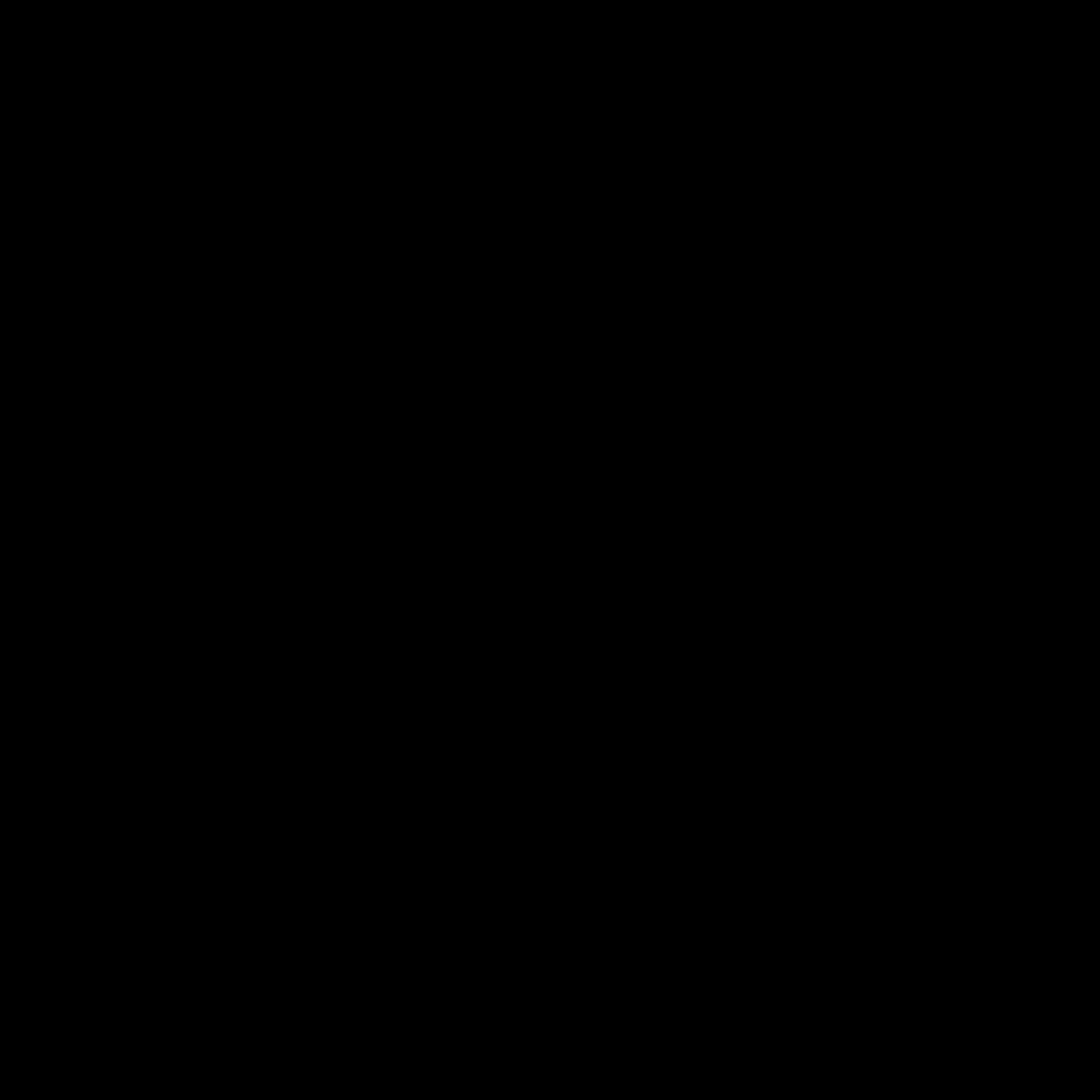

Supplement: S5 Data — (ZIP) [file ppat.1012014.s012.zip › C/C-2/siNC+Mock Cap.tif]

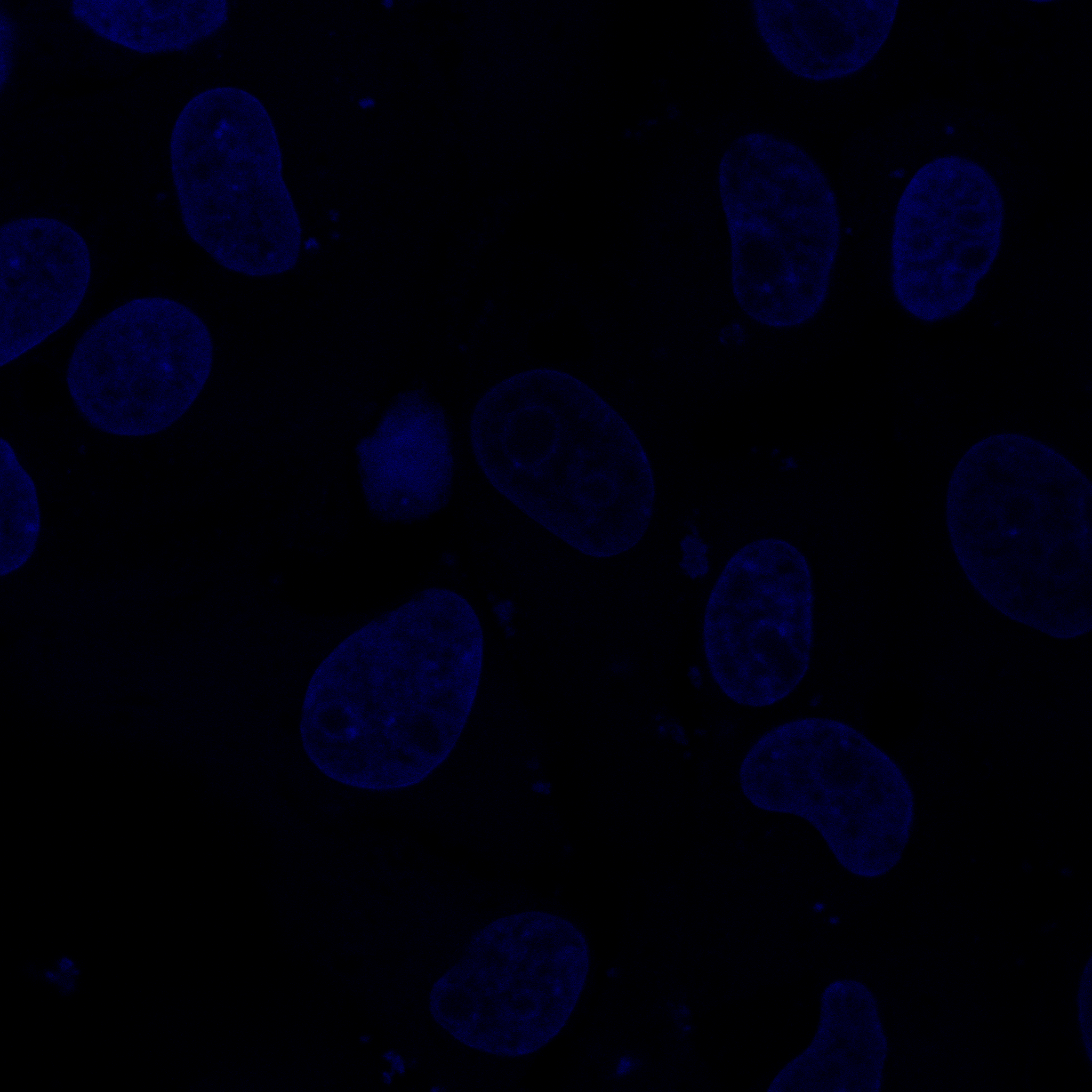

Supplement: S5 Data — (ZIP) [file ppat.1012014.s012.zip › C/C-2/siNC+Mock DAPI.tif]

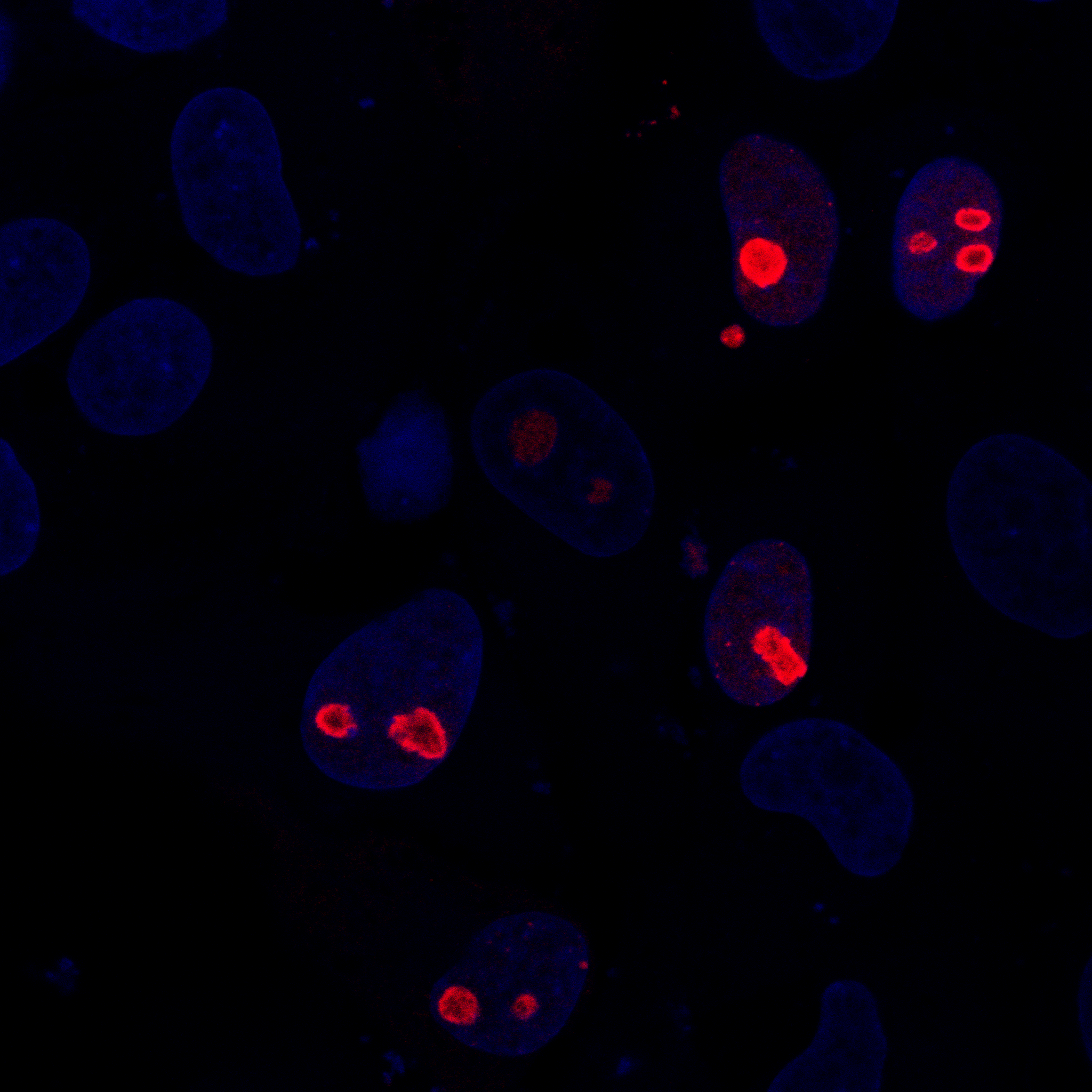

Supplement: S5 Data — (ZIP) [file ppat.1012014.s012.zip › C/C-2/siNC+Mock Merge.tif]

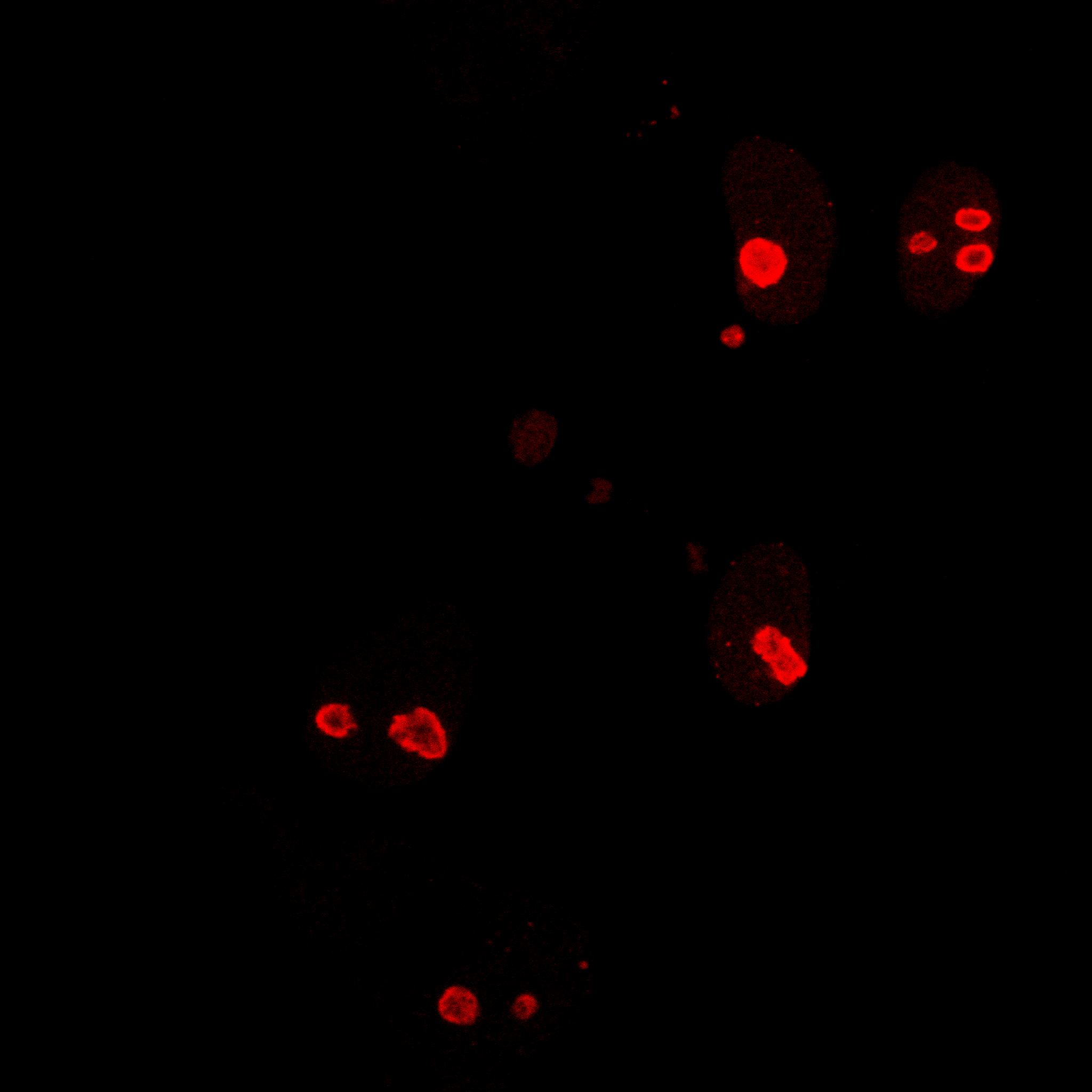

Supplement: S5 Data — (ZIP) [file ppat.1012014.s012.zip › C/C-2/siNC+Mock NPM1.tif]

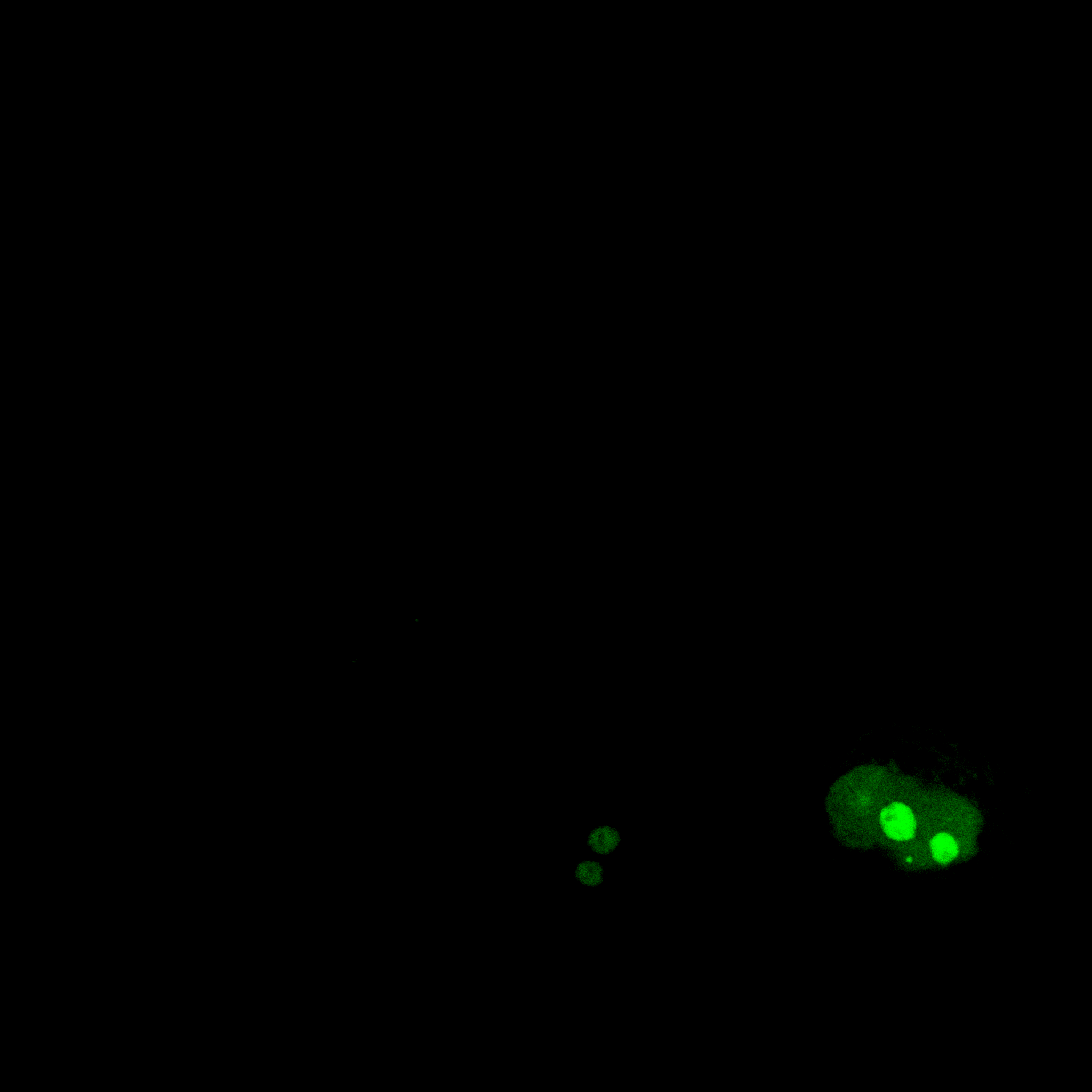

Supplement: S5 Data — (ZIP) [file ppat.1012014.s012.zip › C/C-2/siNC+PCV2 Cap.tif]

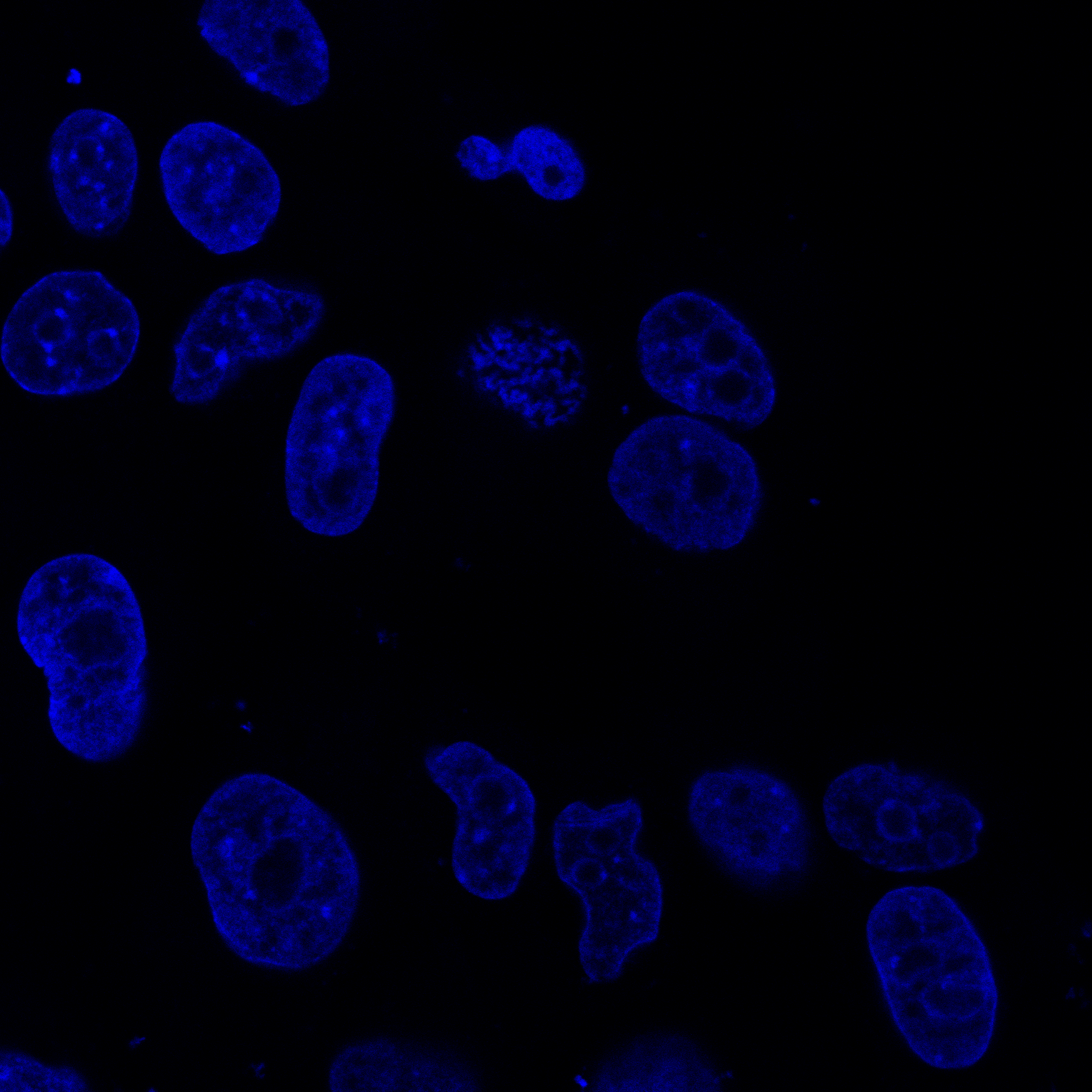

Supplement: S5 Data — (ZIP) [file ppat.1012014.s012.zip › C/C-2/siNC+PCV2 DAPI.tif]

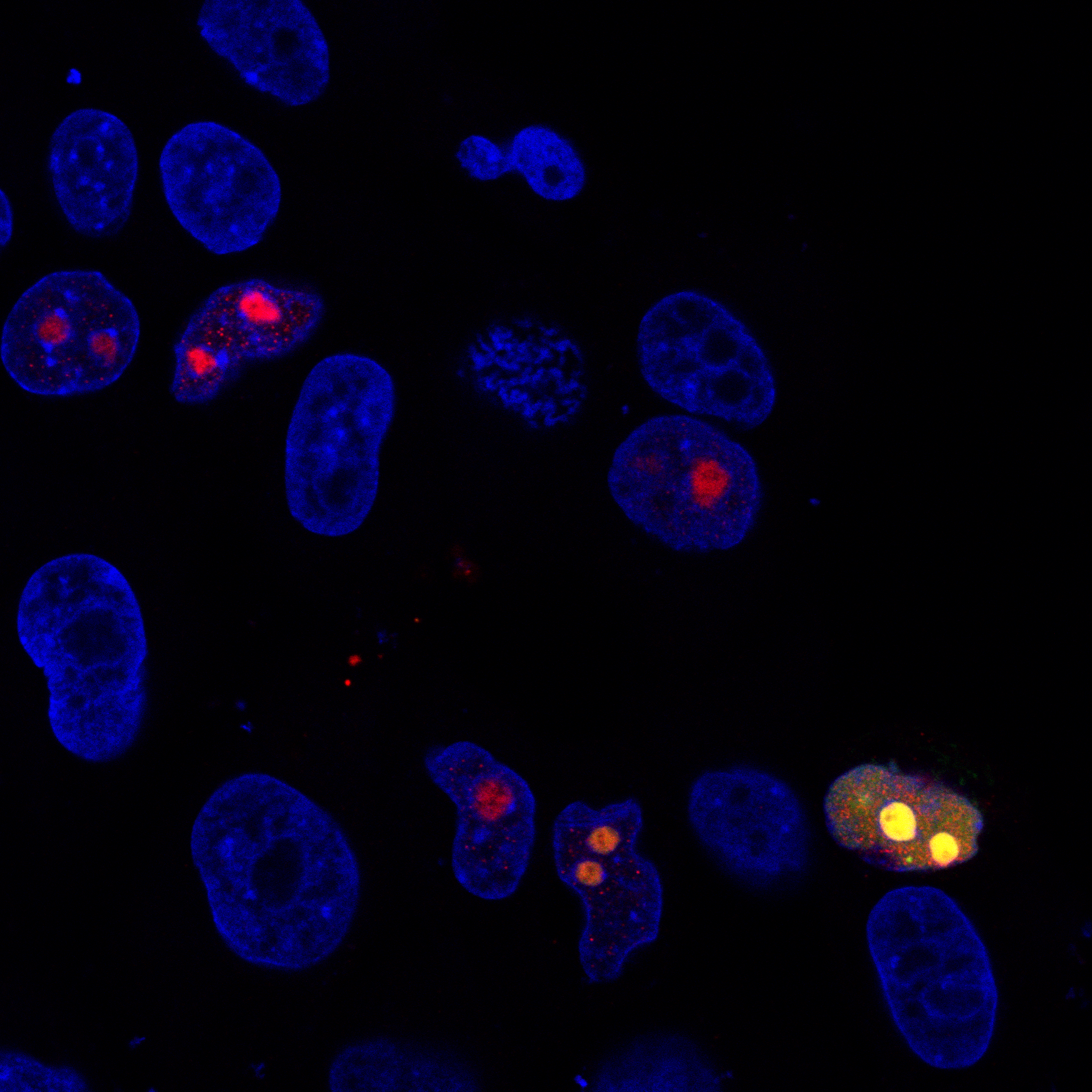

Supplement: S5 Data — (ZIP) [file ppat.1012014.s012.zip › C/C-2/siNC+PCV2 Merge.tif]

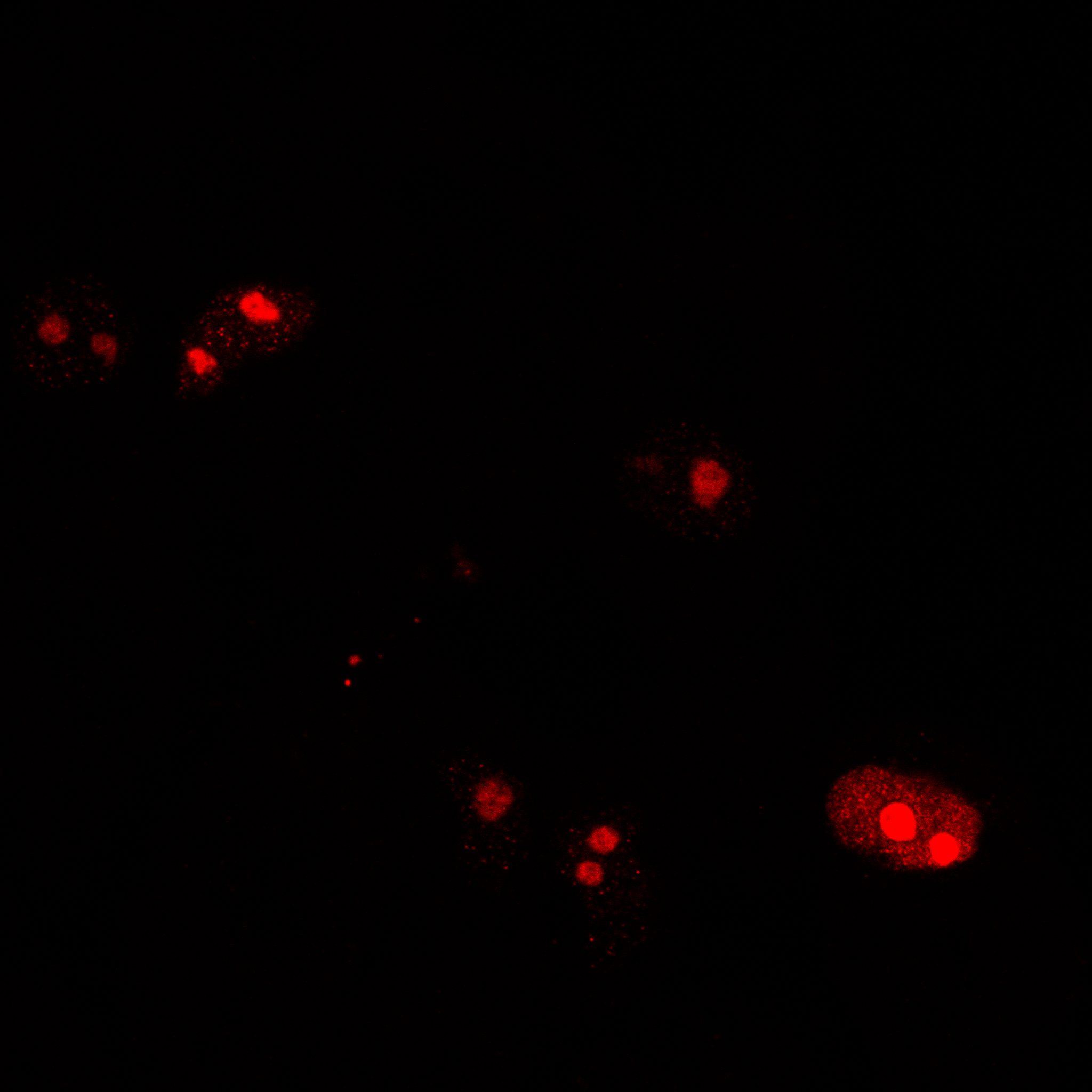

Supplement: S5 Data — (ZIP) [file ppat.1012014.s012.zip › C/C-2/siNC+PCV2 NPM1.tif]

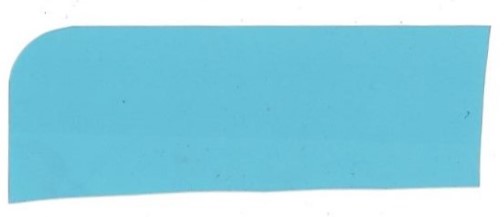

Supplement: S5 Data — (ZIP) [file ppat.1012014.s012.zip › D/1/Cytoplasm-Cap-1.jpg]

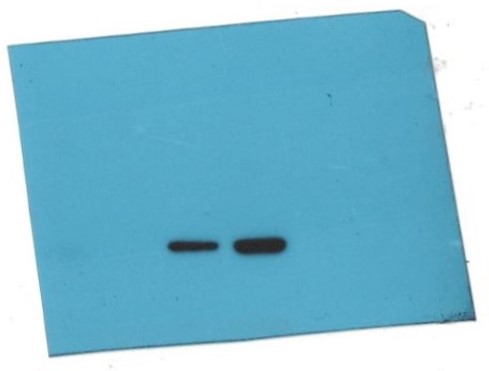

Supplement: S5 Data — (ZIP) [file ppat.1012014.s012.zip › D/1/Cytoplasm-NPM1-1.jpg]

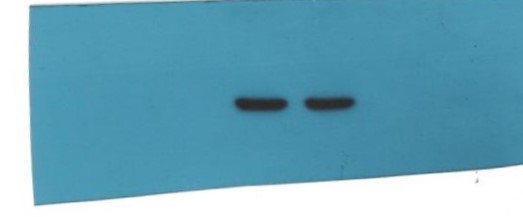

Supplement: S5 Data — (ZIP) [file ppat.1012014.s012.zip › D/1/Cytoplasm-β-actin-1.jpg]

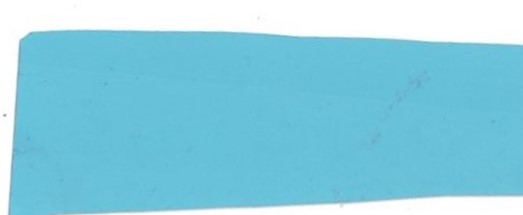

Supplement: S5 Data — (ZIP) [file ppat.1012014.s012.zip › D/1/Nuclear-Cap-1.jpg]

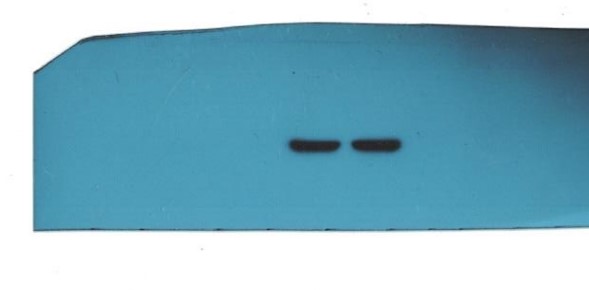

Supplement: S5 Data — (ZIP) [file ppat.1012014.s012.zip › D/1/Nuclear-Lamin B-1.jpg]

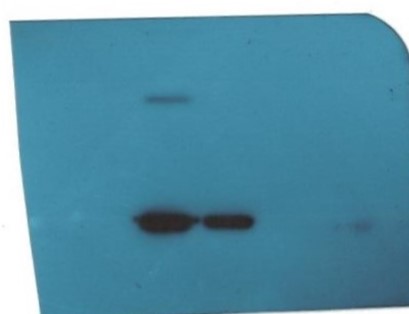

Supplement: S5 Data — (ZIP) [file ppat.1012014.s012.zip › D/1/Nuclear-NPM1-1.jpg]

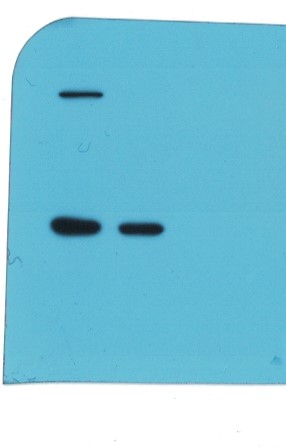

Supplement: S5 Data — (ZIP) [file ppat.1012014.s012.zip › D/2/pNPM1(Nuclear).jpg]

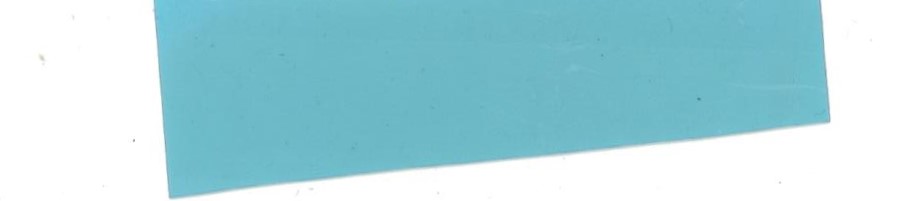

Supplement: S5 Data — (ZIP) [file ppat.1012014.s012.zip › D/2/S5-D-Cap(Cytoplasm).jpg]

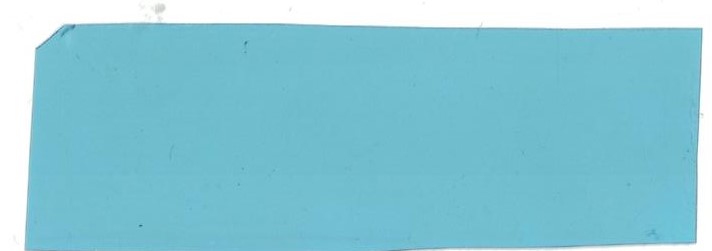

Supplement: S5 Data — (ZIP) [file ppat.1012014.s012.zip › D/2/S5-D-Cap(Nuclear).jpg]

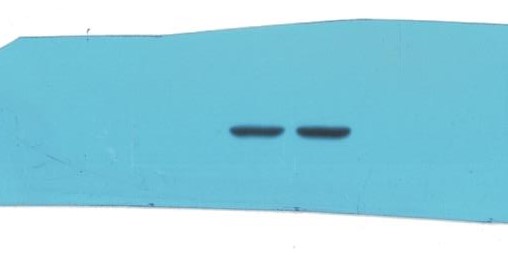

Supplement: S5 Data — (ZIP) [file ppat.1012014.s012.zip › D/2/S6-D-actin.jpg]

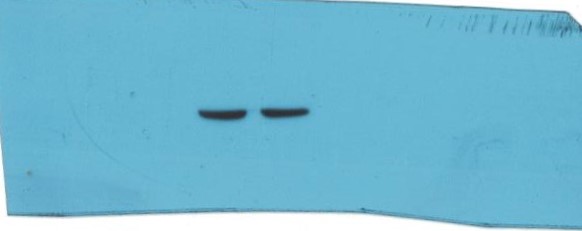

Supplement: S5 Data — (ZIP) [file ppat.1012014.s012.zip › D/2/S6-D-Lamin B.jpg]

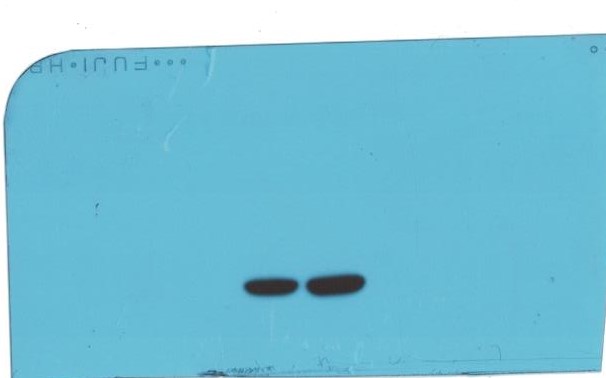

Supplement: S5 Data — (ZIP) [file ppat.1012014.s012.zip › D/2/S6-D-pNPM1(Cytoplasm).jpg]
